# Review: Insight on Porous Carbon Positive Electrode for Sodium‐Ion Capacitors: Interplay Between Synthesis, Properties, and Performance

**DOI:** 10.1002/advs.202523038

**Published:** 2026-02-24

**Authors:** Ademola Adeniji, Adrian Beda, Camélia Matei Ghimbeu

**Affiliations:** ^1^ Institut de Science des Matériaux de Mulhouse (IS2M) CNRS UMR 7361, Université de Haute‐Alsace Mulhouse France; ^2^ Université de Strasbourg Strasbourg France; ^3^ Réseau sur le Stockage Electrochimique de l'Energie (RS2E) CNRS FR3459 Amiens Cedex France

**Keywords:** biomass, (bio)polymer, porous carbon, sodium ion capacitor, specific capacity

## Abstract

Sodium ion capacitors (SICs) represent a major advancement in the field of energy storage technology. SICs combine the high energy of batteries with the high power of capacitors by pairing battery‐type (negative) and capacitor‐type (positive) electrodes, offering a cost‐effective alternative to lithium‐based systems. Since its inception in 2012, numerous research works have focused on the optimization of electrode materials, particularly the porous carbons (PCs) positive electrode material. PCs such as activated carbons are mostly adopted thanks to their good compatibility with high potential windows. However, the commercial viability of SICs is currently being limited by the low discharge capacity of the activated carbon positive electrode. This review provides an in‐depth analysis of how synthesis properties impact porous carbon properties (morphology, porosity, structure and surface chemistry), drawn from literature findings from comprehensive databases. It further establishes the synthesis‐property correlation with the electrochemical performance of PCs in Na metal half‐cell. Also, the charge storage mechanisms as related to capacitive and pseudo‐capacitive mechanisms were extensively examined. Additionally, pre‐sodiation strategies for building dual‐carbon SIC full cells are discussed, alongside the performance of PCs incorporating various carbon‐based negative electrode materials. Finally, it addresses the challenges and opportunities in improving PC performance toward SIC commercialization.

## Graphical abstract

Sodium ion capacitor (SIC) is currently constrained by the low discharge capacity of commercial activated carbon as positive electrode material. This review provides a holistic summary of research efforts on alternative porous carbon materials for SIC. Image created by the authors with www.biorender.com.

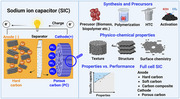

**Abbreviations**
AC: activated carbon
ACMT: activated carbon microtubes
ACN: acetonitrile
ASA: active surface area
BET: Brunauer–Emmett–Teller
CBCS: cuttlebones derived carbon sheets
CE: coulombic efficiency
CF: carbon fibers
CMC: carboxymethyl cellulose
CRPC: carbonyl rich porous carbon materials
CS: carbon monosulfide
CS_2_
: carbon disulfide
CV: cyclic voltammetry
CVD: chemical vapor deposition
DDS: 4, 4′‐diaminodiphenyl sulfones
DEC: diethyl carbonate
DEGDME: diethylene glycol dimethyl ether
DFT: density functional theory
DI: deionized water
DMC: dimethyl carbonate
DME: dimethyl ether
DMF: N,N‐dimethylformamide
DOS: density of states
EA: elemental analysis
EC: ethylene carbonate
ECD: electrochemical dilatometry
EDL: electric double layer
EDLC: electric double‐layer capacitance
EES: electrochemical energy storage
EMI‐TFSI: ethyl‐methylimmidazolium‐bis(trifluoro‐methane‐sulfonyl)imide
EQCM: electrochemical quartz crystal microbalance
ESW: electrochemical stability window
FEC: fluoroethylene carbonate
GCD: galvanostatic charge discharge
GCPL: galvanostatic cycling with potential limitation
GO: graphene oxide
Gr: graphite
HBC: hollow bowl‐like carbon
HC: hard carbon
HPCs: hierarchically porous carbons
HR‐TEM: high resolution transmission electron microscopy
HTC: hydrothermal carbonization
ICE: initial coulombic efficiency
KIC: potassium ion capacitors
LIB: lithium‐ion battery
LIC: lithium‐ion capacitor
MA: maleic acid
MAS: magic angle spinning
MCMS‐K: KHCO_3_‐activated micro‐/mesoporous carbon microspheres
MER: magnolol based resins
MICs: metal ion capacitors
MOF: metal–organic framework
MOs: metal oxides
Na_4_EDTA‐4H_2_O: tetrasodium ethylenediaminetetraacetate tetrahydrate
NaClO_4_
: sodium perchlorate
NaPF_6_
: sodium hexafluorophosphate
NaTFSI: sodium bis(trifluoro‐methane‐sulfonyl)imide
NDPC: nitrogen doped porous carbon
NHPAC: 3D nitrogen‐doped hierarchical activated carbon
NMR: nuclear magnetic resonance
N‐OMC: nitrogen doped ordered microporous carbon
NPC: N/O‐doped porous carbon
NS‐WDC: nitrogen/sulfur co‐doped watermelon‐derived porous carbon
OCP: open circuit potential
PA: polyaniline
PAN: polyacrylonitrile
PC: propylene carbonate
PCC: porous carbon cloth
PCHNS: porous carbon hollow nanospheres/nanosheets
PCNS: ultrathin porous carbon nanosheet 3d architectures
PCs: porous carbons
PMDA: pyromellitic dianhydride
PR: phenolic resin
PSRC: potato starch residue derived carbon materials
PTFE: poly(tetrafluoroethylene)
PVA: polyvinyl alcohol
PVB: polyvinyl butyral
PVP: polyvinylpyrrolidone
PZC: point of zero charge
RF: resorcinol‐formaldehyde
RGO: reduced graphene oxide
SBR: styrene‐butadiene rubber
SC: soft carbon
SEI: solid electrolyte interphase
SIB: sodium ion battery
SICs: sodium ion capacitors
SMP: sodium metal powder
SO: sulfur monoxide
SOPC: sulfur‐ and oxygen‐doped porous carbon
SSA: specific surface area
TE: tetraethylene glycol dimethyl ether
TEABF_4_
: tetraethylammonium tetrafluoroborate
TEM: transmission electron microscopy
TEOS: tetraethyl orthosilicate
TMCs: transition metal carbides
TPD‐MS: temperature‐programmed desorption coupled with mass spectrometry
UCF: ultrathin carbon film
VC: vinylene carbonate
XPS: X‐ray photoelectron spectroscopy
XRD: X‐ray diffraction
ZIC: zinc ion capacitor

## 1 Introduction

Energy consumption worldwide continuously relies on fossil fuels with market shares of more than 80% [1]. This excessive dependency on fossil fuels, accompanied by the ever‐increasing industrialization and population growth, resulted in aggravated global warming, a shortfall in energy, and planet pollution. Recognizing these challenges has driven the development of alternative energy sources and energy storage technologies. Notwithstanding, the utilization of renewable energy sources such as geothermal, wind, solar, and tidal still depends on fast and reliable electrochemical energy storage (EES) devices that provide efficient, high‐energy, and power density. In addition, the fast development of electronic devices and electric vehicles accelerated the development of efficient EES devices that boast of high energy density, high power density, and long‐term cycle life.

**FIGURE 1:**
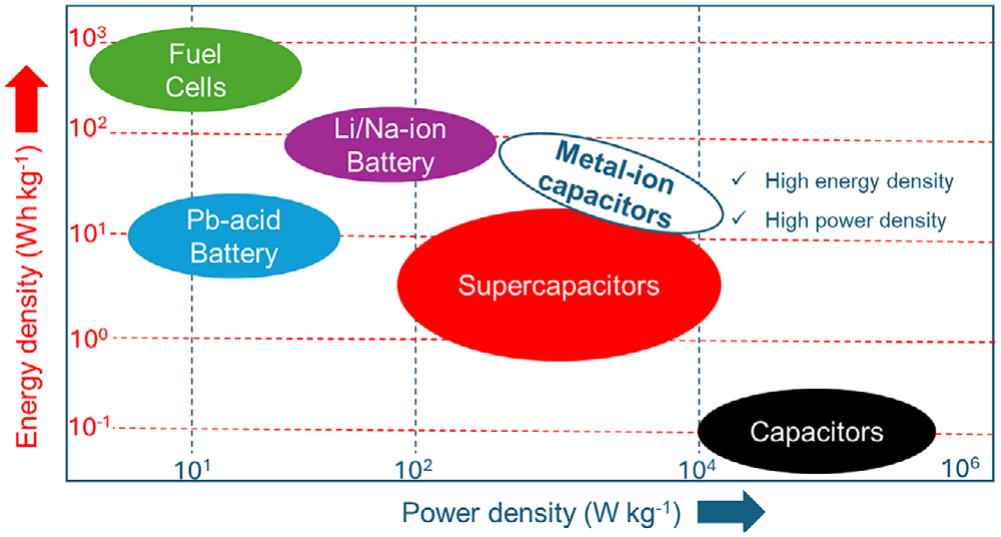
Ragone plot showing the various energy storage technologies.

Based on their energy and power density (Ragone plot), EES devices are typically classified into secondary batteries (Li/Na ion batteries and lead‐acid batteries), electrochemical capacitors, otherwise known as supercapacitors, and fuel cells (Figure 1). The advent and success of lithium‐ion batteries (LIBs) have seen them dominate the secondary battery markets thanks to their competitively high energy density (150–250 Wh Kg^−1^) [2, 3, 4] (Figure 1). However, they still suffer from low power density (<1000 W kg^−1^) and short cycling life (<2500 cycles) due to their slow faradaic reactions at both the negative electrode and positive electrode during the typical charge and discharge process, side reactions, and the solid electrolyte interface buildup [5]. In contrast, supercapacitor devices store charge based on the electric double layer (EDL) mechanism involving fast electrolytic ions adsorption and desorption during the charge and discharge process [6, 7, 8]. This rapid process, based on electrostatic interactions, results in high power density (up to 10 000 W kg^−1^, Figure 1) and extended cycle life (up to 100 000 cycles) [9, 10]. Nevertheless, they are still limited by their low energy density and fast discharge time. In this regard, both LIBs and supercapacitors are insufficient in achieving a combined high‐energy and power‐density device. The current limitation motivated the search for new energy storage systems that provide simultaneously high energy and power density.

The arrival of hybrid batteries, also called metal ion capacitors (MICs), represents a significant advancement in the field of energy storage devices. They boast of both high energy and power density, solving the issues encountered with conventional batteries and electrochemical capacitors by adopting materials from both devices. Typically, anodic materials such as graphite (Gr), hard carbon (HC), and metal oxides (MOs) are examples of negative electrode materials utilized in MICs, while the activated carbon electrode is the most common material employed as a positive electrode in electrochemical capacitors [11]. In the working principle of the MIC, intercalation or insertion of the cations is expected to take place in the negative electrode, while anion adsorption/desorption occurs on the positive electrode. Although the mechanisms are more complex, there can be adsorption of Li^+^/Na^+^ ions on the negative and positive electrode and ion/solvent co‐intercalation [12].

**FIGURE 2:**
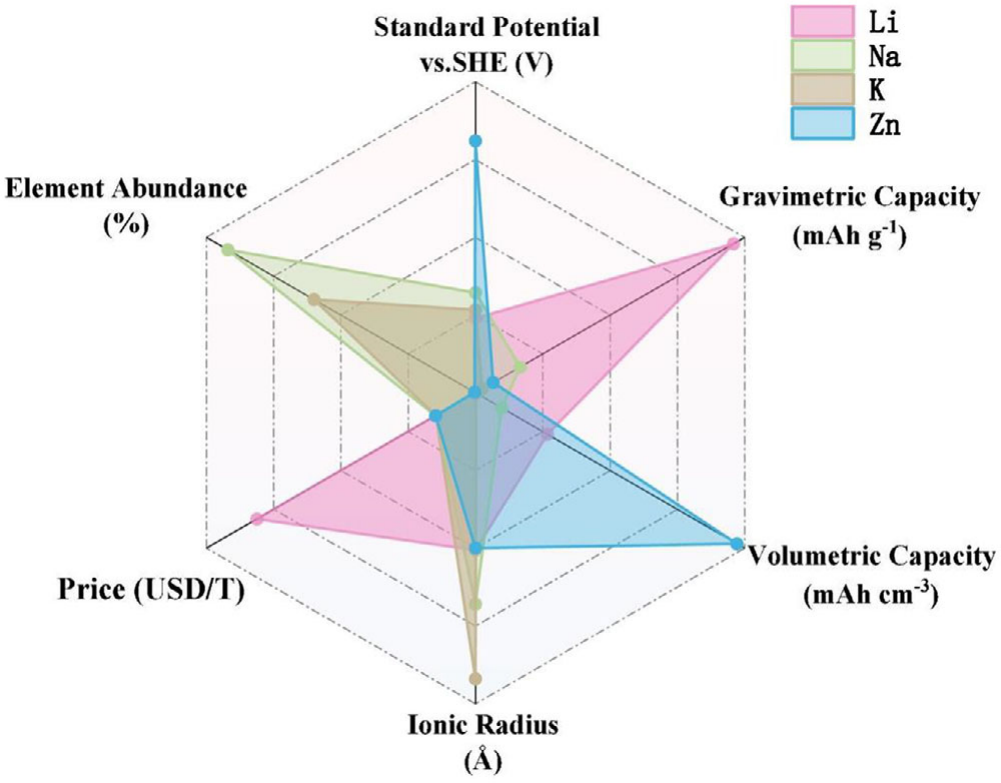
Schematic diagram comparing the performance of Li, Na, K, and Zn metals in terms of their elemental abundance, price, standard potential, gravimetric capacity, and volumetric capacity. Reprinted with permission [19]. Copyright (2024), Elsevier.

Li based chemistry is the most advanced technology for energy storage with a wide range of applications from transport to electronic devices. However, the uneven distribution, cost, and availability of Li metal and other battery (Ni, Co, and Gr) resources are estimated to be insufficient for the ever‐increasing energy consumption by 2040 [13]. This, therefore, requires the search for alternative chemistries. Numerous efforts have been made in this direction, including the development of Na [14], Ca [15], Mg [16], Zn [17], or Al ion [18] batteries. The abundance of Na and K metal resources compared to Li makes them great candidates for commercial applications and sustainability (Figure 2). However, Na stands out due to its closer chemical similarity to Li (ionic radii of Na^+^ (1.02 Å) vs. Li^+^ (0.76 Å) and standard redox potentials of Na/Na^+^ (−2.74 V) and vs. Li/Li^+^ (−3.04 V)), which allows for easy adoption of the existing LIC technology to sodium ion systems [19]. While K metals are also abundant, they are often limited by their low gravimetric capacity, making the adoption of potassium ion capacitor (KIC) still in its infant stage of research. Zinc ion capacitors (ZICs), on the other hand, also leverage the abundance of Zn resources and high stability in aqueous electrolytes. As a negative electrode material, Zn possesses a high theoretical (820 mAh g^−1^) and volumetric capacity (5851 mAh cm^−3^), however, its energy and power density are limited due to the water decomposition potential (1.23 V), thereby constraining its practical application [19]. Thus, Na‐ion‐based chemistry stands out as one of the most promising.

Electrode materials play a pivotal role in determining the electrochemical performance of energy storage devices, such as batteries, supercapacitors, and MICs. A typical MIC features a battery‐type negative electrode paired with a capacitor‐type positive electrode in a metal‐ion electrolyte. In recent SIC research, a wide range of negative/positive electrode materials have been investigated, including carbon‐based materials, metal oxides, MXenes, and metal phosphides [19, 20, 21]. Among these, dual carbon‐based electrodes have garnered significant attention for SICs owing to several compelling advantages: they largely avoid transition metals, thereby lowering costs and alleviating supply‐chain risks [22]. They can be produced from abundant, locally available biowaste or inexpensive carbon precursors, providing excellent tunability of porosity, straightforward surface chemistry modification, and outstanding thermal and chemical stability [21]. The pore structure of these carbons profoundly affects electrochemical performance, enabling simultaneous achievement of high energy and power densities [23].

In dual‐carbon SICs, commonly used electrode materials include hard carbon, soft carbon, and various nanoporous carbon. Hard carbon has emerged as a particularly promising negative electrode material, delivering discharge capacities >300 mAh g^−1^ [14]. Nanoporous carbons, in contrast, are widely employed as positive electrode materials because of their low cost, diverse precursor options, highly tunable physicochemical properties, and compatibility with wide voltage windows. When derived from biomass, biopolymers, or synthetic sources, these carbons typically exhibit high specific surface area, hierarchical porosity, and controllable surface functional groups. However, conventional activated carbons used as positive electrodes generally offer limited discharge capacities (≤100 mAh g^−1^) [23]. This low positive electrode capacity becomes the bottleneck that severely restricts the overall cell capacity of full SIC devices.

In the last decade, there has been a lot of work reporting the synthesis of new porous carbon materials as a positive electrode for SIC, with the aim geared toward improving their performance. This review provides a holistic summary of the different porous carbon materials reported between 2012 and 2025. Several reviews have been published on sodium ion capacitors, and have shine light on the broader insight on sodium ion capacitors [11], charge storage mechanisms [19, 20, 24], materials design strategies (negative electrode, positive electrode and electrolytes) [19, 25, 26], carbonaceous materials (negative electrode and positive electrode) [21, 22, 27], and pre‐sodiation technology (negative electrode, positive electrode and electrolyte) [28], and even recently on the value chain of dual carbon SIC [29]. Differently, this review attempts to provide a comprehensive insight into the synthesis methods of porous carbon and how their textural/structural properties and surface chemistry affect their electrochemical performance as a positive electrode of SICs. It first starts with the broad family of precursors utilized for porous carbon positive electrode and their synthesis conditions by studying more than 100 research articles. Then, it examines porous carbon morphology, structural properties, texture, surface chemistry, and defects, and their impact on electrochemical performance, including specific capacity, influenced by textural properties, mass loading, binders, and electrolytes. Charge storage mechanisms, such as capacitive and pseudocapacitive processes, ion solvation, and ageing, are analyzed alongside pre‐sodiation concepts and SIC full‐cell performance with carbon negative electrodes. Last, this review provides a clear picture of the efficient and optimal engineering of porous carbon positive electrodes to improve their performance towards achieving a high‐performance SIC.

## 2 Basic Concept

This section explores the various possible configurations of SICs based on their charge storage mechanisms at the electrodes. Different combinations of battery‐type, capacitive‐type, and pseudocapacitive electrodes offer distinct advantages in terms of energy and power density trade‐offs. Particular attention is given to the working principle of dual carbon SICs, which utilize carbon‐based materials for both electrodes. Furthermore, this section examines the performance characteristics of commercial activated carbons, which are widely used in SIC applications. The specific capacity of these porous carbon materials plays a critical role in determining the overall device performance, making the selection and optimization of activated carbons a key consideration in SIC design and development.

### 2.1 SIC Configurations

**FIGURE 3:**
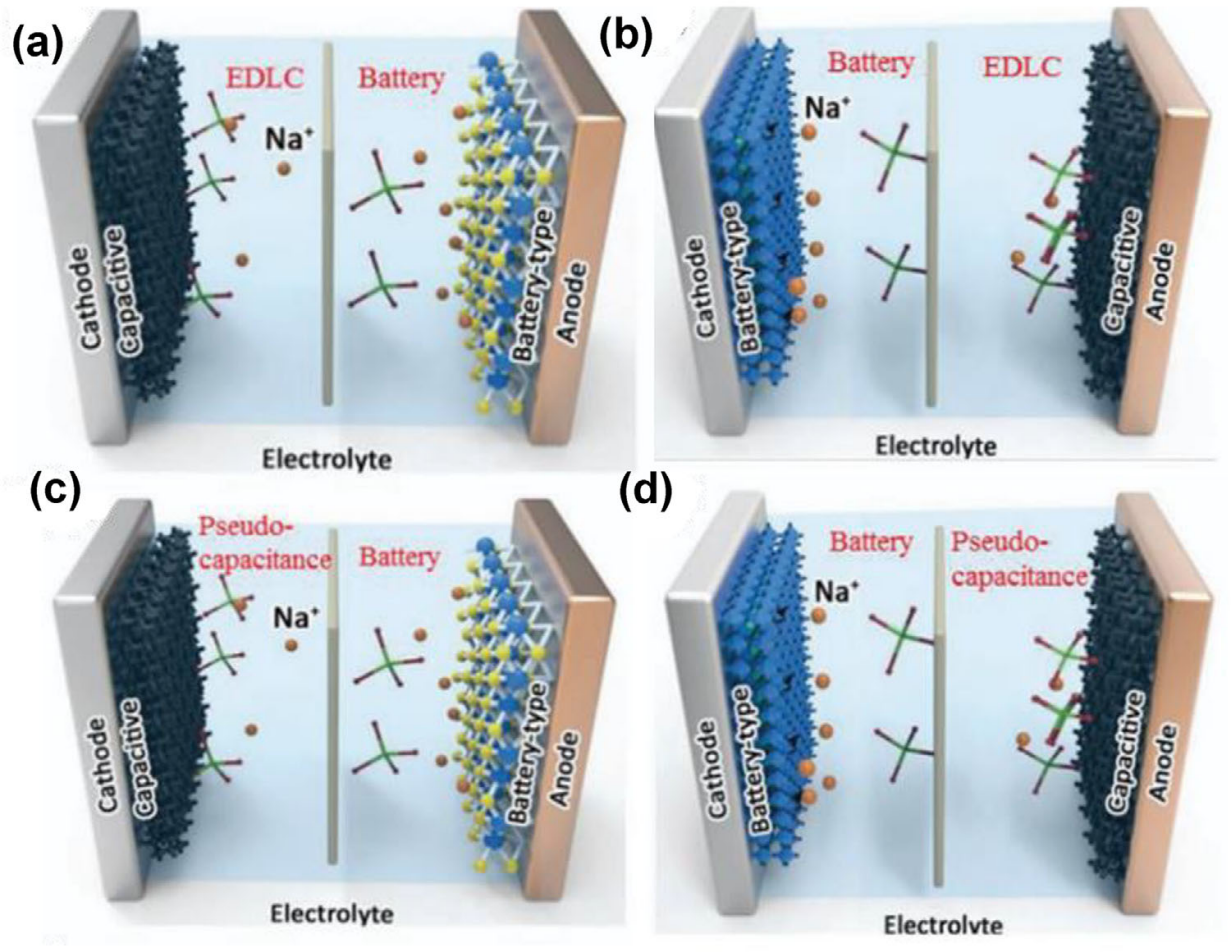
Schematic diagram of the cell structure of SIC electrochemical energy storage devices: (a) battery‐type negative (negative electrode) and capacitive positive (positive electrode) cell configurations, (b) battery‐type positive electrode and capacitive type negative electrode configuration, (c) battery‐type negative electrode and pseudocapacitance type positive electrode configuration, and (d) battery‐type positive electrode and pseudocapacitance type negative electrode configuration. Reprinted with permission [20]. Copyright (2021), John Wiley and Sons.

The working mechanism of SICs can be classified into several configurations consisting of an asymmetric electrode pair (Figure 3).

#### 2.1.1 Battery‐Type Negative Electrode and Capacitive Type Positive Electrode

SICs typically employ a battery‐type negative electrode (negative electrode, called also anode) and a capacitive positive electrode (positive electrode, called also cathode) (Figure 3a and Table 1). During charge/discharge, the negative electrode undergoes faradaic processes involving ion insertion/extraction, while the positive electrode relies on non‐faradaic adsorption/desorption of ions at the surface. This asymmetric configuration combines the high energy density of battery‐type negative electrodes with the high‐power density and cycling stability of capacitive positive electrodes. The battery‐type negative electrodes can be classified into three primary categories based on their sodium storage mechanisms: intercalation‐type, conversion‐type, and alloying‐type materials. Each mechanism exhibits distinct electrochemical characteristics, reaction kinetics, and volume changes, which significantly influence the performance of both half‐cells and full SIC devices (Table 1).

**TABLE 1:** A summary of SICs device configurations. Reproduced with permission [20]. Copyright (2021), John Wiley and Sons.

Battery‐type negative electrode and capacitive type positive electrode							
S/N	Negative/Positive electrodes	Assembly method	Electrolyte	Potential window (V)	Max ED (Wh kg^−1^)	Max PD (W kg^−1^)	Cycling
1	Hard carbon/AC [33]	Two‐step	1 M NaPF_6_ in 1:1 (EC:PC)	1.5–4.2	>100	7000	70% at 5000 cycles; 2 A g^−1^
2	Na_2_Ti_3_O_7_/graphene foam/ graphene foam [34]	One step	Na‐ion conducting gel polymer electrolyte	1.0–3.0	70.6	4000	73.2% at 5000 cycles; 0.5 A g^−1^
3	NiCo_2_O_4_/AC [35]	One step	1.5 M NaClO_4_ in 1:2 (PC:DMC)	0–4.5	23.5	308	61.2% at 2000 cycles; 0.15 A g^−1^
4	MoO_2_@rGO/AC [36]	One step	0.75 M NaPF_6_ in 1:1 (EC:DEC)	0.01–3.0	79	3300	78% at1000 cycles; 0.07 A g^−1^
5	TiSb_2_/AC [37]	One step	1.5 M NaClO_4_ in 1:1 (EC:DEC) with 5 wt.% FEC	1.0–3.6	72	20625	63%at 1000 cycles; 5 A g^−1^

Capacitive‐ type negative electrode and battery‐type positive electrode							
1	AC/Graphite [38]	One step	1 M NaPF_6_ in 6:4 (EC:DMC)	0–4.0	108	6100	97% at 5000 cycles; 0.25 A g^−1^
2	AC/NaMn_1/3_Co_1/3_Ni_1/3_PO_4_ [38]	One step	1 M NaPF_6_ in 1:1 (EC:DEC:DMC)	0–3.0	50	750	92% at 5000 cycles; 0.12 A g^−1^
3	PCNF/CNF@NVPF [39]	One step	Na‐ion conducting gel polymer electrolyte	1.0–4.3	227	4936	80% at 1000 cycles; 0.2 A g^−1^
4	AC/Na_0.5_Mn_0.5_Co_0.48_Mg_0.02_O_2_ [40]	One step	1.0 M NaClO_4_ in 1:1 PC with 5 wt.% FEC	0–3.0	35	150	72% at 3000 cycles; 1 A g^−1^
5	CDC/C‐NVP [41]	One step	1.0 M NaClO_4_ 1:1 (EC:PC)	0–3.0	118	850	95% at 10 000 cycles; 0.12 A g^−1^

Battery‐type and pseudocapacitive ‐type as negative or positive electrode							
1	HC/V_2_C‐MXene [42]	One step	1 M NaPF_6_ in 1:1 (EC:PC)	1 M NaPF_6_ in 1:1 (EC:PC)	35	1500	70% at 3000 cycles; 1 A g^−1^
2	HC/Fe‐V‐O [43]	Two‐step	1 M NaPF_6_ in DEGDME	0–3.8	194	3942	67.2% at 800 cycles; 1 A g^−1^
3	Ti_2_C‐MXene/Na_2_Fe_2_(SO_4_)_3_ [44]	One step	1 M NaPF_6_ in 1:1 (EC:DEC)	0.1–3.8	320	360	96% at 100 cycles; 0.6 A g^−1^
4	Ti_3_C_2_‐MXene/Na_0.44_MnO_2_ [45]	One step	1.0 M NaClO_4_ in 1:1 PC with 5 wt.% FEC	0–4	—	—	85% at 60 cycles; 0.05 A g^−1^

Carbon battery‐type negative electrode and capacitive‐ type positive electrode (Dual carbon SIC)							
1	Nanoporous disordered carbon/N‐hollow microspheres [46]	One step	Na‐ion conducting gel polymer electrolyte	0–4.4	157	2356	70% at 1000 cycles; 0.3 A g^−1^
2	hard carbon (GDHC) //porous carbon (GDPC) [47]	Two‐step	1.0 M NaClO_4_ in 1:1 EC:DEC	1.5–4.2	156	38910	73% at 5000 cycles; 30 A g^−1^
3	S‐doped nanoparticles/highly porous nanoparticles [48]	Two‐step	1.0 M NaClO_4_ in 1:1 EC:DEC	1–4	106	25000	76% at 10000 cycles; 2 A g^−1^
4	Carbon nanosheets/activated carbon nanosheet [49]	Two‐step	1.0 M NaClO_4_ in 1:1 EC:DEC	0–4.0	112	12000	85% at 3000 cycles; 5 A g^−1^
5	N, itrogen‐doped microporous hard carbon/soft carbon	Two‐step	1 M NaPF_6_ in 6:4 (EC:DMC)	0.01–4.7	245.7	13846	1000 cycles; 5 A g^−1^

#### 2.1.2 Capacitive‐Type Negative Electrode and Battery‐Type Positive Electrode

SIC employing a capacitive negative and a battery‐type positive are often referred to as “upside‐down” hybrid configurations (Figure 3b and Table 1). In these systems, the roles of the electrodes are reversed compared to the classic SIC configuration previously discussed: a capacitor‐type material serves as the negative electrode, while a battery‐type material functions as the positive electrode. Typically, activated carbon (AC) is used as the capacitive negative electrode, exhibiting the characteristic triangular potential profile of an ideal electrical double‐layer capacitor during charge–discharge cycles. In contrast, the battery‐type positive electrode is made of a faradaic material that delivers higher specific capacity and displays a relatively flat voltage plateau at a higher potential. As in classic SICs, the overall cell voltage is determined by the potential difference between the battery‐type positive electrode and the capacitive negative electrode. This “upside‐down” design still yields a hybrid charge–discharge profile that combines capacitive and battery‐like behavior, often resulting in a more linear or quasi‐triangular overall voltage curve. Because suitable battery‐type materials that can operate stably as positive electrodes in this configuration are relatively limited, research and development efforts have primarily focused on optimizing and exploring new positive electrode materials for these upside‐down SICs.

#### 2.1.3 Pseudocapacitive Type/Battery Type as Negative Electrode or Positive Electrode

Another typical configuration for sodium‐ion capacitors (SICs) pairs a battery‐type electrode with a pseudocapacitive electrode (either as a negative or positive electrode) as shown in Figure 3c,d and Table 1. Ti_3_C_2_T_x_ MXene, a layered transition metal carbide, is a representative pseudocapacitive material that primarily stores charge through Faradaic redox reactions, with only a small contribution from electric double‐layer capacitance. Despite this dominant Faradaic mechanism, MXene exhibits highly capacitor‐like electrochemical behavior. This, combined with its much higher specific surface area and capacitance compared to activated carbon, makes it an attractive electrode material. However, MXene‐based SICs are frequently constrained by either low energy density or low power density, limiting their overall performance.

### 2.2 Working Principle of Dual Carbon SIC

In the last decades, there has been a major shift in focus towards the search for new materials for sodium ion chemistry, in particular for sodium ion battery (SIB). The low efficiency of graphite (the reference material for LIB negative electrode) to insert Na‐ions [14, 30, 31], has seen the adoption of hard carbon materials, which excellently favor the insertion and de‐insertion of the Na^+^ ion with improved charge capacity. Although the fast development of LIB has led to increased research in lithium‐ion capacitors.

**FIGURE 4:**
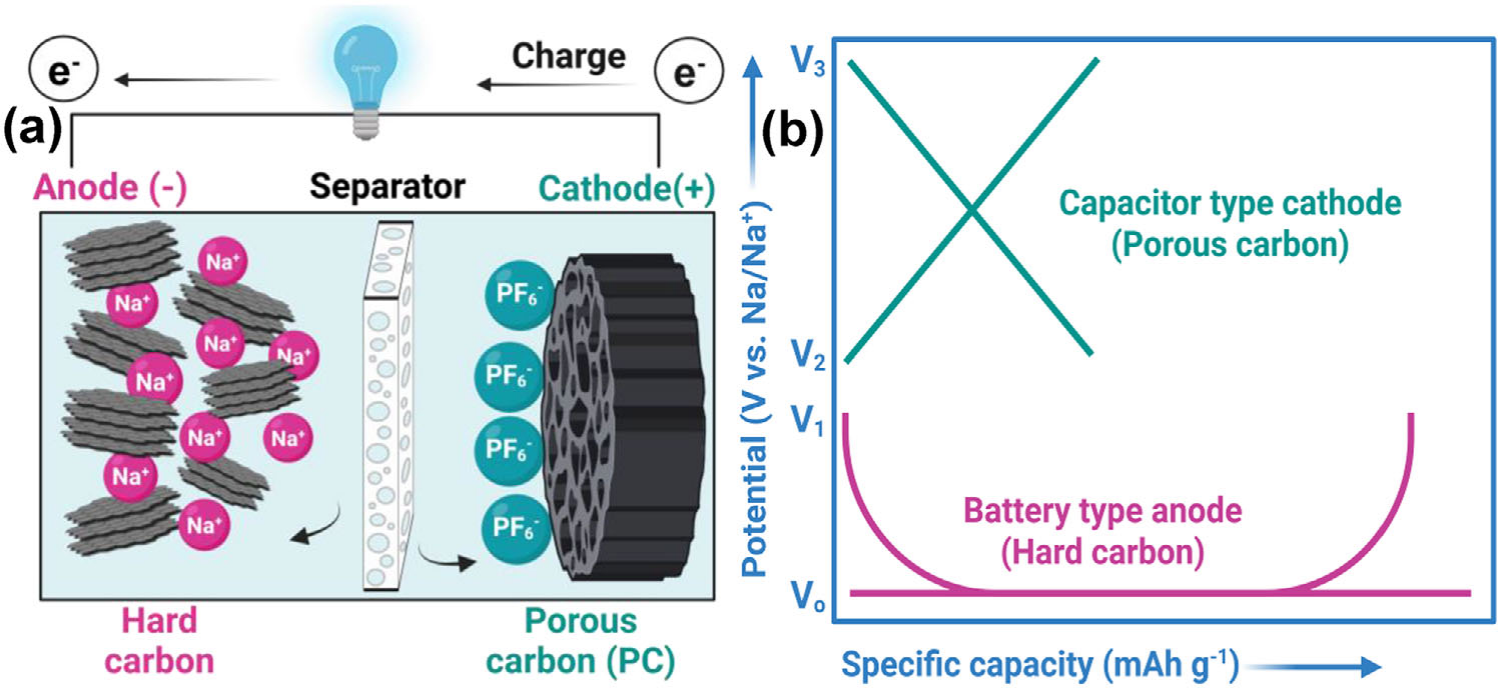
(a) A dual carbon sodium ion capacitor full cell consisting of hard carbon negative electrode and porous carbon (PC) positive electrode. Created by the authors using biorender.com and (b) typical electrochemical behavior of an ideal capacitor‐type positive electrode (cathode) and battery‐type negative electrode (anode) half‐cell vs. Na metal, Created by the authors with biorender.com.

Among the various SIC configurations, the dual‐carbon design, featuring a battery‐type carbonaceous negative electrode and an electric double‐layer capacitor‐type positive electrode, is considered a classical architecture. Initially demonstrated in 2012 [32], the first SIC utilized hard carbon (HC) as the negative electrode due to its low sodiation potential plateau (vs. Na^+^/Na), high reversible capacity, and excellent structural stability, as shown in Figure 4. Since then, HC has remained the benchmark battery‐type negative electrode material for SICs. Paired with activated carbon or other high‐surface‐area capacitive positive electrodes, where cations and anions adsorption/ desorption takes place, as illustrated in Figure 4.

Dual‐carbon SICs, which employ carbonaceous materials as both the negative and positive electrodes, are the most prevalent configuration of SICs. They deliver significantly higher energy and power densities (Table 1) compared to other SIC architectures. These devices are typically assembled using a pre‐sodiated carbon‐based negative electrode (such as HC) that stores charge via a battery‐like mechanism (intercalation/deintercalation of Na^+^ ions) and a high‐surface‐area carbonaceous positive electrode that operates through a capacitive mechanism (adsorption/desorption of anions), all within a sodium‐salt electrolyte that facilitates ion transport. The charge‐storage process is electrolyte‐consuming. During charging, Na^+^ cations migrate to the negative electrode, where they undergo intercalation or reduction, while anions are adsorbed onto the positive electrode surface, similar to conventional supercapacitors. Upon discharge, anions desorb from the positive electrode, and Na^+^ ions de‐intercalate from the negative electrode, returning to the electrolyte.

Pre‐sodiation of the negative electrode is essential for carbon‐based systems. It compensates for irreversible Na^+^ consumption in the first cycle, prevents depletion of sodium ions from the electrolyte, and ensures sufficient free Na^+^ ions are available across a wide operating voltage window, thereby improving capacity, cycling stability, and overall performance. Dual‐carbon SICs are inherently clean and environmentally friendly throughout their lifecycle, from manufacturing to end‐of‐life disposal. They rely primarily on sodium‐ion shuttling with minimal or no dependence on transition metals in the electrodes, which lowers costs, reduces supply‐chain risks, and eliminates reliance on scarce or critical metals. Carbon electrodes can be produced from abundant, low‐cost precursors such as biowaste or inexpensive synthetic sources, further enhancing economic and sustainability benefits. Moreover, inexpensive and abundant aluminum can be used as the current collector for both electrodes, since sodium does not alloy with aluminum at room temperature.

### 2.3 Performance of Commercial Porous Carbons

Porous carbons, such as carbon nanospheres, carbon nanofibers, carbon nanosheets, and hierarchically disordered carbons, are increasingly used as the capacitive positive electrode for the dual carbon SIC due to several key advantages:

1. High Surface Area and Tunable Porosity: Porous carbon materials offer very high specific surface areas and finely tuned pore sizes that match the size of desolvated sodium or electrolyte anion ions, which maximizes ion adsorption/desorption. This enhances the capacitive behavior and overall charge storage capacity of the electrode.
2. Good Electrical Conductivity and Graphitization: The pseudo‐graphitized structure of some porous carbon ensures high electronic conductivity, which enables fast charge transfer and good rate capability for high power output.
3. Structural and Surface Chemistry Control: Nitrogen doping, oxygen‐containing groups, and structural defects in porous carbon offer enhanced pseudocapacitive interactions, improving specific capacity and cycling stability in SICs.
4. Mechanical Stability and Cycle Life: Porous carbons tend to be robust, supporting stable cycling over many charge‐discharge cycles without significant capacity fade, essential for long‐lived SIC devices.
5. High voltage window: Porous carbons tend to operate in wide voltage windows, allowing high energy and power density.
6. Lightweight and Abundance: Carbon is lightweight and widely available, making it a practical and cost‐effective choice for SIC positive electrodes.

Thus, porous carbon provides an optimal combination of high surface area, tailored pore size, good electrical conductivity, stable surface chemistry, and mechanical robustness, making it the preferred positive electrode material in SICs. Ever since, researchers have focused on advancing this field with new electrode materials and electrolytes. Despite the huge potential of SIC in providing a high energy and power density, one of its lingering problems is the poor discharge capacity of the porous carbon positive electrode materials. Hard carbon materials have been reported to achieve a high discharge capacity >300 mAh g^−1^ [14]. Activated carbon, on the other hand, has a discharge capacity of ≤100 mAh g^−1^. Thus, the total capacity ($\frac{1}{C_{T}}=\frac{1}{C_{anode}}+\frac{1}{C_{cathode}}$) of the full SIC cell suffers from the positive electrode's low capacity.

**FIGURE 5:**
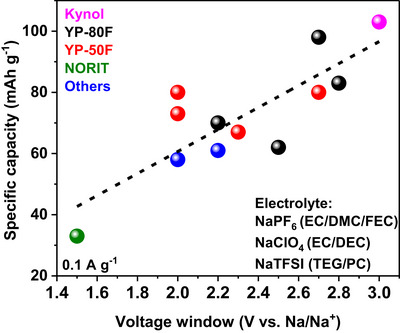
Diagram showing the electrochemical performance of commercial activated carbons vs. Na metal half‐cell. The plot was made based on literature references presented in Table 2.

Till date, several commercial activated carbons including YP‐80F [50, 51, 52, 53], YP‐50F [54, 55], Norit [56], Kynol [23], and others [57, 58], have been tested in cathodic half cells vs. Na metal in different electrolyte salts such as sodium hexafluorophosphate (NaPF_6_), sodium perchlorate (NaClO_4_), and sodium bis(trifluoromethanesulfonyl)imide (NaTFSI) which are typically dissolved in organic solvents including ethylene carbonate (EC), diethyl carbonate (DEC), propylene carbonate (PC), dimethyl carbonate (DMC), diethylene glycol dimethyl ether (DEGDME), and tetraethylene glycol dimethyl ether (TEGDME) and electrolyte additives such as vinylene carbonate (VC) and fluoroethylene carbonate (FEC). The discharge specific capacity of these materials remains ≤ 100 mAh g^−1^ even at very low current density 0.05 A g^−1^ (Figure 5), although many of these materials possess a very high specific surface area (SSA). Liu et al. [51]. measured the BET surface area of YP‐80F to be 2708 m^2^ g^−1^ with an average pore size of 1.68 nm. These activated carbons are also able to reach a low potential down to 1.5 V and a high potential up to 4.5 V (Table 2). Despite the notable textural and structural properties, the electrochemical performance still falls short of expectations.

**TABLE 2:** Summary of commercial activated carbon (CAC) performance in positive electrode half‐cell versus Na metal half‐cell.

S/N	Activated Carbon	Properties	Electrolyte	Potential Window (V)	Maximum Specific Capacity (mAh g^−1^)	Current Density (A g^−1^)	Refs.
1	YP‐80F (Kuraray)	n.a	0.5 M NaPF_6_ in DEGDME (1/1 vol%)	1.5–4	∼62	0.05	[50]
		SSA: 2708 m^2^ g^−1^ Pore size: 1.68 nm	1 M NaClO_4_ in EC/DEC (1/1 vol%)	1.5–4.2	∼98	0.05	[51]
		SSA: 2300 m^2^ g^−1^	1 M NaClO_4_ in EC/DEC (1/1 vol%)	2–4.2	70	0.1	[52]
		n.a	1M NaPF_6_‐TEGDME	1–3.8	83	0.05	[53]
2	YP‐50F (Kuraray)	n.a	1 M NaTFSI in TEG:PC (3/7, wt%)	2–4	80 F g^−1^	0.1	[65]
			1 M NaPF_6_ in EC:PC (1/1, wt%)		73 F g^−1^		
		SSA: 1682 m^2^ g^−1^	1 M NaPF_6_ in VC:DMC (50/50, vol%)	1.5–4.2	∼80	0.1	[54]
		SSA: 1343 m^2^ g^−1^ Pore size: 0.6–2.3 nm	1 M NaClO_4_ in (EC/PC) (1/1, vol%) with 5 wt.% FEC	2–4.3	∼67	0.5	[55]
3	AC (Norit)	n.a	1 M NaClO_4_ in EC/DEC (1/1 vol%)	2.7–4.2	∼33	0.1	[56]
4	Kynol‐5092‐20 (Kynol)	SSA: 2019 m^2^ g^−1^	1 M NaPF_6_ in EC:DMC (1:1), vol%)	1.5–4.5	103	0.1	[23]
5	Others CAC	SSA: 1356 m^2^ g^−1^	1 M NaClO_4_ in (EC/DMC) (1/1 vol%) with 5 wt.% FEC	2.5–4.5	58	0.1	[57]
		n.a	1 M NaClO_4_ in EC/DEC (1/1, vol%)	2–4	∼61	0.05	[58]

n.a., not applicable

The reason for the poor performance of commercial activated carbon could be as a result of the cumulative effect of their textural, structural, and surface chemistry, such as non‐homogeneous pore size distribution [59], incompatible pore size distribution/tortuosity (pore size, ratio micro/mesopores, and pore architecture) with the electrolytes' ion size [60, 61], unfavorable pore structure, which limits charge propagation [62], and unsuitable surface chemistry restricting wettability with the electrolyte or favoring electrolyte degradation [63, 64]. One way to improve the performance is to use a high‐voltage electrolyte, which increases the potential window and thus the specific capacity (Figure 5). However, there is a risk of property destruction (porosity, structure, and surface chemistry) that could affect the long‐term cycling performance of the SIC device.

In light of these challenges and opportunities for specific capacity enhancement, it is essential to accurately quantify and compare the electrochemical performance of porous carbon electrodes using standardized metrics. To express the performance of a porous carbon positive electrode half‐cell in galvanostatic charge‐discharge (GCD) or galvanostatic cycling with potential limitation (GCPL) experiment, it is recommended to use specific capacity, denoted as *C_specific_*, with units of milliampere‐hours per gram (mAh g^−^^1^). This unit allows for a standardized and fair comparison of material performance across different studies in the literature. It is calculated using the formula:

(1) $$C_{specific}\left(\frac{mAh}{g}\right)=\frac{2I}{3.6\times V\times m}\int_{t1}^{t2}Vdt$$

where *I* is the discharge/charge current (mA), *t* is the time (h), and *m* is the mass of active material (*g*). The specific capacity of the porous carbon electrode half‐cell from the cyclic voltammetry (CV) experiment may be evaluated as given below:

(2) $$C_{specific}\left(\frac{mAh}{g}\right)=\frac{1}{v\times m\times 3.6}\int_{V1}^{V2}IdV$$

where *m* is the mass of the active material in *g*, *v* is the scan rate in volt/s, and $\int_{V1}^{V2}IdV$ is the integral area of the CV curve in mAV.

## 3 From Precursor to Porous Carbon

**FIGURE 6:**
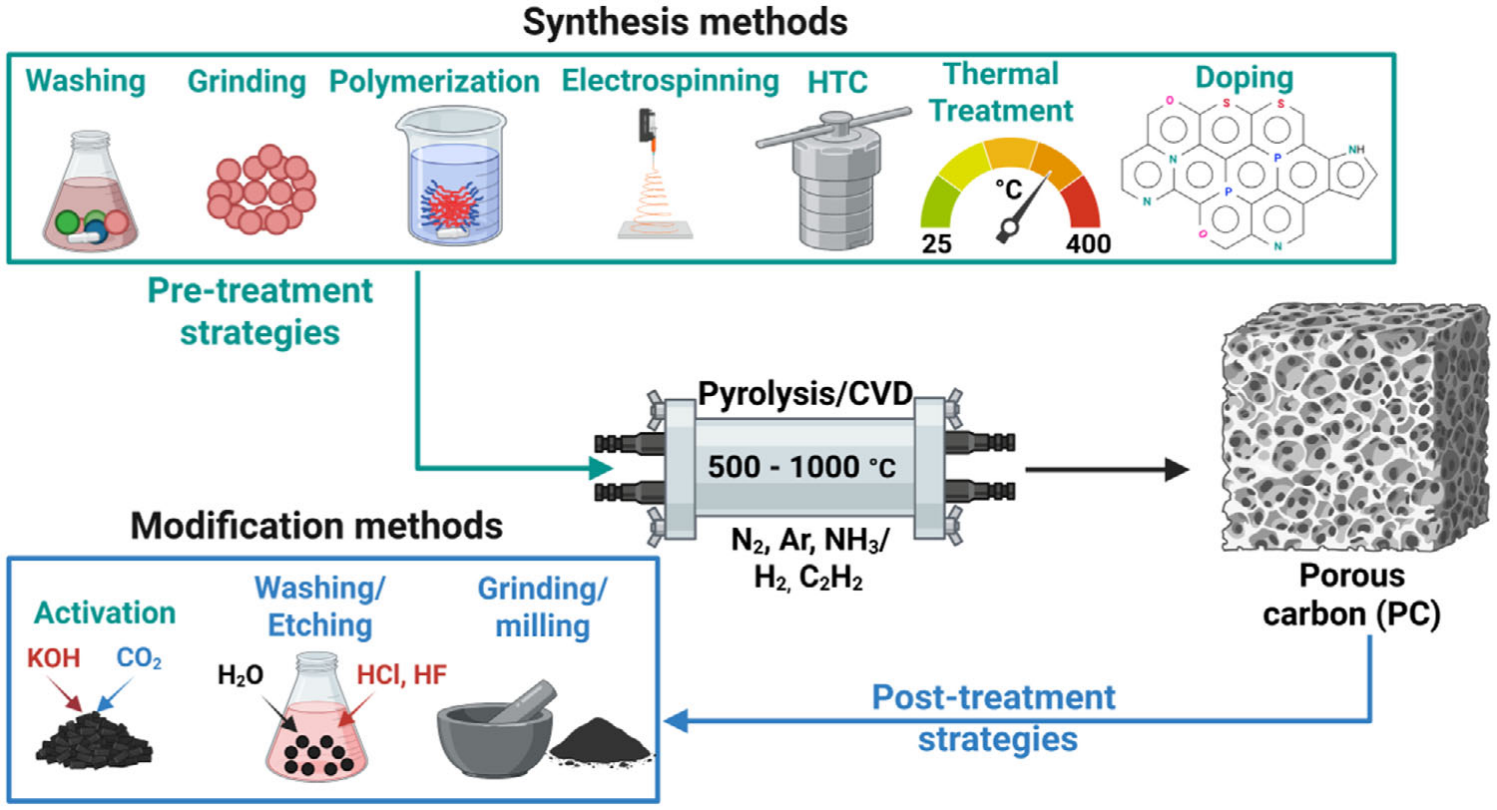
Schematic representation of porous carbon synthesis based on pyrolysis, pre‐and/or post‐treatment processes. Created by the authors with www.biorender.com.

This section discusses the different preparation strategies and processes for producing porous carbon, as shown in Figure 6, using various precursors. The process begins with pre‐treatment strategies, including washing, grinding, followed by synthesis via diffrent routes such as polymerization, electrospinning, and hydrothermal carbonization (HTC), and subsequent thermal treatment (pyrolysis). Post‐treatment strategies such as washing/etching and grinding/milling, along with additional steps like activation and heteroelements doping are also included.

### 3.1 Precursor Selection

The search for advanced materials for porous carbon development has necessitated the utilization of a variety of precursors, with each falling into the broad family of either crude biomass, biopolymer, or synthetic. These various precursors offer porous carbon synthesis opportunities in engineering and optimizing their textural, structural, and surface chemistry properties.

**FIGURE 7:**
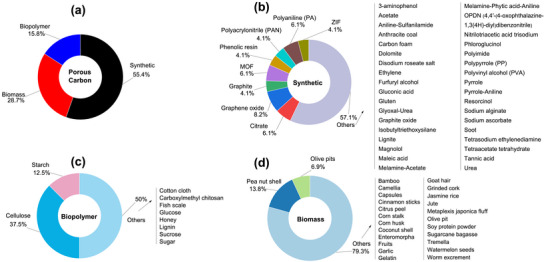
(a) Graphical representation of the various categories of precursors utilized in the synthesis of porous carbon materials. The percentage values are derived from the publications analyzed in Tables 3, 4, 5; Distribution of precursors (%) across three primary categories, along with the raw materials associated with each precursor class: (b) synthetic, (c) biopolymer, and (d) biomass.

As shown in Figure 7a, synthetic precursors account for more than half of the precursors explored as porous carbon positive electrode in SIC. One of the primary benefits of synthetic precursors is in the aspect of using high‐purity chemicals with relatively high carbon yield (>20%) [66], though the carbon yield is less reported for porous carbons in SIC publications. Synthetic precursors offer the opportunity to precisely control the porous carbon microstructural and morphological properties with a variety of materials available. Commonly utilized synthetic materials in research publications include synthetic polymers (Polyaniline, PA [67, 68]; Polyacrylonitrile, PAN [43, 69]; and Phenolic Resin, PR [23, 70]), carbon materials (Graphite/Graphene/Graphene Oxide [71, 72, 73, 74]), and others (Metallic Citrate [75, 76, 77], and Metal Organic Framework (MOF) [78, 79, 80]) as shown in Figure 7b and Table 3. One major drawback of synthetic precursors is that their production often relies on petrochemical‐derived materials and can involve hazardous chemicals, posing risks to both human health and the environment; however, more sustainable alternatives have been proposed to mitigate these issues.

**TABLE 3:** Literature review of porous carbon materials derived from synthetic precursors and their synthesis parameters.

S/N	Carbon source	Synthesis methods	Activating agent	Carbonization Temperature (°C)	Heating Rate,°C min^−1^ (gas)	Washing method	SSA (m^2^ g^−1^)	V_micro_ (cm^3^ g^−1^)	V_meso_ (cm^3^ g^−1^)	Ref.
1	C_2_H_2_, Ethylene/Nickel‐copper nanoparticles coated polyester fabric	Annealing of Nickel‐copper nanoparticles coated polyester fabric at 600°C under Ar flow	n.a.	555–600	n.a. (Ar and H_2_)	FeCl_3_, HCl and distilled H_2_O	346	0.2	0.05	[119]
2	Pristine graphite	Modified Hummer's method for reduced graphene oxide preparation using pristine graphite mixture with H_2_SO_4_, KMnO_4_, NaNO_3_ and H_2_O_2_ solution	n.a.	200–600	n.a. (N_2_)	n.a.	146	0.025	0.02	[72]
3	Graphene oxide/urea/thiourea	n.a.	n.a.	550	5(Ar)	n.a.	n.a.	n.a.	n.a.	[120]
4	Gluten	Salt templating mixture of potassium chloride, ball milled potassium carbonate, and gluten.	KCl	850 (Repeat of carbonization after washing)	5(N_2_)	Hot distilled H_2_O	2630	n.a.	n.a.	[121]
5	Sodium ascorbate	Pre carbonization at 700°C under argon atmosphere, grinding, washing with 2 M HCl, deionized H_2_O and ethanol.	KOH	800	n.a.(Ar)	2 M HCl and deionized H_2_O	1230	0.4	0.3	[122]
6	Metal Azolate Framework (MAF)	Dissolution of Zn(OH)_2_ in NH_4_OH, 2‐ethylimidazole, ethanol and cyclohexane. The obtained powder was mixed with urea and ethanol and sonicated.	KOH	800	n.a.(Ar)	Dilute HCl (10%)	5214	1.7	1.11	[80]
7	Potassium citrate	n.a.	n.a.	800	5(N_2_)	1 M HCl and deionized H_2_O	1398	n.a.	n.a.	[75]
8	Diesel soot	n.a.	KOH	650	2(n.a.)	HCl and distilled H_2_O	966	n.a.	n.a.	[123]
9	Poly(vinyl alcohol), PVA	Electrospinning of solution containing PVA powders, NH_4_VO_3_ and PTFE solution dissolved in deionized water. Pre‐oxidation of the nanofibers at 250°C for 6 h under air atmosphere	n.a.	700	n.a.(NH_3_)	n.a.	1056	n.a.	n.a.	[124]
10	Polyaniline (PA)	Pre heat treatment at 600°C for 1 h under Ar atmosphere	KOH	800	n.a.(Ar)	HCl and ultrapure H_2_O	n.a.	n.a.	n.a.	[67]
11	Melamine, Phytic acid and Aniline	n.a.	NaOH	800	5(n.a.)	Alcohol and distilled H_2_O	2735	1.5	0.3	[125]
12	Magnolol	Addition polymerization of magnolol‐based epoxy resin and cured in muffle furnace at a gradient temperature of 135–250°C for 2 h	KOH	800	5(n.a.)	HCl solution and deionized H_2_O	954–2286	n.a.	n.a.	[51]
13	Furfuryl alcohol	Calcination of commercial NaY zeolite at 530°C for 4 hc and vacuumed for 4 h at 150°C, then impregnated with furfuryl alcohol (FA) under vacuum condition.	NaY zeolite hard template	700–900	n.a.(N_2_)	HF and HCl solution	2791	0.8	0.68	[55]
14	Isobutyl‐triethoxysilane	Solution mixture of Isobutyltriethoxysilane and acetone/methanol dissolved in LiOH·H_2_O and H_2_O and filtering	Isobutyl‐triethoxysilane	900	10(Ar)	10 wt.% HF, deionized H_2_O and ethanol	1022	0.08	2.89	[126]
15	Pyrrole/Aniline	Freeze drying of solution mixture of aniline, pyrrole, ammonium persulfate, SiO_2_ microspheres and pre heating at 700°C under Ar atmosphere	NaOH	800	5(Ar)	Ultrapure H_2_O	n.a.	n.a.	n.a.	[127]
16	Dolomite	Carbonization of dolomite at 850°C under nitrogen atmosphere	K_2_CO_3_	900	n.a.(N_2_)	10 wt.% HF, 5 M HCl solution and deionized H_2_O	1530	0.64	2.53	[128]
17	Aniline/Sulfanilamide	Salt templating mixture of NaCl, Sulfanilamide and Aniline	n.a.	800	n.a.(Ar)	H_2_O	704	n.a.	n.a.	[129]
18	Melamine and PTFE	Mixture of melamine, PTFE and Maganous acetate template with ethanol. Heat treatment at 450°C for 2 h under Ar atmosphere	Maganous acetate	800	3(Ar)	n.a.	530	n.a.	n.a.	[130]
19	OPDN (4,4′‐(4‐oxophthalazine‐1,3(4H)‐diyl)dibenzonitrile)	Salt templating mixture of ZnCl_2_ and OPDN (4,4′‐(4‐oxophthalazine‐1,3(4H)‐diyl)dibenzonitrile)	KOH	600	5(Ar)	n.a.	687	n.a.	n.a.	[131]
20	Magnesium citrate	Mixture of magnesium citrate and ammonium persulfate	n.a.	650–850	n.a.	1 M HCl and deionized H_2_O	1169	1.6	0.5	[76]
21	Resorcinol	Solution mixture of tetraethyl orthosilicate, resorcinol and formaldehyde.	n.a.	700	n.a.(Ar)	HF solution	791	0.93	0.29	[70]
22	Graphite Oxide	Pre‐carbonization at 600°C under Ar atmosphere.	n.a.	600	5(NH_3_)	n.a.	295	n.a.	n.a.	[74]
23	Urea	Annealing of urea at 550°C under Ar flow	KOH	800	n.a.(Ar)	2 M HCl and deionized H_2_O	2846	n.a.	n.a.	[132]
24	Resorcinol	HTC treatment of resorcinol and formaldehyde at 120°C for 200 min	n.a.	800	n.a.(N_2_)	n.a.	733	n.a.	n.a.	[133]
25	Expanded graphite	Heat treatment of pristine expanded graphite and boric acid solution	n.a.	900	10(Ar)	n.a.	n.a.	n.a.	n.a.	[73]
26	Antimony acetate /Thioacetamide	Dissolution of antimony acetate and thioacetamide in ethylene glycol.	n.a.	900	3(Ar)	n.a.	877	n.a.	n.a.	[134]
27	Co(NO_3_)_2_.6H_2_O/2‐ methylimidazole	Dissolution of Co(NO_3_)_2_.6H_2_O in methanol and 2‐methylimidazole. Thermal treatment of 500°C for 5 h under Ar/H_2_ atmosphere	KOH	800	3(N_2_)	2 M HCl and deionized H_2_O	911	0.38	1.29	[135]
28	Tetrasodium ethylenediaminetetra‐acetate tetrahydrate	Thermal annealing of tetrasodium ethylenediaminetetra‐acetate tetrahydrate	KOH	800	10(Ar)	diluted HCl acid and distilled H_2_O	1478	n.a.	n.a.	[136]
29	Pyrrole	Polymerization of pyrrole with ammonium peroxydisulfate and deionized H_2_O	n.a.	1000	5(Ar)	0.1 M HCl and distilled H_2_O	n.a.	n.a.	n.a.	[137]
30	Furfuryl alcohol	Calcination of the product of the mixture of tetraethylorthosilicate, tri‐block copolymer P123, and conc. HCl, at 550°C for 2.5 h. The obtained template was impregnated with furfuryl alcohol and heated at 350°C for 2 h under N_2_ atmosphere	Tetraethylorthosilicate hard template	750	n.a.(N_2_)	2 M NaOH and deionized H_2_O	1090	n.a.	n.a.	[138]
31	3‐aminophenol	Pre carbonization of 3‐aminophenol/TPOS/formaldehyde solution at 800°C for 5 h under Ar atmosphere.	KOH	800	n.a.(Ar)	1 M HCl solution and deionized H_2_O	2126	0.51	0.47	[139]
32	Sodium citrate	Calcination of sodium citrate between 400 and 1000°C and washed with hot deionized water and ethyl alcohol	KOH	800	n.a.(Ar)	2.0 M HCl, washed with ethyl alcohol and deionized H_2_O	1094	n.a.	n.a.	[77]
33	Polyacrylonitrile (PAN)	Carbon fibers: Electrospinning of PAN solution containing DMF then annealing at 280°C for 90 min	n.a.	700	n.a.(Ar)	n.a.	n.a.	n.a.	n.a.	[69]
34	Polyacrylonitrile (PAN)	Carbon fibers: Electrospinning of PAN solution containing DMF then stabilisation at 280°C for 90 min	n.a.	700	n.a.(Ar)	n.a.	n.a.	n.a.	n.a.	[140]
35	Resorcinol	Solution mixture of tetraethylorthosilicate, TEOS, resorcinol and formaldehyde	n.a.	600	n.a.(N_2_)	2 M NaOH and deionized H_2_O	267		n.a.	[46]
36	Zeolitic imidazolate frameworks‐8 (ZIF‐8)	ZIF‐8 carbonization at 800°C under Ar atmosphere	KOH	800	5(Ar)	dilute HCl and deionized H_2_O	3738	n.a.	n.a.	[66]
37	d‐(+)‐Gluconic acid δ‐lactone (GA)	Freeze drying of GA, potassium chloride, followed by the addition of potassium carbonate in liquid nitrogen (‐196°C)	K_2_CO_3_	850	3(N_2_)	Hot distilled H_2_O	2325	0.88	0.51	[141]
38	NH_2_‐MIL‐125 (Ti)	Annealing of synthesized NH_2_‐MIL‐125 (Ti) under Ar and NH_3_ atmosphere. Autoclaving the product with other chemicals at 150°C for 6 h. Then centrifuged and washed with methanol and dried.	n.a.	800	5(Ar)	20% HF solution, washing with 0.01 M NaOH solution and deionized H_2_O	1731	n.a.	n.a.	[142]
39	Polypyrrole	Dissolution of potassium bicarbonate with PPy nanoparticles. Then evaporated by heating	KHCO_3_	800	4(n.a.)	Distilled H_2_O	2970	1.0	1.45	[48]
40	Graphene oxide (GO)	Sonication of solution containing amino‐functionalized silica spheres and graphene oxide and later centrifuged and dried	n.a.	700	n.a.(Ar)	10% HF etching and washing with deionized H_2_O	320	n.a.	n.a.	[143]
41	Glyoxal	Mixing of glyoxal and urea with deionized water and KOH until homogeneous slurry was obtained.	KOH	800	n.a.(Ar)	1 M HCl solution and deionized H_2_O	2961	0.043	0.071	[144]
42	Metal Organic Framework (MOF)	Dissolution of Zn(CH_3_COO)_2_.2H_2_O and 2‐methylimidazole and PVP with deionized (DI) water. The mixture was stirred for 48 h.	KOH	800	n.a.(N_2_)	HCl solution	3285	0.43	2.61	[78, 79]
43	Polyaniline	Polymerization of aniline, HCl, ammonium persulfate and distilled water. The obtained PANi was collected and filtered with water and freeze dried for 48 h. Final treatment at 600°C for 1 h under Ar atmosphere.	n.a.	800	n.a.(Ar)	Diluted HCl solution and ultrapure H_2_O	35	n.a.	n.a.	[68]
44	Polyimide	Dissolution of p‐phenylenediamine in DMF and PMDA and mixture treatment inTeflon‐lined autoclave and heated at 210°C for 10 h, precipitates were then centrifuged, washed with DMF and ethanol, and dried under vacuum at 80°C.	KOH	650	5(N_2_)	Diluted HCl solution and ultrapure H_2_O	1302	0.4	0.27	[145]
45	Graphene oxide (GO)	Heat treatment of graphene oxide in hydrogen peroxide solution at 100°C	n.a.	210	n.a.	n.a.	n.a.	n.a.	n.a.	[146]
46	Sodium alginate	Calcination at 400°C for 2 h in air atmosphere	NaOH	800	n.a.(Ar)	HCl solution, ethanol and distilled H_2_O	3229	n.a.	n.a.	[147]
47	Graphite	Autoclave treatment at 180°C for 24 h	n.a.	n.a.	n.a.	Distilled H_2_O and ethanol	19.7	n.a.	n.a.	[71]
48	Lignite	n.a.	KOH	900	5(N_2_)	HCl and distilled H_2_O	3084	1.08	0.70	[148]
49	Tannic acid	Salt templating with MgSO_4_, NaCl and KCl	MgSO_4_, NaCl and KCl	800	5(N_2_)	distilled H_2_O, diluted HCl and hot distilled H_2_O	1980	0.77	0.25	[52]
50	Maleic acid	Self‐templating of maleic acid with Cs_2_CO_3_	Cs_2_CO_3_	800	1(N_2_)	1 M HCl and deionized H_2_O	3066	0.88	0.38	[54]
51	Carbon foam	Functionalization of carbon foam with H_2_SO_4_/HNO_3_ at 80°C for 2 h	KOH treatment of carbon without acid treatment	700	n.a.(N_2_)	n.a.	1082	n.a.	n.a.	[149]
52	Nitrilotriacetic acid trisodium (NTA‐3Na)	Calcination of NTA‐3Na at 700°C for 1 h under Ar flow	KOH	800	n.a.(Ar)	Dilute HCl and deionized H_2_O	127	n.a.	n.a.	[150]
53	Phloroglucinol/Glyoxylic acid/Guanine	Polymerization of Phloroglucinol/Glyoxylic acid/Guanine and salt template, and heating at 120°C for 6 h to form resin polymer with salt template	CsCl/LiCl/NaCl/NaOH	900	2(Ar)	hot H_2_O at 80°C	2412	0.86	0.21	[23]
54	Disodium roseate salt	Thermal treatment at 700°C under Ar atmosphere	Salt templating	390–450	10(Air)	1 M HCl and deionized H_2_O	1003	1.36	1.01	[151]
55	Anthracite coal/Boric acid/sodium hypophosphite	Grinding and milling of coal and treated in HCl acid to remove impurities	KOH	900	n.a.(Ar)	2 M HCl and deionized H_2_O	1856	n.a.	n.a.	[88]

n.a., not applicable

The second family of precursors is the biopolymers, at approximately 17% (Figure 7c and Table 4). The availability and favorable attributes of biopolymers, including low cost, biodegradability, renewability, non‐toxicity, and desirable textural properties, have spurred researchers to explore their chemical structure, physicochemical characteristics, and potential uses in energy storage applications [81, 82, 83]. Biopolymers have been utilized in the development of an advanced porous carbon positive electrode for SIC. Plant‐based biopolymers such as lignin [84], cellulose [85, 86, 87, 88], and starch [89, 90] are the major precursors for porous carbon synthesis for SIC application. However, animal‐derived polymers such as gelatin, chitosan, and chitin are less explored. The major drawbacks of using biopolymer precursors for porous carbon synthesis stem from their inherent variability and processing challenges [91]. Biopolymers exhibit inconsistent chemical compositions depending on their source and processing history, leading to unpredictable pore structures and properties in the final carbon materials [92]. Although some biopolymers present high C‐yield (∼40–50% [93, 94] for lignin, chitosan, etc.), most of them suffer from low carbon yield (∼8% [90] for starch) due to high oxygen and hydrogen content, making the process economically inefficient compared to fossil‐based alternatives.

**TABLE 4:** Literature review of porous carbon materials derived from biopolymer precursors and their synthesis parameters.

S/N	Carbon source	Synthesis methods	Activating agent	Carbonization Temperature (°C)	Heating Rate,°C min^−1^ (gas)	Washing method	SSA (m^2^ g^−1^)	V_micro_ (cm^3^ g^−1^)	V_meso_ (cm^3^ g^−1^)	Refs.
1	Lignin / PAN	Electrospun of nanofiber films, heat treatment in air at 200–280°C	CO_2_	900	5 (Ar)	n.a.	1390	0.50	0.08	[84]
2	Potato starch residue	Drying and stabilization in air at 230°C	KOH	800	n.a.(N_2_)	HCl solution	954	n.a.	n.a.	[89]
3	Glucose	Heating the mixture of glucose and CaCO_3_ in ethanol/water	CaCO_3_	1200	(inert gas)	HCl solution	1099	n.a.	n.a.	[152]
4	Bacterial cellulose	Freeze‐drying to obtain aerogel, heat treatment at 1000°C under N_2_ atmosphere for 2h	KOH	800	5(n.a.)	n.a.	1963	0.52	0.95	[153]
5	Sucrose	Hard templating with ZnO. Solution mixture of ZnO, Sucrose and NaOH	NaOH	950	1(N_2_)	1 M HCl aqueous solution	1762	n.a.	n.a.	[154]
6	Starch	HTC synthesis of soluble starch in KHCO_3_ and HCl solution at 180°C for 8–20 h	KHCO_3_	900	5(N_2_)	2 M HCl as well as deionized H_2_O	1972	0.79	0.1	[90]
7	Sugar	Autoclave treatment of sugar solution at 190°C for 2h and drying.	KOH	800	n.a.	deionized H_2_O and ethanol	1830	n.a.	n.a.	[155]
8	Honey	Washing of honey with concentrated H_2_SO_4_ until it becomes char, preheated under air at 150°C for 24 h, then dried and grinded, further heat treatment at 350°C for 4 h	KOH	800	5(Ar)	Dilute HCl, H_2_O and ethanol	1554	n.a.	n.a.	[156]
9	Bacterial cellulose	Cutting, mechanical stirring, freeze drying, mixing with KOH solution, pre heating at 200°C under Ar atmosphere	KOH solution	800	5(Ar)	HCl solution and deionized H_2_O	1606	n.a.	n.a.	[85]
10	Fish scale	Heat treatment of fish scale under air at 350°C	KOH	800	2.5(Ar)	1 M HCl as well as deionized H_2_O	3285	0.8	2.02	[157]
11	Bacterial cellulose	Washing bacterial cellulose hydrogel with deionized water and tert‐butanol then, freeze‐drying at ‐45°C for 72 h	n.a.	800	2(n.a.)	n.a.	1764	n.a.	n.a.	[86]
12	Cotton cloth	Heat treatment under Ar at 400°C	KOH	800	5(Ar)	HCl and distilled H_2_O	1995	n.a.	n.a.	[158]
13	Carboxylmethyl chitosan	Heat treatment under Ar at 600°C	FeCl_3_·6H_2_O	900	5(Ar)	HCl solution	351	n.a.	n.a.	[159]
14	Methyl cellulose/β‐cyclodextrin	Mixture of methyl cellulose with β‐cyclodextrin and NaCl in deionized water and frozen. Then thermally treated at 400°C under N_2_ gas.	Salt templating with NaCl and activation KOH	700	5 (N_2_)	2 M HCl and distilled H_2_O	1561	0.94	0.62	[87]
15	Bacterial cellulose (BC)	Freeze drying of BC/PMMA (polymethyl methacrylate) composite. Activation with KOH at 80°C via hydrothermal treatment.	KOH	1050	n.a.	dilute HCl and DI H_2_O	248	0.02	0.27	[160]

n.a., not applicable

The last family of precursors is the biomass that accounts for approximately 29% (Figure 7d). Biomass is an eco‐friendly precursor, including renewable crops or animals, that reduces atmospheric pollution. The conversion of biomass to PC can be done through simple pyrolysis processing methods [95]. There have been comprehensive research reports focused on the production of carbon‐based electrode materials from abundant and cost‐effective renewable biomass sources such as plants, animals, and marine organisms [96, 97, 98]. According to a survey, annual global crop production is approximately 146 billion tons [99]. However, around 100 billion tons of domestic and animal husbandry waste are generated yearly, contributing to significant waste management and environmental challenges [100]. Thus, the use of biomass to produce porous carbon electrodes for capacitor technology offers an effective way to solve the problem of agricultural and domestic waste. Low‐cost activated carbons are frequently made from renewable biomass or agricultural waste such as peanut shells [101], coconut shells [102], corncob [103], onion husk [104], albizia flowers [105], and sugarcane bagasse [106].

Peanut shells account for about 15% of precursors used for porous carbon positive electrode synthesis for SIC application (Figure 7d). Peanuts are widely cultivated leguminous crops with about 46 million tons grown annually worldwide, which generates more than 10 million tons of waste [107]. Thus, researchers have utilized them for numerous applications, including energy storage. Other biomass precursors include coconut shell [108], bamboo [109], camellia [110], medicinal capsules [111], cinnamon sticks [112], citrus peel [73], and garlic [47] (Table 5). One demerit of biomass‐based precursors includes the presence of high impurity content (ashes), complex microstructure, and seasonal/geographical factors, making their adoption and utilization challenging.

**TABLE 5:** Literature review of porous carbon materials derived from biomass precursors and their synthesis parameters.

S/N	Carbon source	Synthesis methods	Activating agent	Carbonization Temperature (°C)	Heating Rate, °C min^−1^ (gas)	Washing method	SSA (m^2^ g^−1^)	V_micro_ (cm^3^ g^−1^)	V_meso_ (cm^3^ g^−1^)	Refs.
1	Jute	Washing of Jute fibers with H_2_SO_4_ and deionized water, then transferred for microwave assisted HTC at 200°C.	KOH	800	4 (Ar)	HCl and deionized H_2_O	1529	n.a.	n.a.	[161]
2	Medicinal capsule wastes	Freeze‐drying (lyophilization), pre‐carbonization at 350°C under air for 2 h	KOH	600	5 (Ar)	0.1 M HCl and deionized H_2_O	1079	0.40	0.06	[111]
3	Abandoned bamboo	Peeling, crushing, washing with deionized water, NH_3_ plasma treatment.	CO_2_	900	(Ar)	HCl and deionized H_2_O	1022	0.3	0.24	[109]
4	Soy protein powder	Direct pre‐carbonization under Ar at 700°C	KOH	800	(Ar)	HCl aqueous solution	2717	n.a.	n.a.	[162]
5	Garlic	Peeling	KOH	900	3 (N_2_)	2 M HCl and deionized H_2_O	1682	n.a.	n.a.	[47]
6	Silkworm excrement	Direct pre‐carbonization at 400°C	KOH	800	5 (N_2_)	1 M HCl and deionized H_2_O	1602	0.85	0.19	[163]
7	Metaplexis japonica fluff	HTC in acetic acid aqueous solution at 180°C and washing with deionized H_2_O	n.a.	600	5 (Ar)	n.a.	906	n.a.	n.a.	[164]
8	Sugarcane Bagasse	Aqueous mixing with KOH	KOH (aqueous)	800	(Ar)	HCl and H_2_O	1762	n.a.	n.a.	[165]
9	Enteromorpha	Washing with deionized H_2_O	KOH	900	3(N_2_)	HCl and H_2_O	1968	0.8	0.71	[166]
10	Jasmine rice	Washing with deionized water, drying, milling, heating in aqueous H_2_SO_4_	KOH	900	5(Ar)	0.1 M H_2_SO_4_ and deionized H_2_O	2377	n.a.	n.a.	[167]
11	Goat hair	Washing with hot H_2_O, isoproponal, acetone and then pre‐carbonized at 450°C under Ar for 6 h	KOH	800	5(Ar)	Dilute HCl, H_2_O and ethanol	2042	n.a.	n.a.	[36]
12	Fruits (i.e., apples, dragon fruits, oranges, and pears)	Juice extraction, centrifugation. Sugar cane juice (HTC at 180°C for 6 h	KOH	800	(Ar)	1 M HCl and Deionized H_2_O	1197	n.a.	n.a.	[168]
13	Olive pit	Crushing and pre‐carbonization under Ar atmosphere	KOH	700	5(Ar)	HCl and water	2225	n.a.	n.a.	[33]
13	Peanut shell	HTC of the outer shell with concentrated H_2_SO_4_ in MQ‐water at 180°C and washing.	KOH	850	5(Ar)	2 M HCl and MQ‐ H_2_O	2050	0.73	0.44	[169]
15	Cinnamon sticks	Crushing, washing with water and drying, grinding with KOH	KOH	650	(Ar)	0.1 M HCl and distilled H_2_O	1540	n.a.	n.a.	[112]
16	Peanut skin	Salt templating with FeCl_3_	FeCl_3_ and KOH	800	5(Ar)	HCl and distilled H_2_O	2070	0.49	1.43	[49]
17	Outer peanut shell	HTC of the outer shell with concentrated H_2_SO_4_ in MQ‐water at 180°C, washing, grinding with KOH	KOH	800	(Ar)	2 M HCl and distilled H_2_O	1900	n.a.	n.a.	[170]
18	Grinded cork	Grinding, direct pre‐carbonization at 600°C in N_2_ atmosphere	NaOH/KOH	700–800	3(N_2_)	15% HCl and distilled H_2_O	2750	1.09	0.15	[171]
19	Peanut shell	HTC of the outer shell with conc. sulfuric acid in MQ‐water at 180°C	KOH	800–850	(Ar)	2 M HCl at 60° and MQ‐H_2_O	2396	0.83	0.47	[172]
20	Corn stalk	Salt templating with aqueous K_2_C_2_O_4_.H_2_O. Grinding of dried salt templating and corn stalk mixture	K_2_C_2_O_4_.H_2_O	800	10 (N_2_)	n.a.	1802	0.69	0.25	[173]
21	Coconut shell	HTC at 200°C for 20 min, drying at 105°C for 12 h. Salt templating with ZnCl_2_.	ZnCl_2_ then/ CO_2_	800	10 (CO_2_ and N_2_)	Dilute HCl solution and ultrapure H_2_O	1795	n.a.	n.a.	[108]
22	Waste watermelon seeds	Washing with distilled water and ethanol, drying in oven at 120°C, grinding to powder and pre‐carbonization at 600°C	KOH (non‐aqueous)	800	(Ar)	0.1 M HCl, ethanol and distilled H_2_O	2383	n.a.	n.a.	[174]
23	Tremella	Pre carbonization of tremella cattle bone at 400°C for 3 h under Ar	n.a.	550	(Ar)	1M HCl and deionized H_2_O	2520	n.a.	n.a.	[175, 176]
24	Gelatin	Dissolution of gelatin in KOH and pre carbonized at 350°C for 3 h	Aqueous KOH	900	2.5 (Ar)	Deionized H_2_O	2600	n.a.	n.a.	[177]
25	Citrus peel	Washing with distilled water and crushing	KOH solution	800	5 (Ar)	Ethanol and distilled H_2_O	1167	n.a.	n.a.	[178]
26	Camellia shell	Salt templating with K_2_C_2_O_4_/CaC_2_O_4_	K_2_C_2_O_4_/CaC_2_O_4_	800	5 (Ar)	HCl and distilled H_2_O	2186	n.a.	n.a.	[110]
27	Cuttle bone	n.a.	NaOH/KOH	700	3(N_2_)	HCl and H_2_O	1489	0.27	n.a.	[179]
28	Corn husk/GO	Washing the corn husk with ethanol and deionized water. Pre‐calcination at 350°C for 2 h under Ar. Then, mixing with graphene oxide.	KOH	800	n.a.(Ar)	n.a.	2250	n.a.	n.a.	[180]
29	Olive pits	Pyrolyzes of dried olive pits at 700°C under N_2_ flow	KOH	700	n.a.(N_2_)	2M HCl and distilled H_2_O	2138	n.a.	n.a.	[181]

n.a, not applicable

In addition to the intrinsic nature of the precursor (biomass, biopolymer, or synthetic), the selection is strongly governed by economic and technical constraints (Table 6):

1. Cost: Biomass precursors are by far the cheapest (often close to zero for agricultural/forestry waste), followed by biopolymers, while synthetic precursors can be several times more expensive.
2. Carbon yield: Synthetic polymers and pitch generally give the highest fixed‐carbon yield (up to 70%), [113, 114], reducing the amount of raw material needed and downstream activation chemicals.
3. Synthesis conditions and scalability: Biomass requires minimal pre‐treatment but often harsh chemical activation to reach high porosity; biopolymers offer intermediate behavior, while synthetic routes allow the most precise control (e.g., hard/soft templating, stoichiometric doping) at the cost of complexity [115, 116].
4. Influence of precursor composition: The presence of natural minerals and heteroatoms in biomass leads to self‐templating/self‐activation effects, but also to ash‐related pore blocking. Biopolymers provide uniform and high heteroatom doping (especially nitrogen >5 wt%) [117], improving surface polarity and reactivity. Synthetic precursors enable carbon‐rich frameworks, incorporate dopants, maximize electrical conductivity, and enable highly ordered or ultra‐high‐surface‐area structures, [118] which is critical for energy storage applications.

**TABLE 6:** Comparison of the three major precursor categories (biomass, biopolymers, and synthetic precursors) for the preparation of porous carbons, focusing on cost, carbon yield, typical synthesis/activation conditions, compositional characteristics, and their resulting influence on the physicochemical properties of the final carbon materials.

Precursor Type	Typical Examples	Approximate Cost (USD/kg, 2023–2025)	Typical Carbon Yield (direct pyrolysis)	Ease of Synthesis / Typical Activation Conditions	Main Compositional Features	Influence on Physicochemical Properties of Porous Carbons	Advantages	Limitations
Biomass	Coconut shell, wood, rice husk, fruit peels, seaweed, waste biomass	0.05–1.5 (often <0.5 for wastes)	15–35%	Easy collection; pyrolysis 500–900°C; activation usually needed (KOH, H_3_PO_4_, steam)	High O, H; natural minerals (K, Ca, Si, Mg); heteroatoms (N, S, P possible)	Intrinsic heteroatom doping; natural templating (SiO_2_, CaCO_3_); high microporosity after activation; ash can act as natural activator or pore blocker	Extremely low cost, renewable, sustainable, inherent doping/templating	High ash content → pore blocking; variable composition batch‐to‐batch; lower graphitization degree
Biopolymers	Cellulose, chitosan, lignin, alginate, starch, proteins	2–50 (chitosan/lignin cheaper, pure cellulose higher)	10–40%	Moderate; pyrolysis 600–1000°C; often requires pre‐carbonization + KOH/ZnCl_2_ activation	Well‐defined O/N/S functional groups; controllable molecular weight	High and uniform heteroatom doping (especially N from chitosan/proteins); tunable oxygen functional groups → better wettability and pseudocapacitance	Reproducible composition, high heteroatom content, good solubility for templating	Higher cost than raw biomass; still contains some impurities
Synthetic precursors	Phenolic resin, polyacrylonitrile (PAN), pitch, furfuryl alcohol, PVDF, ionic liquids	5–200+ (pitch cheapest, PAN/ionic liquids expensive)	40–70% (pitch/PAN >60%)	Controllable; pyrolysis 600–1200°C; template (soft/hard) or direct activation possible	Rich in carbon; dopant can be present, controllable cross‐linking; minimal mineral impurities	Highest carbon purity → excellent electrical conductivity and graphitization; highly designable pore structure (ordered mesoporous carbons, CDC); very high surface area possible (>3000 m^2^/g)	Excellent reproducibility, tunable structure, high yield, high conductivity	High cost, fossil‐based (except some bio‐derived resins), complex synthesis, environmental concerns

### 3.2 Synthesis Methods

In this section, the major porous carbon synthesis methods are discussed.

#### 3.2.1 Polymerization

The use of polymerization reaction is increasingly utilized for porous carbon synthesis. This method is often employed in the synthesis of the so‐called resins. Phenolic resin based on resorcinol and formaldehyde, often called (RF) resin, is the most used resin in porous carbon synthesis. RF resin is a thermosetting polymer formed through the condensation reaction of resorcinol (a dihydric phenol) and formaldehyde. The resulting resin is a thermosetting polymer with a rigid, heat‐resistant structure due to the aromatic backbone and extensive cross‐linking. For instance, Wang et al. [133] used the HTC process to synthesize resorcinol‐formaldehyde (RF) resin. The obtained powder was mixed with ammonium chloride and calcinated at 800°C under Ar atmosphere to obtain nitrogen‐doped carbon with a nanospheres morphology having a SSA of 733 m^2^ g^−1^. The developed carbon electrode exhibited a reversible capacity of 90 mAh g^−1^ at 5 A g^−1^ in 1 M NaPF_6_ in EC:DEC electrolyte. Similarly, Wang and colleagues [46] developed N‐doped carbon hollow microspheres with a core‐shell structure using a sol‐gel method, employing SiO_2_ microspheres as templates, resorcinol/formaldehyde as the carbon source, and ethylenediamine as the nitrogen precursor. These uniform microspheres, ∼120 nm in diameter with ∼10 nm porous shells, exhibited a specific surface area (267 m^2^ g^−^^1^) and pore volume (1.2 cm^3^ g^−^^1^). Compared to non‐doped carbon (62 F g^−^^1^), the N‐doped microspheres showed superior specific capacitance (146 F g^−^^1^ at 10 mV s^−^^1^) in 1 M NaPF_6_ in EC:DMC electrolyte, attributed to enhanced electrical conductivity from 8.2 wt.% N‐doping and hierarchical porosity, which facilitated better electron transfer.

**FIGURE 8:**
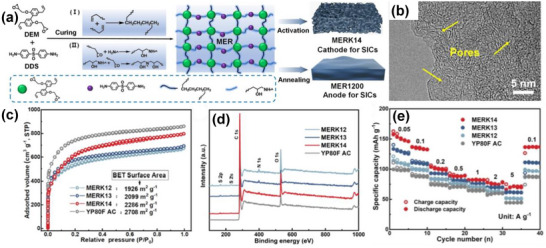
(a) Schematic representation of porous carbon synthesis involving additive polymerization synthesis of magnolol‐based epoxy resin for negative electrode and positive electrode material, (b) HRTEM image of MER based porous carbon, (c) N_2_ adsorption‐desorption isotherms and BET surface area of MER based porous carbons and YP80F commercial activated carbon, (d) XPS survey spectra, and (e) electrochemical performance of MER based porous carbon and AC porous positive electrodes vs. Na metal half‐cell in 1 M NaClO_4_ EC:DEC electrolyte. Reprinted with permission [51] Copyright (2022), Elsevier.

As shown in Figure 8, Liu et al. [51] reported the synthesis of magnolol‐based resins (MER) by the additive curing of Magnol's diglycidyl ether using 4, 4′‐diaminodiphenyl sulfones (DDS) between 135 and 250°C. The resin product was thermally treated with KOH at 800°C, under argon flow, at 5°C min^−1^ heating rate (Figure 8a). The developed porous carbon features a uniform pore size distribution with a Type I isotherm, dominated by micropores, achieving a SSA of up to 2286 m^2^ g^−1^, as shown in Figure 8b and c. The choice of the synthesis route and precursors allows for the introduction of heteroatoms such as O, N, and S, respectively (Figure 8d). The authors reported a high specific capacity of ∼136 and 71 mAh g^−1^ vs. Na metal at 0.1 and 5 A g^−1^, respectively, for the most optimized MER porous positive electrode, which is better compared to the YP‐80F commercial activated carbon (∼98 and 44 mAh g^−1^) used in the study as reference material (Figure 8e). The authors ascribed the enhanced performance to the well‐tuned pore size that matches the size of the fully solvated $ClO_{4}^{-}$ ions and the presence of well‐engineered N, O and S heteroatoms, which promotes efficient capacitive and pseudocapacitive charge storage.

Similarly, Adeniji et al. [23] also obtained nitrogen‐doped porous carbon (NDPC) using a combined soft‐salt template carbon synthesis approach. A resin based on phloroglucinol and glyoxylic acid (carbon source) and guanine (nitrogen source) was mixed with a specific type of salt (CsCl, NaCl, and/or LiCl) and Pluronic F127 [poly(ethylene oxide)‐block‐poly(propylene oxide)‐block‐poly(ethylene oxide, PEO_106_PPO_70_PEO_106_, Mw = 12,600 Da)] surfactant in a water/ethanol solution. The mixture was dried at room temperature, thermopolymerized at 120°C to form a guanine/phenolic resin polymer with a salt template, and pyrolyzed at 900°C under argon. The resulting doped porous carbon gave SSA between 1078 and 2412 m^2^ g^−1^, a maximum nitrogen content of about 8 at. % and, when tested vs. Na metal half‐cell under 1.5–4.5 V window, gave a discharge capacity of 83–159 mAh g^−1^ at 0.05 A g^−1^ in 1 M NaPF_6_ in EC:DMC electrolyte. The authors noted that the improved capacity is driven by the large specific surface area, substantial microporous volume with appropriate pore size, heteroatoms, and structural defects, which enhance ion adsorption. In addition, the capacity retention was promoted by the presence of mesopores and graphitic domains.

#### 3.2.2 Electrospinning

**FIGURE 9:**
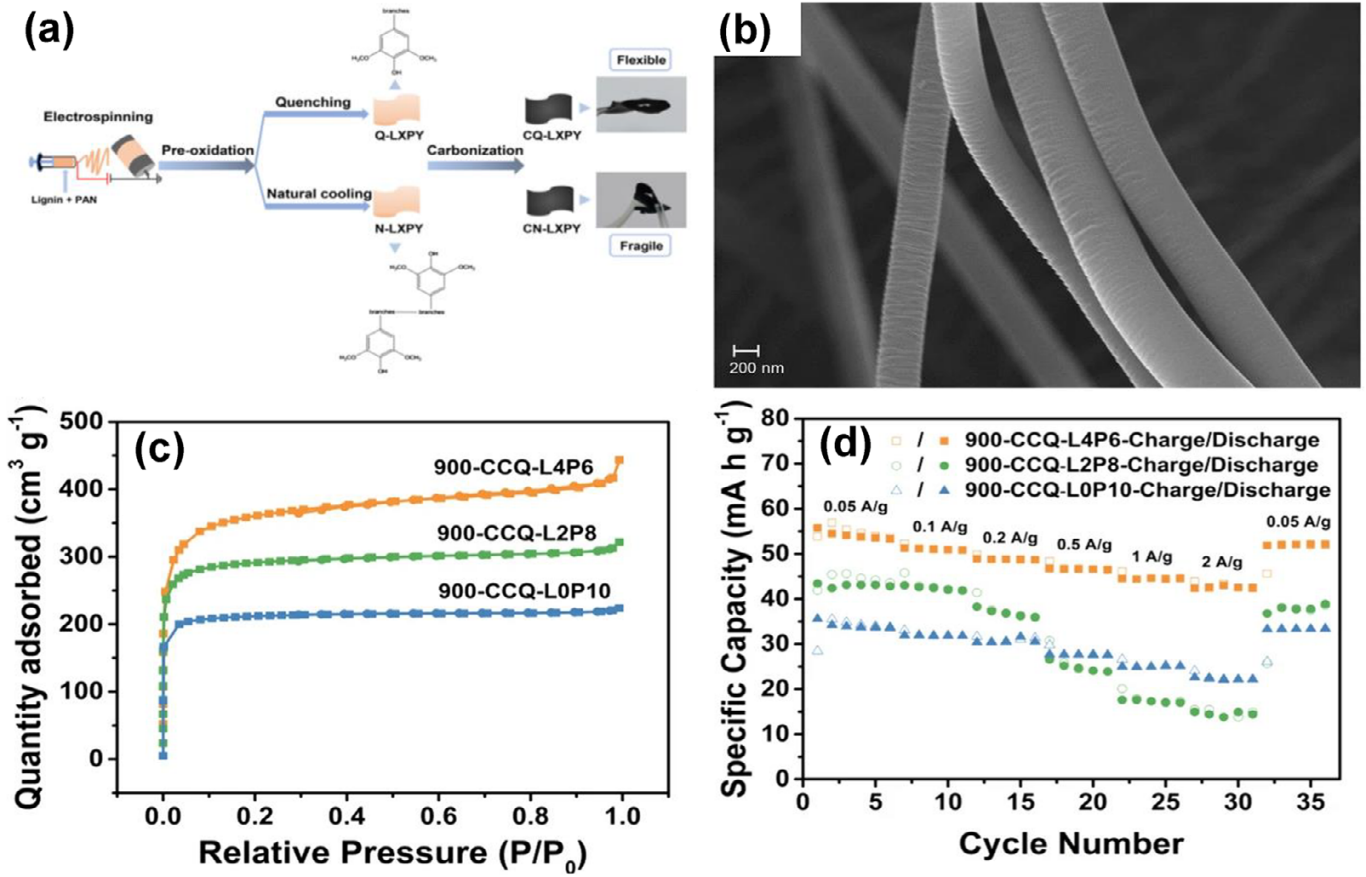
(a) Schematic representation of porous carbon synthesis via electrospinning of lignin and PAN, (b) SEM image of electrospun carbon nanofibers, (c) N_2_ adsorption‐desorption isotherms and (d) Electrochemical performance of 900‐CCQ‐LXPY porous carbons vs. Na metal half‐cell in 1 M NaClO_4_ in EC:PC (1:1) with 5% FEC electrolyte (where “L” represents Lignin, “P” represents PAN, and “X/Y” represents the proportion of lignin and PAN). Reprinted with permission [84] Copyright (2022), SNCSC.

The electrospinning process is a technique that utilizes the force produced from a voltage source to yield electrospun polymer fibers with nanometric diameters. It typically consists of a high voltage source (15–20 kV), a needle, a syringe pump, and a collector plate. The syringe pump exerts a force to push the polymer solution from the syringe through the needle. Typical polymer solutions used in the electrospinning process include Polyacrylonitrile (PAN) [69, 84, 140] or Polyvinyl Alcohol (PVA) [124] dissolved in N,N‐dimethylformamide (DMF) with carbon precursors mainly lignin [84] and phenolic resins [69, 140]. The utilization of PAN polymer precursors results in nitrogen‐doped carbon. PAN polymer typically requires a pre‐carbonization step, usually between ∼200 and 300°C, before the major thermal treatment step to improve its cyclization and prevent melting during the pyrolysis step. The advantage of the electrospinning technique is that a binder‐free electrode can be obtained directly, which is advantageous to reduce the inactive electrode mass, increase conductivity, and simplify electrode steps. For example, Zhao et al. [84] synthesized self‐standing carbon nanofibers by combining lignin and PAN in DMF, pre‐oxidized between 200 and 280°C, and activated with CO_2_ gas between 700 and 900°C (Figure 9a,b). The carbon nanofiber materials activated at 900°C showed a Type I isotherm with SSA of 1390 m^2^ g^−1^ (Figure 9c), showing a maximum specific capacity of ∼55 and 43 mAh g^−1^ at 0.05 and 2 A g^−1^, respectively, in 1 M NaClO_4_ in EC:PC (1:1) with 5% FEC electrolyte. The authors attributed the electrochemical performance to its high specific surface area and pore size, consisting of both micropores and mesopores that facilitate ion adsorption and capacity retention, respectively (Figure 9d). In another work [69] PAN was utilized to obtain carbon fibers (CF) flexible electrodes. PAN was dissolved in DMF, stirred overnight to create a viscous solution, and electrospun at 10–13 kV to form white PAN fibers on aluminum foil. The fibers were annealed at 280°C, then pyrolyzed at 700°C in an Ar atmosphere, yielding flexible carbon fiber electrodes. These exhibited a discharge capacity of 65 mAh g^−1^ at 0.5 A g^−1^, with the CF electrode demonstrating cycling stability over 500 cycles, retaining nearly 100% of its initial capacity in a 1 M NaClO_4_ (EC:DEC) electrolyte. Similar results were obtained by Liu et al. [140].

Furthermore, Yuan et al. [124] produced activated N/F co‐doped carbon nanofiber cages from the electrospinning of PVA in the presence of poly(tetrafluoroethylene) (PTFE) binder and ammonium metavanadate (NH_4_VO_3_). The electrospun fibers were preoxidized at 250°C under air for stabilization and pyrolyzed at 700°C under NH_3_ gas to introduce nitrogen species. The developed activated N/F co‐doped carbon nanofiber cages exhibited a hierarchical porous structure with a SSA of 1506 m^2^ g^−1^. The material achieved a specific capacity of ∼66 mAh g^−1^ when tested at a current density of 0.1 A g^−1^. This performance represents a 65% improvement over commercial activated carbon, which only delivered about 40 mAh g^−1^ under the same conditions. The authors noted that the enhanced performance of the co‐doped nanofiber is due to the highly developed SSA, abundant open nanopores, and the presence of N and F heteroatoms.

#### 3.2.3 Hydrothermal carbonization (HTC)

**FIGURE 10:**
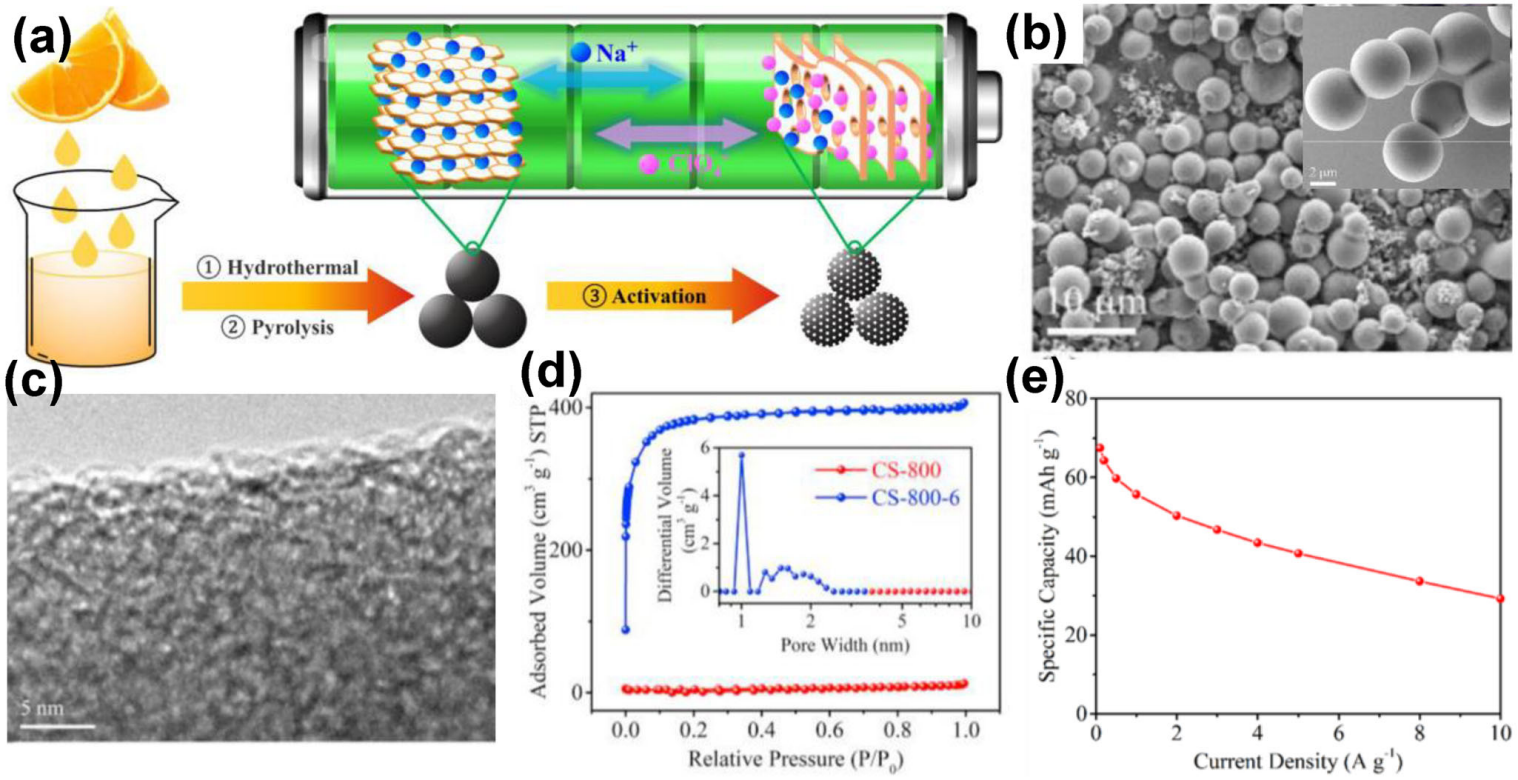
(a) Hydrothermal carbonization followed by activation synthesis of porous carbon from orange juice; (b) SEM images of as‐synthesized carbon spheres (CSs) at 10 µm, inset: SEM image orange juice CSs at 2 µm (c) TEM image of CS‐800‐6 porous positive electrode, (d) N_2_ adsorption‐desorption isotherms of CS‐800‐6 porous positive electrode and CS‐800 negative electrode; inset: pore size distribution, and (e) Electrochemical performance of CS‐800‐6 porous carbon positive electrode vs. Na metal half‐cell in 1 M NaClO_4_ in EC:DEC(1:1) and 5% FEC electrolyte. Reprinted with permission [168] Copyright (2018), Elsevier.

Hydrothermal pre‐treatment or HTC is one of the most adopted methods to control carbon morphology and prevent volume expansion in relation to the precursor during thermal treatment. It involves low temperature treatment (typically 180–230°C) under autogenerated pressure due to the water vapor and the decomposition gases of precursors. Biopolymers, such as starch [90], glucose [182], sucrose [155], and other sources [168], are frequently used as precursors in this regard thanks to their good solubility and adaptability, which enable the creation of particles with different sizes and varied interconnectivity. The control of particle size and shape presents particular importance to obtain homogeneous electrode coating, optimized porosity, and enhanced diffusion of ions within the pores [183, 184]. For example, orange juice [168] was loaded into a stainless autoclave and hydrothermally treated at 180°C for 6 h, followed by thermal treatment at 800°C under N_2_ gas to obtain spherical microspheres (3.3–4.7 µm), as seen in Figure 10a. Following activation with KOH, the spherical particles were preserved but with rougher surfaces (Figure 10b and c). The activated carbon microspheres exhibited a high SSA (1197 m^2^ g^−1^ vs. 4.7 m^2^ g^−1^ for non‐activated), with a Type‐I isotherm, primarily composed of micropores, as shown in Figure 10d. The activated sample showed a discharge specific capacity of 67.5 mAh g^−1^ at 0.1 A g^−1^ with 60.3% capacity retention at 5 A g^−1^, in 1 M NaClO_4_ in EC:DEC(1:1) and 5% FEC electrolyte, Figure 10e.

The residence time (HTC time) is a critical parameter that affects the particle size of the obtained hydrochar in the HTC process. It is the duration for which the precursor feedstock is maintained at the target temperature during the HTC process. It directly affects the extent of precursor decomposition, carbonization, and particle formation [185]. Short residence time may lead to incomplete carbonization, often resulting in larger, less uniform hydrochar particles with lower carbon content and higher volatile matter. While long residence time may promote further breakdown and restructuring of precursors, typically producing smaller, more uniform, and denser hydrochar particles with higher carbon content [186]. For example, Zhang et al. [90] studied the effect of HTC residence time on starch‐derived precursors for synthesizing KHCO_3_‐activated micro‐/mesoporous carbon microspheres (MCMS‐K). Starch was processed in a hydrothermal autoclave at 180°C for 8, 12, 16, and 20 hours, producing carbon microspheres (CMS) with average diameters of 0.49 ± 0.07, 1.09 ± 0.18, 1.29 ± 0.17, and 1.35 ± 0.25 µm, respectively. The authors noted that the longer HTC times increased microsphere size due to polycondensation and Ostwald ripening. CMS‐16, with the narrowest size distribution, was carbonized at 500°C and activated with KHCO_3_ at 900°C under nitrogen, yielding activated carbon microspheres (0.63 ± 0.07 µm) with a high specific surface area (1972 m^2^ g^−1^) and graphitic domains. In the same vein, sugar [155] was hydrothermally treated at 190°C, and the obtained carbon spheres were combined with KOH and pyrolyzed at 800°C. Following thermal treatment, the nanospheres exhibit an irregularly layered structure with SSA of 1830 m^2^ g^−1^, with numerous micropores achieving 141 and 70 F g^−1^ at 1 and 20 A g^−1^, respectively, in 1 M NaClO_4_ (EC:DMC) electrolyte. As reported by Platek et al. [187], the pyrolysis temperature plays an important role in the preservation of spherical particle shape. Low pyrolysis (400–550°C) leads to loss of the shape during the activation step, while higher temperature (700°C) perfectly preserves the shape due to the structurally well‐formed carbon matrix, with less functional O‐groups.

Moreover, porous carbon based on cellulose and complex biomass that are insoluble in water, including Metaplexis japonica fluff [164], peanut shell [49, 169, 170, 171, 172], and coconut shell [108] have also been explored via hydrothermal treatment. For example, Zhao et al. [164] prepared activated carbon via hydrothermal carbonization of Metaplexis japonica fluff biomass in an autoclave at 180°C and carbonized at 600°C under Ar flow, achieving 906 m^2^ g^−1^ SSA. This resulted in a hollow tubular structure and pore size distribution at 2 nm, delivering a specific capacity of 116 mAh g^−1^ at 1 A g^−1^, in 1 M NaClO_4_ (EC:DMC) with 5% FEC electrolyte. Crushed coconut shells [108] were processed in an autoclave at 200°C, activated with ZnCl_2_ solution, and thermally treated at 800°C under N_2_ flow. The resulting material displayed a granular particulate structure with a specific surface area of 1795 m^2^ g^−^^1^, achieving a specific capacitance of 120 F g^−1^ at 0.1 A g^−1^ and retaining 100% capacity after 70 cycles in 1 M NaClO_4_ in EC:DMC electrolyte.

In addition, synthetic precursors such as resorcinol/formaldehyde [133], polyimide [145], and graphite [71] have also been treated via HTC towards the synthesis of carbons for SIC. For example, resorcinol/formaldehyde [133] was mixed in ethyl alcohol, ammonia, and water while the resulting solution was hydrothermally treated in an autoclave at 120°C and then pyrolyzed at 800°C, under N_2_ flow, to produce nitrogen‐derived carbon serving as a positive electrode for SIC. The material is composed of nanospheres with a mean diameter of 300 nm, giving a 733 m^2^ g^−1^ SSA and a discharged capacity of 90 mAh g^−1^ at 0.5 A g^−1^. In addition, Yu and co‐workers [71] utilized HTC to obtain boron‐doped graphite (BG) sheets via HTC treatment of graphite nanosheets and ammonia boric acid in an autoclave at 120°C for 24 h, showing a sheet thickness of 120–150 nm with a low SSA (19.7 m^2^ g^−1^) and graphitic domains. The obtained boron‐doped graphite nanosheet achieved a specific capacitance of 155 and 96 F g^−1^ at 0.5 and 3 A g^−1^, respectively, in 1 M NaPF_6_ in EC:DMC electrolyte. The authors reported that the BG material, despite its low specific surface area (< 20 m^2^ g^−1^), achieves high specific capacitance not through electrical‐double‐layer mechanisms but via pseudocapacitive reactions, which drive its capacitive performance.

Table 7 below summarizes the effect of the major synthesis approaches for porous carbons on their surface chemistry, highlighting their key advantages and common limitations related to porosity development. The three synthesis routes differ significantly in their ability to control surface chemistry. Polymerization and electrospinning offer the strongest control because heteroatom‐doped polymers (containing N, S, P, or B) can be used directly as precursors, allowing precise design of surface chemistry before carbonization. These methods enable intentional doping through monomer selection or polymer blending, with functional groups and dopants uniformly distributed throughout the material. In contrast, HTC provides limited direct control over surface chemistry, as it relies primarily on the inherent composition of biomass feedstocks. To achieve heteroatom doping in HTC, supplementary dopant‐containing molecules must be added to the hydrothermal solution, making the process less direct and precise. This fundamental difference makes polymerization and electrospinning preferred routes when specific surface functionalities are critical for the target application.

**TABLE 7:** Summary of the effect of various porous carbon synthesis approaches on their surface chemistry.

S/N	Methods	Summary
1	Polymerization	Use of synthetic precursors Strong control over surface chemistry Heteroatom‐doped polymers (N, S, P, B) can be directly used as precursors Functional groups introduced during polymerization are retained after carbonization Precise control of dopant content and distribution through monomer selection Co‐polymerization enables multi‐heteroatom doping Surface chemistry can be designed before carbonization Activation is usually required as the carbonization of polymeric precursors typically produces materials with limited porosity. Activation (chemical with KOH, ZnCl_2_ or physical with CO_2_, steam) is almost always needed to develop high surface area and desired pore structure ✓ Without activation, surface areas are often <500 m^2^ g^−1^ ✓ With activation, SSA can achieve >2000 m^2^ g^−1^
2	HTC	Uses renewable carbohydrates, biomass and waste materials as feedstock Limited direct control over surface chemistry Surface chemistry primarily determined by biomass composition Supplementary molecules or dopant precursors must be added to the hydrothermal solution to induce heteroatom doping Co‐hydrothermal treatment with N‐, S‐, or P‐containing compounds enables doping Less precise control compared to polymerization approaches Functional groups depend on feedstock and process conditions HTC typically produces hydrochars with low surface area (<50 m^2^ g^−1^) and poorly developed porosity. The material is often dense with closed pores. Activation is generally necessary to achieving high surface area.
3	Electrospinning	Creates continuous nanofibers with high aspect ratios Strong control over surface chemistry Heteroatom‐doped polymers can be used directly in the spinning solution Multiple precursors can be blended in the solution for co‐doping Functional additives easily incorporated during spinning Uniform distribution of heteroatoms throughout the fibers Surface chemistry tailored through polymer and additive selection Similar flexibility to polymerization methods Electrospun carbon nanofibers inherently have external surface area due to their fibrous morphology. The interconnected fiber network provides some porosity without activation However, activation is still commonly used to increase surface area and create micropores within the fibers.

In terms of porosity development, polymerization offers excellent control over pore structure and surface chemistry, and control over the pore structure and hierarchy using synthetic precursors, but carbonized polymers typically yield low surface area (<500 m^2^ g^−1^) without subsequent activation. With chemical (KOH, ZnCl_2_) or physical (CO_2_, steam) activation, surface areas exceeding 2000 m^2^ g^−1^ are routinely achieved [188, 189]. HTC, a green, low‐temperature (180–250°C), water‐based route that directly processes renewable or waste biomass (even wet feedstock) into carbon‐rich hydrochars or spheres. It is cost‐effective and scalable, yet the resulting hydrochars are usually dense with very low surface area (<50 m^2^ g^−1^) and require post‐activation to open porosity and reach high surface areas [190]. On the other hand, electrospinning uses limited precursors and requires a stabilization step in producing continuous carbon nanofibers that naturally form interconnected 3D networks with high aspect ratios and external surface area. The fibrous morphology provides inherent meso/macroporosity; however, micropore development and further surface area enhancement are commonly achieved through additional activation [191, 192]. The electrospinning process can be used for binder‐free electrode production. In all three methods, activation (chemical or physical) remains a critical step to unlock ultra‐high surface area and well‐developed microporosity required for efficient ion adsorption and charge storage.

**FIGURE 11:**
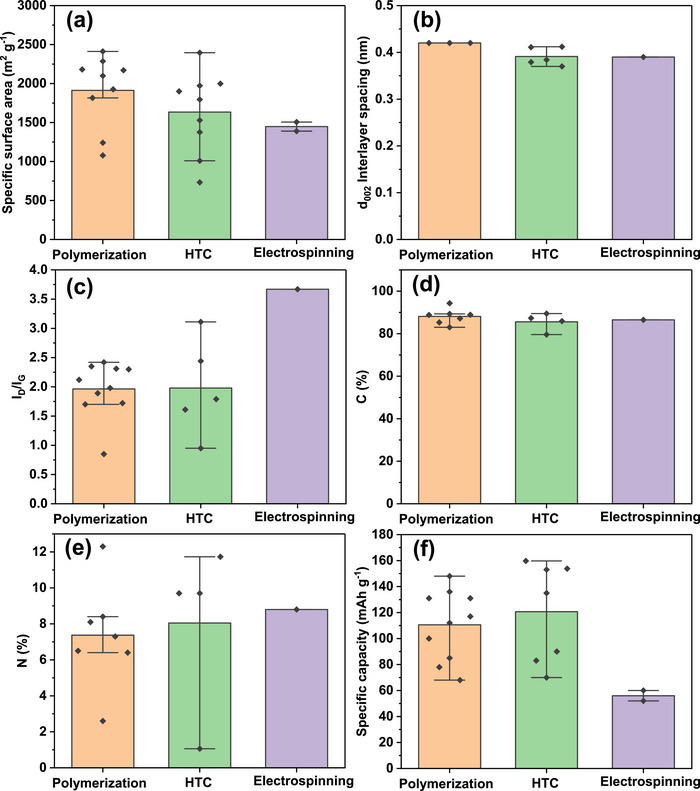
Porous carbon synthesis methods as a function of the (a) SSA, (b) d_002_ interlayer spacing, (c) Raman disordere degree, *I*_D_/*I*_G_, (d) C %, (e) N %, and (f) specific capacity at 0.1 A g^−^^1^ vs. Na/Na^+^ half cells in NaClO_4_ or NaPF_6_ electrolytes. The points on the graphs are different materials precursors utilizing either polymerization, HTC, or electrospinning process in the synthesis of porous carbons. The graphs were built based on the references in Tables 11, 12, 13.

A critical evaluation of the three synthesis methods reveals clear structure–property–performance relationships for porous carbons used as positive electrodes in SIC. It is important to note that all three synthesis methods proceed to the activation process. Figure 11 summarizes the typical ranges and distributions of six key material characteristics derived from a large dataset (Tables 3, 4, 5 and 11, 12, 13).

Regarding the textural properties from SSA (Figure 11a) point of view, polymerization yields the highest average values around 1900 m^2^ g^−1^, though with considerable variability among samples, ranging from approximately 1000 to 2400 m^2^ g^−1^. HTC shows an even broader range of surface areas, from approximately 700 to 2400 m^2^ g^−1^, with a mean around 1600 m^2^ g^−1^, suggesting that processing conditions significantly influence the final material structure. Electrospinning reveals surface areas tightly clustered around 1400–1500 m^2^ g^−1^, which are moderate SSA due to their fibrous morphology; note that the electrospinning dataset is the smallest of the three.

In terms of the structural properties, the graphite interlayer spacing analysis (Figure 11b) shows the similarity across all three methods. Polymerization, HTC, and electrospinning all produce materials with interlayer spacings around 0.38‐0.42 nm, which is higher than the theoretical graphite interlayer spacing of 0.335 nm. This indicates that all three methods generate carbon materials with disordered structures, and the synthesis route does not significantly impact the fundamental layer‐to‐layer distance in the carbon lattice. The *I*_D_/*I*_G_ ratio (Figure 11c), which reflects the degree of disorder and defect density in the carbon structure, shows substantial differences between methods. Both polymerization and HTC produce materials with *I*_D_/*I*_G_ ratios around 1.7–2.0, indicating a moderate level of structural disorder with a balance between crystalline graphitic domains and defects. Electrospinning, however, yields materials with a notably higher *I*_D_/*I*_G_ ratio approaching 3.6, suggesting significantly more structural defects and smaller graphitic domains. Although these values are a bit tricky due to the difference in evaluating the *I*_D_/*I*_G_ ratio (from 2, 4 and 5 Raman peaks deconvolution methods) [193, 194]. Thus, a uniformized approach is suggested for evaluating the disorder domain in porous carbon materials.

For surface chemistry, Carbon content (Figure 11d) remains relatively high and consistent across all methods, ranging from approximately 84%–88%. All three synthesis approaches produce materials with comparable carbon purity, with polymerization showing a slightly wider distribution, hinting at the possibility of tuning the carbon surface properties. Nitrogen content (Figure 11e) shows considerable variation both within and between methods. Polymerization produces materials with nitrogen levels ranging from approximately 2.5% to 12.5%, with an average of around 7%. HTC exhibits the widest range, from less than 1% to nearly 12% nitrogen content, averaging around 8%. Electrospinning yields more consistent nitrogen incorporation at approximately 8–9%. The presence of nitrogen heteroatoms can enhance electrochemical performance by providing additional active sites, improving surface wettability, and potentially increasing electrical conductivity through electron donation to the carbon framework.

The most critical parameter, specific capacity (Figure 11f), reveals a trend. The electrospun material exhibits dramatically lower specific capacity at approximately 55 mAh g^−^^1^, despite possessing the highest *I*_D_/*I*_G_ ratio. While elevated *I*_D_/*I*_G_ ratios typically indicate more defect sites available for ion storage, excessively high values can also reduce electrical conductivity [23, 195]. Additionally, the textural property analysis reveals that electrospun materials yielded the lowest surface area values, which likely contributes to their diminished specific capacity performance.

Both polymerization and HTC methods generate materials with substantially higher capacities, averaging around 110–120 mAh g^−1^, with some samples reaching 130–160 mAh g^−1^. The wide distribution in polymerization samples (ranging from about 65 to 145 mAh g^−1^) suggests that optimization of synthesis parameters is crucial for achieving high performance.

### 3.3 Strategies for Electrochemical Performance Enhancement

The electrochemical performance of porous carbons is governed by a complex interplay of structural and chemical factors that collectively determine their effectiveness in electrochemical energy storage. While the inherent properties of porous carbons provide a promising foundation, their practical performance often requires deliberate optimization to meet the demanding requirements of modern electrochemical devices. This section examines three primary strategies for enhancing the electrochemical performance of porous carbons: porosity enhancement, heteroatom doping, and impurity removal. Porosity enhancement focuses on developing hierarchical pore structures and optimizing pore size distributions to facilitate efficient ion transport and maximize electrochemically accessible surface area. Heteroatom doping introduces specific elements such as nitrogen, oxygen, sulfur, or phosphorus into the carbon framework to modify surface chemistry, improve wettability, and introduce additional pseudocapacitive active sites. Finally, impurity removal addresses the critical need to eliminate metallic and mineral contaminants that can compromise electrochemical stability and performance. Together, these strategies provide a comprehensive toolkit for tailoring porous carbon properties to specific electrochemical applications, bridging the gap between fundamental material properties and practical device requirements.

#### 3.3.1 Porosity Enhancement

Porosity enhancement is fundamental to maximizing the electrochemical performance of porous carbons, as pore structure directly governs ion accessibility, charge storage capacity, and mass transport kinetics. Two principal approaches have emerged for developing optimized pore architectures: pyrolysis and activation processes, which create porosity through controlled thermal treatment and selective gasification or chemical etching, and templating methods, which employ sacrificial templates to direct pore formation with precise control over size, distribution, and hierarchy. These complementary strategies enable the systematic engineering of pore networks tailored for enhanced capacitive charge storage.

##### 3.3.1.1 Pyrolysis and Activation

The pyrolysis step is an important stage in the synthesis of porous carbon. It involves the thermal treatment of pristine or pre‐carbonized precursors at a very high temperature, mostly between 500 and 1000°C, under an inert gas (N_2_ or Ar) atmosphere. This process typically takes place in a high‐temperature muffled furnace, which helps to drive the decomposition of biomass or organic matter, while removing volatile components to form a carbonaceous framework. It is worth noting that the inert atmosphere prevents carbon oxidation, ensuring the carbon structure's integrity. The final porosity structure and pore size distribution are highly influenced by the selected pyrolysis temperature, heating rate, and precursor composition.

**FIGURE 12:**
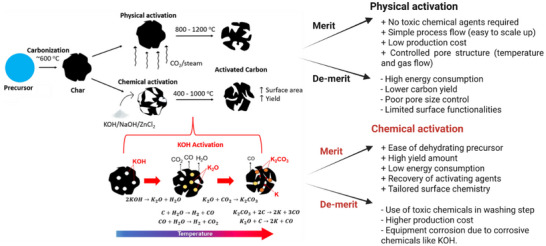
Schematic representation of physical and chemical activation approach for porous carbon synthesis. It also highlights both the merits and demerits of physical and chemical activation methods. Reproduced from [196], the Royal Society of Chemistry.

Activation is one of the most important steps in porous carbon synthesis. It is a critical stage that intends to increase/introduce porosity to the carbon materials. It is mostly divided into two groups: physical and chemical activation (Figure 12). The physical activation process involves the treatment of carbon precursor/biochar with CO_2_, air, or steam (water vapor) atmosphere. The chemical activation process involves the introduction of chemical agents such as alkali (KOH and NaOH), and salts (ZnCl_2_, FeCl_3_, K_2_C_2_O_4_·H_2_O, and K_2_C_2_O_4_/CaC_2_O_4_), which help to create micropores and/or mesopores during the carbonization process. These agents improve the specific surface area and allow control over the textural properties and pore size distribution. In addition, the different chemical agents can also be mixed to achieve improved porous properties.

###### 3.3.1.1.1 Physical Activation

In the physical activation methods, oxygen‐containing molecules such as water vapour, CO_2_, or air are used as activating agents that react with carbon materials at a high temperature (800–1200°C) to remove the carbon atoms, resulting in a high degree of burn‐off which develops its porous structure. The physical activation methods allow for fine control of the pore by adjusting the temperature and the gas flow. Increasing the carbon activation temperature and flow rate progressively enhances the number of pores and the SSA. However, this improvement comes with a trade‐off: as activation becomes more aggressive, the pores can become too large or even collapse, leading to a reduction in desirable textural properties. The activating agent significantly influences the pore structure in physical activation, particularly for biomass‐based precursors. CO_2_ and O_2_, due to their higher reactivity [197], produce smaller pores compared to H_2_O vapor. However, precise control of the carbonization process is essential to prevent over‐oxidation of the carbon surfaces, ensuring optimal pore development. For instance, Cai et al. [109] synthesized porous carbon from ground bamboo biomass powder, which was calcinated under CO_2_/Ar gas flow for 2 h at 900°C and was further treated with NH_3_ plasma for nitrogen doping. The developed material exhibits a maximum SSA of 1022 m^2^ g^−1^ with a discharge capacity of 101 mAh g^−1^ at 0.1 A g^−1^ in NaClO_4_‐based electrolyte. Similarly, Jayaraman et al. [108] prepared an activated carbon electrode from coconut shell via a combination of salt template ZnCl_2_ and CO_2_/N_2_ activating agents, thermally treated at 800°C for 2 h. The obtained porous carbon material showed a BET SSA of 1795 m^2^ g^−1^ and a pore volume of 2.2 cm^3^ g^−1^. The activated carbon positive electrode delivered a capacity of ∼83 mAh g^−1^ at 0.1 A g^−1^ in 1 M NaClO_4_ in EC:DMC electrolyte. In a different study, Zhao and co‐workers [84] constructed flexible lignin‐based carbon materials from the electrospinning of Kraft lignin and PAN. The obtained electrospun nanofibers were pre‐oxidized at 280°C, carbonized at 900°C, and activated with CO_2_ for 0.5 h at 900°C. The obtained porous carbon exhibits a SSA of 1390 m^2^ g^−1^ and a discharge capacity of 55 mAh g^−1^ at 0.05 A g^−1^ in 1 M NaClO_4_ in EC:PC (1:1) with 5% FEC electrolyte.

The physical activation process offers several advantages, including the absence of toxic chemical agents, a simple process flow that is easy to scale up, low production costs, and the ability to control pore structure through temperature and gas flow adjustments (Figure 12). However, it has drawbacks, such as high energy consumption, significant carbon loss, limited precision in pore size control, and limited surface functionalities.

###### 3.3.1.1.2 Chemical activation

Chemical activation is a widely used method involving the use of alkali such as KOH, NaOH, K_3_CO_3_, or KHCO_3_ as the chemical activating agent (Tables 3, 4, 5). This approach typically treats carbon precursors such as biomass, biopolymers, or synthetic precursors with alkali under a high carbonization temperature in an inert gas atmosphere. The process helps to introduce micropores and mesopores, which offer enhanced specific surface area often exceeding 1000 m^2^ g^−1^. Chemical activation favors the ability to engineer a tailored porous structure with high yield compared to the physical activation method approach. For instance, Halder et al. [111] developed a porous carbon from medicinal capsule wastes pre‐treated by different chemical and thermal treatments. The produced char was mixed with KOH in a 1:3 ratio, then thermally treated at 600°C for 3 h under Ar atmosphere. In the thermal treatment stage, the activation reactions below led to the formation of the well‐developed porous structure.

(3) $$6\mathrm{KOH}+2C\to 2K+3H_{2}+2K_{2}CO_{3}$$

(4) $$K_{2}CO_{3}+C\to 3K_{2}O+2CO_{2}$$

The obtained porous carbon was washed with 0.1 M HCl and deionized water to remove the alkali and other impurities. The MAC porous carbon exhibits SSA of 1079 m^2^ g^−1^ with an average pore volume of 0.405 cm^3^ g^−1^. Similarly, Guo et al. [179] constructed a porous carbon involving pre‐carbonization of cuttlebones at 600°C for 1 h under N_2_ gas flow and freeze‐dried to obtain cuttlebones‐derived carbon sheets (CBCS). The CBCS was then mixed with NaOH and KOH (1:1:2 mass ratio), pyrolyzed at 700°C for 1 h under N_2_ flow, and then washed with 1 M HCl and deionized water to obtain a pH 7. The developed porous carbon showed a remarkable BET specific surface area of 3229 m^2^ g^−1^. Also, Zou et al. [136] obtained 3D nitrogen‐doped hierarchical activated carbon (NHPAC) from tetrasodium ethylenediaminetetraacetate tetrahydrate (Na_4_EDTA‐4H_2_O) following a pre‐carbonization at 800°C for 2 h under argon flow. The obtained carbon was further mixed with KOH at a 1:4 mass ratio, pyrolyzed at 800°C for 1 h, under an argon flow, and then washed with diluted HCl and distilled water several times. The obtained activated carbon displayed BET SSA of 1478 m^2^ g^−1^ and a total pore volume of 1.08 cm^3^ g^−1^. Chemical activation provides multiple benefits, including the ease of dehydrating the precursor, high yield amounts, low energy consumption, recovery of activating agents, and the ability to tailor surface chemistry (Figure 12). Major disadvantages include the corrosive nature of agents such as KOH, and the final carbon washing steps involve the use of toxic chemicals such as HCl or HF to etch potassium metal, alkali, and other impurities.

When preparing heteroatom‐doped (N, S, P, B, etc.) porous carbons, chemical activation (typically using KOH, NaOH, H_3_PO_4_, ZnCl_2_, or K_2_CO_3_) is the most common strategy to achieve a high specific surface area (SSA > 2000 m^2^ g^−1^) and well‐developed porosity, as summarized in Table 8 below [198]. However, the presence of heteroatoms dramatically changes how the activator works compared with pure carbon precursors, because the activator not only etches the carbon lattice but also reacts with the heteroatom‐containing fragments, which strongly influences the final pore size distribution and SSA.

**TABLE 8:** Summary of the effect of heteroatoms on activation chemistry in the synthesis of porous carbons.

Heteroatom	Common Dopant Sources	Preferred/Most Used Activator	Typical Activation Temp. (°C)	Specific Surface Area (SSA)	Dominant Pore Size	Effect of Heteroatom on Activation and Pore Development	Heteroatom Retention After Activation	Notes / Typical Observations
N	Urea, melamine, PANI, biomass (proteins), MOFs/COFs	KOH (most common), ZnCl_2_	700–900	Very high: 2200–4000 m^2^ g^−1^	Micropores (0.7–2 nm) + small mesopores (2–4 nm)	Strong reaction with KOH + N → HCN, NH_3_, cyanate species → intense extra gas evolution → higher SSA + widened micropores	Low–moderate (20–50% of initial N remains)	Highest SSA among single‐doped systems; bimodal pore distribution very common
S	Thiophene, sulfur powders, biomass (lignin, cysteine)	KOH, ZnCl_2_	750–900	Moderate–high: 1500–2800 m^2^ g^−1^	Small–medium mesopores (3–10 nm)	Forms COS, H_2_S, K_2_S → creates larger pores; less micropore development than N‐doping	Moderate (40–70%)	More mesoporous character; ZnCl_2_ preserves more S than KOH
P	Phytic acid, phosphoric acid, triphenylphosphine	H_3_PO_4_ (self‐activation), KOH	700–950	Moderate: 1000–2200 m^2^ g^−1^	Uniform wide micropores and small mesopores (1.5–4 nm)	Phosphate/polyphosphate esters act as spacers → very uniform pore size; less aggressive etching	High (60–90%)	Excellent pore uniformity
B	Boric acid, H_3_BO_3_, phenylboronic acid	KOH	700–850	High: 2200–3400 m^2^ g^−1^	Micropores + small mesopores (0.8–3 nm)	B protects some carbon layers from excessive etching → retains more microporosity	Moderate–high (50–80%)	Often co‐doped with N; helps preserve micropores compared to pure N‐doping
N+S	Thiourea, cysteine, sulfonated polymers	KOH	750–900	Very high: 2800–4000 m^2^ g^−1^	Hierarchical: micro + meso (2–10 nm)	Stepwise release of N‐ and S‐gases → broadens pore size distribution dramatically	Low–moderate (N: 20–40%, S: 40–60%)	hierarchical porous carbons
N+P	Phytic acid + gelatin/urea, ammonium polyphosphate	H_3_PO_4_ or KOH	800–950	High: 2000–3500 m^2^ g^−1^	Micropores + small mesopores (1–5 nm)	Synergistic effect: P creates uniform pores, N boosts SSA	Moderate–high (N: 30–60%, P: 70–90%)	Good charge storage ability
N+B	PVP + boric acid, ZIF‐8 derived	KOH	700–850	High: 2500–3800 m^2^ g^−1^	Bimodal (0.8 nm + 2–4 nm)	B limits over‐etching of N‐rich zones → better micropore retention than pure N‐doping	Moderate (N: 40–70%, B: 60–85%)	Good balance between SSA and remaining doping level

In heteroatom‐doped porous carbons, the activator not only etches carbon but also reacts vigorously with the heteroatom functional groups, releasing additional gases (HCN, NH_3_, COS, H_2_S, PO_x_, etc.). This almost always leads to: higher SSA than the undoped analog (often >2000 m^2^ g^−1^), broader pore size distribution (smaller mesopores 2–8 nm), and significant loss of the heteroatom during activation (trade‐off between porosity and doping level) [117]. Therefore, when designing heteroatom‐doped porous carbons, the choice and amount of activator must be adjusted according to the desired balance between maximum SSA, hierarchical porosity, and remaining heteroatom.

##### 3.3.1.2 Templating Methods

Templating process involves the use of agents such as hard‐template—also called exo‐templating (using inorganic oxides and metal salts)—soft templates (or endo‐templating), such as block copolymer or amphiphilic surfactants, and self‐templates [54, 199]. The templating strategy offers an opportunity to precisely tune the pore size and architecture, which enables tailored material properties.

###### 3.3.1.2.1 Hard Templating

**FIGURE 13:**
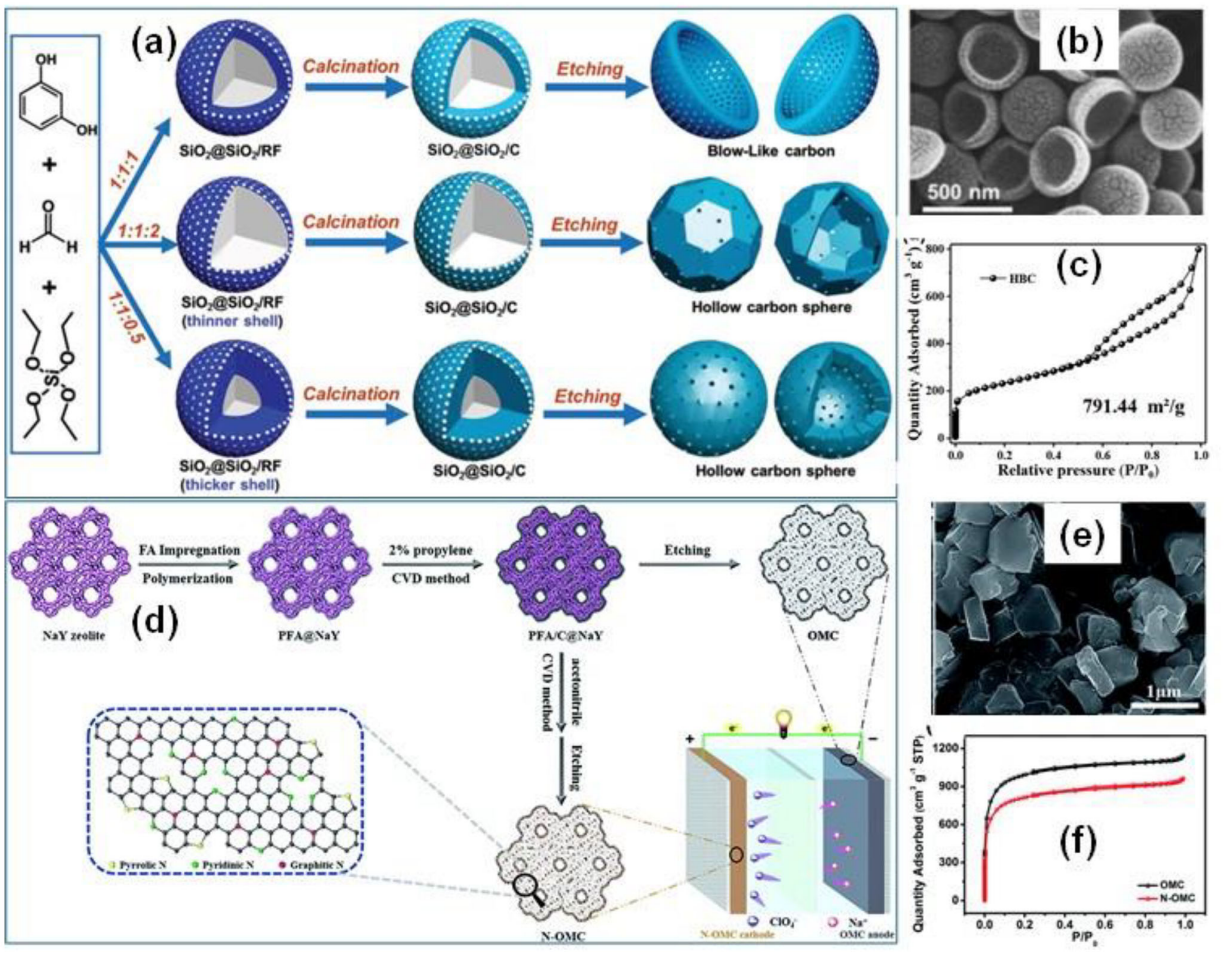
(a) Schematics of the formation process of hollow bowl‐like carbon (HBC) or hollow carbon sphere (HCS). (b) SEM image and (c) N_2_ adsorption isotherm of HBC. Reprinted with permission [70] Copyright (2020), John Wiley and Sons. (d) Schematic graphs of the synthesis of nitrogen‐doped ordered microporous carbon (N‐OMC). (e) SEM image and (f) nitrogen adsorption isotherm of N‐OMC. Reproduced from [55], the Royal Society of Chemistry.

The hard templating strategy involves the use of rigid, pre‐formed template/sacrificial template (such as mesoporous/colloidal silica, zeolites, or metal oxides) as a model to create porous carbon. One major merit of hard templating is its precise and reproducible pore structure that is suitable for fundamental studies of porous carbon materials. In the hard templating synthesis, two cases are possible. First, the template (such as nanoparticles or Si, Mg precursors), infiltrates into the matrix of the carbon precursor during carbonization and is subsequently removed via washing or etching, leaving a porous carbonaceous framework. For example, Fei et al. [70] synthesized Hollow Bowl‐Like Carbon (HBC, Figure 13a) via the use of tetraethyl orthosilicate (TEOS) as a source for SiO_2_ hard template and resorcinol as carbon precursor to obtain silica core‐shell structured nanospheres. The obtained spheres were pyrolyzed at 700°C for 2 h under Ar flow and then washed with 40% HF to etch silica from the carbonized nanospheres. The carbonized nanospheres have a hollow‐like structure (diameter of ∼300 nm, Figure 13b) resembling deflated volleyballs. Their N_2_ adsorption‐desorption curve shows a type IV‐ hysteresis loop at 0.5–1.0 P/P_0_ pressure range, indicating significant mesopore presence (Figure 13c). They show a BET specific surface area of 791 m^2^ g^−1^ and a total pore volume of 1.22 cm^3^ g^−1^. The full cell assembly consisting of MoSe_2_@HBC negative electrode and HBC positive electrode delivered a specific capacity of 47.2 mAh g^−1^ at 0.1 A g^−1^, which can still achieve 24.1 mAh g^−1^ ^g−1^ at a high current density of 20 A g^−1^ in 1 M NaClO_4_ in PC with 5 wt% FEC electrolyte. The authors attributed the electrochemical performance to the similar bowl‐like structures of both the negative electrode and positive electrode, which effectively enhance electrolyte infiltration and ion transfer to the porous surface.

Similarly, Qiu et al. [139] constructed Hierarchical Porous Hollow Carbon Bowls (HPCB) using Tetrapropyl orthosilicate (TPOS) template and resorcinol as the carbon precursor, which achieved a SSA between 1774 and 2189 m^2^ g^−1^. Also, Thangavel and co‐workers [143] prepared graphene hollow nanospheres using tetraethyl orthosilicate (TEOS), graphene oxide (GO), thiourea, and an ammonium hydroxide catalyst. The resulting nitrogen/sulfur co‐doped graphene hollow nanospheres (NS‐GHNS) exhibited a BET specific surface area of ∼320 m^2^ g^−1^ and a pore volume of 1.32 cm^3^ g^−1^. The NS‐GHNS positive electrode delivers discharge capacities of approximately 52 mAh g^−1^ at 0.2 A g^−1^ and 19 mAh g^−^^1^ at 20 A g^−1^ in 1 M NaClO_4_ EC/DEC electrolyte. The authors attribute the capacity to the porous morphology, characterized by numerous mesopores, which enhances the adsorption/desorption process. Additionally, pseudocapacitive interactions between sodium ions and oxygen functionalities, as well as sulfur heteroatoms, contribute to the performance. Metal oxides like MgO [200, 201], TiO_2_ [202], Al_2_O_3_ [203], ZnO [204], and Fe_2_O_3_ [205] are also effective hard templates for synthesizing porous carbon, with MgO being particularly prominent. MgO is simple to prepare, easy to remove, and widely used in porous carbon synthesis [200, 201]. For example, Mo and Wu^204^ prepare heteroatom‐doped hierarchical porous carbon with MgO as a template and polyacrylamide as a carbon source. They were carbonized between 600 and 800°C and washed with acid and water.

In the second case, infiltration of mesoporous silica or microporous zeolites into carbon sources is done, which leads to a carbon replica with the opposite structure of the original template. For example, Li et al. [55] synthesized nitrogen‐doped ordered microporous carbon (N‐OMC, Figure 13d) using furfuryl alcohol (FA) and propylene as liquid and gaseous carbon sources, respectively, with commercial NaY zeolite as the hard template. The mixture was heat‐treated at 700°C under a nitrogen atmosphere. The as‐prepared porous carbon exhibited an analogous irregular shape and dimensions as a result of the hard template (Figure 13e). N‐OMC shows type‐I N_2_ adsorption behavior with a sharp knee, flat adsorption plateau, and no significant hysteresis, indicating a microporous carbon structure with a SSA of 2791 m^2^ g^−1^ (Figure 13f).

A major shortcoming of the hard templating route involves the use of hazardous chemicals such as hydrofluoric acid (HF) or concentrated NaOH for template removal.

###### 3.3.1.2.2 Salt Templating

**FIGURE 14:**
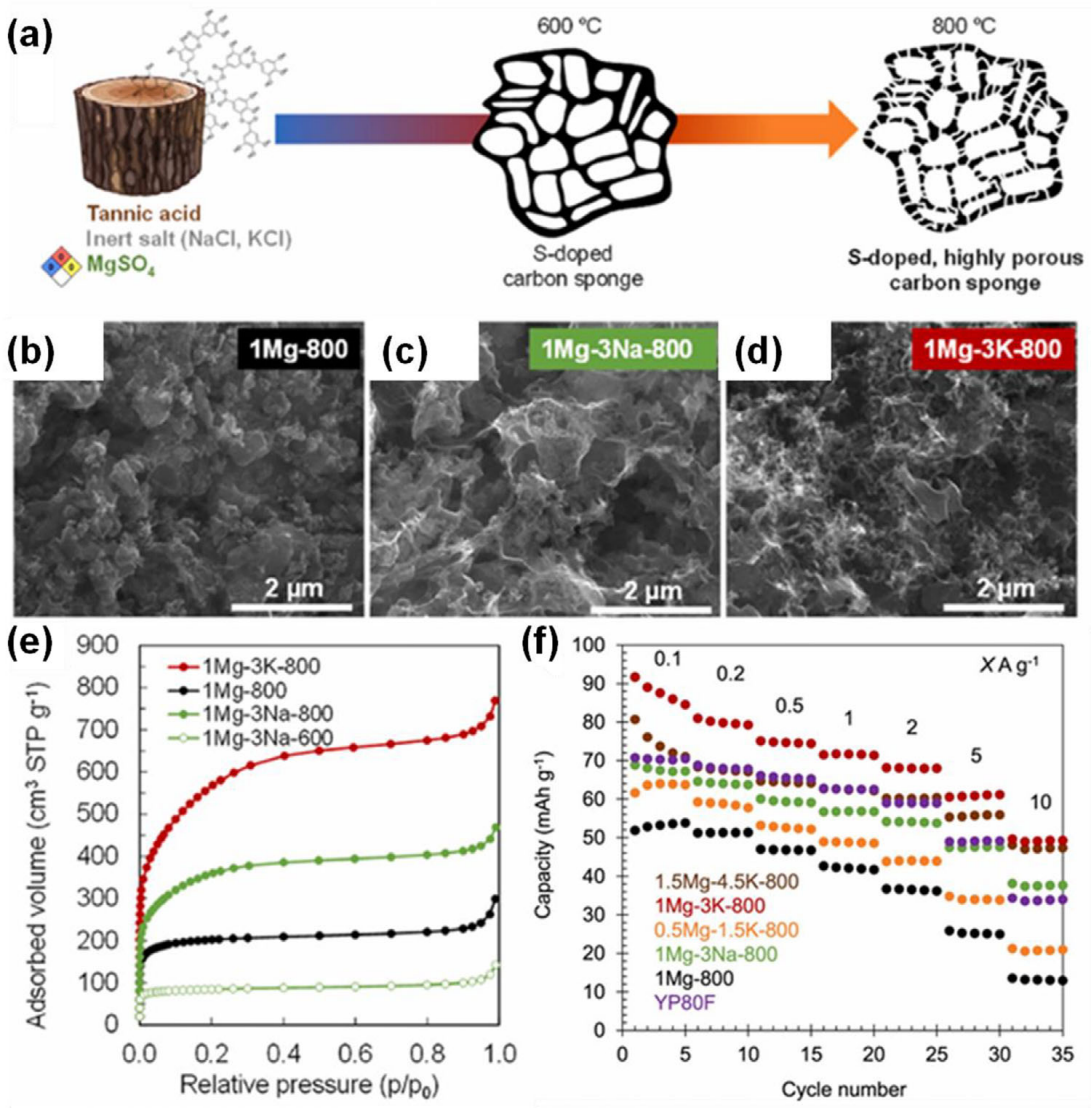
(a) Synthesis route of foam like porous carbon from salt templated tannic acid, SEM image of obtained porous carbon (b) without template, (c) with NaCl salt, and (d) KCl salt, (e) N_2_ adsorption isotherms and (f) electrochemical performance of the synthesized porous carbon vs. Na metal in 1 M NaClO_4_ (EC:DEC) electrolyte. Reprinted with permission [52] Copyright (2019), Elsevier.

The salt templating method involves the use of water‐soluble inorganic salts such as NaCl, KCl, ZnCl_2_, FeCl_3_, LiCl, or CsCl as porogens mixed with carbon precursors, pyrolyzed, and then removed by dissolution to generate pores. This is a simpler method than the hard templated approach. It also offers tunable porosity by varying the salt type, size, and concentration. For instance, Paya et al. [52] recently constructed sulfur‐doped porous carbon using tannic acid as carbon precursor, MgSO_4_ as S dopant, and inert salt (NaCl/KCl) as salt template porogens. The mixture was heated at temperatures between 600 and 800°C, under nitrogen flow (Figure 14a). The product obtained was washed with diluted HCl and distilled water to obtain a thin carbon foam morphology (Figure 14b–d). The authors noted that increasing carbonization temperature from 600 to 800°C enhances nitrogen adsorption at low relative pressures and widens the knee below 0.4 P/P_0_, indicating micro‐ and mesopore formation above 600°C. Also, the total pore volume and SSA rise from 0.16 cm^3^ g^−1^ and 300 m^2^ g^−1^ for 1Mg–3Na‐600 to 1.07 cm^3^ g^−1^ and 2040 m^2^ g^−1^ for 1Mg–3K‐800 (Figure 14e). The synthesized materials delivered a maximum specific capacity of 92 mAh g^− 1^ at 0.1 A g^−1^, in 1 M NaClO_4_ (EC:DEC) electrolyte, which is higher than that of YP80F commercial activated carbon (∼70 mAh g^−1^) with a SSA of 2300 m^2^ g^−1^, as shown in Figure 14f. The authors attribute the enhanced performance to the high specific surface area, optimal micropore size, micro‐mesoporous structure, sponge‐like morphology, and high electronic conductivity. Similarly, Liu et al. [159] obtained ultrathin carbon film (UCF) as a positive electrode for SIC via the salt templating of carboxymethyl chitosan with FeCl_3_·6H_2_O, roasted at 900°C, under argon flow. The obtained carbon film displays graphene‐like agglomerations due to iron's catalytic role during preparation. They have a BET SSA of 351.7 m^2^ g^−1^ and show excellent cycling stability, with a discharge capacity of 156.32 mAh g^−1^ and 98.42% capacity retention after 2500 cycles at 1 A g^−1^ in 1 M NaPF_6_ EC:PC electrolyte. The high capacity and retention are due to UCF's nanoporous structure, large surface area, and lattices of short‐range order, enhancing adsorption ability and rate capability.

###### 3.3.1.2.3 Soft Templating

Soft templating strategy relies on self‐assembly of amphiphilic molecules such as copolymers or surfactants, with carbon‐based precursors (typically phenolic resin), which decompose upon thermal treatment to obtain mesoporous carbon. The soft templating involves the self‐assembly of surfactants to co‐assemble into micelles or mesophases via hydrogen bonding or electrostatic interactions. It offers advantages such as simplicity, time efficiency, and convenient removal of the template by simple thermal annealing during the pyrolysis step. For example, Moussa and colleagues [206] synthesized nitrogen‐doped mesoporous carbon involving the co‐assembly of green phenolic resin carbon precursor (Phloroglucinol and Glyoxylic acid), amphiphilic polymer soft template (Pluronic F‐127), and Guanine as the nitrogen precursor, in a water/ethanol mixture. The presence of the soft template helps in the mesopore formation during thermal annealing (600–900°C). The obtained porous carbon showed a maximum BET SSA of 570 m^2^ g^−1^. Adeniji et al. [23] combined a soft template with a salt template to increase the SSA and to tune the textural and structural properties. The obtained nitrogen doped porous carbon (NDPC) shows SSA between 1078 and 2412 m^2^ g^−1^, micropore volume between 0.41 and 0.86 cm^3^ g^−1^ and mesopore volume between 0.22 and 0.59 cm^3^ g^−1^. The resulting doped porous carbon gave a discharge capacity of 83–159 mAh g^−1^ at 0.05 A g^−1^ in 1 M NaPF_6_ in EC:DMC electrolyte. Also, the authors demonstrated through positive linear correlations that the discharge specific capacity is driven by a large specific surface area and significant microporous volume with suitable pore sizes, while capacity retention is enhanced by mesoporous volume and graphitic domains.

Also, Phan et al. [138] synthesized ordered mesoporous carbon with a combination of both hard (tetraethyl orthosilicate) and soft (triblock copolymer P123) templates, thermally treated at 600–900°C under argon flow. The obtained carbon powder was washed with 2 M NaOH and deionized water to remove the silica template. The developed mesoporous carbon showed BET SSA of 944–1090 m^2^ g^−1^ and with a total volume of 1.1–1.2 cm^3^ g^−1^. The ordered mesoporous carbon delivered a discharge capacity of ∼47 and ∼13 mAh g^−1^ at 0.25 and 2 A g^−1^, respectively, in 1 M NaClO_4_ EC/DMC in electrolyte. The authors ascribed the performance to high‐surface‐area mesoporous carbons with wider pores (>4 nm), which likely provide more active sites for efficient ion adsorption and desorption.

###### 3.3.1.2.4 Self‐Templating

**FIGURE 15:**
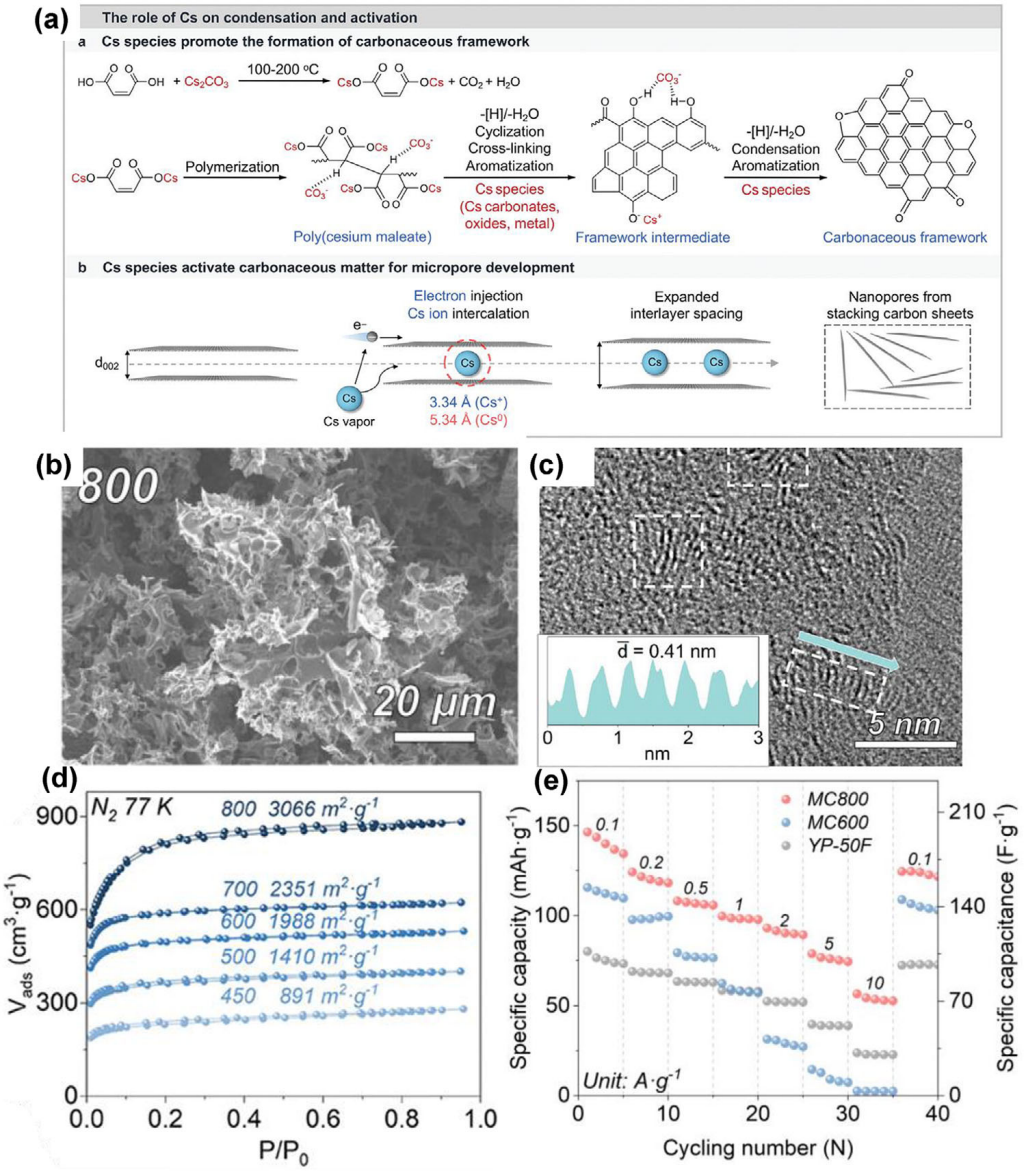
(a) Synthesis route of self‐templated porous carbon from maleic acid and Cs_2_CO_3_ alkali, (b) SEM micrograph and (c) TEM image of MC800 porous carbon (d) N_2_ adsorption isotherm of MC porous carbon at different temperatures and (e) Electrochemical performance of MC porous carbon and YP‐50F commercial activated carbon vs. Na metal in 1 M NaPF_6_ in VC: DMC (50: 50 vol%) with 5 wt% FEC electrolyte. Reprinted with permission [54] Copyright (2024), John Wiley and Sons.

In this method, the carbon precursor itself or the intermediates formed during pyrolysis play the role of an internal template to generate porosity without the need for an external template. Metal organic precursors are commonly used as self‐templates [207]. In the work of Li et al. [54], porous carbon was produced via a non‐etching templating strategy by mixing Cs_2_CO_3_ and maleic acid (MA) in a molar ratio of 2:1 or 1:1 via polymerization and crosslinking aromatization (Figure 15a). The obtained polymers were heated at 800°C for 2 h under a nitrogen atmosphere. The resulting products were washed with 1 M HCl and rinsed with deionized water, exhibiting a smooth scaffold with 1–5 µm cavities with macroscopically homogeneous layers (Figure 15b,c). The carbon product gave a yield up to 25% with an ultra‐high SSA of 3066 m^2^ g^−1^ and delivers a maximum specific capacity of ∼147 mAh g^−1^ at 0.1 A g^−1^ (Figure 15d,e) in 1 M NaPF_6_ in VC: DMC (50:50 vol%) with 5 wt% FEC electrolyte.

#### 3.3.2 Heteroatom Doping

Heteroatom doping is a key approach for enhancing carbon material performance. Incorporating heteroatoms like nitrogen, sulfur, phosphorus, or boron with similar atomic radii, orbitals, electronegativity, and charge density to carbon creates heteroatom‐doped porous carbon. These heteroatoms alter electron density, boost electron mobility, increase defect sites, and introduce new active centers, thereby enhancing electron transfer reactions. Heteroatoms are incorporated along the edges of carbon surfaces. These groups can tune the physicochemical properties of carbon. Heteroatom doping provides an opportunity to enhance pseudo‐capacitance [208]. In addition, they improve the wettability and conductivity of the doped carbon materials, the ion diffusion/adsorption, and improve charge propagation, leading to higher capacity and retention [209]. There are two typical ways of introducing heteroelements into porous carbons: post‐treatment and in‐situ doping. The post‐treatment process entails the thermal treatment of reagents containing heteroatoms alongside undoped porous carbon. At elevated temperatures, these heteroatoms integrate into the porous carbon structure, modifying its properties. In‐situ doping involves the direct carbonization of heteroelement‐containing carbon precursors. This approach offers better homogeneous distribution, higher amount, and stability of heteroatoms in the carbonaceous network [198, 210].

Oxygen is the inherent dopant present in porous carbon due to its natural presence in all precursors, especially in biomass and biopolymer precursors. These precursors naturally contain oxygen‐rich functional groups such as hydroxyl (─OH), carbonyl (C═O), and carboxyl (─COOH), which persist in varying degrees during carbonization processes like pyrolysis or hydrothermal carbonization, embedding oxygen atoms within the carbon matrix. For instance, biomass precursors typically yield oxygen contents ranging from 5 to 20 wt% in the resulting carbon, depending on the precursor type and processing conditions [211, 212]. This inherent doping enhances the material's surface chemistry without additional steps, contributing to improved wettability and electrochemical properties. Additional oxygen can be introduced by post‐treatment: chemical (HNO_3_, H_2_SO_4_, and H_2_O_2_) or thermal (air, O_2_, O_3_, CO_2_). These treatments can increase oxygen content by 5–15 wt%, but they require careful control to avoid structural collapse or excessive defect introduction [211, 212].

Nitrogen‐doped porous carbon is the most commonly prepared doped carbon material. It is a versatile method in tuning the physico‐chemical properties of carbon materials as a result of their high electrochemical activity. The presence of lone pairs of electrons leads to their higher electronegativity value (3.04) compared to carbon atoms (2.55). They become more electron dense, causing a favorable interaction with positive species. For instance, the nitrogen atoms can introduce pseudocapacitive sites, improving interactions with ions (e.g., H^+^, Li^+^, or Na^+^) in the electrolyte. This leads to higher specific capacitance and better charge storage performance. Quaternary and pyridinic nitrogen sites attract cations from aqueous or organic electrolytes, enhancing charge transfer. In addition, N‐doping can also offer improvement in the electrical conductivity and wettability of carbon materials. N‐doped carbon can be achieved via both post‐treatment and in situ strategies. Typical doping agents in the synthesis of nitrogen‐doped carbon include the use of urea [132], NH_3_ [74, 109, 124], melamine [125, 130], and guanine [23].

S‐doped carbon has attracted significant attention in energy storage technologies. Sulfur, recognized as a highly reactive element for heteroatom doping, owes its reactivity to unpaired electrons and a broader bandgap from its electron‐withdrawing properties. It possesses a minor electronegativity variation with carbon (2.58 for S and 2.55 for C), and its readily polarizable nature [213, 214]. These characteristics enable S‐doped carbon to exhibit enhanced electrochemical properties, such as improved electrical conductivity, increased surface area, and a higher density of active sites for ion adsorption or redox reactions [87]. Sulfur is present in the waste streams of various industries such as leather processing, oil refining, and natural gas production. It is generated in substantial amounts as a byproduct, especially in nations rich in petrochemical resources like Qatar [215]. Utilizing sulfur in energy storage applications, including supercapacitors, promises to decrease sulfur waste and provide more cost‐effective solutions over time. Typical doping agents in the synthesis of sulfur‐doped carbon include the use of elemental sulfur [87], H_2_S [216], MgSO_4_ [87], thiourea [217], thiophene [218], and l‐cysteine [219].

**FIGURE 16:**
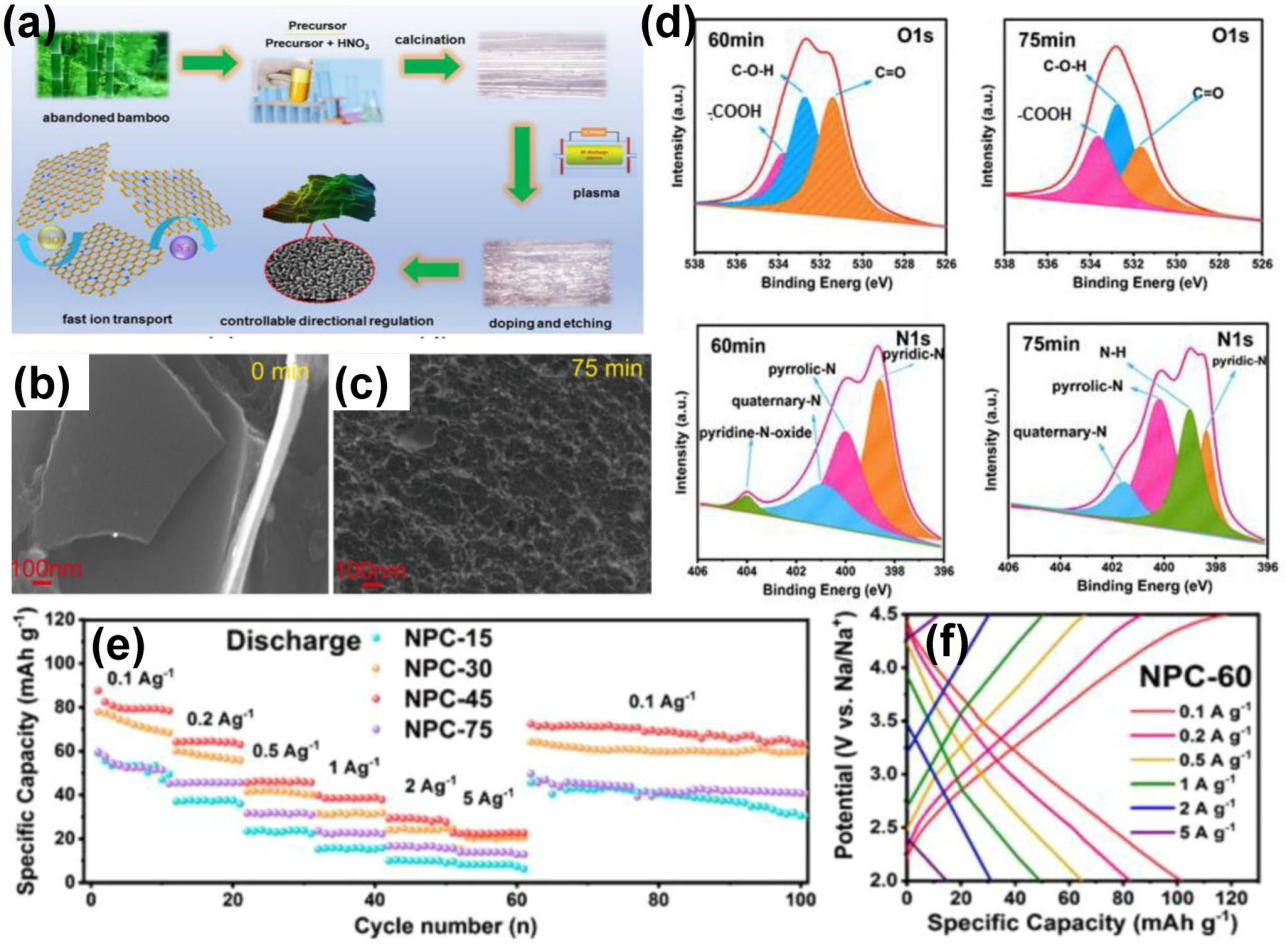
(a) Schematic diagram for the synthesis of nitrogen‐doped porous carbon positive electrode (NPC) from bamboo and NH_3_ plasma. SEM images of (b) non‐plasma and (c) NH_3_ plasma‐treated porous carbon, (d) O 1s and N 1s high‐resolution XPS spectra of NPC‐60 and NPC‐75. Electrochemical performance of (e) NPC series and (f) NPC‐60 vs. Na metal half‐cell in NaClO_4_ electrolyte. Reprinted with permission [109]. Copyright (2021), Elsevier.

As shown in Figure 16, Cai and co‐workers [109] developed N/O‐doped porous carbon (NPC) using NH_3_ plasma treatment on CO_2_‐activated shredded bamboo, as described in their study (Figure 16a). The treatment, conducted at 300 W and 90 MPa for 15 to 75 mins, altered the material's properties. As treatment time increased from 15 to 60 mins, the NPC exhibited increased SSA (577 to 1022 m^2^ g^−1^), pore size (2.63 to 3.02 nm), nitrogen content (3.91 to 4.7%), and hydrophilicity (contact angle decreased from 15° to 7°). However, at 75 mins, excessive etching reduced the surface area to 899 m^2^ g^−1^. Also, a decreased level of structural ordering was observed as the NH_3_ plasma etching progressed (Figure 16b,c). The plasma treatment shifted chemical compositions, converting pyrrole‐N to pyridine‐N and ─C─O─ to C═O, with NPC‐60 showing the highest pyridinic‐N and C═O content, enhancing active sites for charge storage (Figure 16d).

In Na metal half‐cell tests, with NaClO_4_ electrolyte, NPC‐60 delivered the highest specific capacity (101 mAh g^−1^ at 0.1 A g^−1^), as shown in Figure 16e and f. NPC‐60 also maintained a capacity of 79 mAh g^−1^ after cycling at 5 A g^−1^, attributed to pyridinic‐N and C═O facilitating anion adsorption and pseudocapacitive storage.

**FIGURE 17:**
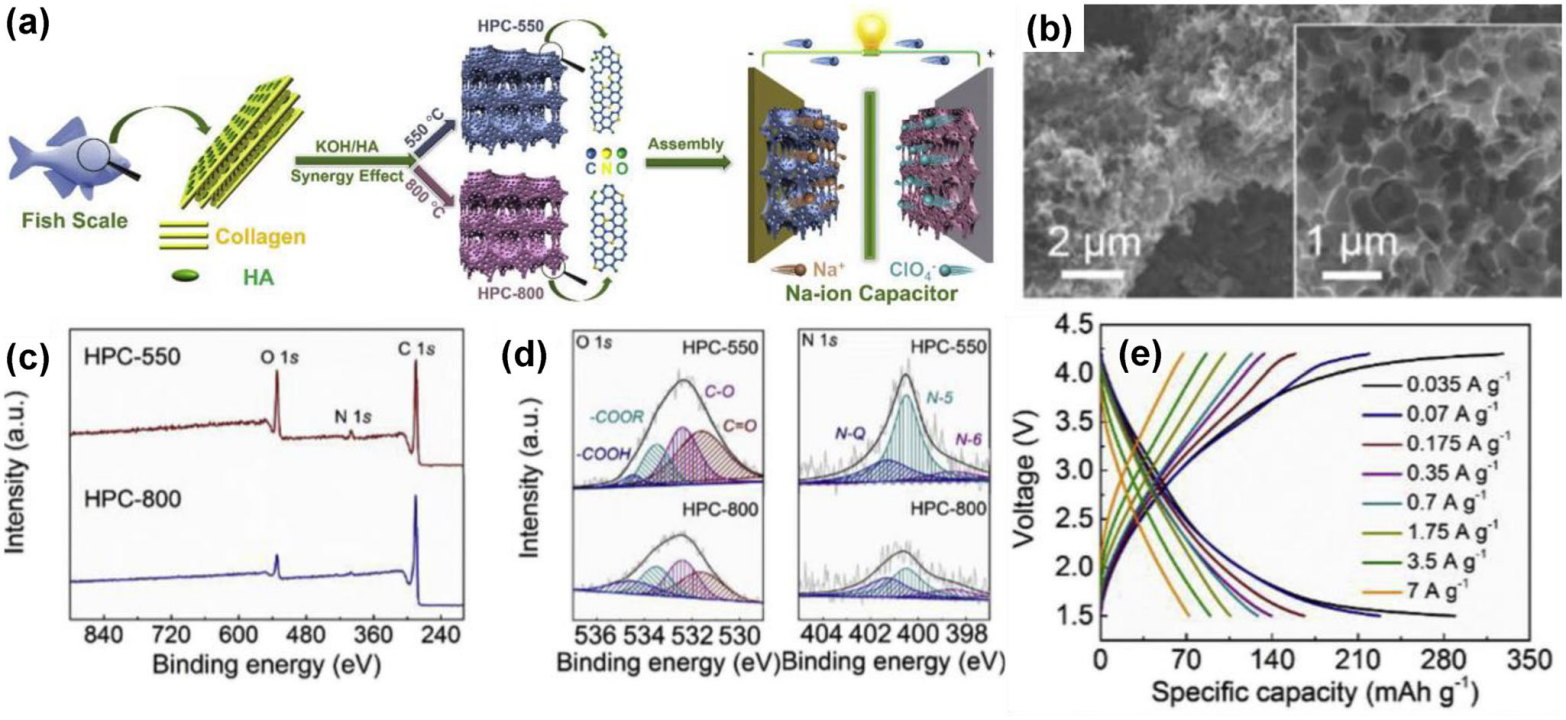
(a) Schematic diagram of nitrogen doped hierarchical porous carbon (HPC‐ 800) synthesis from the activation of fish scale, (b) SEM images of HPC‐800 positive electrode, (c) XPS survey spectra along with (d) O 1s and N 1s high resolution XPS spectra of HPC‐550 negative electrode and HPC‐800 positive electrode, and (e) Electrochemical performance of HPC‐800 positive electrode vs. Na metal half‐cell in 1 M NaClO_4_ PC:EC:FEC (1:1:0.05 in vol %) electrolyte. Reprinted with permission [157] Copyright (2019), Elsevier.

Also, Liu et al. [157] fabricated hierarchically porous carbons (HPCs) with N/O heteroatoms via the thermal treatment of fish scale biopolymer with KOH, at 800 °C under Ar flow (Figure 17a). The developed porous carbon exhibited a honeycomb‐like structure (Figure 17b), with rich interconnected macropores and with an extremely high SSA of 3285 m^2^ g^−1^. In terms of surface chemistry, the content of N and O atoms of porous carbon is 2.7 and 7.6 at. %, respectively. The material is rich in both nitrogen (pyridinic (N‐6), pyrrolic/pyridine (N‐5), and quaternary (N‐Q) nitrogen) and oxygen groups (C═O, C─O, ─COOR, and ─COOH), as shown in Figure 17c,d. The developed porous carbon delivered a large capacity of 128 mAh g^−1^ at 0.7 A g^−1^, which is still maintained at 72 mAh g^−1^ even at 7 A g^−1^ (Figure 17e) vs. Na metal half‐cell in 1 M NaClO_4_ PC:EC:FEC (1:1:0.05 in vol %) electrolyte. The authors ascribed the improved performance to redox reactions of the electrolyte with nitrogen/oxygen‐containing functionalities that contribute to pseudocapacitance.

Similarly, Ren et al. [87] prepared sulfur‐ and oxygen‐doped porous carbon (SOPC) through an in‐situ doping method. Carbon precursors (methyl cellulose and β‐cyclodextrin) and a salt template (NaCl) were mixed, frozen, and pre‐carbonized at 400°C. The resulting material was activated with KOH at 700°C in an N_2_ atmosphere to yield Oxygen‐rich porous carbon (OPC). OPC was then mixed with elemental sulfur at mass ratios of 1:10, 1:20, and 1:30, and heated at 550°C under N_2_ gas to produce SOPC. The material displays an interconnected three‐dimensional honeycomb pore structure. The authors note that as the sulfur doping ratios increased, both the SSA and total pore volume of OPC decreased progressively: OPC (1776, 1.61) and SOPC‐30 (1031 m^2^ g^−1^, 0.78 cm^3^ g^−1^). Elemental analysis revealed that sulfur content rose from 5.8 to 10.2 at.%, while oxygen content declined from 11.8 to 7.4 at.%. The material delivered a maximum discharge specific capacities of 178.9 and 63.8 mAh g^−1^(SOPC‐20) at 0.05 and 10A g^−1,^ respectively, in 0.8 M NaPF_6_ (EC:DEC) electrolyte. The authors reported that the specific capacity increases with the S‐doping and reaches a plateau when the S and O content are at 7 and 9.8 at.%, respectively. Thus, the doping treatment can enhance the charge transport capability and ion diffusion rate of the electrode materials, resulting in increased specific capacity. Furthermore, Thangavel and colleagues [174] synthesized nitrogen/sulfur co‐doped watermelon‐derived porous carbon (NS‐WDC) using a multistep process. Watermelon seeds were washed, annealed at 600°C under argon, activated with KOH at 800°C, and doped with nitrogen and sulfur using thiourea, followed by annealing at 600°C. XPS confirmed 4.13 wt.% N and 3.31 wt.% S content. Compared to non‐doped carbon, NS‐WDC showed improved wettability (contact angle: 34.2° vs. 45.6°) and conductivity (5.1 vs. 1.1 S cm^−1^). In a symmetric two‐electrode cell with 1 M NaClO_4_ in EC:DMC electrolyte, NS‐WDC exhibited superior specific capacitance (124 F g^−1^) compared to non‐doped carbon (59 F g^−1^) at 10 A g^−1^. The superior performance is attributed to the synergistic effects of nitrogen and sulfur doping in the carbon framework.

The performance of porous carbon in SICs can be enhanced by heteroatom doping with oxygen (O), nitrogen (N), and sulfur (S), as these introduce functional groups that contribute to pseudocapacitance through faradaic redox reactions, improved wettability, and enhanced conductivity. However, not all functional groups are equally active; the electrochemically active ones include quinone/carbonyl (for O), pyridinic and pyrrolic N (for N), and thiophene/sulfoxide/sulfone (for S). To selectively increase these active groups while minimizing inactive or unstable ones (e.g., carboxyl for O, which can cause self‐discharge), specific synthesis and post‐treatment strategies are employed. Below, we outline key methods tailored for electrochemical capacitor application, grouped by dopant type. These are drawn from established strategies in carbon material synthesis, focusing on selectivity through precursor choice, temperature control, activation techniques, and co‐doping.

##### 3.3.2.1. Oxygen Doping

**FIGURE 18:**
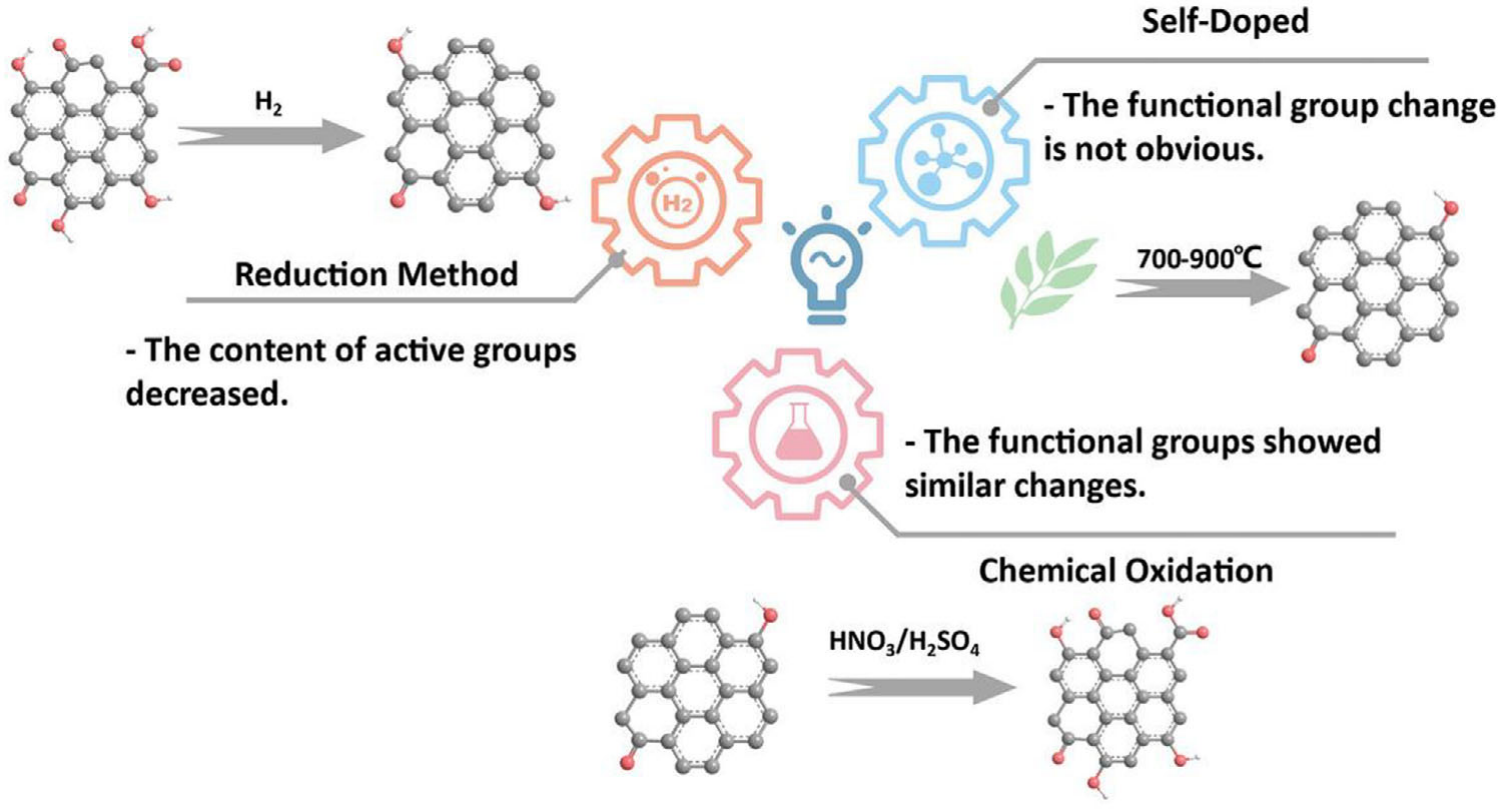
Common way of regulating oxygen‐containing functional groups on the surface of carbon materials. Adapted from [151] Copyright (2024) with permission from Wiley and Sons.

Oxygen functionalization primarily introduces stable carbonyl/quinone (C═O) groups that enable reversible redox reactions, significantly enhancing pseudocapacitance. Ideal O/C ratios range from 5–10 at%, favoring in‐plane quinone/hydroxyl over unstable out‐of‐plane carboxyl groups. Key methods include (Figure 18)

- Adjusting oxygen in precursors and carbonization temperature → limited effect, typically achieves low oxygen doping (<10 at%).
- Post‐oxidation with strong oxidants (e.g., conc. H_2_SO_4_, HNO_3_, or mixed acids) → introduces abundant oxygen groups and active sites, but significantly reduces electrical conductivity, harms electrochemical performance, and lacks selectivity (all oxygen groups increase together).
- Post‐reduction of oxygen‐rich carbon with reducing agents (e.g., H_2_‐Ar at high temperature) → restores conductivity and allows minor adjustment of functional‐group ratios by selectively removing less stable groups (carboxyl, hydroxyl), but is hard to control precisely and risks over‐reducing stable, desirable groups (e.g., carbonyl/quinone).

##### 3.3.2.2. Nitrogen Doping

Selective nitrogen doping targets electron‐donating pyridinic (N‐6) and pyrrolic (N‐5) configurations (optimal total N 5–10 at%) for pseudocapacitance and wettability, while minimizing less‐active quaternary‐N. Effective approaches involve pyrolysis of N‐rich precursors (urea, melamine, chitosan; low‐temperature <750°C favors N‐6/N‐5), post‐annealing in NH_3_ atmosphere or microwave‐assisted treatment, and N_2_/NH_3_ plasma functionalization.

##### 3.3.2.3. Sulfur Doping

Sulfur doping (1.9–8 at%) introduces conductive thiophene‐S and redox‐active oxidized‐S (sulfoxide/sulfone) species. Common strategies include hydrothermal treatment of carbon precursors with thiourea/Na_2_S (neutral pH favors thiophene‐S), high‐temperature carbonization of natural S‐containing biomass, and deliberate N/S or N/S/O codoping. Sulfur improves electrolyte wettability, anion attraction, and rate capability.

##### 3.3.2.4. General Strategies and Co‐Doping

Codoping (N/S, N/O, N/P, N/S/O) creates synergistic effects by combining electron‐donating and electron‐withdrawing dopants, reducing bandgap, and boosting quantum capacitance. Template‐assisted synthesis and controlled defect engineering enhance selectivity. Matching active functional groups to electrolyte type (quinone for acidic, pyridinic‐N for basic) is critical. Moderate doping levels, guided by DFT predictions and post‐annealing, typically improve specific capacitance by 20–50% without sacrificing conductivity or cycle life. Biomass‐derived carbons remain the most sustainable and cost‐effective starting platform for implementing these strategies.

Other heteroatoms, such as boron (B) and phosphorus (P), are also highly promising doping agents for porous carbon materials. They can effectively enhance pseudocapacitive charge storage by introducing additional active sites, modifying the electronic structure, and improving surface polarity and wettability. These dopants have been successfully employed in carbon‐based positive electrodes for various dual‐carbon hybrid systems (e.g., lithium‐ion capacitors and zinc‐ion capacitors). However, compared to N, O, and S doping, B‐ and P‐doping strategies remain relatively underexplored and rarely utilized in SICs, representing a valuable direction for future performance optimization in such devices.

**TABLE 9:** Selecting the appropriate pretreatment method for different types of precursors used in porous carbon synthesis.

Precursor type	Typical examples	Material compositions	Goal of pretreatment	Recommended pretreatment methods	Mechanism: How pretreatment influences later steps
Biomass	Wood, straw, rice husk, fruit peels, shells, seaweed	High ash (Si, K, Ca, Mg), lignin–cellulose–hemicellulose complex, moisture, volatiles	Remove/mineralize ash, open structure, homogenize composition	Water washing + acid washing (HCl, HF, H_3_PO_4_) Hydrothermal pre‐carbonization (HTC 180–250°C) Torrefaction (200–300°C in N_2_) Ball‐milling + alkali swelling	Acid washing dissolves alkali/alkaline‐earth metals → prevents catalytic graphitization and eutectic melting during high‐T activation Removes SiO_2_ (especially important for rice husk) → avoids blocking micropores HTC creates primary carbon skeleton + oxygen groups → better KOH/CO_2_ activation (more reactive sites) Reduces volatile loss during direct pyrolysis → higher carbon yield
Biopolymers	Cellulose, chitosan, chitin, alginate, lignin, proteins	Relatively clean, but crystalline domains, high oxygen content, some impurities	Increase reactivity, control cross‐linking, introduce N/S/P heteroatoms	Simple water/ethanol washing + drying Freeze‐drying or supercritical CO_2_ drying Pre‐oxidative stabilization (air, 200–300°C) Ionic coordination (e.g., Ca^2^^+^/Zn^2^^+^ crosslinking of alginate) Protonation (chitosan in acetic acid)	Freeze‐drying preserves nanofibrillar structure → direct template for mesopores Pre‐oxidation creates carbonyl/quinone groups → anchoring points for KOH → higher micropore volume Metal coordination forms uniform ion templates → after carbonization gives homogeneous metal oxide nanoparticles that can be etched to pores
Synthetic	PAN, pitch, phenolic resin, resorcinol–formaldehyde, PVC, PVDF, block copolymers	Controllable but sometimes low carbon yield, halogen or N content	Increase carbon yield, prevent melting, introduce porosity early	Pre‐oxidation/stabilization (PAN: 200–300°C in air) Hypercrosslinking (Friedel–Crafts for polystyrene) Foaming/blowing agent addition Emulsion/self‐assembly templating (block copolymers)	Pre‐oxidation of PAN creates ladder structure → no melting at 900°C, preserves fiber morphology Hypercrosslinking locks permanent microporosity before pyrolysis → micropores survive high‐T treatment Block‐copolymer self‐assembly gives ordered mesopores that remain after polymer burn‐out

**TABLE 10:** Summary of the key mechanism of how pretreatment controls the final porous carbon properties.

Effect	Pretreatment responsible	Impact on activation/carbonization
Ash/mineral removal	Acid washing (HCl/HF)	Prevents catalytic effects (K, Ca accelerate graphitization → loss of porosity); prevents pore blocking by molten salts
Creation of reactive oxygen groups	HTC, pre‐oxidation, HNO_3_ treatment	Oxygen groups react with KOH → CO_2_/CO evolution → micropores (K_2_CO_3_/K_2_O mechanism)
Primary carbon skeleton	Hydrothermal carbonization, torrefaction	Reduces mass loss during high‐T pyrolysis → higher yield; creates more homogeneous material
Preservation of nanostructure	Freeze‐drying, supercritical drying, pre‐stabilization	Prevents collapse of fibrils or gels → retains inherent meso/macropores
Early introduction of porosity	Hypercrosslinking, soft‐templating (Pluronic, etc.)	Porosity is “locked‐in” before high temperature → survives even aggressive KOH activation
Heteroatom doping control	Choosing chitosan (N), alginate + thiourea (N/S)	Pretreatment fixes N/S in the skeleton → remains after carbonization
Particle size and homogeneity	Ball‐milling, dissolution–reprecipitation	Smaller particles → better activator diffusion → more uniform activation

#### 3.3.3 Impurities Removal

Porous carbon materials used in electrochemical capacitors, derived from three main categories of precursors: biomass, biopolymers, and synthetic sources, present distinct impurity profiles, removal strategies, and effects on material performance.

Biomass‐derived porous carbons, sourced from abundant and sustainable plant wastes (such as garlic peel, rice husks, wheat straw, sugarcane bagasse, wood sawdust, and agricultural residues) or animal wastes, commonly contain high levels of inorganic impurities including metals like K, Ca, Mg, Si, P, heavy metals (Pb, Cd, Hg), ash, and activation byproducts (e.g., K_2_CO_3_) [220], as shown in Table 9. Organic residues such as pesticides or toxins may also persist. In the case of biopolymer‐derived porous carbons, extracted from natural polymers like cellulose, lignin, chitin/chitosan, starch, agar, gelatin, keratin, or alginate (often from biomass but purified), feature residual inorganics from extraction (e.g., ash, metals like Na, K, or Ca) [221], heteroatoms (N, P, or S) [92] if not fully purified, and impurities from separation processes. Synthetic precursors, such as phenolic resins, PAN, petroleum pitch, coal, or other synthetic polymers, generally offer higher initial purity with fewer natural contaminants. Impurities mainly arise from synthesis (e.g., water‐soluble ionic salts, unreacted monomers, solvents) or activation (e.g., K_2_CO_3_ by‐products, metal catalysts like Fe or Zn, ash from coal/pitch) [222, 223].

These impurities can block the pores, reduce surface area, and limit hierarchical porosity by introducing defects and excess oxygen [221]. Additionally, impurities may induce undesired side reactions with the electrolyte. Effective removal involves acid washing (HCl, HF, or HNO_3_), water/ethanol washing, thermal pre‐treatment, or chemical extraction before or after pyrolysis [224, 225], as shown in Table 10. Purification of raw or waste biomass precursors significantly improves the properties of derived carbon materials by removing organic and inorganic impurities. By minimizing impurities through purification, the resulting carbons exhibit higher SSA, increased pore volume, greater carbon content, more uniform and defect‐free structures, controlled and tunable porosity, and enhanced structural integrity, critical attributes for superior electrochemical performance [224, 225, 226].

The washing process serves as a conventional and effective method for stripping impurities from biomass precursors. By removing organic and inorganic contaminants, washing enhances the quality of the derived carbon materials, improving their electronic conductivity, surface area accessibility, and ion diffusion properties, while minimizing side reactions and inactive mass. As one of the pre‐carbonization steps (Figure 3), the washing process involves treatment with chemical agents such as solvents (water, isopropanol, acetone, and ethanol) and acids (H_2_SO_4_ and CH_3_COOH) as shown in Tables 3, 4, 5. These agents can be used individually or merged in a combination of steps. For instance, Cai et al. [109] prepared doped porous carbon from harvested bamboo, involving cleaning the exposed middle layer with deionized (DI) water. Nagmani et al. [223, 224] synthesized PC from jute fiber, wherein the raw jute fibers were pretreated with H_2_SO_4_ acid in deionized water (DI). Similarly, Zhao et al. [164] prepared porous carbons by initially washing Metaplexis japonica with acetone and DI water and further treatment with acetic acid aqueous solution. Separately, another study utilized a stepwise treatment involving hot water, isopropanol, and acetone to process goat skin biomass, effectively eliminating bacteria and parasites.

The post‐activation washing process is typically utilized in the purification and modification of materials following activation and templating. It helps in removing or etching impurities, enhancing porosity, and engineering the surface properties of porous carbon materials (Table 10). This process ensures that the newly formed micropores and mesopores are free of blockages. This maximizes the surface area and pore volume, which are critical for the enhanced electrochemical performance of the developed activated carbon [228] and prevents impurities from reacting with the electrolyte, causing initial leakage current and increased resistance [222]. Soluble impurities such as metal ions, salts, metal oxides, ash contents, or residual agents are mostly removed with the help of deionized/distilled water, ethanol, NaOH, H_2_SO_4_, HCl, and HF. The washing agent choice, concentration, as well as the treatment duration and sequence, could vary based on the type of carbon precursors, templated and targeted porosity. The activation of biomass with KOH activating agents or templated salt, such as ZnCl_2_, mostly requires the use of a combination of washing steps that necessitate the use of HCl acid and deionized water.

For example, Wang et al. [168] prepared activated carbon spheres via the KOH activation of carbon spheres derived from fruit waste. The obtained solid products were washed with 1 M HCl and deionized water until a pH 7 was reached. Also, Ajuria and co‐workers [33] prepared porous carbon based on raw olive pits activated with KOH and washed with HCl and water. Porous carbon prepared from peanut skins [49], activated with FeCl_3_ or KOH templated salts, was thoroughly washed with HCl solution and distilled water. A similar protocol was also followed when using Coconut shells/ZnCl_2_ [108] or abandoned bamboo/CO_2_ activator [109]. However, Kim et al. [178] obtained porous carbon via the activation of citrus peels with KOH at 800°C, and the obtained carbon product was washed using only distilled water and ethanol without using HCl acid. In the case of biopolymer‐based precursors such as starch [89, 90], sucrose [154], honey [156], cellulose [87], etc., they similarly follow the washing protocol as biomass activated with KOH/NaOH/FeCl_3_. For example, Gao et al. [89] synthesized KOH‐activated potato starch residue‐derived carbon materials (PSRC), the obtained final carbon products were stirred in 3M HCl to eliminate K compounds.

On the other hand, the use of synthetic precursors offers a variety of washing steps that include environmentally friendly steps (mostly water) to hazardous ones (such as HCl and HF solutions) to achieve efficient removal of unwanted layers or impurities in the final porous carbon materials. For instance, Paya et al. [121] prepared a salt‐templated‐based porous carbon from gluten/KCl salt/K_2_CO_3_ at 850°C under argon atmosphere, and the obtained carbon solids were washed several times with hot water to remove inorganic salt residues. In the work of Li et al. [122], porous carbon synthesized from KOH‐activated sodium ascorbate gave an activated product washed with 2 M HCl and subsequently washed with deionized water to reach neutral pH. In the case of synthetic precursors based on dolomite [128], resorcinol and formaldehyde [70], NH_2_‐MIL‐125(Ti) [142], graphene oxide [143] and hard templates (such as commercial NaY zeolite [55] and isobutyl‐triethoxysilane [126]), the washing process mostly involves the use of HF acid to etch the obtained carbon product and remove residues and templates. For example, Li et al. [126] obtained nitrogen‐doped ordered microporous carbon (N‐OMC) from commercial NaY zeolite hard template with furfuryl alcohol carbon precursor. The final carbon products were washed with HF and HCl to remove the NaY template. Also, Lin et al. [126] fabricated ultrathin porous carbon nanosheet 3D architectures (PCNS) using isobutyltriethoxysilane precursor at 900°C under Ar, and the obtained carbon sample was washed with water, 10 wt% HF aqueous solution, deionized water, and ethanol to remove silicon oxide and as‐formed Li‐silicate. Similarly, Wang and co‐workers [128] prepared hierarchical porous carbon nanoplates (HCNPs) from dolomite by calcination and a hydration process involving in situ activation with K_2_CO_3_; the obtained carbon products were washed in 5 M HCl to remove Ca and Mg, and then using 10 wt% HF to remove silicon traces.

In general, the preparation of porous carbon involves a multifaceted approach that integrates various pre‐treatment and post‐treatment strategies to tailor the material's properties for specific applications. Pre‐treatment methods such as washing, polymerization, electrospinning, hydrothermal carbonization, and thermal treatment lay the foundation for creating a suitable carbon structure from diverse precursors. Subsequent post‐treatment processes, including washing/etching with H_2_O, HCl, or HF, grinding/milling, activation with KOH or CO_2_, and heteroatom doping, further enhance the porosity, surface chemistry, and functionality of the carbon material. These integrated approaches, as depicted in Figure 6, enable the production of porous carbon with tailored characteristics for enhanced performance.

**FIGURE 19:**
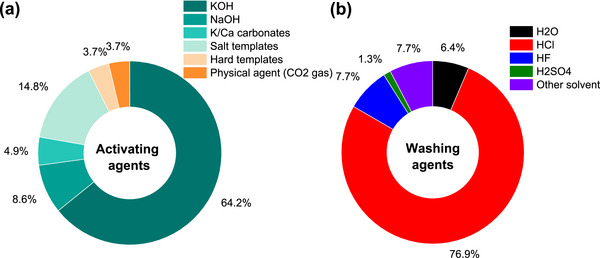
Distribution of (a) activating agents used in the synthesis of porous carbon and (b) washing agents employed post‐activation. The percentage values are derived from the publications analyzed in Tables 3, 4, 5.

Figure 19a shows a distribution summary of the various activating agents adopted in the synthesis of PC, typically KOH dominates as the primary activating agent, alongside NaOH, K carbonates, salt‐templates, and hard‐templates. The widespread use of KOH is undeniable; however, it comes with a major drawback, with its highly corrosive nature. This thus calls for the use of alternatives like citrates and carbonates‐based activating agents [199]. Their adoption could pave the way for more environmentally benign processes. In the post‐activation washing phase, HCl is mostly the preferred choice (Figure 19b), ahead of H_2_SO_4_, HF, H_2_O, and other solvents. Despite its high effectiveness, there is a need to explore greener alternatives that align with sustainability goals. The combination of green and sustainable precursors, paired with activating agents like citrates or carbonates, and washing with H_2_O or mild organic solvents, offers a hopeful path. It is posed to reduce environmental impact but also sets a precedent for cost‐effective, eco‐conscious carbon production.

## 4 Properties of Porous Carbon

Many factors are known to impact the formation and general properties of porous carbon, such as its morphology, structural properties, texture, and surface functionalities. In this regard, the different precursor types and thermal process parameters and pathways determine the final carbon architecture. This section explains how the different carbon precursor properties and processing parameters affect the porous carbon properties. This review focuses on the three main precursor groups: synthetic, biopolymer, and biomass.

### 4.1 Porous Carbon Morphology

**FIGURE 20:**
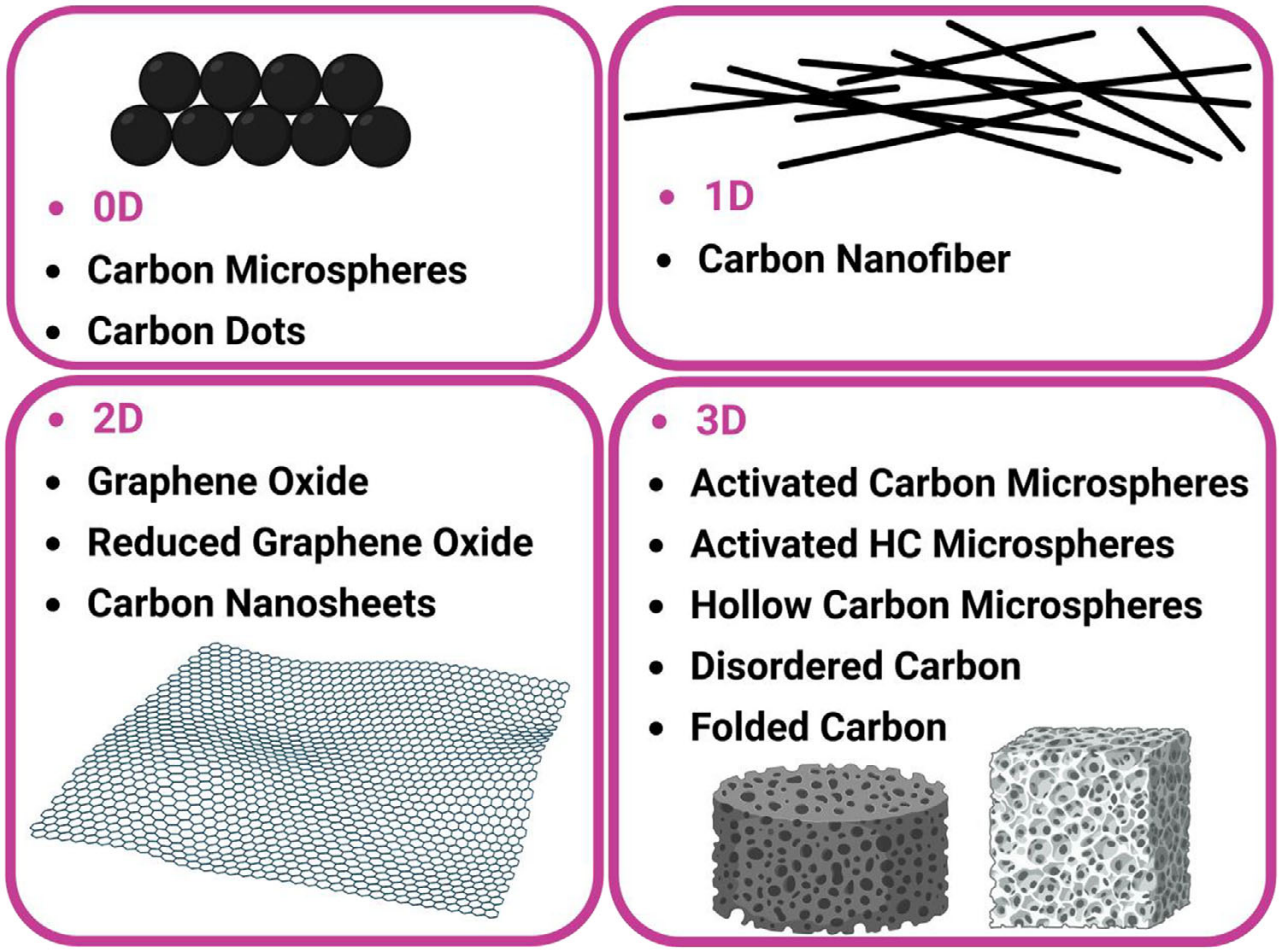
Pictorial diagram showing the different structural morphology of porous carbons. Created by the authors with www.biorender.com.

Porous carbon materials exhibit diverse structural architectures ranging from zero‐dimensional (0D) to three‐dimensional (3D) configurations, encompassing nanoparticles, nanosheets, nanofibers, and interconnected disordered networks synthesized from different carbon sources (Figure 20). This dimensional classification serves as a fundamental framework in materials science for understanding and predicting the physical, chemical, and electronic behaviors of carbon‐based nanomaterials.

Zero‐dimensional (0D) [90], architecture features spherical or quasi‐spherical geometries, exemplified by carbon microspheres and carbon dots. These structures are characterized by their isotropic morphology and high surface‐to‐volume ratios, making them particularly suitable for applications requiring uniform particle distribution and controlled surface chemistry. The preferred synthesis routes for 0D carbon materials include hydrothermal carbonization (HTC) of glucose, sucrose, and other carbohydrate‐rich precursors, where the self‐assembly and nucleation processes under subcritical water conditions naturally favor spherical morphology formation. Additionally, solvothermal methods, microwave‐assisted synthesis, and sol‐gel approaches followed by carbonization are commonly employed to produce carbon dots and microspheres with controlled size distribution.

One‐dimensional (1D) [158] structures display elongated morphologies, primarily represented by carbon nanofibers. The synthesis of 1D carbon structures predominantly relies on electrospinning of polymer solutions (such as polyacrylonitrile, polyvinyl alcohol, or lignin‐based precursors) followed by stabilization and carbonization. This technique enables continuous fiber production with controllable diameter and alignment.

Two‐dimensional (2D) [72, 130] materials present sheet‐like configurations, including graphene oxide, reduced graphene oxide, and carbon nanosheets. These atomically thin structures are distinguished by their remarkably high surface area and superior electrical conductivity. The primary synthesis routes for 2D carbon materials include chemical exfoliation methods, particularly the Hummers method and its modifications for producing graphene oxide from graphite, followed by chemical or thermal reduction to obtain reduced graphene oxide. Additionally, liquid‐phase exfoliation of graphite in organic solvents or aqueous surfactant solutions, CVD on metal substrates (copper or nickel) for high‐quality graphene, and carbonization of layered biomass precursors such as chitosan, alginate, or cellulose nanocrystals under controlled conditions can yield 2D carbon nanosheets. Self‐assembly approaches using amphiphilic molecules or block copolymers as structure‐directing agents also enable the formation of thin carbon sheets.

Three‐dimensional (3D) [55, 89, 152] frameworks comprise intricate bulk architectures with greater structural complexity, such as activated carbon microspheres, activated hard carbon microspheres, hollow carbon microspheres, disordered carbon, and folded carbon networks, rendering them particularly well‐suited for energy storage applications. These materials typically possess high porosity and interconnected pores. The synthesis of 3D carbon architectures employs diverse strategies depending on the desired pore structure and morphology. Direct carbonization of lignocellulosic biomass (wood, coconut shells, bamboo, agricultural residues) followed by physical activation (CO_2_ or steam) or chemical activation (KOH, H_3_PO_4_, ZnCl_2_) is the most widely used approach, producing highly porous activated carbons with tunable pore size distributions, but uncontrolled morphology (random‐like particles). Template‐assisted methods enable precise morphological control: hard templates such as silica nanoparticles and zeolites faithfully replicate the parent template's shape, while MOFs generally preserve their characteristic rhombic or cubic geometries (though this can be lost under certain conditions). Soft templates using surfactant micelles or block copolymers can produce either controlled or random morphologies depending on the synthesis conditions employed. Self‐assembly techniques, freeze‐drying of hydrogels or aerogels, and hydrothermal carbonization combined with activation are also employed to create hierarchical 3D structures. For hollow carbon microspheres, template‐sacrificial methods using polystyrene beads or silica spheres, or self‐templating approaches via Ostwald ripening during hydrothermal synthesis, are preferred routes.

**FIGURE 21:**
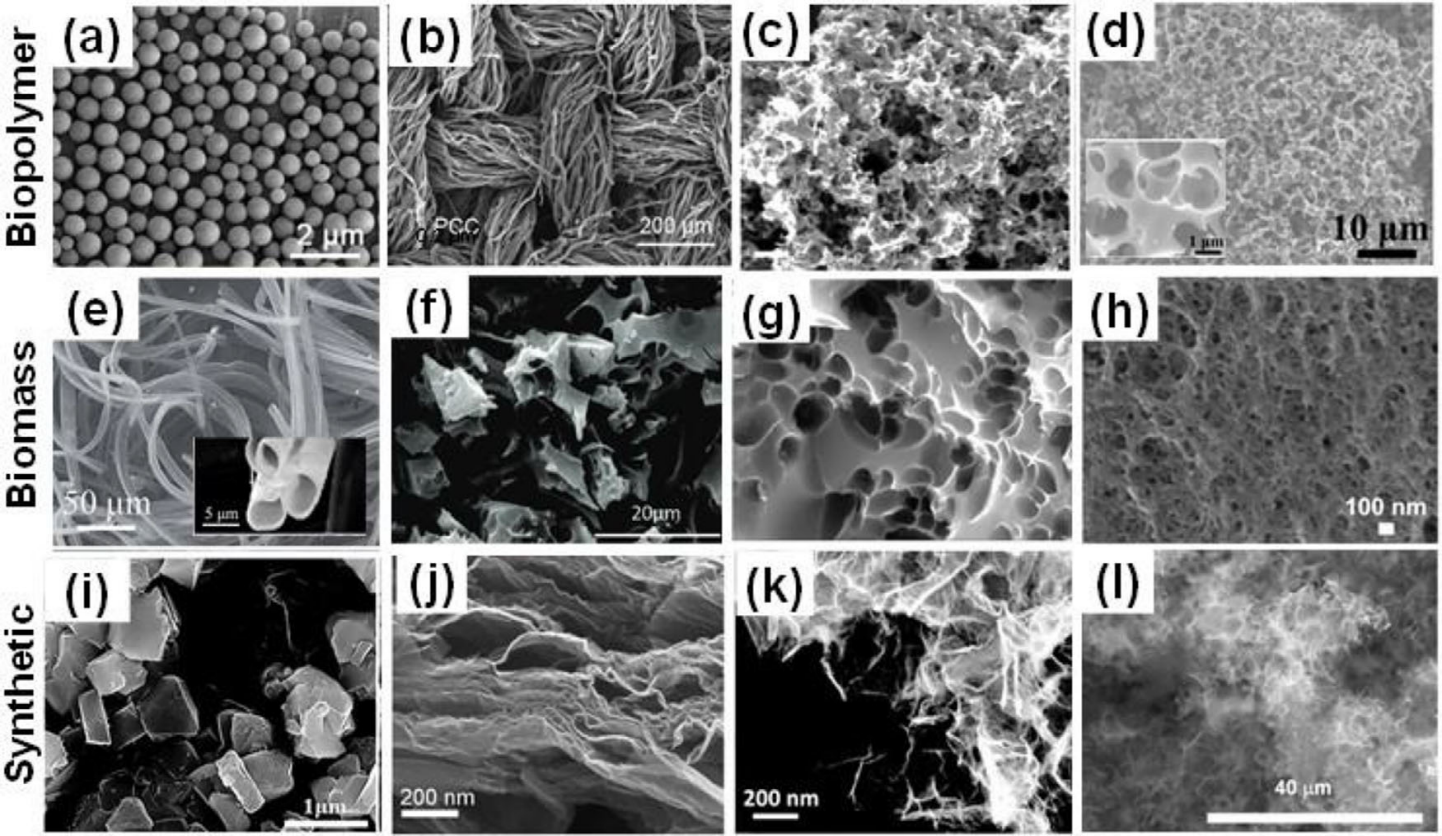
SEM images of porous carbons exhibiting different structural morphologies (a) spherical architecture, Reprinted with permission [90] Copyright (2020), Elsevier. (b) flexible fibres mats, Reprinted with permission [158] Copyright (2024), Royal Society of Chemistry. (c) honeycomb‐like, Reprinted with permission [152] Copyright (2021), Royal Society of Chemistry. (d) 3D interconnected porous framework, Reproduced, [89] Copyright (2022) Springer Nature. (e) hollow tubular, Reprinted with permission [164] Copyright (2024), Royal Society of Chemistry. (f) 3D particulates, Reprinted with permission^[189][1]^ under the terms of CC‐BY license. (g) squishy like, Reprinted, [36] Copyright (2018) American Chemical Society. (h) 3D hierarchical porous structure, Reprinted with permission [162] Copyright (2020), Elsevier. (i) analogous irregular shape, Reprinted with permission [55] Copyright (2021), Royal Society of Chemistry. (j) 2D sheet‐like, Reprinted, [72] Copyright (2023) American Chemical Society. (k) porous nanosheets, Reprinted with permission [130] Copyright (2021), Royal Society of Chemistry. (l) 3D sponge‐like with thin nanosheets, Reprinted with permission [121] Copyright (2023), Royal Society of Chemistry.

Spherical carbons are the most common 0D structures, with their formation heavily influenced by the synthesis method rather than the precursor type. HTC and polymerization reactions are commonly used, utilizing sources like biopolymers (e.g., starch [90]) to control particle shape and size. Key factors such as precursor solution concentration, temperature, and reaction time play a critical role in tailoring the properties of these spherical carbons. Depending on the preparation method, porous carbons from biopolymer‐based precursors can exhibit 0D, 1D, 2D, or 3D architecture. For instance, Zhang and co‐authors [90] prepared porous 0D microspheres with a size distribution between 0.63 ± 0.07 µm (Figure 21a) from starch precursor using HTC (180**°**C for 8–20 h) followed by activation with KHCO_3_ and thermal treatment of the solid mixture at 900°C for 2 h, under N_2_ atmosphere. Also, Wang et al. [158] synthesized porous carbon woven flexible fibres (1D, Figure 21b) from cotton cloth via pre‐carbonization at 400°C under argon flow, for structure stabilization, and subsequent treatment in KOH solution and pyrolysis at 800°C for 4 h under Ar atmosphere. The developed porous carbon cloth exhibited efficient energy storage with a high microporous and self‐supporting structure. Similarly, Cui et al. [152] designed a 3D hierarchical honeycomb‐like porous carbon structure (Figure 21c) from the dissolution of modified Ca(CO_3_)_2_ nanoparticles and glucose in ethanol and distilled water. The precursor after evaporation and drying at 80°C was thermally treated at 1200°C for 3 h.

The authors found that the obtained porous carbon exhibited a high specific surface area (1099 m^2^ g^−1^) with enriched oxygen functionalities and large interlayer spacing (0.386 nm). In addition, the 3D honeycomb structure, which comprises unified carbon sheets, facilitates sodium ion insertion and diffusion in the porous channels. The developed porous carbon showed an enhanced specific capacity of 119 F g^−1^ at 0.1 A g^−1^ when utilized in a symmetrical two‐electrode cell with 1 M NaPF_6_ in diglyme electrolyte. Furthermore, Gao et al. [89] synthesized a 3D interconnected porous carbon framework (Figure 21d) from ground potato starch, mixed with KOH, and pyrolyzed at 800°C for 2h under nitrogen atmosphere. The large number of pores enabled fast ion kinetics in potassium and sodium ion capacitors.

In the case of biomass material precursors, the final porous carbon materials exhibit 3‐dimensional morphology. For example, Zhao and co‐workers [164] obtained activated carbon microtubes (aCMT) with a 3D hollow tubular morphology (Figure 21e) from *Metaplexis japonica* fluff biomass, via heat at 180°C for 2 h in a Teflon‐lined autoclave and then carbonized at 600°C for 2 h under argon inert atmosphere. The aCMT showed a high discharge capacity of 116 mAh g^−1^ at 1 A g^−1^. Xu et al. [163] produced micrometer‐scale 3D particulates (Figure 21f) activated carbon from dried silk worm excrement biomass following a pre‐carbonization at 400°C, KOH activation, and thermal pyrolysis at 800°C, under N_2_ flow. The AC materials obtained possessed a SSA of 1602 m^2^ g^−1^, an interlayer spacing of 0.4 nm, and an intensity ratio of the defective‐band and graphitic‐band (*I*_D_/*I*_G_) of 1.41. The developed AC–AC symmetric hybrid configuration achieved a maximum specific capacitance of 158 F g^−1^ at 0.4 A g^−1^ in 1 M NaClO_4_ in 1:1 EC/DEC electrolyte. Ramakrishnan and co‐workers [36] also produced an activated carbon electrode from goat hair via KOH activation and thermal treatment at 800°C for 2 h under argon flow, giving rise to a highly 3D porous carbon structure with squishy nature (Figure 21g), which is favorable for efficient capacitive ion adsorption/desorption. The developed porous carbon delivered a SSA of 2042 m^2^ g^−1^ and a maximum capacitance of 256 F g^−1^ at 0.02 A g^−1^ in 1 M NaClO_4_ in an EC/DEC electrolyte. Finally, Zhang et al. [162] synthesized a 3D hierarchical porous structure (Figure 21h) from KOH activation of thermally treated soy protein powder in Zn(NO_3_)_2_·6H_2_O solution at 900°C under argon flow. The obtained porous carbon displayed a SSA of 2717.8 m^2^ g^−1^ with a pore size range between 0.5 and 2 nm and 2 and 10 nm, suitable for ion adsorption. It delivered high specific capacitances of 234 F g^−1^ and 158 F g^−1^ at current densities of 0.5 A g^−1^ and 10 A g^−1^, respectively, in 1 M NaClO_4_ in EC:DMC electrolyte at a voltage range of 2.5–4.2 V.

On the other hand, synthetic‐derived porous carbon exhibits a variety of surface morphologies based on 2D and 3D shapes. For example, Li et al. [55] synthesized nitrogen‐doped ordered microporous carbon (N‐OMC) using furfuryl alcohol (FA) and propylene as liquid and gaseous carbon sources, respectively, with commercial NaY zeolite as the hard template. The mixture was heat‐treated at 700°C under a nitrogen atmosphere. The as‐prepared porous carbon exhibited an analogous irregular shape and dimensions (Figure 21i) as a result of the hard‐template, a SSA of 2791 m^2^ g^−1^ and a narrow pore size distribution of 1.27 nm. The N‐OMC delivered a maximum specific capacity of 105.6 mAh g^−1^ at 0.5 A g^−1^ under a voltage window of 2–4.3 V in 1 M NaClO_4_ in EC:DMC with 5 wt.% FEC. Similarly, Zhang et al. [72] manufactured 2D reduced graphene oxide (RGO) from pristine graphite via modified Hummer's method [229] and thermal treatment between 200 and 600°C under N_2_ atmosphere. The developed RGO shows a typical 2D morphology (Figure 21j), having a narrow (002) diffraction peak at 23.2°. The symmetric RGO SIC full cell electrode delivered a maximum specific capacity of 76 mAh g^−1^ at 0.5 A g^−1^, maximum energy density of 92 W h kg^−1^ and a maximum power output of 10896 W kg^−1^ in 1 M NaPF_6_ DME. In addition, Hu et al. [130] prepared a one‐pot Mn single atoms (MnSAs) implanted within N and F co‐doped carbon nanosheets (MnSAs/NF–CNs) by mixing Mn acetate, melamine, and PTFE and thermally treating the obtained mixture under argon at 800°C. The obtained carbon possesses a porous nanosheet structure (Figure 21k), interlayer spacing of 0.41 nm and delivers a reversible specific capacity of 87 mAh g^−1^ at 0.1 A g^−1^, in 1 M NaClO_4_ in EC:DMC electrolyte, under a potential window of 2.5–4.2 V. Finally, Paya and co‐workers [121] synthesized 3D porous carbon from the mixture of potassium chloride, potassium carbonate and gluten then thermally treated at 850°C under N_2_ flow. The developed porous carbon shows a 3D sponge‐like morphology (Figure 21l) characterized by interconnected thin nanosheets due to the templating effect of the micron‐sized KCl and K_2_CO_3_ particles with a BET SSA of 2630 m^2^ g^−1^ and a large pore volume of 1.25 cm^3^ g^−1^. The materials delivered a maximum discharge capacity of 110 mAh g^−1^ under a voltage window of 2–4.2 V in 1 M NaClO_4_ in EC:DEC(1:1) electrolyte. The authors showed that the microstructure of the material can be altered simply due to the templating effect of the micron‐sized KCl salt and K_2_CO_3_ particles.

### 4.2 Porous Carbon Texture

**FIGURE 22:**
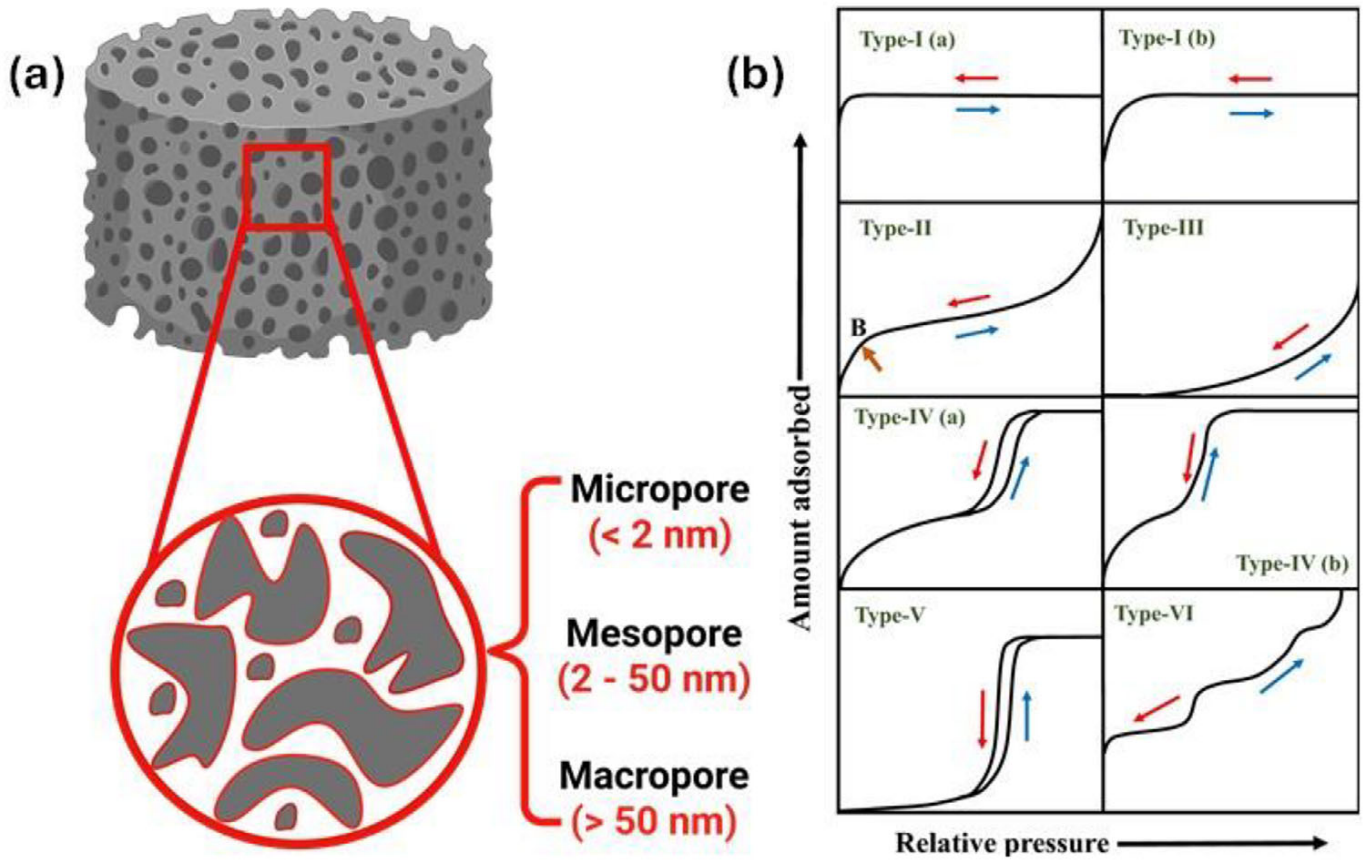
(a) Distribution of pore size in porous carbon, created by the authors with www.biorender.com. (b) Classification of physisorption isotherms. Reprinted with permission [235] Copyright (2023), Springer Nature.

The textural attributes of porous carbon materials, such as the specific surface area, pore volume, and the pore size distribution and design, are essential towards their enhanced performance in capacitive electrochemical storage systems. It has been widely reported that porous carbon with a high specific surface area and high electrode–electrolyte wettability improves the capacitive performance and storage capabilities both in conventional supercapacitors [64, 230, 231] and SIC [23, 51, 173]. Moreover, porosity is a crucial factor in ion diffusion paths as well as the discharge capacity. Thus, the optimization of this parameter is of utmost importance. The pores of porous carbons are mostly divided into three classes, which consist of the micropores having a pore size < 2 nm, mesopores (2–50 nm), and macropores (> 50 nm) (Figure 22a). Micropores contribute to capacitance as they enhance the electric double‐layer formation on the surface of the electrode. On the other hand, both mesopores and macropores enhance the power density of capacitors via the provision of an ion pathway to allow their rapid and efficient movement during fast charge and discharge. However, excessive meso/macropores reduce carbon electrode density, reducing volumetric capacitance (C_vol_). The larger pores absorb excess electrolyte, increasing the overall weight of the supercapacitor device with little contribution to C_vol_ [161, 222, 228].

Adsorption/desorption experiment via a probing gas molecule is the most used method for investigating the pore architecture and for estimating the specific surface area of solid materials. Nitrogen gas at 77 K is the widely used molecule that evaluates the micropores and mesopores and determines the Brunauer–Emmett–Teller (BET) SSA of porous carbons. According to the updated IUPAC technical report on adsorption isotherm classification, Figure 22b illustrates six primary types of adsorption isotherms [236]. Type I isotherms are observed in microporous carbon adsorbents, which possess relatively small external surface areas. These isotherms are reversible and reach a saturation limit at a specific bulk phase pressure, indicating complete filling of the micropores. Type I(a) isotherms are suitable for materials with narrow pore widths (≤1 nm), while Type I(b) isotherms apply to materials with a broader range of pore widths, including narrow mesopores (≤2.5 nm) [236].

**FIGURE 23:**
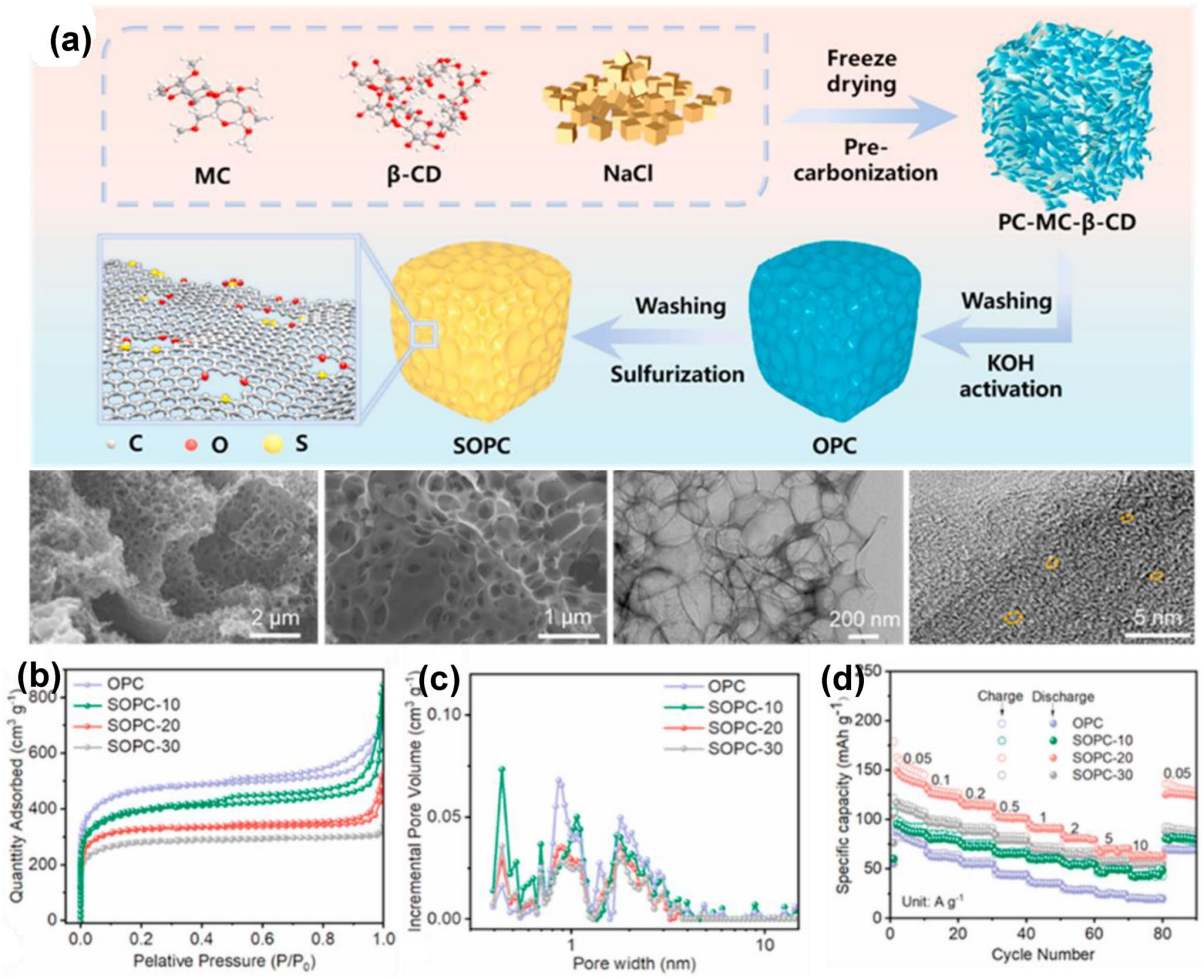
(a) schematic representation of the synthesis of sulfur‐ and oxygen‐doped porous carbon (SOPC) with the SEM and TEM image of the SOPC (b) N_2_ adsorption‐desorption results, (c) pore size distribution, and (d) porous carbon electrochemical performance of SOPC vs. Na metal half‐cell in 0.8 M NaPF_6_ (EC:DEC) electrolyte. Reprinted with permission [87] Copyright (2025), Elsevier.

Type II and Type III isotherms characterize adsorbents with a range of pore sizes, from micropores (≤2 nm) to mesopores (2–50 nm) and macropores (>50 nm). Type IV(a) isotherms display hysteresis linked to capillary condensation, which occurs when pore widths exceed a critical size dependent on the adsorption system and temperature. Type IV(b) isotherms are observed in mesoporous adsorbents with smaller pore widths or in cylindrical/conical mesopores with closed tapered ends. Type V isotherms resemble Type III at lower relative pressures due to weak interactions between the adsorbate and adsorbent [236]. Type VI isotherms describe reversible, stepwise layer‐by‐layer adsorption on highly uniform non‐porous adsorbents. The step height indicates the adsorption capacity of each layer, while the steepness of the curve depends on the system and temperature. Examples include argon or krypton adsorption at low temperatures on graphitized carbon blacks [236].

The development of pore structures in porous carbon is influenced by several key factors, including the type of activation agents (chemical or physical), the ratio of precursor to activation agent, and the activation temperature and time. Chemical activating agents, such as KOH, NaOH, and ZnCl_2_, promote the formation of micropores (<2 nm) and mesopores (2–50 nm) through etching reactions that create highly porous materials with SSA often exceeding 1000 m^2^ g^−1^, and up to 3000 m^2^ g^−1^ in optimized conditions [237]. For example, KOH activation of biomass precursors can yield micropore volumes of 0.5–1.2 cm^3^ g^−1^ and SSA values of 1500–2500 m^2^ g^−1^ [237]. Conversely, physical agents like steam or CO_2_ produce a wider range of pore sizes, from micropores to mesopores and macropores, due to gasification processes that remove carbon atoms, typically resulting in SSA values of 500–1500 m^2^ g^−1^ and pore volumes of 0.3–0.8 cm^3^ g^−1^ [238]. The choice of activation agent directly affects the pore size distribution and SSA, with chemical agents generally achieving higher SSA but requiring careful handling due to their corrosive nature. Additionally, the ratio of precursor (e.g., synthetic polymers, biopolymers, or biomass) to activation agent significantly influences porosity development. For instance, a higher KOH‐to‐precursor ratio (e.g., 4:1) enhances micropore formation but may reduce overall yield, while a lower ratio (e.g., 1:1) balances mesopore development and material capacity retention [237]. Activation temperature (typically 600–900°C) and time further fine‐tune pore size and SSA, with higher temperatures favoring mesopore formation.

Carbonization conditions, such as temperature and duration, play a major role in porosity control. Higher temperature boosts the reaction rate between the precursors and the activating agents, promoting the pore development. During thermal treatment, temperatures between 600 and 900°C are common, with higher temperatures favoring the formation of micropores due to increased carbon consumption [239]. For instance, Casal and co‐workers [171] synthesized porous carbon from ground cork using a mixture of hydroxides (NaOH/KOH). The influence of the temperature (700, 750, and 800°C) was investigated on the mixture under nitrogen flow. The developed porous carbon exhibits a SSA of 2590, 2770, and 2910 m^2^ g^−1^ and a pore volume of 1.17, 1.20, 1.34 cm^3^ g^−1^ at 700, 750, and 800°C, respectively. This highlights the dependency of carbonization on porosity development. However, extremely high temperatures (>800°C) can lead to widening of pores or pore collapse and reduction of specific surface area [59]. The activation time is also important; longer durations allow more extensive reactions, increasing pore volume, but prolonged exposure may cause over‐activation, merging smaller pores into larger ones [239, 240]. For physical activation, temperatures above 800°C are often required to achieve significant gasification, but the process is slower, necessitating longer times. Precise control of temperature and time is crucial to achieve the desired balance between micro/meso/macropore distributions. To achieve this, high‐precision equipment, such as programmable muffle or tube furnaces with controllers, should be used to maintain stable temperatures within ±1°C, while carefully controlling heating rates (e.g., 1–10°C/min) to promote uniform pore development or favor specific pore sizes. For instance, slower heating rates encourage micropore formation, while rapid heating may lead to macropores. Additionally, stepwise heating or isothermal holds at specific temperatures (e.g., 400–600°C for micropores or 800–1000°C for mesopores in carbon materials) and optimized dwell times are critical, with shorter times preserving micropores and longer times enlarging pores. Real‐time monitoring with thermocouples or infrared pyrometers ensures accuracy.

The textural properties of porous carbon are significantly influenced by the type and amount of precursors used. For example, Liu et al. [87] recently obtained sulfur/oxygen co‐doped porous carbon materials (SOPC) from methylcellulose and β‐cyclodextrin with NaCl template and KOH mixture as activating agents for pore formation (Figure 23a). From the nitrogen (77 K) adsorption experiment, the synthesized SOPC showed a hysteresis loop between 0.4 and 0.9 P/P_0_ pressure range, indicative of the presence of mesopores along with micropores (< 0.4 nm). Tuning the sulfur amount allows achieving SSA between 1219 and 1776 m^2^ g^−1^ and a total pore volume of 0.78–1.61 cm^3^ g^−1^ (Figure 23b). The samples showed identical micropore size (0.4–2 nm) with a narrow mesopore size (2–4 nm), Figure 23c. The rise in sulfur doping increased the micropores volume ratio from 58 to 75%, while the average mesopore size decreased from 8.22 nm to 3.40 nm. This highlights that the amount of heteroatoms plays a crucial role in tuning the textural properties of doped porous carbon materials. In terms of the electrochemical performance, the doped SOPCs exhibited specific capacity between 87.4 and 178.9 mAh g^−1^ at 0.05 A g^−1^ in 0.8 M NaPF_6_ (EC:DEC) electrolyte (Figure 23d). The authors attributed the excellent performance to the well‐designed porous structure and surface functionalities.

Numerous studies have focused on the relationship between the specific capacitance and the SSA and the pore size distribution of carbon‐based materials [241, 242, 243]. In many cases, higher SSA correlates with increased capacitance. However, this relationship is often nonlinear with carbon materials with extremely developed SSA, which can be detrimental to the EDLC [241].

The pore size distribution is another critical factor affecting capacitance. Fundamentally, closed pores do not contribute to charge accumulation, and pores smaller than or equal to the ion size typically do not participate in electric double‐layer formation. However, partial ion desolvation may exist under strong polarization [64]. Conflicting findings exist as regards the dependence of capacitance on pore size, particularly for pores around 1 nm. Chmiola et al. [6] and Largeot et al. [244] reported the anomalous increase in normalized capacitance for carbide‐derived carbon in organic electrolytes when accessible pore sizes are below 1 nm. Equally, Raymundo et al. [245] determined that EDL formation is most effective at pore sizes of approximately 0.7 nm in aqueous media, 0.8 nm in organic media, and 1.1 nm in Li‐based electrolytes [246]. On the other hand, Centeno et al. [243] analyzed 28 porous carbons and observed relatively stable capacitance in organic media for pore sizes ranging from 0.7 to 15 nm. Urita et al. [247] also found that hierarchical porous carbon structures with worm‐like surface morphology and pore sizes ranging from that of a solvated ion to a solvent molecule are optimal for EDL capacitors' electrode performance. Thus, the optimization of the global textural properties, such as the SSA and pore volume/pore size distribution, is imperative in the design of a high‐performance positive electrode for SIC.

### 4.3 Porous Carbon Structure, Defects, and Surface Chemistry

#### 4.3.1 Porous Carbon Structure

**FIGURE 24:**
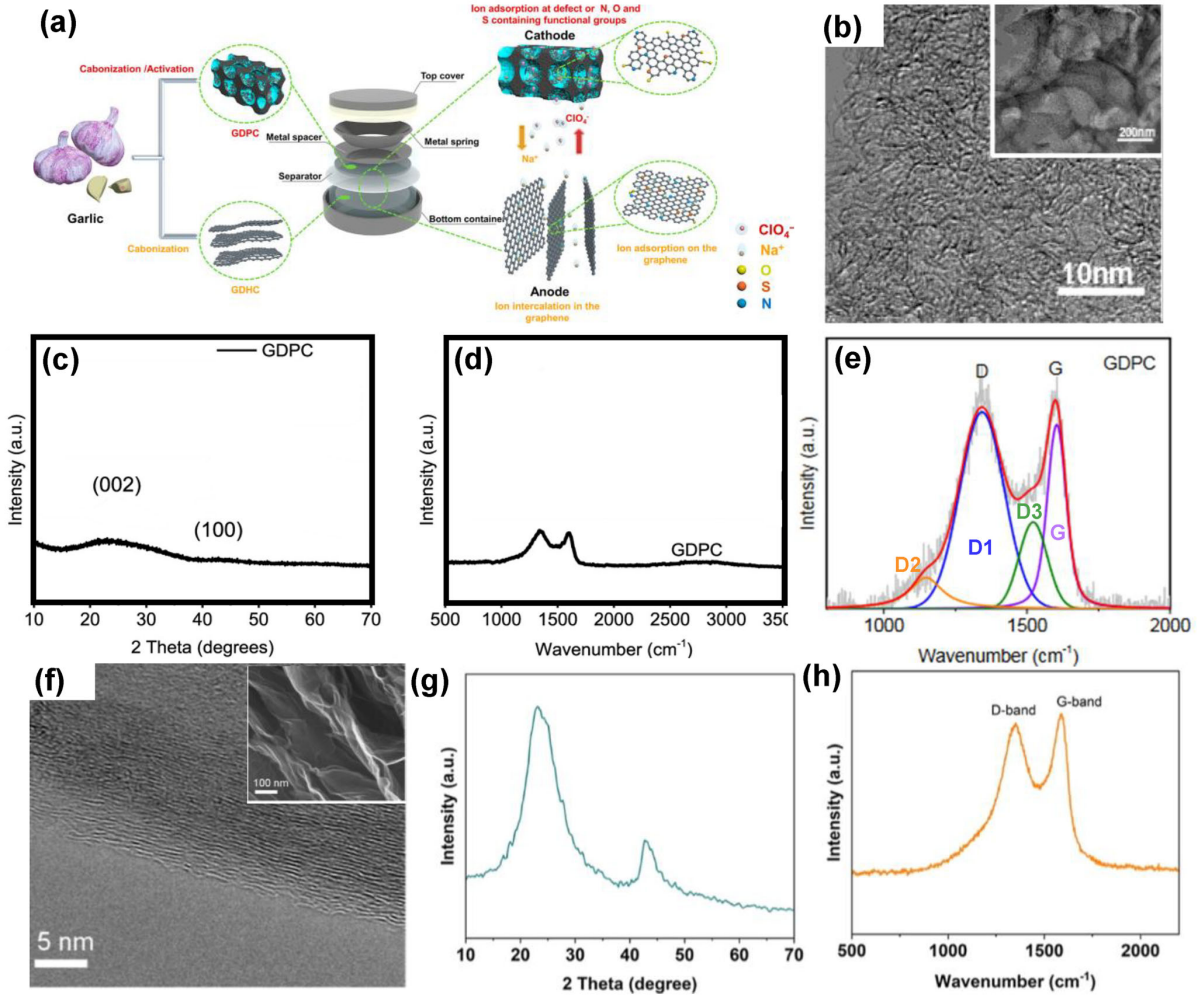
(a) Porous carbon synthesis from the carbonization of garlic, (b)TEM image, (c) XRD pattern, (d) Raman spectrum of GDPC, and (e) four peaks deconvolution of GDPC. Reprinted with permission [47]. Copyright (2019) American Chemical Society. (f) HRTEM and SEM image, (g) XRD pattern, and (h) Raman spectrum of RGO nanosheets. Reprinted, [72] Copyright (2023) American Chemical Society.

The porous carbon structure plays a critical role in the adsorption of electrolytic ions due to its high surface area and tunable pore architecture. X‐ray diffraction (XRD), a non‐destructive technique, is widely used to probe the structural characteristics of PC, providing insights into parameters such as graphitic interlayer spacing (d_002_), crystallinity, and impurities. A larger d_002_ spacing indicates greater disorder or less graphitic character, as defects or amorphous regions increase the interlayer distance. The interlayer spacing of porous carbon is generally higher than that of graphite due to a high degree of disorder, leading to a shift in the (002) plane to a 2θ angle lower than 26°. In PC, XRD patterns often exhibit a broad or nearly flat (002) peak, indicative of a low degree of graphitization and a highly disordered structure. This broadening can obscure precise determination of the d_002_ spacing, complicating quantitative analysis. For example, Liu et al. [47] obtained garlic‐derived porous carbon (GDPC) as a positive electrode for SIC from the traditional KOH activation of peeled garlic biomass (Figure 24a). The obtained GDPC showed abundant porous structure and irregular graphitic sheets (Figure 24b) displaying two broad peaks centered at 24° and 43°, indicating a disordered/amorphous carbon (Figure 24c). Additionally, the (002) peak may show multiple contributions from graphitic and disordered carbon phases, making data interpretation challenging. These complexities highlight the need for complementary techniques to fully elucidate the structural properties of PC.

Raman spectroscopy is one of the most widely used complementary techniques for structural characterization of highly disordered materials such as porous carbon. It is sensitive not only to crystal structures but also to short‐range molecular structures [194]. Raman spectroscopy experiment involves the incident of a laser beam with a specific wavelength that interacts only with the π electrons of sp^2^‐hybridized carbon networks, while providing the information regarding the sp^3^‐hybridized carbon networks [248] indirectly. Raman spectra of disordered carbon show two main primary peaks –disorder‐induced (D) band, found at 1330–1350 cm^−1^, corresponds to ring breathing modes activated primarily in the presence of structural disorder and first‐order graphite (G) band, located at 1580–1590 cm^−1^, arises from in‐plane vibrations of sp^2^‐hybridized carbon atoms (Figure 24d). This D band is directly associated with six‐fold aromatic rings and gains intensity in materials with structural defects [194, 248]. In addition, an overtone of the D‐band observed at 2500–2800 cm^−1^ (2D) is related to ordered graphitic materials [249, 250] and is mostly absent in highly porous carbon materials (Figure 24d). In this regard, a well‐defined D‐band indicates the presence of structural defects, whereas the graphitic domains are linked to an intense G‐band and the presence of the 2D‐band. The 2D band is highly sensitive to the number of graphene layers, stacking order, doping, strain, and electronic structure.

The ratio of the integrated areas or intensities of disordered and ordered bands (*I*_D_/*I*_G_) is widely used to assess the degree of disorder in carbon materials. A higher *I*_D_/*I*_G_ ratio indicates a greater degree of disorder or defects in the carbon structure. It reflects the presence of amorphous carbon, edge defects, or sp^3^‐hybridized carbon compared to sp^2^‐hybridized graphitic carbon [194]. Although performing the integrated area gives more accurate results than taking the intensities of the D and G‐bands [251], to gain deeper insights into the structural properties of these materials, deconvolution of the Raman spectra is often performed. This process allows for a more accurate determination of the integrated areas of the D and G bands compared to simply using their peak intensities. By calculating the integrated areas, a more reliable and quantitative measure of the disorder can be obtained, as it accounts for the full spectral contribution of each band rather than relying solely on peak height [23, 64]. A four peak deconvolution of the D and G bands, as shown in Figure 24e, is attributed to various carbon configurations: a D_1_‐disordered graphitic lattice (A_1g_‐symmetry), a D_2_‐disordered graphitic lattice (C─C/C═C domains) at the edges of graphene layers (A_1g_‐symmetry), D_3_‐amorphous carbon of organic molecules and functional groups, and a G‐ideal graphitic lattice (E_2g_‐symmetry), respectively [194].

Transmission Electron Microscopy (TEM) is another powerful and widely used technique for characterizing the structural properties of porous carbon materials at the nanoscale. By utilizing a high‐energy electron beam that passes through an ultra‐thin sample, TEM generates high‐resolution images that reveal intricate details about the morphology, pore structure, and arrangement of graphene sheets. TEM's ability to achieve resolutions down to the atomic level allows researchers to visualize pore sizes, shapes, and distributions, as well as defects, layer stacking, and crystallinity, which are critical to understanding the material's performance in applications such as energy storage, catalysis, and gas adsorption. In a typical TEM analysis of porous carbon, electrons interact with the sample, producing transmitted and scattered electrons that form images or diffraction patterns. Bright‐field and dark‐field imaging modes provide contrast based on electron scattering, highlighting the porous network and structural features. For instance, micropores (<2 nm), mesopores (2–50 nm), and macropores (>50 nm) can be directly observed, offering insights into the hierarchical pore structure. Additionally, high‐resolution TEM (HRTEM) can reveal the graphitic or amorphous nature of the carbon framework, including lattice fringes and interlayer spacing, which are indicative of the degree of graphitization. This is particularly useful for correlating structural properties with the material's electrical conductivity or mechanical strength.

XPS is another technique used to evaluate the carbon structure that helps in assessing the Csp^2^/Csp^3^ ratio, which quantifies the relative amounts of sp^2^‐hybridized carbon (ordered, graphitic‐like) to sp^3^‐hybridized carbon (disordered, diamond‐like or amorphous). A higher Csp^2^/Csp^3^ ratio indicates a more ordered, graphitic structure, while a lower ratio suggests more defects or amorphous carbon. Recently, Liu and colleagues [252] employed nuclear magnetic resonance (NMR) spectroscopy to examine local structural ordering in carbon materials and its influence on capacitance in organic electrolytes. The authors quantified the local structural disorder of commercial activated carbons by the chemical shift difference (Δ*δ* value). The magnitude of Δδ value is a measurement of the strength of the ring current effect, thus it is a powerful detection of the local structure and “ordered domain size” of nanoporous carbon. The authors observed a positive correlation between the capacitances and the Δδ values, with carbons possessing Δδ values of smaller magnitude showing higher capacitances. This suggests that carbons with smaller ordered domains (smaller Δδ values) give rise to higher capacitances.

In this regard, recent research approach has shown the possibility of having local graphitization in highly porous carbon materials. The presence of local graphitization (such as in salt templated carbons and graphene materials) plays a major role in improving the conductivity of porous carbon materials and the electrochemical performance of supercapacitor electrode in terms of rate capability and cycle stability [231]. For instance, Zhang et al. synthesized reduced graphene oxide from pristine graphite via the modified Hummer's method [229]. The obtained nanosheets showed the typical morphology of 2D structures. HRTEM revealed that as‐prepared RGO nanosheets consist of both disordered and ordered carbon layers (Figure 24f). This is consistent with the presence of a narrow graphite (002) diffraction peak at ~ 24° from the XRD pattern (Figure 24g), indicating that abundant ordered carbon layers still exist alongside disorder domains in the RGO lattice structure following the exfoliation process. The relative intensity of the G‐band is higher than that of the D‐band in the Raman spectrum (Figure 24h), which further confirms the presence of short‐range ordered carbon layers in the RGO lattice structure. The dual carbon symmetric SIC full cell in 1 M NaPF_6_ in dimethyl ether (DME) electrolyte demonstrated specific capacities of 76, 70, 63, 55, and 53 mAh g^−1^ at current densities of 0.5, 1.0, 2.0, 5.0, and 10 A g^−1^, showing good rate capabilities thanks to the presence of the ordered carbon domains.

**FIGURE 25:**
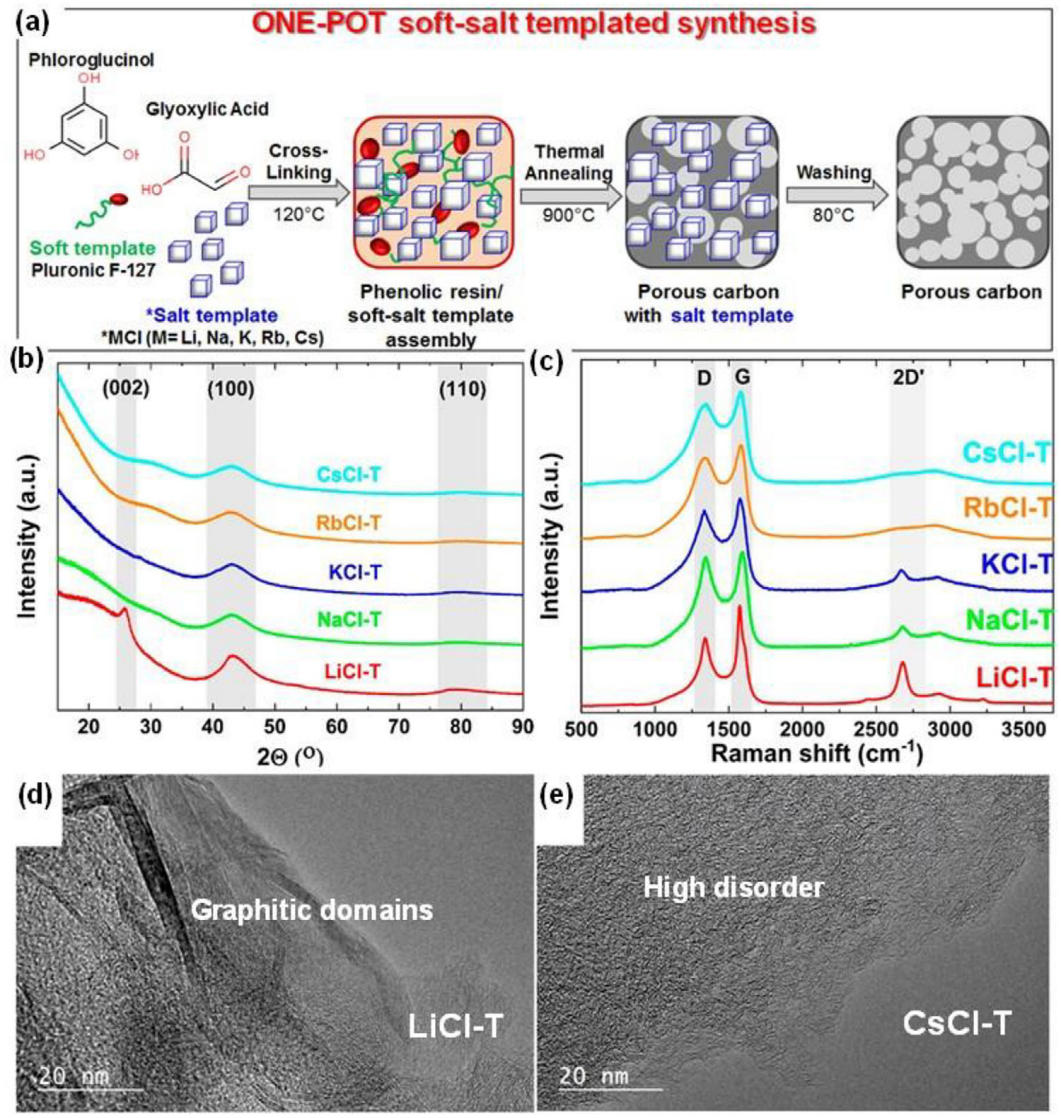
(a) Scheme of combined soft‐salt templated carbon, Structural characterization of the materials: (b) XRD patterns, (c) Raman spectra of templated porous carbons, (d) TEM images of (d) CsCl‐T and (e) LiCl‐T carbons, Reprinted, [231]. Copyright (2021) American Chemical Society.

In the research works of Nita et al. [253], Platek‐Mielczarek et al. [231, 254], Adeniji et al. [23], and Klimek et al. [64], the authors utilized a combined soft‐salt templated (Figure 25a) approach to synthesize porous carbon materials from phenolic resin acting as the carbon precursors, Triblock copolymer Pluronic F127 as the soft template, and a specific type of salt (such as CsCl, RbCl, KCl, NaCl, LiCl, or their mixtures) acting as the salt template. The type of salt utilized determines the nature of the obtained porous carbon in terms of its structural properties. CsCl salt‐based porous carbon exhibited the most disordered, while the carbons based on either KCl, LiCl, or NaCl showed the presence of local structural ordering [23, 64, 231, 253, 254]. The XRD patterns (Figure 25b) of LiCl salt‐based showed distinct diffraction peaks at 2θ values of 26°, 43°, and 78°, corresponding to the (002), (100), and (110) planes of hexagonal graphite, indicating the presence of graphitic domains.

For other materials, the (002) peak, linked to graphene plane stacking, is weak and broad, suggesting disordered carbon structures, consistent with porous carbon materials. The Raman spectrum of LiCl‐T shows a 2D overtone, indicating graphitic domains (Figure 25c,d). NaCl‐T and KCl‐T show disordered structures with some graphitic domains, while RbCl‐T and CsCl‐T lack stacked graphene‐like sheets (Figure 25c,e). The effect of the graphitic domains on electrochemical performance is discussed in Section 5.1.

#### 4.3.2 Porous Carbon Structural Defects

**FIGURE 26:**
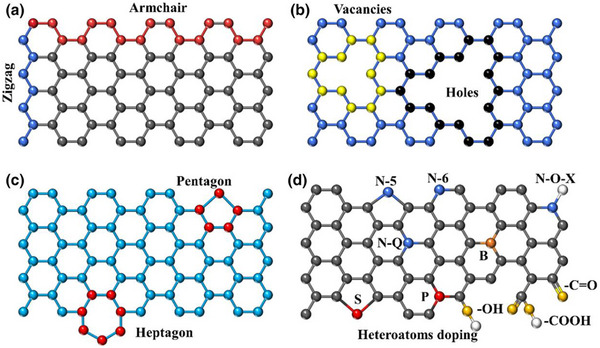
Schematic Figure of (a–c) intrinsic and (d) extrinsic carbon defects. Reprinted with permission [256] Copyright (2024), John Wiley and Sons.

The term “defects” often comes across with a negative meaning, connoting imperfection in materials. With increasing depth of research, scientists have gradually understood how defects can influence the properties of materials. In carbon, they can be classified as intrinsic or extrinsic depending on their origin (Figure 26). Intrinsic defects are made up of sp/sp^3^ hybridized carbon atoms within carbon layers, such as edges (armchair edge and zigzag edge), holes, vacancies, topological defects (pentagons or heptagons), dislocations, and grain boundaries, as shown in Figure 26a–c [255, 256]. These defects act as active sites, boosting electrochemical activity by promoting π‐electron system reactivity. Extrinsic defects include heteroatoms such as boron, oxygen, nitrogen, phosphorus, sulfur, or fluorine incorporated into the carbon matrix, altering the carbonaceous skeleton for improved electrochemical performance, see Figure 26d [257]. In electrochemical capacitors, disorder domains/defects play an important role in the adsorption of ions and enhance the quantum capacitance associated with the electronic density of state (DOS). Carbon defects disturb the arrangement of carbon atoms, altering their band structure and inducing space charge redistribution of carbon atoms surrounding the defect [258].

The presence of defects may introduce new Fermi‐level states, potentially improving the capacitance [259]. The most common characterization techniques for studying the structural defects in porous carbons are Raman spectroscopy and TPD‐MS. Both of these techniques give different but complementary information. Raman spectroscopy provides qualitative information on the internal organization of the carbon and the degree of disorder (with *I*_D_/*I*_G_ ratio), [194, 252] while TPD‐MS provides quantitative information on the total number of active sites (comprising defects) present in the carbon structure by determining the active surface area (ASA) [260]. These techniques uniquely allow for the quantitative evaluation of the basal and edge plan defects such as stacking faults, dislocations, and atom vacancies. For instance, Ding et al. [172] obtained a nanosheet porous carbon with a hierarchically porous architecture from peanut shells with KOH activation. The KOH amount (2× or 3×) and pyrolysis temperature (800 or 850°C) were optimized, giving rise to porous carbon nanosheet with varying *I*_D_/*I*_G_ ratio (2.44 for 800°C and 1.79 for 850°C) obtained from Raman spectra, where the authors highlight that the disorder domains decrease with an increase in the carbonization temperature. Similarly, Li et al. [151] prepared carbonyl‐rich porous carbon materials (CRPC) from disodium roseate salt thermally treated at 700°C under an argon atmosphere and then oxidized at different temperatures. The obtained porous carbon gave a SSA between 783 and 1003 m^2^ g^−1^ with *I*_D_/*I*_G_ values of 4.2, 2.9, 2.5, and 2.3 corresponding to oxidation absence, 390, 420, and 450°C oxidation temperature, respectively. The authors reported that as the oxidation temperature increases, the degree of graphitization in carbon material augments due to the elimination of thermally unstable amorphous carbon on its surface.

The ASA measurement was recently demonstrated for highly porous carbon materials for the quantification of their defects/active sites by TPD‐MS [23, 63, 64], which is associated with chemically accessible and reactive sites that allow covalent bonds. To analyse the ASA, the surface of the PC is first heat‐treated (oxygen‐based functional groups removed under a secondary vacuum), then exposed to oxygen chemisorption (150°C). The newly formed oxygenated surface groups are quantified by TPD‐MS, and the active surface area is thus determined. More precisely, ASA formula considers the amount of CO and CO_2_ desorbed during TPD‐MS (up to 950°C) and the area of an edge carbon site, assuming that: the effective area of a carbon atom edge is 0.083 nm^2^, the edge carbons lie in the (100) plane, and only one bond is formed between oxygen and the edge carbon [23, 63, 260].

Previous studies of ASA measurement were done on graphitic nano‐onions [261] and hard carbon materials [14]. Only recently was the protocol on the measurement of the ASA of porous activated carbon materials implemented [63]. In their pioneering work, Réty et al. [63] measured the ASA of various commercial activated carbons. They found that oxygen chemisorption at 150°C resulted in lower ASA values (9–86 m^2^ g^−1^) compared to 300°C (17–109 m^2^ g^−1^). To prevent sample burn‐off and avoid overestimating ASA, the authors recommended performing oxygen chemisorption at 150°C for activated carbons. They also noted that higher ASA values indicate greater disorder in carbon materials, correlating with enhanced capacitance via pseudo‐capacitance in aqueous electrolytes. Similarly, Adeniji et al. [23] measured the ASA at 150°C of nitrogen‐doped porous carbon (NDPC). The authors highlighted that the graphitic NDPC typically gives rise to low ASA (18 m^2^ g^−1^) values in comparison to highly disordered NDPC (28 m^2^ g^−1^). In the same vein, Klimek et al. [64] studied the ASA of salt‐templated carbons at 150°C. They reported ASA values ranging from 18 m^2^ g^−^^1^ for LiCl/NaCl‐T to 37 m^2^ g^−^^1^ for CsCl‐T. The authors identified a linear relationship between ASA and the *I*_D_/*I*_G_ area ratio, noting that the highest ASA corresponds to the greatest degree of disorder in the salt‐templated carbons.

#### 4.3.3 Porous Carbon Surface Chemistry

One of the most crucial properties of porous carbon is its surface chemistry, as it directly affects its electrochemical performance in terms of its wettability with electrolyte, ion selectivity and interaction, and electrochemical stability [262, 263]. Porous carbon exhibits abundant surface functionalities, which mainly consist of oxygen‐based surface groups such as hydroxyl (C─OH), carboxyl (─COOH), and ether (─C─O─C─). These groups are ever‐present on the surface of porous carbon due to their inherent presence in the precursor used during synthesis and/or due to air exposure. These surface groups are mostly derived from the precursor's composition and/or from external sources utilized in the surface chemistry modification. It is possible to incorporate additional heteroatoms such as N, S, and F into the carbonaceous network.

The characterization techniques used in the evaluation of porous carbon chemistry are X‐ray photoelectron spectroscopy (XPS) and temperature‐programmed desorption coupled with mass spectrometry (TPD‐MS), often complemented by elemental analysis (EA).

**FIGURE 27:**
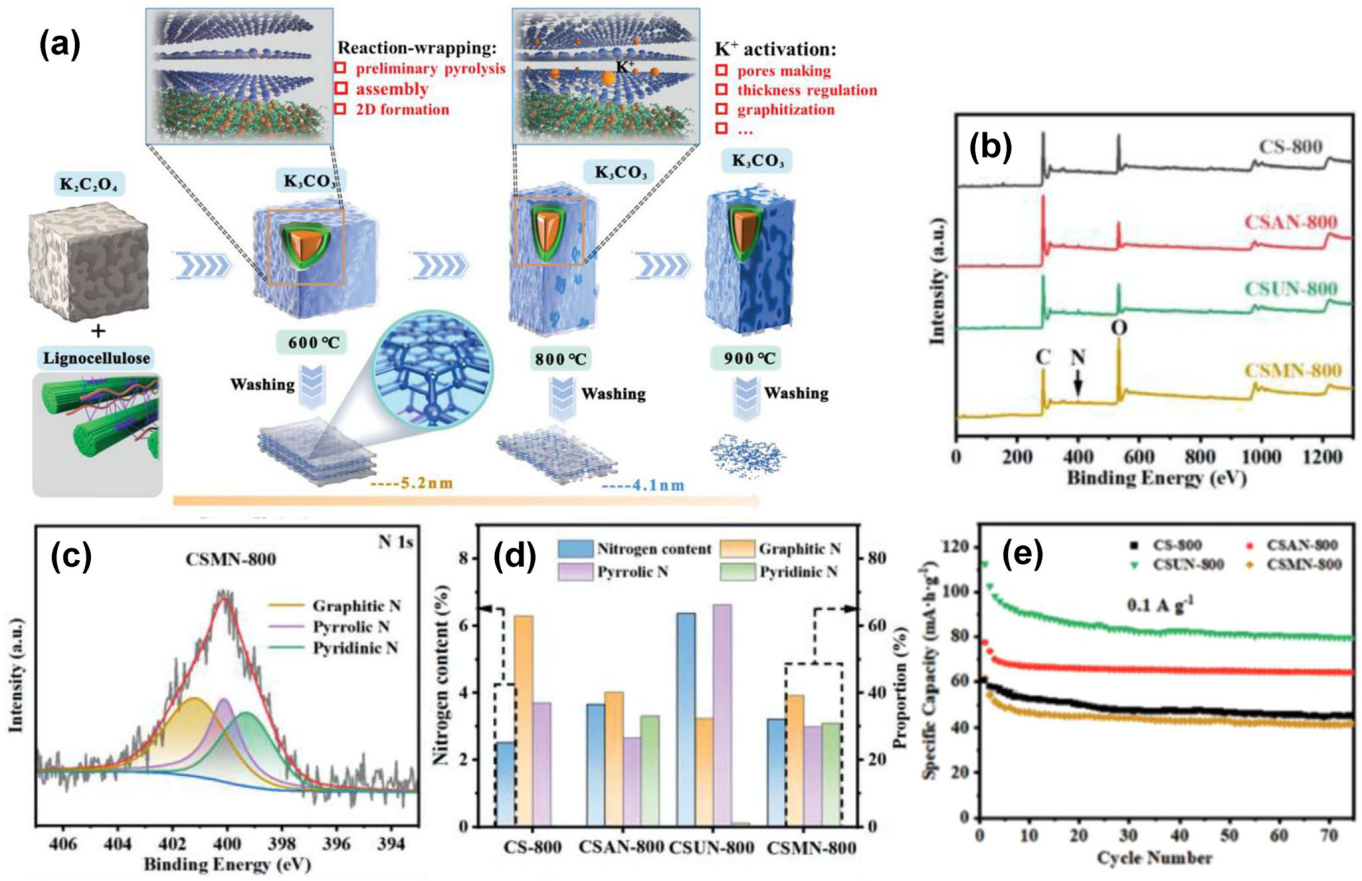
(a) Synthesis of 2D porous carbon by salt templating of cornstalk biomass; (b) XPS survey spectra; (c) N 1s high resolution XPS spectra; (d) Nitrogen content and nitrogen species in cornstalk porous materials, (e) Cycling performances at 0.1 A g^−1^ for the undoped and nitrogen‐doped carbon materials vs. Na metal half‐cell in 1 M NaPF_6_ in DME electrolyte. Reprinted with permission [173] Copyright (2024), John Wiley and Sons.

**FIGURE 28:**
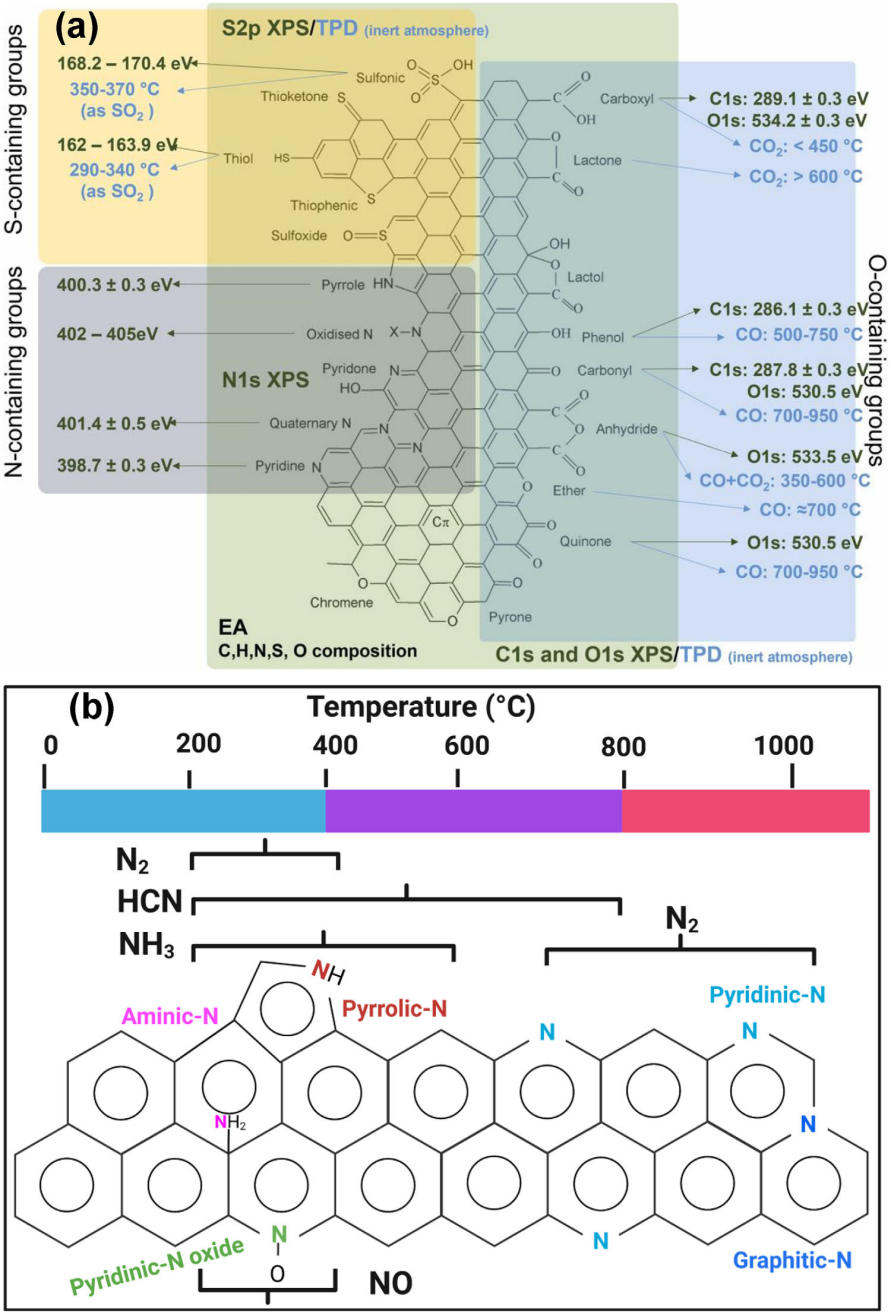
(a) Identification/quantification of O, N, and S surface groups in carbon materials based on XPS and TPD‐MS techniques. Reprinted with permission [264] Copyright (2023), Elsevier. (b) Nitrogen functional groups present on the carbon surface and their thermal decomposition gases by TPD‐MS. Reproduced from [23], the Royal Society of Chemistry.

XPS is a useful technique to investigate the chemical composition of PC surfaces (∼10 nm) and is commonly used to investigate the hybridization state of carbon atoms and oxygen atoms, the presence of impurities, and the chemical bonds. Tables 11, 12, 13 contain the summary of the amount of C, O, and N present on PC surfaces. For instance, in a recent study, Yang and co‐workers [173] directly synthesized ultrathin 2D porous carbon materials from corn stalk, corncob, and soybean straw via co‐pyrolysis with potassium oxalate (Figure 27a). In addition, nitrogen‐doped 2D carbon was also synthesized using urea or melamine as nitrogen sources. From the XPS analysis, the synthesized materials are composed of C, O, and N (2.5–6.4 wt.%). High‐resolution N 1s spectrum of the obtained nitrogen‐doped porous carbon is composed of pyridinic‐N, pyrrolic‐N, and graphitic‐N at 399.3, 400.1, and 401.2 eV, respectively. There are other N‐functionalities that occur at 399 eV, named aminic‐N (Figure 27b–d). In addition, the XPS spectra also contain oxygen functionalities in the form of C═O, C─OH/C─O─C, and ─COOH. Figure 27e shows the cycling performance of the synthesized doped and undoped 2D carbon, showing a capacity of 79.6 and 41.8 mAh g^−1^, respectively, after 70 cycles at 0.1 A g^−1^ in 1 M NaPF_6_ in DME electrolyte. The authors noted that the presence of nitrogen doping helps to effectively improve the specific capacity of 2D carbon materials. Similarly, Liu et al. [157] fabricated hierarchically porous carbons (HPCs) with N/O heteroatoms via the thermal treatment of fish scale biopolymer with KOH, at 800 °C under Ar flow. The developed porous carbon exhibited a honeycomb‐like structure, with rich interconnected macropores and with an extremely high SSA of 3285 m^2^ g^−1^. In terms of surface chemistry, the content of N and O atoms of porous carbon is 2.7 and 7.6 at. %, respectively. The material is rich in both nitrogen (pyridinic (N‐6), pyrrolic/pyridine (N‐5), and quaternary (N‐Q) nitrogen) and oxygen groups (C═O, C─O, ─COOR, and ─COOH). The developed porous carbon delivered a large capacity of 128 mAh g^−1^ at 0.7 A g^−1^, which is still maintained at 72 mAh g^−1^ at 7 A g^−1^ in 1 M NaPF_6_ in DME electrolyte. The authors ascribed the improved performance to redox reactions of the electrolyte with nitrogen/oxygen‐containing functionalities that contribute to pseudocapacitance,

Aside from XPS, TPD‐MS is another complementary technique or advanced tool to probe the nature and quantify the oxygen‐containing functions and the active surface area (ASA) in porous carbons. Briefly, the fundamental principle of the method is to thermally heat the porous carbon materials under a secondary vacuum (or gas flow) and record the spectra of desorbed gases (e.g., CO_2_, CO, H_2_O, H_2_, N_2_, NH_3_, NO, Ar, etc.), as byproducts of the decomposition of functional groups, using quadrupole mass spectrometry [63]. The evaluation of CO and CO_2_ gases is of high importance because they offer valuable information on the different O‐functional groups present, according to the release temperature, as presented in Figure 28a. CO_2_ desorbs at rather low temperatures (<500°C) from various oxygen‐containing functional groups, such as carboxylic acid, anhydride, and lactone groups. Instead, CO is desorbed at higher temperatures (>500°C) from more thermally stable oxygen‐containing functional groups, such as carbonyl, phenol, ether, and quinone [264]. When the two types of groups are summed (i.e., CO + CO_2_), the COx groups can be obtained to provide a global view of the O‐based functionalities of the material. A recent report [23] found positive correlations between total CO, CO_2_, and COx (CO + CO_2_) desorption and specific capacity, with R^2^ values of 0.65, 0.23, and 0.51, respectively. The lower COx correlation suggests interference from certain groups. CO desorption showed a stronger correlation, indicating CO‐based groups (anhydrides, ethers, phenols, quinones, and carbonyls) may boost pseudocapacity, while CO_2_‐based groups (carboxylic acids and anhydrides) might reduce it in 1 M NaPF_6_ in EC:DMC electrolyte.

In the case of the nitrogen functional groups (Figure 28b), the quantification by TPD‐MS is less common and more challenging. Nitrogen oxide (NO) is released at lower temperatures (200–500°C), which is a decomposition product from pyridinic N‐oxide. They can undergo recombination with themselves or other C(N) product groups to give N_2_, [265] which is responsible for the low‐temperature small N_2_ contribution between 200 and 500°C. N_2_ can also be released at higher temperatures (> 600°C), which is related to the removal of temperature‐stable N‐functionalities (pyridinic‐N and graphitic‐N) from the carbon structure, up to 1800°C, [265, 266], where the oxygen functional groups are found to be negligible. Although conventional TPD‐MS is limited to temperatures below 1000°C [63], most nitrogen functional groups in carbon materials desorbed at higher temperatures, up to 1800°C [266].

The NH_3_ contribution arises from the decomposition and reactions of pyrrolic surface groups and/or aminic‐N, [265, 266] leading to higher desorption amounts compared to the NO group. The desorption temperature of NH_3_ for these functional groups occurs between 200 and 500°C for aminic‐N and 500 and 900°C for pyrrolic‐N [265, 266]. The HCN groups are also noteworthy, formed by the surface reaction of C(N) groups with C(H) groups, attributed to the decomposition of pyridinic and pyrrolic groups. Different reports [23, 267] found that nitrogen‐doped carbon materials exhibit enhanced performance due to different nitrogen configurations. Negatively charged pyridine and pyrrole groups act as Faradaic reaction sites, boosting pseudocapacitance. In contrast, positively charged graphitized nitrogen and pyridine‐N‐oxide improve electron transport, enhancing conductivity. The overall capacitance gain depends on factors such as doping level, configuration, and the specific electrode–electrolyte system used.

## 5 Influence of Properties of Porous Carbon on Electrochemical Performance

The electrochemical performance of porous carbon electrodes in SIC heavily relies on their combined textural, structural, morphological, and surface chemical properties of the porous carbon materials. The alteration of such properties could directly impact their performance, in terms of adsorption/desorption of ions, discharge capacity, and long‐term cycling. As SIC technology is still in its early stages, there exists no general protocol, best practices, or standard for the evaluation of porous carbon materials at the laboratory scale, thus making it difficult to compare the experimental results of one article to another. Notwithstanding, some rough trends built by using literature works have been noted between the properties and electrochemical performance in Na metal half cells over a wide range of porous carbon.

### 5.1 Structural Properties

**FIGURE 29:**
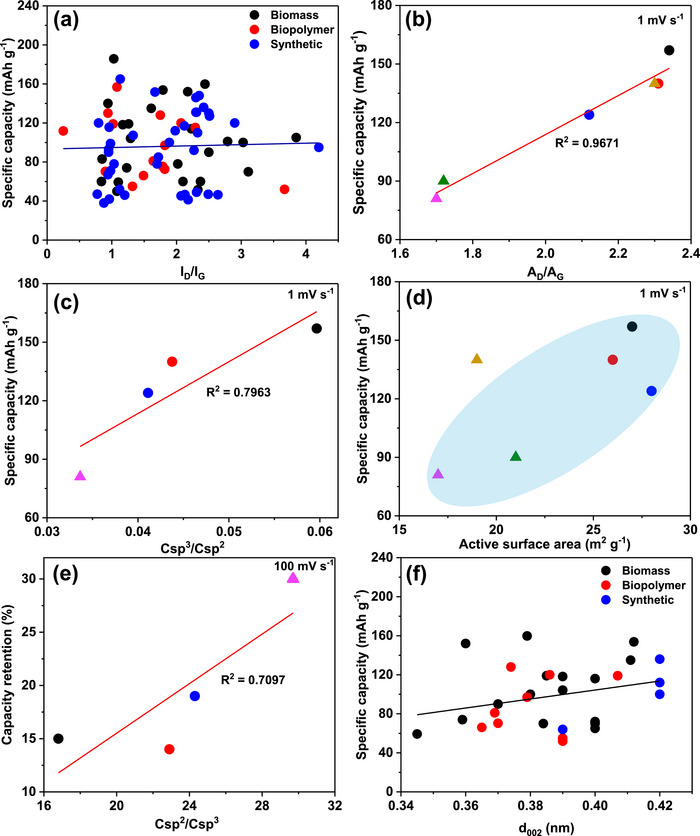
(a) Specific capacity at 0.1 A g^−1^ from Na metal half‐cell as a function of the *I*_D_/*I*_G_ for the three classes of porous carbon precursors. The graphs were built based on the references in Tables 11, 12, 13. Relationship between specific capacity at 1 mV s^−1^ from Na metal half‐cell of nitrogen doped porous carbon (NDPC) and (b) A_D_/A_G_, (c) Csp^3^ /Csp^2^, and (d) ASA. (e) Relationship between capacity retention and Csp^2^/Csp^3^. Reproduced with permission [23], the Royal Society of Chemistry. (f) Specific capacity at 0.1 A g^−1^ from Na metal half‐cell as a function of the interlayer spacing for carbons obtained with the three classes of precursors. The graphs were built based on the references in Tables 11, 12, 13.

The interplay of Raman, XPS, and TPD‐MS offers a comprehensive approach to understanding how the structural properties of porous carbon materials, such as graphitic domains, structural order, and defect density, influence their electrochemical performance. Particularly, the relationship between the specific capacity and the *I*_D_/*I*_G_ ratio, which ranges from 0.25 to 4.2, shows no clear correlation across the three classes of precursors examined (Figure 29a).

This lack of correlation may stem from inconsistencies in calculating the *I*_D_/*I*_G_ ratio, as different studies employ either peak intensity or the area under deconvoluted peaks, leading to variability in specific capacity across the observed range. However, a recent study [23] reported a linear correlation between the specific capacity and the area ratio of the D and G bands (Figure 29b).

In this regard, structural imperfections or defects improve the total capacity (C_EDL_, electric double‐layer capacity + C_Q_, quantum capacity) of the porous carbon positive electrode. Structural defects, including double vacancies, stone‐Wales defects, nitrogen doping, and edge site defects—characterized by increased A_D_/A_G_ ratio, Csp^3^/Csp^2^ ratio (Figure 29c), and of ASA (Figure 29d) with specific capacity—introduce additional electronic states near the Dirac point—the energy level where material's unique electronic properties are defined. This leads to a significant increase in the quantum capacity, thereby enhancing the total capacity. On the other hand, the presence of well‐ordered (graphitic) domains, indicated by a higher Csp^2^/Csp^3^ ratio, contributes to capacity retention (Figure 29e). However, the impact of structural properties on capacity retention is often underexplored in discussions, despite its critical role in electrochemical performance. The balance between defective and graphitic structures influences not only capacity enhancement but also the stability and longevity of the electrode's performance during charge‐discharge cycles.

The interlayer spacing (d_002_) is also an important factor that governs the diffusion of the ions towards the pores. In general, a positive correlation (Figure 29f) was observed between the d_002_ and the specific capacity as the d_002_ increases from 0.34 to 0.42 nm, the specific capacity ranges from 40 to ∼160 mAh g^−1^. A high d_002_ indicates larger spaces to accommodate the electrolyte ions, but also more disordered carbon. Therefore, the observed correlation between the d_002_ and the capacity is in line with the other techniques.

In this regard, Raman spectroscopy, XPS, and TPD‐MS reveal that structural defects in porous carbon materials, indicated by higher *I*_D_/*I*_G_ ratios, higher Csp^3^/Csp^2^ ratios, and increased ASA values, enhance specific capacity by boosting quantum capacity. Larger interlayer spacing also improves ion diffusion into the pores and rate capability. However, more ordered graphitic structures (higher Csp^2^/Csp^3^) improve capacity retention. Balancing disorder and order of porous carbon is key to optimizing electrochemical performance in SIC.

### 5.2 Textural Properties

**FIGURE 30:**
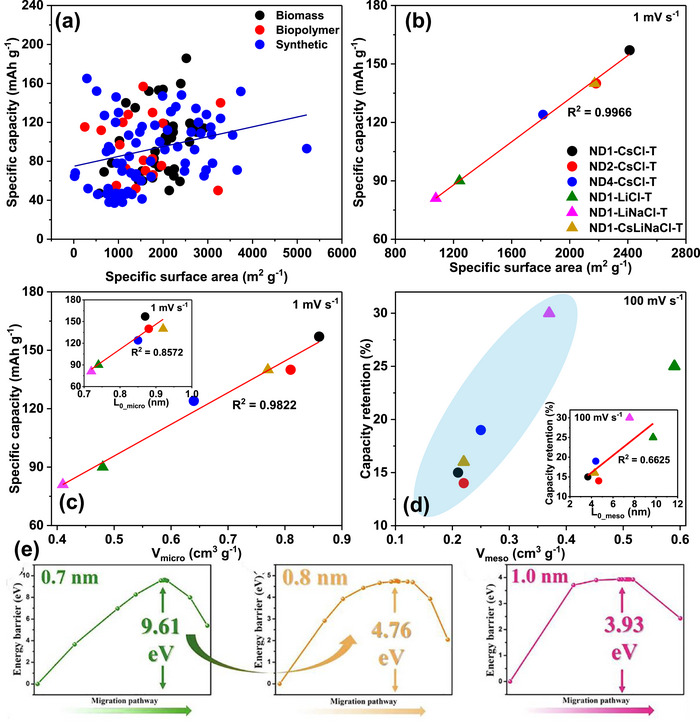
(a) Specific capacity at 0.1 A g^−1^ from Na metal half‐cell as a function of the SSA for the carbon materials obtained with the three classes of precursors. The graphs were built based on the references in Tables 11, 12, 13. Relationship between specific capacity at 1 mV s^−1^ from Na metal half‐cell of nitrogen doped porous carbon (NDPC) and (b) SSA, (c) micropore volume and average micropore diameter (inset). (d) Relationship between capacity retention at 100 mV s^−1^ from Na metal half‐cell and mesopore volume and average mesopore diameter (inset). Reproduced from [23], the Royal Society of Chemistry. (e) Energy barrier of PF_6_^−^ ion adsorption for porous carbon with pore sizes of 0.7, 0.8, and 1.0 nm. Reprinted with permission [270] Copyright (2021), Elsevier.

The relationship between the electrochemical performance of PC and its textural properties, such as SSA and pore size, is intricate and not straightforward. While it is traditionally assumed that higher SSA enhances capacitance, this correlation is not always linear, and excessively high SSA can sometimes diminish performance [243, 268, 269]. As depicted in Figure 30a, the scatter plot illustrates a broad range of SSAs, reaching up to 5214 m^2^ g^−1^ across three material classes: biomass, biopolymer, and synthetic precursors. An upward‐sloping trend line suggests a general positive correlation between specific capacity and SSA, yet significant data scattering reveals that SSA alone does not dictate capacity. It is important to mention that factors such as electrode formulation (some binders block porosity, while SSA in the correlation reflects only the active material and not the entire electrode) and electrochemical conditions (way of calculating capacitance and potential window used) may also influence the specific capacity.

For biomass precursors, SSA ranges from 680 to 2770 m^2^ g^−1^, with specific capacities varying widely from 47 to 186 mAh g^−1^. Biopolymer precursors exhibit SSAs between 248 and 3229 m^2^ g^−1^, with capacities spanning 47 to 157 mAh g^−1^. Synthetic precursors show the widest SSA range, from 35 to 5214 m^2^ g^−1^, and capacities between 37 and 165 mAh g^−1^. This variability indicates that capacity can differ substantially at similar SSA values, regardless of precursor type, highlighting that other factors beyond SSA (such as pore structure or material composition) play a critical role.

Further insight comes from recent research [23], which reported a positive correlation in carefully engineered porous carbon materials derived from polymers between specific capacity and SSA (Figure 30b). This suggests that optimizing the SSA could enhance performance in specific cases, potentially mitigating the limitations observed with high SSA values due to inaccessible pores or inefficient charge storage.

Pore size is a critical factor in porous carbon ion adsorption/desorption SIC. Closed pores and pores smaller than or equal to the ion size do not contribute to charge accumulation on the EDL surface, though partial ion desolvation may occur under strong polarization [64]. Micropores (< 2 nm) significantly contribute to capacitance by enabling EDL on electrode surfaces. Mesopores (2–50 nm) and macropores (> 50 nm), however, enhance power density by providing efficient ion transport pathways during rapid charge‐discharge cycles. Liu et al. [51] demonstrated that the electrosorption behavior of anions, such as ClO_4_^−^, varies with pore size. When pores are smaller than the solvated ClO_4_^−^ ion size (1.48 nm), specific capacitance increases with pore size. Conversely, when pores exceed the solvated ion size, capacitance decreases, as observed in YP‐50F activated carbon (pore size >1.48 nm). The maximum specific capacity is achieved when the pore size closely matches the solvated ClO_4_^−^ ion diameter. Adeniji et al. [23] found a linear correlation between specific capacity and micropore volume/average micropore diameter (Figure 30c and inset), emphasizing the need to optimize pore size with electrolytic ion size (PF_6_^−^ ions in this case).

A minimum pore size threshold of 0.8 nm was found effective for the adsorption of PF_6_^−^ ions, while pores smaller than this threshold create unfavorable thermodynamic conditions. Zou et al. [270] calculated energy barriers for PF_6_^−^ ion adsorption in PC as shown in Figure 30e, revealing that PC‐0.7 (0.7 nm pores) has a high energy barrier (9.61 eV), while PC‐0.8 (0.8 nm) and PC‐1.0 (1.0 nm) have significantly lower energy barriers (4.76 eV and 3.93 eV, respectively). Since the energy barriers for PC‐0.8 and PC‐1.0 are comparable, pores ≥ 0.8 nm are favorable for dynamic PF_6_^−^ ion adsorption.

A linear correlation between mesopore volume/average mesopore size and capacity retention (Figure 30d) indicates that mesopores enhance ion transport, improving rate capability and electrochemical performance. However, high SSA does not guarantee improved capacity retention without the presence of mesopores, and can compromise electronic conductivity by disrupting conductive pathways, limiting rate performance, and power capability [230]. Introducing heteroatoms into the carbon structure is an effective strategy to enhance electronic conductivity and mitigate these limitations. Accelerated aging tests (floating protocol) further reveal that highly microporous, disordered carbon materials with oxygen functionalities degrade faster [271], while more graphitic, less microporous materials exhibit longer floating periods, indicating better durability [64]. While higher SSA can increase specific capacity, excessively high SSA may lead to reduced capacity retention and faster degradation. Pore size optimization is crucial: micropores drive capacitance, while mesopores and macropores support power density through efficient ion transport. Balancing SSA, pore size distribution, and electronic conductivity is essential for designing high‐performance porous carbon materials for SICs.

### 5.3 Surface Chemistry

**FIGURE 31:**
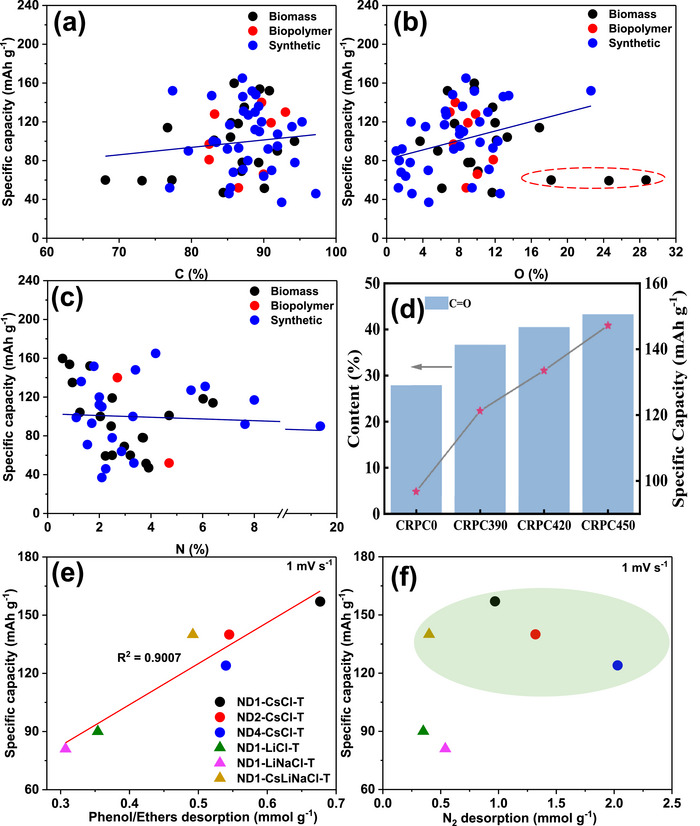
Specific capacity at 0.1 A g^−1^ from Na metal half‐cell as a function of the (a) C (%), (b) N (%), (c) O (%), for the three classes of porous carbon precursors. The graphs were built based on the references in Tables 11, 12, 13. (d) Relationship between C═O content and specific capacity in Na metal half‐cell, Reprinted with permission [151] Copyright (2025), John Wiley and Sons. Relationship between specific capacity at 1 mV s^−1^ from Na metal half‐cell of nitrogen doped porous carbon (NDPC), and (e) phenol/ethers desorbed amount, and (f) N_2_ desorbed amount. Reproduced from [23], the Royal Society of Chemistry.

The surface chemistry plays a significant role in ion storage and performance of PCs in SICs. The surface group is primarily governed by the carbon network itself, which is modified by the presence of heteroatoms such as O, N, and others, which introduce surface functionalities that modify the surface reactivity, conductivity, active sites, and the electrochemical properties. The surface atoms of the PCs were correlated with their specific capacities (Figure 31). Most data points for carbon content (C %) fall between 85%–95%, with a few outliers. The highest‐performing materials have 85–90% C, corresponding to 10–15% O/N content. This suggests a balance between oxygen functionalities, which enhance pseudocapacitance, and carbon, which boosts electronic conductivity. Higher C % likely improves conductivity, but scatter in biomass and biopolymer‐derived carbons (<80% C) indicates that impurities or heteroatoms may impair performance, as shown in Figure 31a.

For oxygen content (Figure 31b), a positive trend is evident, with 8–18% appearing optimal. High oxygen levels (18–29%) correlate with low capacities (∼40 mAh g^−^^1^), likely due to oxygen‐induced defects reducing conductivity. However, some biomass and biopolymer materials with moderate O % (5–15%) achieve higher capacities, suggesting that the type of oxygen functionality is critical. The presence of surface oxygen groups such as phenolic/ether (Figure 31e) has been recently identified to aid pseudocapacitance, while carboxylic acid groups are detrimental [23, 272]. Huo et al. [273] recently highlighted the effect of C─O and C═O functionalities in porous carbon hollow nanospheres/nanosheets (PCHNS) to contribute to extra capacity to the positive electrode through pseudocapacitance reaction. Li and colleagues [151] developed a carbonyl‐rich porous carbon material by reducing unstable oxygen‐containing functional groups (─COOH, ─OH) and oxidizing functional groups (─H, ─OH) on SP_3_‐C at high temperatures to form carbonyl groups. These carbonyl groups are electrochemically active on the carbon positive electrode surface, boosting the electrode's pseudocapacitance capacity as their content increases. Additionally, carbonyl groups serve as dual‐ion active sites for Na^+^ and ClO_4_^−^, enabling pseudocapacitive behavior under varying voltage conditions (Figure 31d). Several other works [51, 109, 274] have also highlighted the role of carbonyl groups in pseudocapacitive reactions for Na‐ion‐based chemistries.

In the case of the nitrogen %, the trend is relatively flat with capacities between 40 and 160 mAh g^−1^ across 0–20% N, as shown in Figure 31c. While nitrogen doping can enhance electrochemical performance, the lack of a clear trend suggests that the type and distribution of nitrogen (e.g., pyridinic, pyrrolic vs. quaternary) are more critical than total content. Biomass and biopolymer carbon materials show a wide range of N%, while synthetic precursors are more controlled. No clear trend was also observed from N_2_ desorption from the TPD‐MS experiment (Figure 31f) [23]. This is due to the limitation of the TPD‐MS as it is limited to <1000°C [63], while most nitrogen functional groups desorbed at higher temperatures, up to 1800°C [266]. However, the XPS experiment identified groups like pyrrolic‐N showing a positive trend with specific capacity, highlighting their impact towards pseudocapacitive contribution [23].

Yang et al. [173] noted that nitrogen doping (6.4 wt. %) can effectively improve the specific capacity of 2D carbon materials, decrease charge transfer resistance while enhancing the electronic conductivity. In fish scale‐derived nitrogen‐doped porous carbon for SIC, Liu et al. [157] reported that the redox reactions between electrolyte ions (Na^+^ and ClO_4_^−^) are as a result of the nitrogen/oxygen‐containing functionalities present on the carbon surface.

**FIGURE 32:**
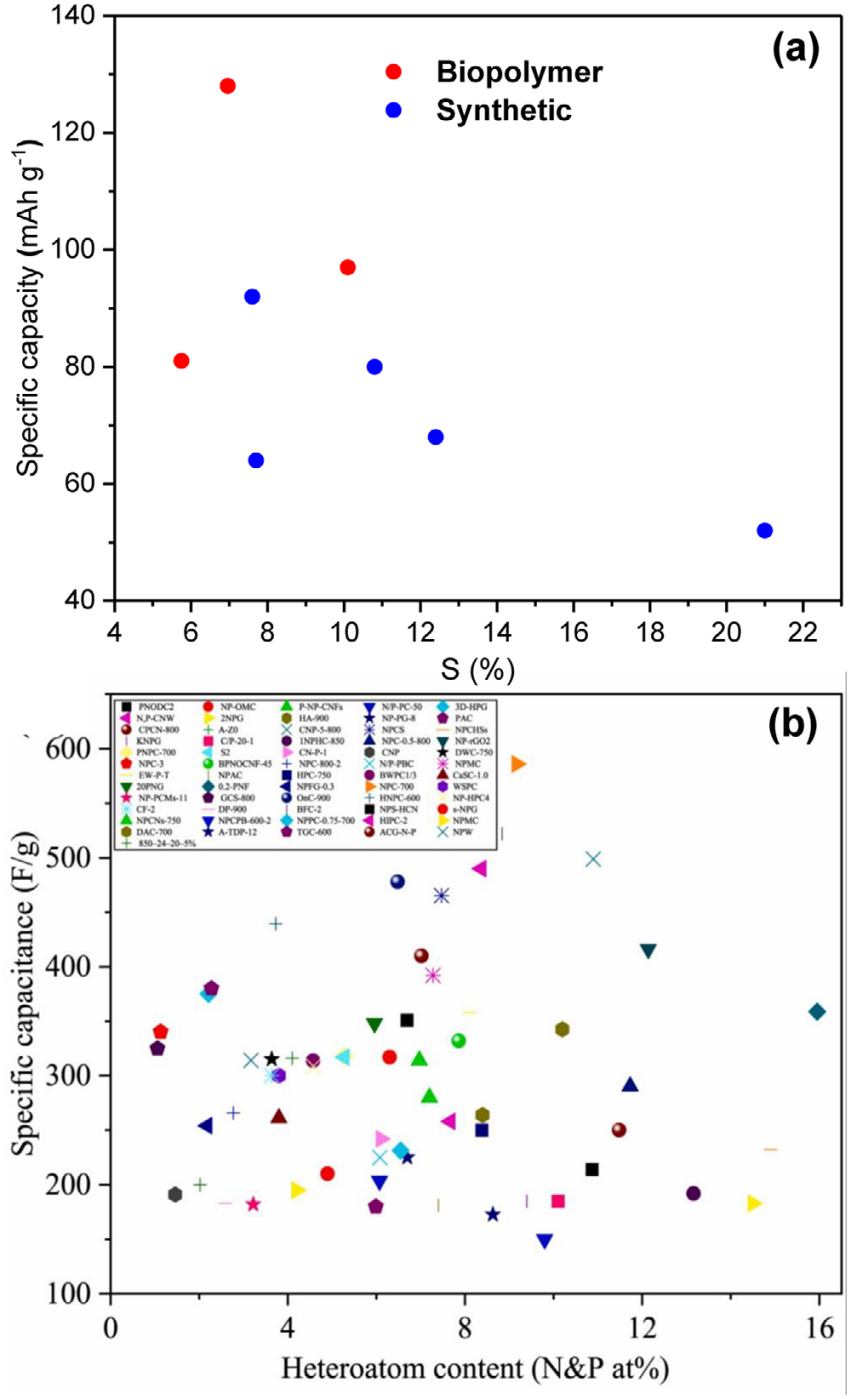
(a) Specific capacity at 0.1 A g^−1^ from Na metal half‐cell as a function of the S (%), The graph is built based on the references in Tables 11, 12, 13. (b) Specific capacitance changes with heteroatom content (N/P) for electrochemical capacitors. Reprinted with permission [275]. Copyright (2024), Elsevier.

Sulfur‐doped porous carbon as a positive electrode material for SICs remains underexplored. Sulfur content typically ranges from ∼5.8 to 10.2 at.%, with optimal doping (<14 at.%) delivering reversible capacities >60 mAh g^−^^1^ (Figure 32a). Excessive S doping, however, reduces conductivity and impairs electrochemical performance. A recent study by Ren et al. [87] shows that moderate S‐doping introduces defects, expands interlayer spacing, and enables faradaic redox reactions, significantly boosting capacity and Na^+^ storage kinetics.

During the charging process, PF_6_^−^ adsorbs on the positive electrode surface/defects; Na^+^ desorbs; reversible reactions C–O–Na → C═O and Na_2_S_x_ → S occur. During the discharging, PF_6_^−^ desorbs; Na^+^ adsorbs and reacts to reform C–O–Na and Na_2_S_x_.

(5) $$Na_{2}S_{x}\overset{\mathrm{charge}}{\underset{\mathrm{discharge}}{⇌}}xS+2Na^{+}+2e^{-}$$

(6) $$C-O-\mathrm{Na}\overset{\mathrm{charge}}{\underset{\mathrm{discharge}}{⇌}}C=O+Na^{+}+e^{-}$$

(7) $$\begin{array}{ccc} \left\{\mathrm{SOPC}-x\right\}∥Na^{+} & + & \mathrm{SOPC}-x+{\mathrm{PF}}_{6}^{-}\overset{\mathrm{charge}}{\underset{\mathrm{discharge}}{⇌}}\mathrm{SOPC}\\ & - & x+Na^{+}+\left\{\mathrm{SOPC}-x\right\}∥{\mathrm{PF}}_{6}^{-} \end{array}$$

Thus, PF_6_^−^ contributes via surface adsorption/desorption, while Na^+^ participates in both adsorption and faradaic redox with C═O and S sites, enabling high energy density and fast kinetics. The Optimized S,O‐dual‐doped porous carbon achieves 135.2 mAh g^−^^1^ at 0.05 A g^−^^1^, enabling SICs with 105.6 Wh kg^−^^1^ energy density (at 714 W kg^−^^1^) and excellent cycling stability (83.5% retention after 4000 cycles at 10 A g^−^^1^).

Phosphorus‐doped porous carbons are rarely explored as positive electrodes for SICs, despite extensive use in conventional supercapacitors. P and N are frequently co‐doped to exploit their synergistic advantages: although both belong to the same group with identical valence electrons, P offers stronger electron‐donating ability, more pronounced n‐type doping, and a larger atomic radius that significantly enlarges carbon interlayer spacing, improves wettability, modulates charge distribution, and narrows the band gap [275]. Co‐doping generates higher defect density and more active sites than single heteroatom doping, markedly boosting electrochemical performance. Optimal total heteroatom content is typically 6–11 at.%, with peak specific capacitance (e.g., 586 F g^−^^1^ at 9.17 at.%) achieved within this range for electrochemical capacitors (Figure 32b). Although N/P co‐doped carbons have delivered outstanding results in lithium‐, potassium‐, and zinc‐ion hybrid capacitors, their application in SICs remains limited. For example, Shan et al. [276] developed an N–S–P–O co‐doped hierarchical fibrous carbon foam from fish bones that, when used in LIC, achieved 160 mAh g^−^^1^ (positive electrode vs. Li/Li^+^^+^), 131 Wh kg^−^^1^ energy density, retained 72 Wh kg^−^^1^ even at 62 kW kg^−^^1^, and maintained 82% capacity after 20 000 cycles, highlighting the potential of multi‐heteroatom co‐doping once systematically applied to SIC positive electrode.

**FIGURE 33:**
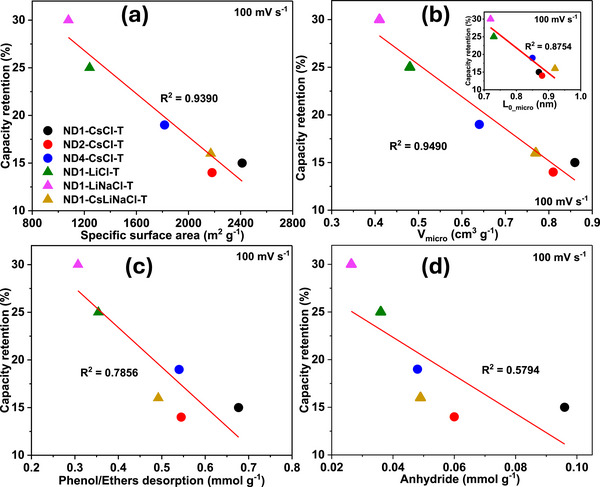
Capacity retention (%) at 100 mV s^−1^ from Na metal half‐cell as a function of the (a) SSA, (b) V_micro_ with L_0_micro_, (c) phenol/ethers desorption, and anhydride desorption (d), for soft‐salt templated carbons. Reproduced with permission [23], the Royal Society of Chemistry.

Another important yet often overlooked factor is the influence of textural properties, specifically SSA (Figure 33a), micropore size and volume (Figure 33b), and surface functional groups (Figure 33c,d) on rate capability and long‐term capacity retention. As shown in Figure 33, all these properties exhibit a negative correlation with capacity retention. This indicates that excessively high SSA, overly narrow micropores, and abundant surface functional groups are detrimental to sustained capacity and rate performance during cycling. Although oxygenated functional groups can provide additional pseudocapacity, they often lead to poor cycling stability over extended periods [23].

Therefore, achieving optimal performance requires a careful balance between textural, structural, and surface chemical properties. An ideal porous carbon material should feature:

✓ moderate specific surface area,
✓ a well‐balanced pore size distribution with both micropores and mesopores
✓ minimal surface functional groups (particularly oxygenated ones).

Such a design enables simultaneously high specific capacity, excellent rate capability, and superior long‐term cycling stability.

## 6 Porous Carbon Electrode Properties

This section examines the characteristics of porous carbon electrodes, focusing on binder selection, electrode mass loading, and electrolyte properties.

### 6.1 Binders

**FIGURE 34:**
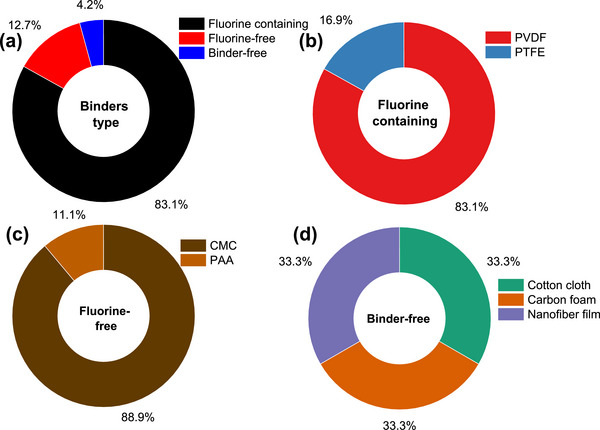
(a) Graphical representation of the various categories of binders utilized in the porous carbon positive electrode preparation. The percentage values are derived from the publications analyzed in Tables 11, 12, 13; Distribution of precursors (%) across three primary categories, along with the binder examples: (b) fluorine‐containing, (c) fluorine‐free, and (d) binder‐free.

Binders are an important component of electrode materials for the efficient performance of the porous carbon positive electrode in SIC. They ensure good adhesion of the active material particles, the conductive carbon (about 5–20% of electrode formulation to enhance conductivity), and the current collector, as well as providing channels for ion conduction. Binder selection can influence the electrode's mechanical strength, stability, ionic and electrical conductivity, and self‐discharge of porous carbon active materials. The binders adopted in porous carbon electrode preparation do not differ from those utilized in conventional batteries (LIB and SIB) and supercapacitors. The most employed binders in porous carbon electrode preparation include thermoplastic fluoropolymers (∼83%), like polyvinylidene fluoride (PVDF) [47, 109, 162] and polytetrafluoroethylene (PTFE) [85, 128, 136], as shown in Figure 34a,b. The type of binder used determines the solvent employed during electrode preparation. For instance, PVDF requires the use of NMP solvent, which has been classified as a carcinogenic, mutagenic, or toxic for reproduction [277, 278]. For PTFE binder, numerous studies [23, 63, 64, 231] have utilized this with solvents such as ethanol and have been processed into self‐standing electrodes.

Recently there has been a transition to fluorine free and aqueous formulated binders (∼13%), such as carboxymethyl cellulose (CMC) [171, 180, 279], PAA (Polyacrylic Acid) [154], [280] PVP (Polyvinylpyrrolidone), [281] and PVB (Polyvinyl Butyral), [282] as a result of the disposing difficulty of fluoropolymers which complicates battery electrode recycling. CMC is the most employed fluorine‐free binder (Figure 34c) and is obtained from natural cellulose alkalization and etherification. On the other hand, binders for porous carbons require careful selection to avoid porosity blockage, which could decrease the specific surface area and affect material electrochemical performance [283, 284, 285]. It is important to match binders and porous carbon, for instance, more hydrophobic porous carbons are suited with hydrophilic binders such as CMC and PVP, while hydrophobic binders such as PVB are better for hydrophilic porous carbons [29, 286, 287]. Notwithstanding, these binders may be insufficient to achieve high mass loading electrodes due to cracks, which may require rubberizing agents such as Styrene‐Butadiene Rubber (SBR) or branched polysaccharides like starch and xanthan gum. Additionally, aqueous binders are complicated when adding pre‐sodiating agents like NaN_3_, NaNO_3_, etc., to porous carbon [29]. Thus, future works on SIC research should also focus on fluorine‐free binders and stable alternatives to ensure the sustainable development of SIC.

With the push for sustainable research and development, fluorine‐based binders are being replaced by eco‐friendly alternatives. Binders are essential for binding carbon particles and securing them to a current collector. However, eliminating binders can enhance electrode accessibility to the electrolyte, improving performance. To tackle this, researchers are investigating binder‐free porous carbon electrodes (Figure 34d), such as PAN nanofiber film [84], carbon filter paper [288], Kynol novolak resin cloth [23, 63, 269], cotton cloth [158], and carbon foam [149]. They account for only ∼4.2% of used electrodes (Figure 34a) due to their difficulty in obtaining, structural integrity, mass loading scalability, and mechanical robustness [289]. Nonetheless, these materials offer diverse textural properties for flexible electrodes, enabling the study of long‐term electrochemical behavior without binder contribution.

### 6.2 Mass Loading

One of the most challenging aspects of electrochemical capacitors is the issue of mass loading, as it refers to the amount of active mass of carbon material per unit area of electrode. In most SIC research reports, porous carbon electrode materials are often tested with low areal mass loading (<5 mg cm^−2^) to show their theoretical performance, which helps maximize the exposition of the active site and shorten ion charge and transport distances. Yet, practical applications require the areal active mass loading in capacitor devices to be as high as possible (>10 mg cm^−2^) for enhanced energy density. Commercial electrochemical capacitors typically employ thicker electrodes between 100 and 200 µm with the active mass materials accounting for at least 30% of the total cell components, corresponding to a mass loading between 10 and 12 mg cm^−2^ [232, 290, 291]. However, high mass loading or electrode thickness reduces rate performance, which leads to decreased active surface area accessibility, higher resistance, and poor electrolyte wetting [232]. For instance, in the study of Liu et al. [51], the influence of the mass loading of N,S co‐doped porous carbon vs. Na metal was studied, and a 20% difference in specific capacity was reported between 1 mg cm^−2^ (100 mAh g^−1^) and 5 mg cm^−2^ (80 mAh g^−1^). The current industrial outlook for SIC projects a cell design featuring a porous carbon positive electrode with a mass loading of 6.9–9.0 mg cm^−2^ and a negative electrode with a mass loading of 2.9–4.5 mg cm^−2^ [292]. Thus, it is imperative to design porous carbon materials with well‐optimized architecture for ion and charge transport with appropriate electrode characteristics, with the aim of bridging the gap between laboratory practices and commercial applications.

The optimization of porous carbon tap density is also critical for achieving high mass loading in PC electrodes, especially for hybrid capacitors, where both gravimetric and volumetric performance are significant considerations. The tap density is described in terms of mass per unit volume that is obtained when voids or air gaps between particles are removed. However, this parameter remains rarely studied in the literature, despite its importance for practical applications. Porous carbons optimized for high SSA (1000 to 3300 m^2^ g^−1^) often exhibit low tap densities (0.27 to 0.56 g cm^−3^) [293], making it challenging to fabricate thick electrodes with sufficient mechanical integrity and electrical conductivity. While high tap density enables higher mass per unit volume and allows electrodes to reach commercial‐level mass loadings, it typically comes at the expense of reduced SSA and, consequently, lower gravimetric capacitance [294]. Therefore, a careful compromise between SSA and tap density must be sought to optimize both volumetric and gravimetric performance.

Zong et al. [295] synthesized a high tap‐density graphene (HTDG) powder of 0.7 g cm^−3^ (SSA: 235.4 m^2^ g^−1^), which is significantly higher than the 0.01 g cm^−3^ of pristine graphene (SSA: 487 m^2^ g^−1^). This nearly 50% reduction in SSA shows the trade‐off inherent in densification strategies. Nevertheless, the high tap density allows for increased mass loading in the electrode up to 13.5 mg cm^−2^, more than doubling that of the pristine sample (4 mg cm^−2^). The enhancement in tap density directly improves the specific capacity (135.4 for HTDG vs. 94.9 mAh g^−1^ for pristine graphene) and efficiency of lithium‐ion capacitors by enabling more active material to be packed into the electrode, elevating the device's overall performance. Songthan et al. [296] in their recent study revealed that the tap density of water filtration‐grade granular activated carbon (WFG‐AC) improves the supercapacitor performance in volume‐constrained designs like 18650 cells. Processing techniques such as ball milling, mechano‐fusion, and annealing raised the tap density from approximately 0.23 (SSA: 2254 m^2^ g^−1^) to 0.63 g cm^−3^ (SSA: 886 m^2^ g^−1^), improving electrode packing and mass loading capabilities. This elevated tap density enabled a high cell capacitance of 105.72 F (7.71 mWh/cm^3^) and remarkable cycling stability with 84.55% retention after 120,000 cycles, despite the moderate specific surface area. This shows that optimizing tap density, rather than solely focusing on the carbon textural properties, is pivotal for achieving high volumetric performance and cost‐effective large‐scale capacitors. Though the optimal balance between tap density and SSA remains application‐dependent and warrants further systematic investigation.

### 6.3 Electrolytes

**FIGURE 35:**
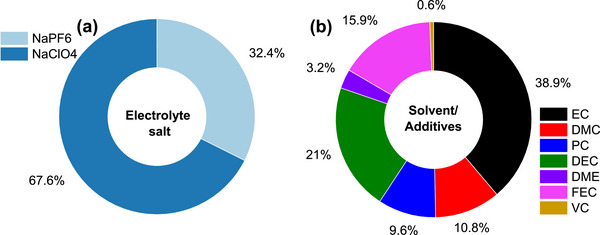
Distribution of different (a) salts and (b) solvent/additives used in electrolyte for SIC application. The percentage values are derived from the publications analyzed in Tables 11, 12, 13.

Typically, SIC electrolytes should exhibit high ionic conductivity, a wide electrochemical potential window, and effective stabilization of the electrode/electrolyte interphase. Many research papers based on porous carbon positive electrode have utilized different types of electrolytes, including organic salts such as NaClO_4_ and NaPF_6_. However, the most used electrolytic salt is the NaClO_4_ (∼68%), thanks to its efficient performance and low cost (Tables 11, 12, 13 and Figure 35a). Notwithstanding, its utilization on a large‐scale application is unlikely due to the highly explosive nature of the dry salts and poses safety risks [29, 297]. Alternative salt like NaPF_6_ (∼32%), which has gained acceptance in SIB, should be considered as the electrolyte standard for SICs. In addition, its usage allows for good performance of SIC and also its ability to better stabilize the SEI. It is also thermally stable and does not form parasitic reaction activities of HF as found in its LiPF_6_ counterpart [298, 299].

**TABLE 11:** Literature review of porous carbon materials derived from synthetic precursors: physico‐chemical and electrochemical performance.

	Synthesis		Surface chemistry			Structure		Porosity	Testing conditions			Performance		Refs.
S/N	Precursors	Synthesis methods	C %	N %	O %	d_002_ (nm)	*I*_D_/*I*_G_	SSA‐N_2_ (m^2^ g^−1^)	Electrode Formulations	Electrolyte	Potential window (V vs. Na/Na^+^)	Current density (A g^−1^)	Specific Capacity (mAh g^−1^)	
1	Sodium ascorbate	Thermal treatment	93.3	n.a.	6.7	n.a.	n.a.	1230	75/10/15, PC/CMC/CB	1 M NaClO_4_ in EC:DMC (1:1) with 5 wt. % FEC	2.5–4.2	0.1	106.8	[122]
2	Metal Azolate Framework (MAF)	Polymerization	86.53	1.71	11.76	n.a.	n.a.	5214	80/10/10, PC/PVDF/CB	1 M NaClO_4_ in EC:DEC (1:1)	2.0–4.2	0.1	93	[80]
			87.13	1.54	11.33	n.a.		3657					71	
			86.24	1.96	11.80	n.a.		2917					65	
3	Potassium citrate	Thermal treatment	n.a.	n.a.	n.a.	0.38	0.94	1398	80/10/10, PC/PVDF/CB	n.a.	2.0–4.2	0.1	67	[75]
4	Diesel soot	Thermal treatment	n.a.	n.a.	n.a.	n.a.	1.035	966	90/10, PC/PVDF	1 M NaPF_6_ in diglyme	1.5–4.0	0.1	78	[123]
5	PVA	Electrospinning	n.a.	n.a.	n.a.	n.a.	n.a.	1506	n.a.	n.a.	2.5–4.2	0.1	60	[124]
6	Melamine‐Phytic‐Aniline	Thermal treatment	n.a.	n.a.	n.a.	n.a.	n.a.	2735	80/10/10, PC/PVDF/CB	1 M NaPF_6_ in EC:DEC (1:1)	1.5–4	0.1	134.2	[125]
7	Magnolol	Polymerization	89.3	1.3	8.4	0.42	2.42	2286	80/10/10, PC/PVDF/CB	1 M NaClO_4_ in EC:DEC (1:1)	1.5–4.2	0.1	136	[51]
			88.8	2.0	8.1	0.42	1.98	2099					112	
			83.0	3.3	12.3	0.42	1.89	1926					100	
8	Furfuryl alcohol	Hard templating	95.3	2.0	2.7	n.a.	0.796	2791	80/10/10, PC/PVDF/CB	1 M NaClO_4_ in EC:PC (1:1) with 5 vol.% FEC	2–4.2	0.1	120	[55]
9	Pyrrole‐Aniline	Hard templating	n.a.	n.a.	n.a.	n.a.	n.a.	3106	80/10/10, PC/PVDF/CB	1 M NaPF_6_ in EC:DEC (1:1)	1.5–4.2	0.1	78	[127]
10	Dolomite	Thermal treatment	83.28	n.a.	16.72	n.a.	0.96	1530	80/10/10, PC/PTFE/CB	1 M NaClO_4_ in EC:DEC (1:1)	2.0–4.2	0.05	115.6	[128]
11	OPDN (4,4′‐(4‐oxophthalazine‐1,3(4H)‐diyl)dibenzonitrile)	Salt templating	87.86	5.55	6.59	n.a.	2.51	687	80/10/10, PC/PVDF/CB	1 M NaClO_4_ in EC:DEC:FEC (1:1:0.05)	1.5–4.2	0.1	127	[131]
			84.98	7.64	7.42	n.a.	2.27	827					92	
12	Magnesium citrate	Thermal treatment	n.a.	n.a.	n.a.	n.a.	2.18	1529	80/10/10, PC/PVDF/CB	1 M NaClO_4_ in EC:DMC (1:1) with 5 vol.% FEC	2.5–4.2	0.1	41.3	[76]
			n.a.	n.a.	n.a.	n.a.	2.31	1210					49	
			n.a.	n.a.	n.a.	n.a.	2.64	1169					46.4	
			n.a.	n.a.	n.a.	n.a.	2.13	745					46.6	
			n.a.	n.a.	n.a.	n.a.	2.07	917					45.3	
			n.a.	n.a.	n.a.	n.a.	2.49	1074					46.9	
13	Resorcinol	Polymerization	n.a.	n.a.	n.a.	n.a.	n.a.	791	80/10/10, PC/PVDF/CB	1 M NaClO_4_ in PC with 5 wt.% FEC	3–4.2	0.1	38	[70]
14	Graphite oxide	Thermal treatment	87.05	4.18	8.77	n.a.	1.13	295	90/10, PC/PVDF	1 M NaPF_6_ in EC:DMC (1:1)	1.5–4.2	0.1	165	[74]
15	Carbon nitride/Urea	Thermal treatment	n.a.	n.a.	n.a.	n.a.	n.a.	2846	75/10/15, PC/PVDF/CB	1 M NaClO_4_ in EC:DMC (1:1) with 5 wt.% FEC	0–3.0	0.1	108.8	[132]
			n.a.	n.a.	n.a.	n.a.	n.a.	2494					78	
			n.a.	n.a.	n.a.	n.a.	n.a.	2479					70	
			n.a.	n.a.	n.a.	n.a.	n.a.	2705					92	
16	Resorcinol	Polymerization	79.58	19.36	1.06	n.a.	0.95	733	80/10/10, PC/PVDF/CB	1 M NaPF_6_ in EC:DEC (4:6)	1.0–4.7	0.5	90	[133]
17	Co(NO_3_)_2_.6H_2_O/2‐ methylimidazole	Thermal treatment	92.48	2.1	4.66	n.a.	n.a.	911	75/10/15, PC/PVDF/CB	1 M NaClO_4_ in EC:DEC (1:1)	2.7–4.2	0.1	37	[135]
18	tetrasodium ethylenediaminetetraacetate tetrahydrate	Thermal treatment	89.4	2.1	8.5	n.a.	2.33	1478	70/15/15, PC/PTFE/CB	1 M NaClO_4_ in EC:EMC:DMC (1:1:1) with 5 wt.% FEC	2.0–4.5	0.1	110	[136]
19	Furfuryl alcohol	Hard templating	n.a.	n.a.	n.a.	0.39	0.88	1090	80/10/10, PC/Teflonized acetylene black/CB	1 M NaClO_4_ in EC:DMC (1:1)	3.0–4.5	0.25	38	[138]
			n.a.	n.a.	n.a.	0.39	0.78	944					47	
			n.a.	n.a.	n.a.	0.39	0.96	979					42	
20	ZIF‐8	Thermal treatment	88.4	1.79	9.71	n.a.	1.67	3738	90/5/5, PC/PTFE/CB	1 M NaClO_4_ in EC:PC (1:1) with 5 wt.% FEC	1.5–4.5	0.1	151.7	[66]
21	d‐(+)‐Gluconic acid δ lactone (GA)	Mechanochemical	n.a.	n.a.	n.a.	n.a.	n.a.	2325	n.a.	1 M NaClO_4_ in EC:DEC (1:1)	2.0–4.2	0.1	95	[141]
22	NH_2_ ‐MIL‐125 (Ti)	HTC	n.a.	2.86	n.a.	0.39	n.a.	1731	75/10/15, PC/PVDF/CB	1 M NaClO_4_ in EC:DEC (1:1) with 5 wt.% FEC	2.0–4.2	0.2	64	[142]
23	Polypyrrole	Solution synthesis	n.a.	n.a.	n.a.	n.a.	n.a.	2970	80/10/10, PC/CMC/CB	1 M NaClO_4_ in EC:DEC (1:1)	2.0–4	0.1	128	[48]
24	Graphene oxide (GO)	Sonication	85.39	3.34	9.45	n.a.	1.12	320	75/15/10, PC/PVDF/CB	1 M NaClO_4_ in EC:DEC (1:1)	2.5–4.2	0.2	52	[143]
25	Glyoxal/Urea	Polymerization	n.a.	n.a.	n.a.	n.a.	n.a.	2961	80/10/10, PC/ PVDF/CB	1 M NaClO_4_ in EC:DEC (1:1) with 2 vol.% FEC	2.0–4.0	0.1	71	[144]
26	MOF	Polymerization	n.a.	n.a.	n.a.	n.a.	n.a.	3285	80/10/10, PC/ PTFE/CB	1 M NaClO_4_ in EC:DMC:EMC (1:1) with 5% FEC	2.0–4.2	0.1	124	[78, 79]
27	Polyaniline	Polymerization	n.a.	n.a.	n.a.	n.a.	n.a.	35	80/10/10, PC/Teflonized acetylene black/CB	1 M NaClO_4_ in EC:DMC (1:1)	3.0–4.5	0.25	68	[68]
28	Polyimide	Polymerization/HTC	85.2	2.25	12.55	n.a.	1.2	1302	60/10/30, PC/PTFE/CB	1 M NaClO_4_ in EC:DEC (1:1) with 0.3 wt.% FEC	1.5–4.2	0.1	46	[145]
29	Graphite	HTC	n.a.	n.a.	n.a.	n.a.	n.a.	19.7	80/10/10, PC/ PVDF/CB	1 M NaPF_6_ in EC:DMC (6:4)	2.5–4.5	0.5	64.6	[71]
30	Lignite	Thermal treatment	91.92	n.a.	8.08	n.a.	1.33	3084	80/10/10, PC/PTFE/CB	1 M NaClO_4_ in EC:DEC (1:1)	2.0–4.2	0.2	107.25	[148]
31	Tannic acid	Salt templating	77.0	21.4 (S wt.%)	1.3	n.a.	n.a.	770	80/10/10, PC/CMC/CB	1 M NaClO_4_ in EC:DEC (1:1)	2.0–4.2	0.1	52	[52]
			85.8	12.4 (S wt.%)	1.6	n.a.	n.a.	1280					68	
			90.0	7.7 (S wt.%)	2.1	n.a.	n.a.	1380					64	
			90.6	7.6 (S wt.%)	1.7	n.a.	n.a.	2040					92	
			87.8	10.8 (S wt.%)	1.4	n.a.	n.a.	1720					80	
32	Maleic acid	Self templating	82.8	n.a.	13.5	n.a.	n.a.	1988	80/10/10, PC/PTFE/CB	1 M NaPF_6_ in VC:DMC (50:50)	1.5–4.2	0.1	147	[54]
			93.9	n.a.	4.3	n.a.	n.a.	3066					115	
33	Carbon foam	Chemical oxidation	77.4	n.a.	22.6	n.a.	n.a.	513	Free standing	1 M NaPF_6_ in EC:DMC (3:7)	1.5–4.2	0.1	152	[149]
			91.1	n.a.	4.6	n.a.	n.a.	1082					70	
			97.2	n.a.	2.8	n.a.	n.a.	537					46	
34	Nitrilotriacetic acid trisodium	Thermal treatment	n.a.	n.a.	n.a.	n.a.	n.a.	3556	80/10/10, PC/ PVDF/CB	1 M NaPF_6_ in EC:DEC (1:1) 5 wt.% FEC	2.0–4.2	0.5	106.9	[150]
35	Phloroglucinol/Glyoxylic acid/Guanine	Polymerization/Salt templating	88.9	3.4	7.3	n.a.	2.35	2412	80/10/10, PC/PTFE/CB	1 M NaPF_6_ in EC:DMC (1:1)	1.5–4.5	0.1	148	[23]
			87.2	6.1	6.5	n.a.	2.31	2181					131	
			85.3	8	6.4	n.a.	2.12	1816					117	
			n.a.	n.a.	n.a.	n.a.	1.72	1241					85	
			94.3	2.5	2.6	n.a.	1.7	1078					78	
			n.a.	n.a.	n.a.	n.a.	2.30	2171					131	
36	Disodium roseate salt	Salt templating	91.9	n.a.	8.1	n.a.	4.2	783	80/10/10, PC/ PVDF/CB	1 M NaClO_4_ in EC:DMC (1:1) with 5 wt. % FEC	1.5–4.2		95	[151]
			89.7	n.a.	10.3	n.a.	2.9	937					120	
			88.8	n.a.	11.2	n.a.	2.5	1003					130	
			87.1	n.a.	12.9	n.a.	2.3	949					146	
37	Anthracite coal	Thermal treatment	83.45	1.11	10.33	n.a.	0.98	1394	80/10/10, PC/ PVDF/CB	1 M NaPF_6_ in EC:DEC (1:1) with 5 wt.% FEC	1.5–4.0	0.1	98.9	[88]

n.a, (not applicable)

**TABLE 12:** Literature review of porous carbon materials derived from biopolymer precursors: physico‐chemical and electrochemical performance.

S/N	Synthesis		Surface chemistry			Structure		Porosity	Testing conditions				Performance	Refs.
	Precursors	Synthesis methods	C %	N %	O %	d_002_ (nm)	*I*_D_/*I*_G_	SSA‐N_2_ (m^2^ g^−1^)	Electrode Formulations	Electrolyte	Potential window (V vs. Na/Na^+^)	Current density (A g^−1^)	Specific Capacity (mAh g^−1^)	
1	Gluten	Salt templating	91.8	n.a.	3.6	n.a.	n.a.	2630	80/10/10, PC/CMC/CB	1 M NaClO_4_ in EC:DEC (1:1)	2.0–4.2	0.1	110	[121]
2	Lignin/PAN	Electrospinning	86.5	4.7	8.8	0.39	3.67	1390	Binder free	1 M NaClO_4_ in EC:PC (1:1) with 5 wt. % FEC	2.0–4.0	0.1	52	[84]
3	Potato starch residue	Thermal stabilization	n.a.	n.a.	n.a.	0.39	1.32	954	80/10/10, PC/ PVDF/CB	1 M NaPF_6_ in EC:DMC (1:1)	2.0–4.0	0.2	55	[89]
4	Glucose	HTC	n.a.	n.a.	n.a.	0.386	2.07	1099	80/10/10, PC/PVDF/CB	1 M NaPF_6_ in Diglyme	1.0–3.7	0.1	120	[152]
5	Bacterial cellulose	HTC	n.a.	n.a.	n.a.	n.a.	1.786	1963	80/10/10, PC/PVDF/CB	1 M NaClO_4_ in EC:PC (1:1) with 5 wt. % FEC	2.0–4.2	0.1	75.48	[153]
			n.a.	n.a.	n.a.	n.a.	1.82	1163					72.65	
6	Sucrose	Hard templating	n.a.	n.a.	n.a.	n.a.	n.a.	1762	80/5/5/10, PC/CMC/ PAA/CB	1 M NaClO_4_ in EC:PC:FEC (45:45:10 wt%)	3.2–4.4	0.1	82.5	[154]
7	Honey	HTC	n.a.	n.a.	n.a.	n.a.	1.08	1554	80/10/10, PC/PVDF/CB	0.75 M NaPF_6_ in EC:DEC (1:1)	1.5–4.3	0.1	156.8	[156]
8	Bacterial cellulose	HTC	n.a.	n.a.	n.a.	0.37	0.9	1606	80/10/10, PC/PTFE/CB	1 M NaClO_4_ in EC:PC (1:1)	2.0–4.2	0.1	70.28	[85]
9	Fish scale	Thermal treatment	89.7	2.7	7.6	n.a.	n.a.	3285	80/10/10, PC/PVDF/CB	1 M NaClO_4_ in EC:PC:FEC (1:1:0.05)	1.5–4.2	0.35	140	[157]
10	Cellulose	Hydrothermal	93	n.a.	7	n.a.	0.94	1764	80/10/10, PC/PVDF/CB	1 M NaPF_6_ in EC:PC (1:1)	1.5–4.5	0.1	130	[86]
11	Cotton cloth	Thermal treatment	91.01	n.a.	8.99	0.407	1.02	1995	Binder‐free	1 M NaPF_6_ (DME)	1.0–4.0	0.1	119	[158]
12	Methyl cellulose/ β‐cyclodextrin	Salt templating/ HTC	89.97	0 (S%)	10.03	0.365	1.49	1776	80/10/10, PC/CMC/CB	0.8 M NaPF_6_ in EC:DEC (1:1)	1.2–4.2	0.1	66	[87]
			82.45	5.75(S%)	11.8	0.369	1.64	1561					81	
			83.2	6.96(S%)	9.84	0.374	1.75	1219					128	
			82.46	10.15(S%)	7.39	0.379	1.82	1031					97	
13	Bacterial cellulose	HTC	n.a.	n.a.	n.a.	0.40	2.29	248	80/10/10, PC/PVDF/CB	1 M NaClO_4_ in EC: PC (1:1)	2.0–4.2	0.1	115.3	[160]

n.a, (not applicable)

**TABLE 13:** Literature review of porous carbon materials derived from biomass precursors: physico‐chemical and electrochemical performance.

	Synthesis		Surface chemistry			Structure		Porosity	Testing conditions				Performance	Refs.
S/N	Precursors	Synthesis methods	C %	N %	O %	d_002_ (nm)	*I*_D_/*I*_G_	SSA‐N_2_ (m^2^ g^−1^)	Electrode Formulations	Electrolyte	Potential window (V vs. Na/Na^+^)	Current density (A g^−1^)	Specific Capacity (mAh g^−1^)	
1	Abandoned bamboo	NH_3_ plasma treatment/ Thermal treatment	83.15	4.7	12.15	n.a.	2.79	1022	80/10/10, PC/ PVDF/CB	1 M NaClO_4_ (n.a)	2.0–4.5	0.1	101	[109]
			84.41	3.91	11.68	n.a.	n.a.	577					47.1	
			86.93	2.97	10.1	n.a.	n.a.	680					69.1	
			87.03	3.67	9.3	n.a.	n.a.	850					78.2	
			90.08	3.81	6.11	n.a.	2.33	898					51.5	
2	Soy protein powder	Thermal treatment	n.a.	n.a.	n.a.	n.a.	n.a.	2717	80/10/10, PC/ PVDF/CB	1 M NaClO_4_ in EC:DMC (1:1) with 5 wt. % FEC	2.5–4.2	0.5	110.5	[162]
3	Garlic	Thermal treatment	90.78	1.64	6.74	0.36	2.17	1682	75/10/15, PC/PVDF/CB	1 M NaClO_4_ in EC:DEC (1:1) with 5 wt. % FEC	1.5–4.2	0.05	152	[47]
4	Sugarcane bagasse	Aq. Mixing/ Thermal treatment	n.a.	n.a.	n.a.	n.a.	n.a.	1762	70/10/20. PC/PVDF/CB	1 M NaClO_4_ in PC with 5% FEC	0–4.0	0.1	63	[165]
5	Jasmine rice	HTC	73.17	2.24	24.59	0.345	1.1	2377	80/10/10, PC/ PVDF/CB	1 M NaPF_6_ in EC:DEC (1:1)	2.0–4.0	0.1	59.3	[167]
6	Goat hair	HTC	86.47	6.02	7.51	0.39	1.17	2042	80/10/10, PC/ PVDF/CB	0.75 M NaPF_6_ in EC:DEC (1:1)	1.5–4.2	0.2	118.2	[36]
7	Olive pit	Thermal treatment	n.a.	n.a.	n.a.	n.a.	n.a.	2225	95/5, PC/PVDF	1 M NaPF_6_ in EC:PC (1:1)	1.5–4.2	0.1	110	[33]
8	Cinnamon sticks	Thermal treatment	n.a.	n.a.	n.a.	n.a.	0.84	1540	80/10/10, PC/teflonized acetylene black/CB	1 M NaClO_4_ in EC:DMC (1:1)	3.0–4.6	0.4	60	[112]
9	Peanut skin	Salt templating	91.88	2.46	5.66	0.37	2.5	1821	80/10/10, PC/ PVDF/CB	1 M NaClO_4_ in EC:DEC (1:1)	2.7–4.2	0.1	90	[49]
			94.26	2.04	3.7	0.38	3.03	2070				0.1	100
10	Peanut skin	HTC	n.a.	n.a.	n.a.	0.37	—	1900	80/5/15, PC/PVDF/CB	NaClO_4_	1.5–4.2	0.1	153	[170]
11	Grinded cork	Thermal treatment	n.a.	n.a.	n.a.	n.a.	n.a.	2750	80/10/10, PC/CMC/CB	1 M NaClO_4_ EC:DEC (1:1)	2.0–4.2	0.1	110	[171]
			n.a.	n.a.	n.a.	n.a.	n.a.	2590					100
			n.a.	n.a.	n.a.	n.a.	n.a.	2770					110	
			n.a.	n.a.	n.a.	n.a.	n.a.	2910					114	
12	Peanut skin	HTC	85.91	0.58	9.7	0.379	2.44	2396	75/10/15, PC/PVDF/CB	1 M NaClO_4_ EC:DEC (1:1)	1.5–4.2	0.1	159.75	[172]
			89.45	0.85	9.7	0.412	1.79	1998					153.75	
			87.31	0.96	11.73	0.411	1.61	1376					135	
13	Jute	HTC	n.a.	n.a.	n.a.	0.384	3.11	1529	80/20, PC/PVDF	1 M NaPF_6_ in EC:DEC (1:1)	1.5–3.2	0.1	70	[161]
14	Corn stalk	Salt templating	76.7	6.4	16.9	n.a.	2.23	2650	70/10/20, PC/PVDF/CB	1 M NaPF_6_ DME	2.0–4.0	0.1	114	[173]
			89.3	3.7	9	n.a.	2.02	1802					78	
			68.1	3.2	28.7	n.a.	2.37	1458					60	
			77.3	2.5	18.2	n.a.	2.10	1452					60	
15	Coconut shell	HTC	n.a.	n.a.	n.a.	n.a.	0.85	1795	80/10/10, PC/ PVDF/CB	1 M NaClO_4_ in EC:DMC (1:1)	2.7–4.3	0.1	83	[108]
16	Tremella	Thermal treatment	n.a.	n.a.	n.a.	n.a.	1.03	2520	80/10/10, PC/ PVDF/CB	1 M NaClO_4_ in EC:DEC (1:1) with 5 wt. % FEC	1.5–4.2	0.175	185.7	[175, 176]
17	Gelatin	HTC	85.5	2.5	12	0.385	1.26	2600	80/10/10, PC/ PVDF/CB	1 M NaClO_4_ EC:DEC (1:1)	2.0–4.2	0.035	119	[177]
			84.3	n.a.	15.7	0.359	1.23	1911				0.035	74	
18	Citrus peel	Thermal treatment	n.a.	n.a.	n.a.	n.a.	0.94	1167	80/10/10, PC/ PVDF/CB	1 M NaClO_4_ EC:PC (1:1)	2.7–4.2	0.1	140	[178]
19	Camelia shell	Salt templating	85.41	1.25	13.34	0.39	1.29	2186	70/10/20, PC/PVDF/CB	1 M NaClO_4_ EC:DEC (1:1) with 5 wt. % FEC	0–4.0	0.1	104.2	[110]
20	Cuttle bone	Thermal treatment	n.a.	n.a.	n.a.	n.a.	n.a.	3229	80/10/10, PC/ PVDF/CB	1 M NaClO_4_ in EC:DEC (1:1)	2.7–4.2	0.1	50	[179]
21	Corn husk/GO	HTC	n.a.	n.a.	n.a.	0.4	n.a.	2224	80/10/10, PC/CMC/CB	1 M NaClO_4_ EC:DEC (1:1) with 5 wt. % FEC	2.0–4.2	0.1	116	[180]
			n.a.	n.a.	n.a.	0.4	n.a.	2124					70	
			n.a.	n.a.	n.a.	0.4	n.a.	2240					65	
			n.a.	n.a.	n.a.	0.4	n.a.	2250					72	
22	Olive pits	Thermal treatment	n.a.	n.a.	n.a.	n.a.	1.08	2138	80/10/10, PC/ PVDF/CB	1 M NaPF_6_ EC:PC (1:1)	1.6–3.0	0.1	50	[181]

n.a., not applicable.

Other electrolytic salts, including NaTFSI, have been recently shown to be promising electrolytes for SIC due to their good electrochemical stability with commercial activated carbon, comparable to conventional NaPF_6_‐based electrolytes [65]. Although NaTFSI is impractical for full cells (oxidation occurs from 4.0 V), [65] necessitating the design of new additives and solvents. Organic solvents like carbonates and ethers, including EC, DMC, PC, DE, and DME are commonly used in electrolytes (Figure 35b). These solvents are typically combined with electrolyte salts. Carbonate‐based solvents (EC, DMC, PC, DEC) dominate the formulations due to their wide electrochemical stability window, good ionic conductivity, and stable SEI/CEI formation [300, 301]. Additives such as FEC and VC are often added in small proportions to stabilize the electrode‐electrolyte interphase, improving energy density, cycling performance, and safety [29, 302].

## 7 Porous Carbon Charge Storage Mechanisms

The charge storage mechanisms occurring in the porous carbon positive electrode in SIC are often time misconstrued and lack a clear understanding. This section provides a detailed overview regarding the potential window, open circuit potential (OCP), point of zero charge (PZC), the capacitive charge storage mechanism, pseudocapacitive mechanism, and the solid electrolyte interphase.

An important aspect of the electrochemical testing of porous carbon positive electrode versus Na metal is in the determination of the electrochemical stability window (potential window), open circuit potential (OCP), and the point of zero charge (PZC). The OCP is the open circuit voltage of the porous carbon fresh cell when in contact with Na metal without the application of current or voltage. Typical OCP of fresh porous carbon with Na metal is between 2.8 and 2.9 V [23, 33, 52]. The reversible electro‐sorption of the negative electrolytic ions into the microporous channels takes place in the potential above the OCP due to positive polarization, and below the OCP the adsorption/desorption of the electrolytic cations occurs [33].

**FIGURE 36:**
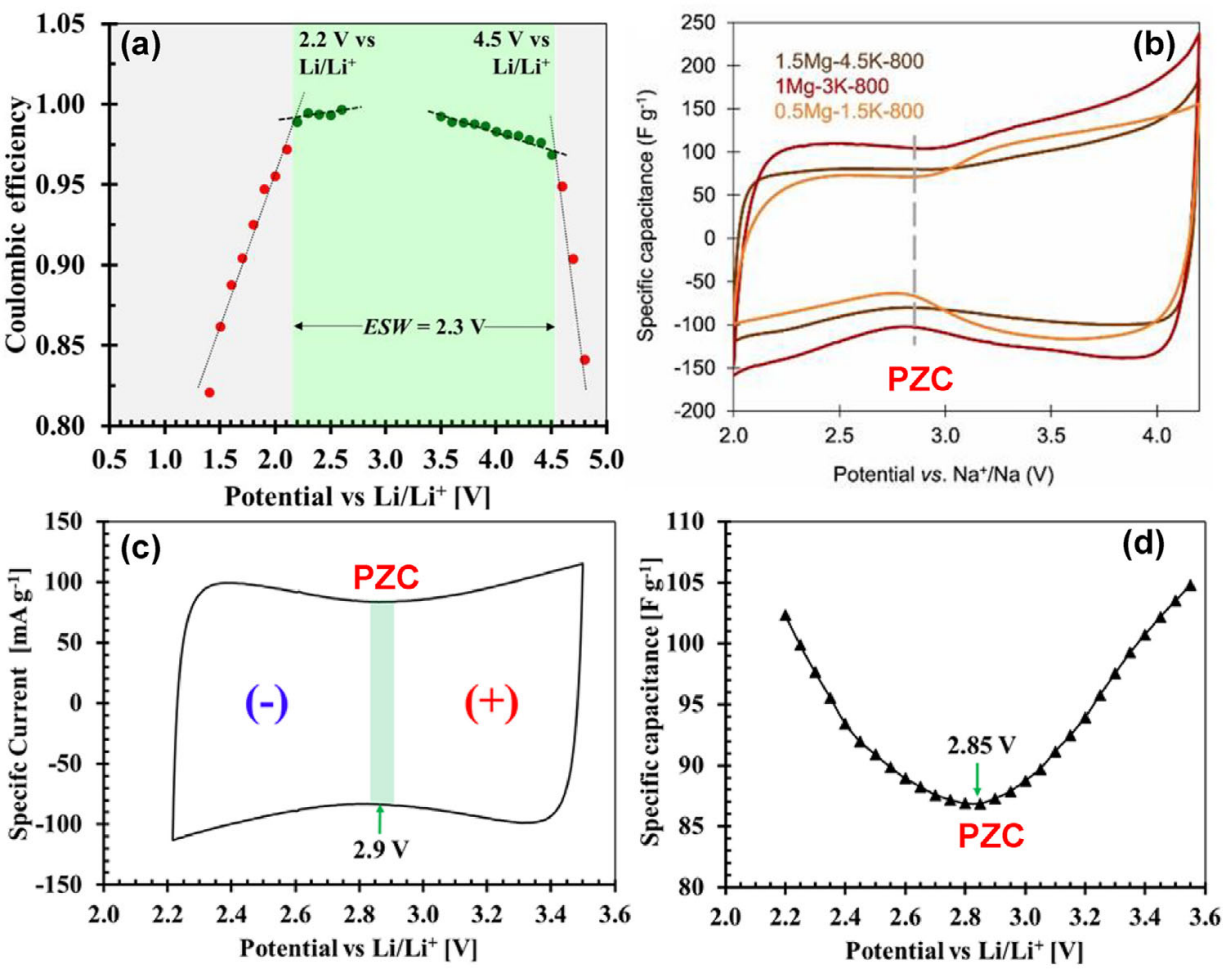
(a) Plot of the Coulombic efficiency vs. the terminating potential from the OCP, Reprinted with permission [306] under the terms of CC‐BY license. (b) Cyclic voltammetry of porous carbon based on tannic acid at 1 mV s^−1^ between 2.0 and 4.2 Na^+^/Na, showing the PZC. Reprinted with permission [52] Copyright (2025), Elsevier. (c) CV curve of porous carbon at 1 mV s^−1^ in LiPF_6_ electrolyte for PZC determination. (d) Plot showing the capacitance value as a function of potential as obtained from the SPEIS experiment for PZC determination. Reprinted with permission [306] under the terms of CC‐BY license.

On the other hand, the potential window helps in achieving a high energy density based on the direct relationship between capacitance and the voltage (*E*  =  0.5 *CV* ^2^). Often, the potential window depends on the type of electrolyte used in electrochemical testing. Typical electrolytes such as NaClO_4_ and NaPF_6_, which are the most used electrolytic salt, have been reported to demonstrate a large voltage window up to 5 V vs. Na/Na^+^ without decomposition [303, 304]. One way to do this is using the opening method, as described in detail by Xu et al. [305] and also reported by Parejo‐Tovar et al. [306] for LIC. Briefly, it involves performing CV experiments at a low scan rate at a potential lower or higher than the OCP. The Coulombic efficiency (CE) obtained by dividing the anodic and cathodic capacities is then plotted against the terminating potential in the voltage window. The potential at which the CE is closer to 1 is thus selected as the operating range (operating potential window), as shown in Figure 36a for a LIC.

Moreover, the point of zero charge is the potential point where the least capacitive current in the cyclic voltammogram is observed. The PZC is an important factor in determining the charge storage mechanisms in energy storage systems. It provides information about the potential where charges are not required for the electric double layer formation [307]. CV experiment is one of the easiest ways to determine the PZC. This is mostly done by selecting a potential range from the lower limit of the electrochemical stability window (ESW) and a potential slightly higher than the OCP. From the obtained CV curve at a low scan rate, the region at which a drop in the capacitive current or flat shape is observed is thus referred to as the PZC potential. For instance, Paya and co‐workers [52] recently showed the PZC of tannic acid‐derived porous carbon as a positive electrode for SIC. CV was performed at a scan rate of 1 mV s^−1^ between a potential window of 2.0–4.2 Na^+^/Na, as shown in Figure 36b. A distortion in the CV curve, known as the PZC, is observed at 2.9 V. A similar observation was reported by Parejo‐Tovar et al. [306], when testing YP‐50F commercial activated carbon vs. Li metal in LiPF_6_ electrolyte, the PZC was observed at 2.9 V, which corresponds to the potential of the least capacitive current, as seen in Figure 36c. Another approach in the determination of the PZC is via the use of step potential electrochemical impedance spectroscopy (SPEIS), which involves performing impedance at different potential ranges, and the obtained resistance is used in the calculation of the capacitance. The potential corresponding to the least observed specific capacitance is thus taken as the PZC. The PZC result of this technique has been reported to be consistent with that of the CV experiment for lithium‐ion capacitors, as shown in Figure 36c and d. Also, in the work of Slesinka et al. [307], the authors observed comparable results when determining the PZC in electrochemical capacitors using CV and SPEIS experiments.

Therefore, for the porous carbon positive electrode, the location of the PZC is imperative as it determines the ions involved during the charge and discharge process. The use of advanced characterization techniques such as electrochemical quartz crystal microbalance (EQCM) and electrochemical dilatometry (ECD) is proposed to further elucidate the charge storage mechanisms of ions in the positive electrode.

### 7.1 Capacitive Mechanism

The capacitive charge storage mechanism is based on the adsorption and desorption of ions at the electrode‐electrolyte interface, in the absence of charge transfer reactions. This behavior is often seen in high specific surface area electrode materials, which are characterized by the electric double layer (EDL). The capacitive mechanism is developed by the arrangement of ions and/or electrons towards the electrode‐electrolyte interfaces. When the electrodes are polarized, there is an ion migration, particularly the cations and anions relocate to the negative and positive electrodes, respectively. In the absence of polarization, the ions are removed from the electrodes and recombined in the electrolytic solution. This is the concept of the charge and discharge for the electric double‐layer capacitive storage mechanism.

**FIGURE 37:**
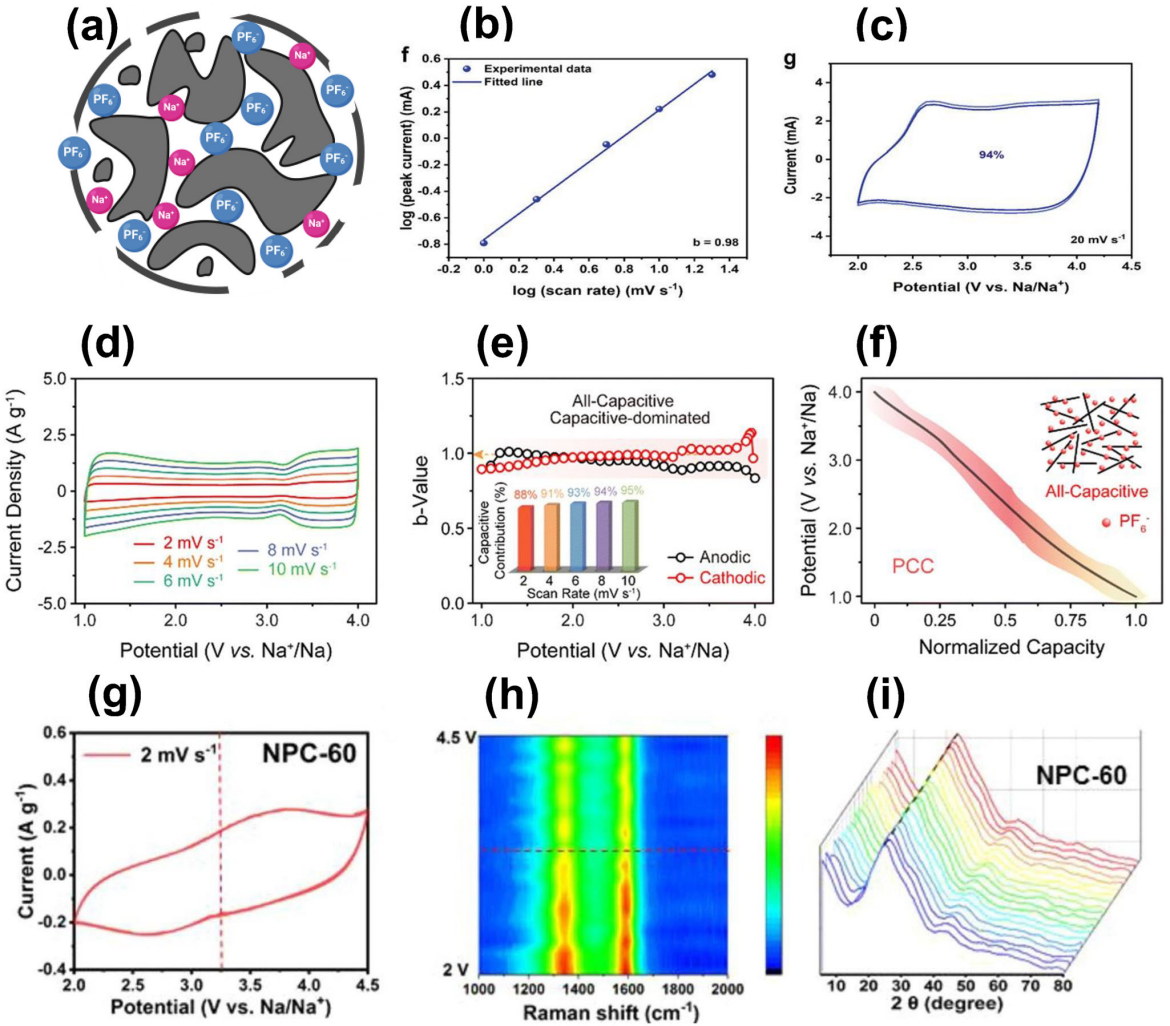
(a) Schematics showing the electrolytic ion adsorption in PC. Created by the authors using www.biorender.com. (b) Plot of log (current) versus log (scan rate) from peak currents. (c) Capacitive contribution of K‐MDC(20) at 20 mV s^−1^ vs. Na metal half‐cell in 1 m NaClO_4_ in EC/DEC (1:1 v/v) electrolyte, Reprinted with permission [80] Elsevier under the terms of CC‐BY license. (d) CV curves between 2 and 10 mV s^−1^, (e) b‐values at different potentials and the contribution of capacitive controlled processes, and (f) capacitive storage mechanisms diagram vs. Na metal half‐cell in 1 M NaClO_4_ in PC (5% FEC) electrolyte. Reproduced from [158], the Royal Society of Chemistry. (g) CV curve, (h) ex‐situ Raman, and (i) ex‐situ XRD patterns of NPC‐60 positive electrode in NaClO_4_ electrolyte, Reprinted with permission [109] Copyright (2021), Elsevier.

In the porous carbon positive electrode for SIC, the adsorption‐desorption of the positive (Na^+^) and negative (PF_6_^−^) electrolytic ions occurs in and out of the microporous channels present in the material, as shown in Figure 37a. One of the widely used approaches in the determination of the capacitive mechanism via CV curve is the use of the power law relation [308], *i*  =  *av^b^*, where *i* and *v* are the current (mA) and scan rate (mV s^−1^), respectively, and *a* and *b* are constants. The value of the *b* is determined from the slope of the linear plot between *Log*(*i*) vs. *Log*(*v*), while *a* is determined from the intercept, as shown in Figure 37b. When the value of b is close to 0.5, it indicates the process mechanism of ions to be diffusion‐controlled into the porosity, while a value of b closer to 1 verifies the capacitive mechanism, where the electro‐sorption of the electrolytic ions takes place in the pore channels. Jung and co‐workers [80] reported a value of b close to 1 (Figure 37b) for MOF‐derived porous carbon, K‐MDC(20), which confirms the capacitive nature of the charge storage process.

**FIGURE 38:**
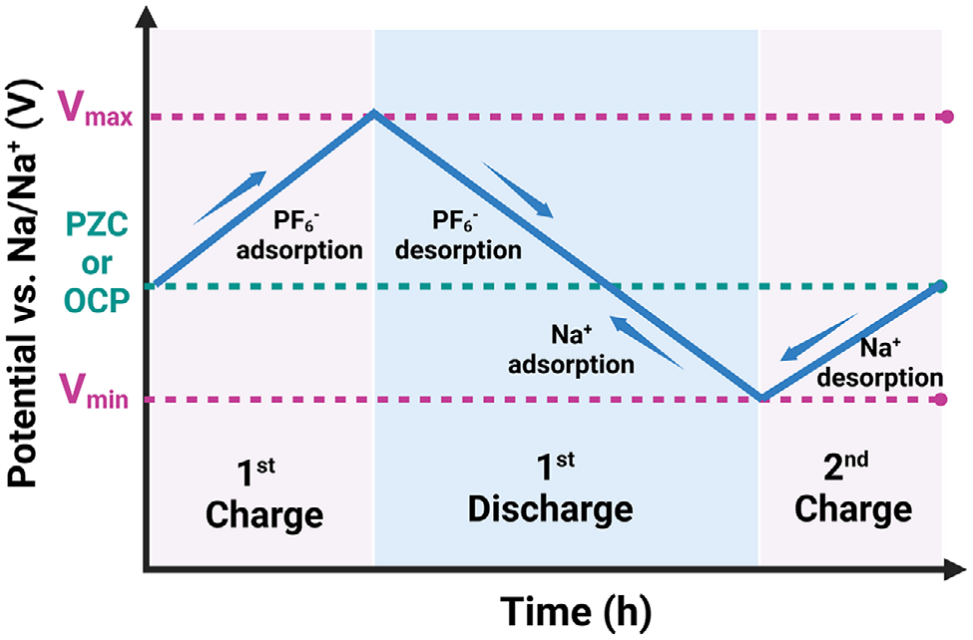
Proposed schematic of the galvanostatic profile of porous carbon positive electrode during the initial charge/discharge and second charge sequence vs. Na metal half‐cell showing the adsorption of anions and cations, possibly involved in the storage mechanisms. Created by the authors using www.biorender.com.

Moreover, Dunn's approach [308] further helps to account for the capacitive and diffusion‐controlled process at different scan rates, using the CV curves. This also relies on the relationship between the current and the scan rate. Porous carbon typically exhibits a strong capacitive response, particularly at high scan rates, as observed in Figure 37c–e. Wang et al. [158] also report CV curves with nearly rectangular shapes, suggesting that charge storage is primarily governed by the electric double‐layer effect. Analysis of the b‐value across various potentials yields values above 0.8, confirming the dominance of the capacitive mechanism. Examination of the PCC discharge curves indicates that the energy storage is a surface‐controlled, fully capacitive process driven by the adsorption and desorption of Na^+^ cations and PF_6_^−^ anions (Figure 37f). Also, in the work of Cai et al. [109], the developed porous carbon from bamboo biomass showed a quasi‐rectangular CV shape (Figure 37g) with the appearance of humps indicative of the presence of both electric double layer capacitance effect and subordinate pseudocapacitance. The authors investigated the ClO_4_^−^ pseudocapacitive reaction mechanisms, and employed ex‐situ Raman spectroscopy to study the NPC‐60 electrode processes during the first charging cycle within a 2.0–4.5 V range. As shown inthe Raman spectra presented in Figure 36h, the charging process can be divided into two distinct stages. Combined with the CV curve in Figure 37g, the *I*_D_/*I*_G_ ratio remains largely stable from 2.0 to 3.75 V, indicating an adsorption mechanism within nanopores during the initial charge state, where ClO_4_^−^ adsorption occurs without significant changes to defects (I_D_) or sp^2^ carbon structures (I_G_). As charging progresses from 3.75 to 4.5 V, the *I*_D_/*I*_G_ ratio decreases, suggesting pseudocapacitive reactions between covalent N/O and ClO_4_^−^, consistent with the CV curve. To further validate these findings, ex‐situ XRD tests were performed on NPC‐60, revealing no significant changes in the (002) and (100) peak intensities, confirming that the positive electrode mechanism in SICs is primarily capacitor‐based (Figure 37i).

In this regard, the charge storage mechanism in the porous carbon positive electrode has become an interesting subject, owing to the different information on the capacitive behavior happening in the positive electrode. The schematic presented in Figure 38 explains the potential variation of the porous carbon positive electrode in the Na metal half‐cell. We propose that the charge exchange is thus divided into three sections (Figure 38). In the first charge, the porous carbon electrode is polarized above the OCP or PZC up to the maximum potential limit (V_max_, potential < electrolyte decomposition). During this period, there is electro‐sorption of the negative electrolytic ions (PF_6_^−^) in the micropores. In the second stage, the first discharge process, the anions are desorbed until the PZC or OCP, then below this potential value, the adsorption of the electrolytic cations, Na^+^, takes place until the lower potential (V_min_) is reached. In the last stage, the porous carbon is polarized to the PZC or OCP via the charge process, during which the desorption of the Na^+^ ions occurs. However, worth noting that the schematic we propose has no reported experimental details to support it, even though most of the works consider only the anion adsorption—as the main mechanism in the PC positive electrode.

Although Shellikeri et al. [309], Levi et al. [310], Zheng et al. [311], and Parejo‐Tovar et al. [306] have investigated the electrochemical processes involved in lithium‐ion transformations in LICs using techniques such as in situ NMR, theoretical calculations, electrochemical quartz crystal microbalance (EQCM), operando electrochemical dilatometry (ECD), and in situ potentiostatic electrochemical impedance spectroscopy (PEIS), tahe fundamental ion‐shuttling mechanisms during charge and discharge are as follows:

The potentials of the negative electrode (negative electrode) and positive electrode (positive electrode) are referenced individually. During charging (above open‐circuit voltage, OCV): PF_6_^−^ anions from the electrolyte are attracted to the positive electrode (positive electrode), where they adsorb onto the porous carbon surface. Simultaneously, Li^+^ ions are drawn to the negative electrode (negative electrode), where they intercalate or dope into the carbon structure (Li doping/pre‐lithiation). During discharging, anions desorb from the positive electrode surface and return to the electrolyte. At the negative electrode, pre‐doped Li is oxidized, releasing Li^+^ ions into the electrolyte. These Li^+^ ions then migrate to the positive electrode, where they form or reinforce an electric double layer on the carbon surface. During charging below OCV: Li^+^ ions desorb from the electric double layer at the positive electrode and are pulled toward the negative electrode, where they are re‐inserted (doped) into the carbon structure.

Thus, further investigations are required to unravel this proposed schematic for porous carbon positive electrode behavior in the SIC system.

**FIGURE 39:**
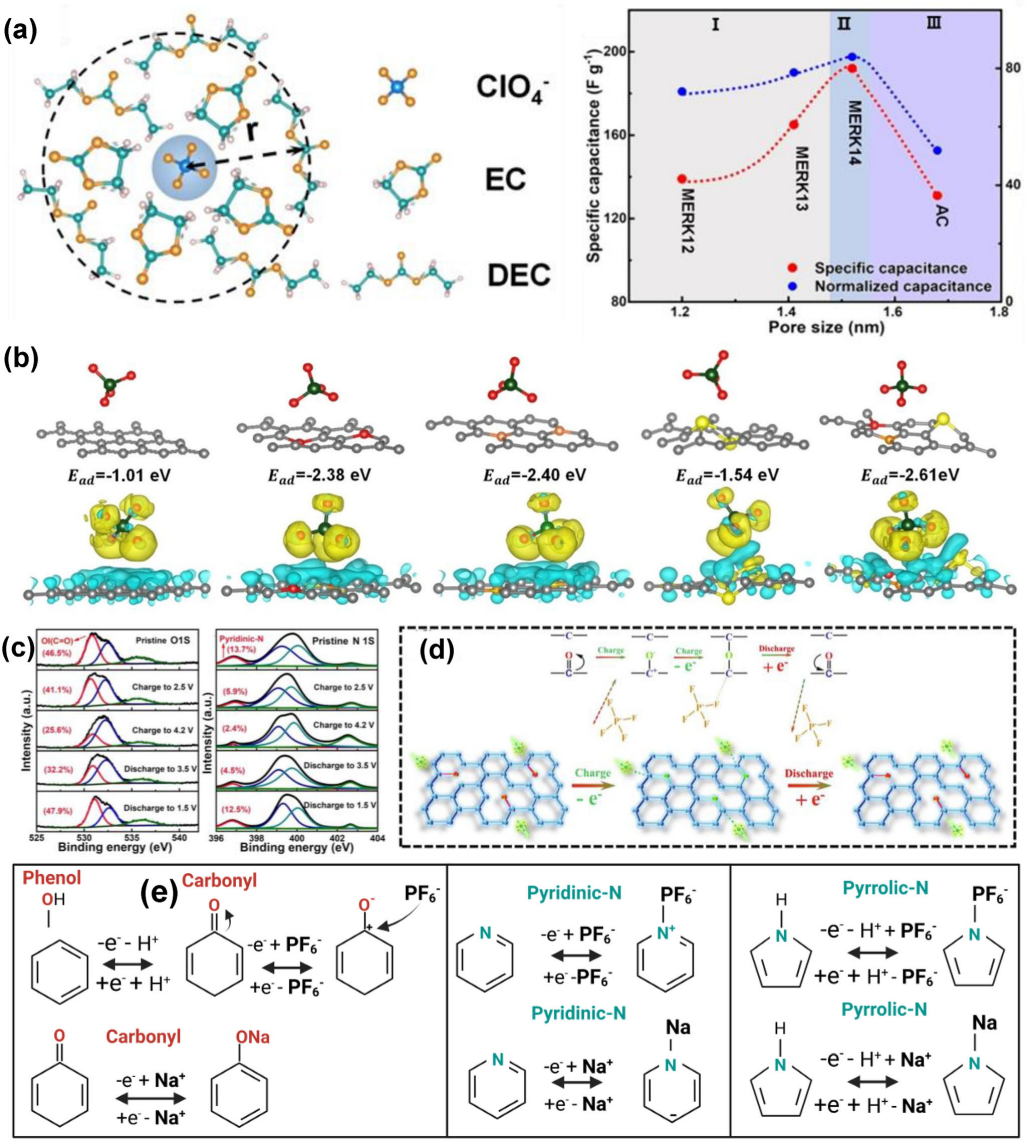
(a) Full solvation shell of ClO_4_^−^ ions and specific and normalized capacitance dependence on the pore size. (b) Optimized structures and absorption energy of ClO_4_^−^ ion adsorbed on pristine, O, N, S‐doped, and ternary‐doped carbon. (c) ex‐situ XPS spectra of O 1s and N 1s at specific different states of MERK14, Reprinted with permission [51] Copyright (2022), Elsevier. (d) Faradaic mechanism based on C = O functional group in lithium‐ion capacitors, Reprinted with permission [270] Copyright (2022), Elsevier. (e) possible pseudocapacitive charge storage mechanism based on the redox activity of PF_6_^−^ and Na^+^ with phenolic, pyridinic‐N, and pyrrolic‐N groups. Reproduced from [23], the Royal Society of Chemistry.

Away from the intrinsic effect of the specific surface area and microporous/mesoporous volume, porous carbon pore size represents a critical factor affecting carbon capacitance. In the pioneering work of Chmiola et al. [6] and Raymundo‐Piñero et al. [245], the authors demonstrated that nanopores less than 1 nm showed an increase in capacitance. This finding challenges the traditional belief that pores smaller than solvated electrolyte ions cannot contribute to charge storage. However, research activities on ion solvation are heavily centered on the cations, such as Li^+^ and Na^+^, with electrolytic aqueous solvent clusters. Of equal importance is the desolvation concept of the anions for the porous carbon positive electrode. Liu et al. [51] investigated the solvation mechanism of electrolytic ions in NaClO_4_ in EC:DEC using DFT calculations, where it was shown that solvated ClO_4_^−^ ion has to overcome a large energy barrier to enter smaller pores than the larger pores, and this energy can be lowered with the presence of heteroatoms. Moreover, the solvation shell of ClO_4_^−^ ion randomly arranged with EC and DEC molecules, which form a thermodynamically stable diameter of 1.48 nm (Figure 39a). When the pore size matches the solvated ion diameter (1.48 nm), there is an elevated capacitance due to favorable ion packing. However, in the case of larger pores (> 1.48 nm) than the solvated electrolytic ions, a decrease in capacitance is experienced and an increase in the distance between the pore walls and ions, which limits storage efficiency (Figure 39a). Thus, the optimum state towards ion adsorption /desorption is when the ion size matches or is less than that of the pore size, in line with organic/aqueous/ionic liquids electrolyte observations [231, 261].

### 7.2 Pseudocapacitive Mechanism

Delivering high energy and power density SIC requires the benefit of pseudocapacitive ion storage, and the understanding of this charge storage mechanism is imperative, particularly in a porous carbon positive electrode. Often, pseudocapacitive charge storage is linked to the presence of heteroatoms on the surface of porous carbon. Redox processes between ions and the functional groups are mostly investigated via both experimental and computational approaches (DFT calculations). For instance, Liu et al. [51] investigated the adsorption energies of ClO_4_^−^ ion on the surface functional groups modified by oxygen (O), nitrogen (N), and sulfur (S) and a combined ternary O, N, and S mixture. These functional groups showed higher energies (−1.54 eV to −2.61 eV) compared to pristine carbon (−1.01 eV), due to accumulated charge densities, which improve the charge kinetics (see Figure 39b). The investigation of capacitive and redox charge storage kinetics via XRD and XPS analyses revealed that aside from the presence of capacitive charge storage, there are also some reversible pseudocapacitive reactions with the C═O carbonyl and pyridinic‐N functional groups during charge and discharge processes (see Figure 39c). This finding is also corroborated by a recent study by Li et al. [151], where the authors showed that oxidation of groups such as –H and ─OH on Csp^3^ helps in the formation of C═O carbonyl groups, which are highly electrochemically active on the surface of highly porous carbon. Another interesting finding is the work of Adeniji et al. [151], where the authors showed that the presence of phenolic/ether groups varies linearly to the specific capacity via pseudocapacitive storage contribution. In the work of Zou and co‐workers [270] on lithium ion capacitors, the authors demonstrated the interconversion process that takes place between C═O and C─O, which helps in the generation of a carbon atom that is slightly electropositive, which attracts PF_6_^−^ion (Figure 39d). Adeniji et al. [23] and Huo et al. [273] also explained that particular nitrogen functionalities, such as pyrrolic‐N and pyridinic‐N, are active species in nitrogen‐based reversible pseudocapacitive reactions (Figure 39e).

## 8 Porous Carbon Ageing and Cathode Electrolyte Interphase (CEI)

Porous carbon ageing issues are often seen when working in high voltage regimes and temperatures, which result in rapid electrochemical performance loss (low capacity, increasing internal resistance and charge transfer resistance), leading to cell breakdown with safety concerns [312]. The ageing process includes the generation of side reactions leading to electrolyte degradation, porosity blockage, and build‐up of cell internal pressure as a result of the volatile gas products. The side reactions are closely related to the porous carbon surface morphology, structure, surface chemistry, and the electrolyte properties. The precursor type and the activation process used in the generation of a high specific surface area porous carbon play a major role in the charge storage kinetics and electrode ageing pathways [313]. In conventional electrochemical capacitors, functional groups such as carboxyl, lactone, and phenol groups have been reported to cause the presence of leakage currents and gas release during cycling in organic electrolytic medium [314].

Porous carbon ageing can be investigated via testing protocols, such as the cycling stability test via galvanostatic charge discharge or the floating test method. The cycling stability tests are straightforward and record several thousand GCD cycles, which can be time consuming. The floating protocol employs a potentiostatic hold, completed in a shorter time frame. This test has been documented in the literature for porous carbon positive electrodes in electrochemical capacitors [315, 316], LIC [271], and SIC [65]. The cycling and floating test exert different impacts on the testing operation time, electrode properties, and cell resistance in aqueous solution. Cycling test induces changes in the carbon structure without a significant effect on the recorded resistance. However, the floating test is often referred to as the “accelerated ageing test” with a significant impact on the resistance, due to high exposure to high potential hold of the porous carbon electrode. The floating test examines the state of health (SOH) of energy storage devices rapidly, including sodium ion capacitors [65]. Monitoring the process involves keeping a record of the capacity loss and an increase in the internal resistance (solution resistance). Additionally, the international standard (IEC 62391‐1), [317] considers a system failure with a capacity loss below 80% or a doubled internal resistance. For example, Platek et al. [316] investigated the performance fade in electrochemical capacitors with a 1 mol·L^−^^1^ KI redox‐active electrolyte using commercial activated carbon electrodes. Floating tests were more detrimental than the cycling test, triggering redox side reactions that increase system resistance via polyiodide deposits blocking micropores, with possible iodate/carbonate salt contributions. Cycling tests, while longer, cause less resistance change but more pronounced carbon structure damage due to ionic fluxes. Both tests reduce the carbon SSA, similarly, correlating with capacitance fading. There is no work for SIC in this direction.

The solid electrolyte interphase (SEI) is one of the most highly discussed phenomena in the field of electrochemical energy storage, especially in lithium and sodium ion batteries. This is a phenomenon that is mostly studied on the negative electrode, such as in graphite (for LIB) and hard carbon (SIB) [279]. The SEI forms predominantly on the negative electrode because it operates at low electrochemical potentials (close to or below 0 V vs. Li/Li^+^ or Na/Na^+^) [318]. At these potentials, the electrolyte is thermodynamically unstable and undergoes reductive decomposition, leading to the formation of the SEI. The SEI layer is an insulating layer and a conductor of anions and cations, which assist in the insertion or intercalation of these ions. It can play a protective role for further electrolyte decomposition, and depending on its composition and quality, it strongly impacts the cycle stability and life. The SEI layer is also present in the positive electrode, (often called cathode electrolyte interphase (CEI)), but has less content compared to that of the negative electrode. In LIB, the SEI typically contains densely packed inorganic components such as LiF, Li_2_O, and LiO [319] and loosely packed inorganic and organic species like Li_2_CO_3_ and lithium alkyl carbonate (ROCO_2_Li), respectively, with polymer on the electrolyte side [5]. Also in SIB, Beda et al. [279] reported that SEI formation in hard carbon materials is both driven by electrochemical degradation of the electrolytic salt and solvent. The SEI was reported to consist of both inorganic (Na_2_CO_3_ and NaPF_x_) and organic (ROCO_2_Na, RCO_2_Na, and PEO) core and shell, respectively, which promote a decrease in the resistance of the hard carbon electrode. Other authors reported the presence of other inorganic species, such as NaF and Na_2_O, similar to those in LIB [320].

**FIGURE 40:**
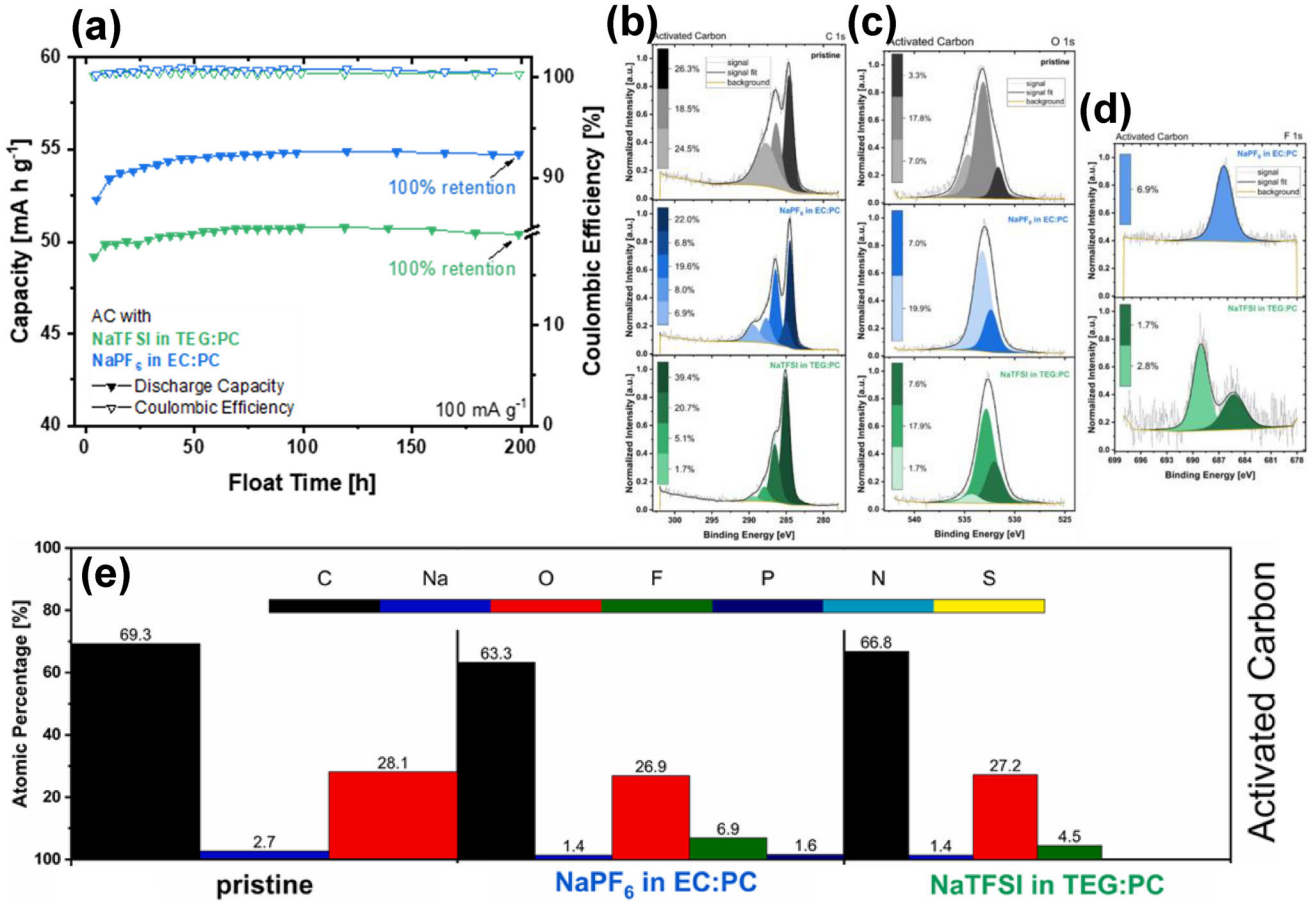
(a) Float test of YP‐50F commercial activated carbon vs. Na metal while holding the potential at 3.8 V. (b) C 1s, (c) O 1s and (d) F 1s high resolution XPS spectra (e) Surface elemental compositions of pristine and floated activated carbon electrodes in NaPF_6_ and NaTFSI based electrolyte, Reprinted with permission [65] under the terms of CC‐BY license.

The development of CEI on a porous carbon positive electrode in SIC is rarely studied. One of the most recent studies investigated the possible development of SEI formation on porous carbon positive electrode in a half‐cell consisting of YP‐50F commercial activated carbon and NaPF_6_ in EC:PC and NaTFSI in TEG:PC electrolytes [65]. The authors investigated the reason for the high floating stability of the carbon electrodes in both electrolytes. The porous carbon positive electrode was put through a floating process between a potential range of 2.2–3.8 V, wherein the potential was held at 3.8 V for 5 h at 0.1 A g^−1^. After about 200 h, the PC electrode demonstrated 100% stability (see Figure 40a). Postmortem study via XPS was conducted to reveal the decomposition products on the surface of the PC electrode (Figure 40b‐e). The results show a slight reduction in carbon content, accompanied by an increase in fluorine content and a minor increase in oxygen content, indicating alterations in the electrode surface chemistry (Figure 40e). In addition, F1s peaks indicate different fluorinated compounds formed, revealing the formation of NaPFx (in EC:PC) and NaF + NaPFx (in NaTFSI), as shown in Figure 40d.

The authors could not establish a concrete conclusion on whether there is a possibility of CEI formation on the AC electrode. Additionally, a critical look into the experiment reveals that the floating of the electrode was performed at a relatively low potential window (2.2–3.8 V) and low maximum potential (3.8 V). Thus, future work should investigate expanding the potential window for the adequate investigation of CEI formation on the porous carbon electrode in SIC. Particularly, the phenomenon has been recently revealed in the case of a porous carbon positive electrode in the LIC system, [271] where the authors reported CEI formation on the PC electrode when performing floating from a maximum voltage of 4.0 V up to 4.6 V in LiPF_6_ EC:DMC electrolyte. The authors noted structural and chemical modifications on the surface of the electrode. First, porosity changes were observed as a result of pore clogging and species from electrolyte decomposition products. On the other hand, the surface chemistry was modified that caused lower conductivity and disruption to the C‐sp^2^ network. Electrolyte decomposition products comprising both organic and inorganic species, like those found in the SEI content in LIB, were also presented. In light of this, it is important to properly assess the impact of the CEI formation on the porous carbon positive electrode for SIC.

## 9 Full Cell Performance of Porous Carbons With Carbon Negative Electrode Materials

This section explains the full cell performance of a porous carbon positive electrode with a carbon‐based negative electrode. Dual carbon sodium ion capacitors have been tipped to be the future of sodium ion capacitors as they are of a similar material system and have the ability to work in a large potential window [29]. The discussion covers the SIC full cell mechanism, pre‐sodiation process, performance evaluation metrics, and the assembly of carbon‐based and carbon‐composite negative electrodes with porous carbon materials.

### 9.1 SIC Full‐Cell Mechanism

The operating principle of SIC is similar to that of conventional LIC, which consists of a battery‐type negative electrode (hard carbon) where the faradaic process/reaction takes place and a capacitive‐type positive electrode (porous carbon), where electrostatic sorption occurs. These two processes allow to achieve high‐energy and power‐density devices. The first report on SIC assembly was published in 2012, when Chen et al. [321] combined V_2_O_5_/CNT as a negative electrode and activated carbon as a positive electrode between a potential range of 0.1–2.8 V in 1 M NaClO_4_ in PC electrolyte, delivering an energy density of 38 Wh kg^−1^ and a power density of 140 W kg^−1^. In this assembly, during the charging process, sodium ion insertion takes place in the V_2_O_5_/CNT negative electrode, while ClO_4_^−^ anion adsorption occurs in the positive electrode. On the other hand, during the discharge process, sodium extraction and ClO_4_^−^ anion desorption occur at the negative electrode and positive electrode, respectively. In the same period, Kuratani et al. [32] published the first dual carbon sodium ion capacitor that is based on hard carbon negative electrode—for sodium ion insertion/extraction—and an activated carbon positive electrode—for Na^+^ and PF_6_^−^ ions adsorption and desorption—that works between 1.7 and 3.7 V in NaPF_6_ based electrolyte (Figure 4a,b). This work spurred the beginning of dual carbon SIC, which is responsible for the research advancements today, although this technology is not commercially available yet.

### 9.2 Pre‐Sodiation

**FIGURE 41:**
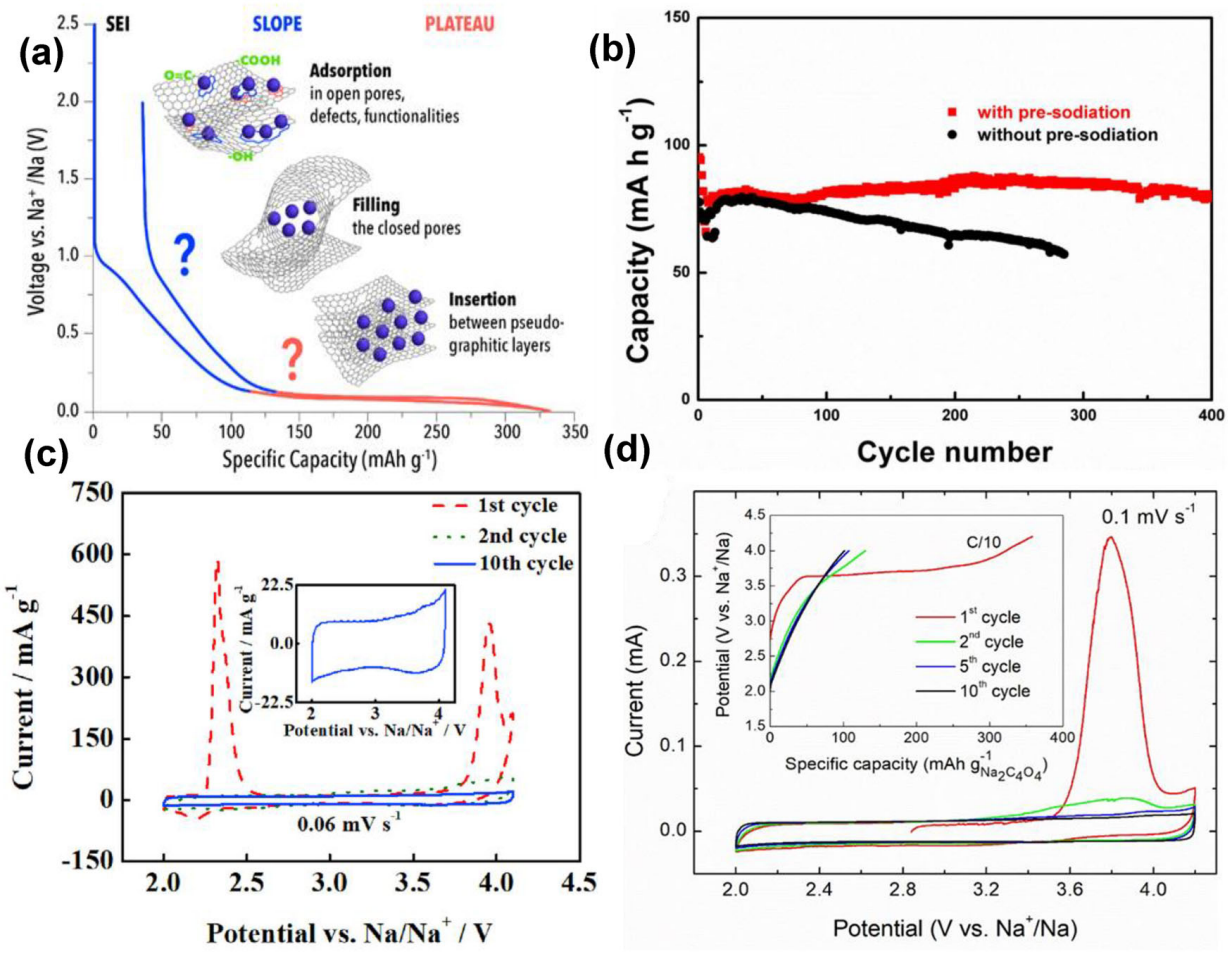
(a) Graphical representation of Na‐ion storage mechanisms in hard carbon and SEI formation during the 1^st^ discharge/charge cycle, Reprinted with permission [14] Copyright (2024), John Wiley and Sons. (b) Cycling performance of sodium dual‐ion batteries with and without pre‐sodiation in 1 M NaPF_6_ in EC/DMC (6:4, v/v) electrolyte, Reprinted (adapted), [323] Copyright (2018) American Chemical Society. (c) Cyclic voltammogram of AC‐Na_2_S vs. sodium counter/reference electrode in 1M NaClO_4_ EC/PC(1:1 vol%) electrolyte at 0.06 mV s^−1^, Reprinted with permission [324] Copyright (2019), Elsevier. (d) Cyclic voltammetry at 0.1 mV s^−1^; inset: Galvanostatic charge at C/10 of AC‐Na_2_C_4_O_4_, in NaPF_6_ in (EC:PC) (1:1 vol%) electrolyte. Reproduced with permission [325], the Royal Society of Chemistry.

The concept of pre‐sodiation involves the addition of sodium into the negative electrode material (hard carbon in this case) from an external source to compensate for the limited sodium present in the electrolyte and its absence in the porous carbon positive electrode. During the initial cycle of SIC, the hard carbon negative electrode exhibits the formation of a solid electrolyte interphase, which leads to irreversible capacity loss (Figure 41a). Pre‐sodiation is very important, as it compensates for this irreversible capacity loss and limits electrolyte decomposition. Since the typical OCP of hard carbon is about 400 mV less than that of porous carbon material (2.8 V vs. Na/Na^+^) due to textural/structural differences, pre‐sodiation helps to reduce the hard carbon OCP to a few mV (< 10 mV), which undoubtedly widens the SIC full cell operating potential window [23, 29]. Thus, the aim of the pre‐sodiation approach is to refill the initial sodium loss, improve the irreversible capacity, and reduce the potential of the negative electrode to benefit and extend the operating window. The cycling stability of the (HC) negative electrode can be improved following pre‐sodiation (Figure 41b).

**FIGURE 42:**
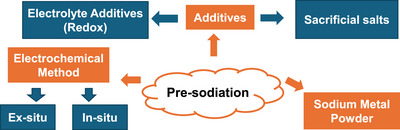
Classification of pre‐sodiation methods of hard carbon in SIC.

Pre‐sodiation can be achieved via three main approaches, which are electrochemical methods, sodium metal powder (SMP), and the use of additives, as shown in Figure 42. The electrochemical method is a widely used method and can be subdivided into ex‐situ and in‐situ methods. The ex‐situ method involves the assembly of the negative electrode against the sodium metal, which acts as both counter and reference electrode, which completes the half cell. The technique was reported by Aida et al. [322] wherein the authors assembled a LIC full cell comprising a pre‐lithiated artificial graphite electrode and a porous carbon positive electrode. The cell is allowed to discharge (sodiation, sodium ion adsorption/filling/insertion into the material, Figure 41a) at a low current density (typically 0.05 A g^−1^)) to ensure a complete electrochemical process. The sequence is followed by a charging process (extraction of sodium), and this goes on for a few cycles (5 cycles; to ensure formation of stable SEI and prevent unwanted side reactions), wherein the sequence terminates with a discharge process. The ex‐situ approach is the most widely used method in the full cell assembly of dual carbon‐based SIC. This procedure was adopted in the first assembly by Kuratani et al. [32], which confirms the efficiency of the hard carbon sodiation steps prior to the SIC full cell assembly.

Despite the advantage of the ex‐situ pre‐sodiation method, this approach is often not sustainable for commercialization. The process is not feasible as it increases costs and steps due to the need for cell opening after pre‐sodiation and a second assembly for coupling with the porous carbon positive electrode. Another concern is the fact that damage to the sodiated electrode may lead to poor performance of the final device. This realization led to the in‐situ electrochemical method, where a sodium metal auxiliary electrode is used to achieve pre‐sodiation via short circuit time control [326, 327]. During this process, sodium metal dissolves in the electrolytic medium, which diffuses across the electrodes in the setup. Although this method eliminates disassembly and assembly as seen for ex‐situ methods via a simplified process, for practical applications, the in‐situ method increases the manufacturing cost. Also, there is a risk of thermal runaway, as the sodium metal still remains in the final device [326].

Another method is the SMP, which involves the direct coating of sodium powder on the electrode surface under a certain pressure or coating dissolved sodium metal in organic solvents such as naphthalene or biphenyl on the prepared electrode surface. For instance, Tang et al. [328] heated sodium in mineral oil, dispersed it into fine particles via ultrasonication, and the resulting SMP was centrifuged in hexane. The SMP dispersion was added drop‐wise to the carbon negative electrode, dried, and pressed mechanically to bring it into close contact. Compared to the untreated negative electrode, the OCV of the cell decreased by ∼1 V, and the first cycle irreversibility dropped from 19.8% to 8%. As a result of the high activity, SMP pre‐sodiation can only be achieved inside the glove box, and the complex nature of the SMP manufacturing process makes it more expensive. In addition, there is a risk of thermal runaway when sodium metal is in contact with the negative electrode. Thus, the SMP pre‐sodiation approach is still in its infant stage and an impractical method for industrial applications.

Sacrificial salts are another class of pre‐sodiation used to replenish sodium ions in the SIC full cell to improve irreversibility in the first cycle. This approach is simpler as it is often utilized for positive electrode materials pre‐sodiation. It involves the electrochemical oxidation of the sodium source present in sacrificial salts during the first charge‐discharge process into the electrolyte and then to the negative electrode material surface to achieve pre‐sodiation. The usability of sacrificial salts is dependent on some major requirements, such as i) high electrochemical reversibility, ii) reduction of chemical and mechanical impact on the porous carbon positive electrode, and iii) compatibility of the salt's oxidation potential with the positive electrode operating voltage window. In the past years, various sacrificial salts have been investigated, such as Na_2_S [324], NaNH_2_ [329], NaBH_4_ [330], NaCN [331], Na_2_C_4_O_4_ [325], Na_2_CO_3_ [332], Na_2_C_3_O_5_ [333], and Na_2_C_2_O_4_ [334], and their properties are detailed in Table 14.

**TABLE 14:** Summary of sacrificial salts properties utilized as porous carbon additives for pre‐sodiation of the negative electrode. **Adapted with permission [** **29** **]** **Copyright (2025), John Wiley and Sons**.

S/N	Positive electrode additives	Oxidation potential (V)	Theoretical/Reversible capacity (mAh g^−1^)	Content in AC electrode (%)	Negative electrode type	Binder type/Solvent	Electrolyte	Toxicity/Air Stability	Source	Gas products
1	Na_2_S [324]	2.2, 3.7–3.8	687/620	40	Sn_4_P_3_	PTFE/H_2_O, Isopropanol and n‐heptane (in glove box)	1 M NaClO_4_ in EC/PC (1:1 vol%)	NO/NO	Commercial	Polysulfides [335]
2	NaNH_2_ [329]	3.8	686/680	25	HC	PTFE/ H_2_O, 2‐propanol and n‐heptane (in glove box)	1 M NaClO_4_ in EC/PC (1:1 vol%)	NO/NO	Commercial	NH_2_NH_2_, N_2_, H_2_
3	NaBH_4_ [330]	2.4–2.7	710/700	25	HC	PTFE/ H_2_O, ethanol and n‐heptane (in glove box)	1 M NaClO_4_ in EC/PC (1:1 vol%)	NO/NO	Commercial	H_2_
4	NaCN [331]	2.9	547/556	36	HC/Sn_4_P_3_	PTFE/ H_2_O and n‐heptane (in glove box)	1 M NaClO_4_ in EC/PC (1:1 vol%)	NO/NO	Commercial	(C_2_N_2_)_n_
5	Na_2_C_4_O_4_ [325]	3.7–4.15	338/275	40	HC	PVDF/NMP	1 M NaClO_4_ in EC/PC (1:1 vol%)	YES/YES	Prepared in the lab	CO, CO_2_
6	Na_2_CO_3_ [332]	3.8–4.4	505/417.1	40	HC	PVDF/NMP	1 M NaClO_4_ in EC/PC (1:1 vol%)	NO/YES	Commercial	H_2_, CO_2_ and CH_4_
7	Na_2_C_3_O_5_ [333]	4.3	331/253	40	—	PVDF/NMP	NaPF_6_ in (EC:PC) (1:1 vol%)	NO/YES	Commercial	CO_2_
8	Na_2_C_2_O_4_ [334]	3.7–4.0	400/393.8	40	HC	PVDF/NMP	1 M NaClO_4_ in EC/PC (1:1 vol%) with 5% FEC	YES/YES	Commercial	CO, CO_2_

From the different reports, the processing of the sacrificial salts can be divided into two groups. The first is the preparation of the salt/porous carbon powder composite in an Ar‐filled glove box due to the instability of the salt in air, such as Na_2_S^337^, NaNH_2_ [329], NaBH_4_ [330], and NaCN [331]. For example, in 2019, Pan et al. [324] introduced a positive electrode containing 40 wt. % sodium sulfide (Na_2_S) for the pre‐sodiation of Sn_4_P_3_ negative electrode in 1 M NaClO_4_ in EC/PC (1:1 vol%). Sodium sulfide exhibits a theoretical capacity of 687 mAh g^−1^, with two oxidation potentials of 2.2 V and 3.7–3.8 V, which are linked to sulfide anion oxidation and sodium extraction, respectively. The method allows for proper pre‐sodiation of the Sn_4_P_3_ negative electrode while releasing hazardous chemicals like polysulfides [335] (Figure 41c).

In the second group, the sacrificial salts can be processed under air, such as Na_2_C_4_O_4_ [325], Na_2_CO_3_ [332], Na_2_C_3_O_5_ [333], and Na_2_C_2_O_4_ [334]. For instance, Arnaiz et al. first reported the use of disodium squarate (Na_2_C_4_O_4_) as porous carbon additive in SIC [325] The disodium squarate have a theoretical capacity of 339 mAh g^−1^ requiring a salt content of 40 wt.% in the porous carbon positive electrode (40 wt.%) to pre‐sodiate the negative electrode in 1 M NaClO_4_ in EC/PC (1:1 vol%). Na_2_C_4_O_4_ has an oxidation potential at 3.7–3.8 V vs. Na/Na^+^, matching the AC potential range, and releases CO and CO_2_ gases as by‐products (Figure 41d). Also recently, sodium mesoxalate (Na_2_C_3_O_5_) was studied as a pre‐sodiation agent by Canal‐Rodriguez et al. [333], having a high reversible capacity of 331 mAh g^−1^. The irreversible oxidation takes place at 4.3 V vs. Na/Na^+^ in NaPF_6_ in (EC:PC) (1:1 vol%) electrolyte. However, there is a risk of hole creation in the electrode carbon structure during salt oxidation.

Redox‐active electrolyte was recently demonstrated by Maćkowiak and co‐workers [336] as a pre‐sodiation attempt to replenish sodium in dual carbon metal ion capacitors. The authors selected thiocyanate salts as a result of their solubility in organic solvents and redox activity in aqueous electrolytes [337]. Thiocyanates were found to be redox‐active species in organic electrolytes at ca. 3.5 V vs. metallic reference electrode. This effective incorporation of redox thiocyanate salt into the electrolytic medium allowed charge compensation in the activated carbon positive electrode, facilitating intercalation/insertion in the negative electrode without requiring electrode modification during cell manufacturing. However, there is a risk of toxic gases formation, such as carbon monosulfide (CS), sulfur monoxide (SO), carbon disulfide (CS_2_), and potentially cyanides [29], which could impact their scalability for practical application.

### 9.3 Performance Evaluators

For the determination of the electrochemical performance of the full‐cell sodium ion capacitor, one of the most used metrics is the energy density (E) in Wh kg^−1^ and the power density (P) in W kg^−1^ and the cycle life. These evaluators can be determined based on the GCPL or GCD curve. Thus, when the full‐cell performance is linear, the energy density (E) is calculated as [19, 20].

(8) $$E\left(\mathrm{Wh}\;kg^{-1}\right)=\frac{I\times \Delta t\times \left(V_{2}^{2}-V_{min}^{2}\right)}{\left(2\times m\times 3.6\right)}$$

When the GCD is not linear;

(9) $$E\left(\mathrm{Wh}\;kg^{-1}\right)=\int \frac{UdQ}{m}=\int \frac{Udt}{m\times 3.6}=I\times \int \frac{Udt}{m\times 3.6}\;\;$$

where U is the potential of the cell in volts, *m* is the mass (g) of the active material, and *t* is the discharge time in seconds.

The power density (P) is calculated as;

(10) $$P\left(W\;kg^{-1}\right)=\frac{3600\times E}{\Delta t}$$

where *V*_2 _ =  *V*_*max*_ −  *IR_drop_* and the *IR_drop_* is the ohmic drop term to be subtracted from the maximum voltage (*V_max_*). The ratio of the mass of the negative electrode to the positive electrode is evaluated with the equation below:

(11) $$m_{a}C_{a}V_{a}=m_{c}C_{c}V_{c}$$

where *m* is the mass (g) of the negative electrode or positive electrode, *C* is the discharge‐specific capacity (mAh g^−1^) of the negative electrode or positive electrode, and *V*is the potential window (V) of the negative electrode or positive electrode.

### 9.4 Carbon Negative Electrode

Hard and soft carbon are the most common negative electrode materials for sodium‐ion capacitors, thanks to their electrochemical advantages and high potential for industrial applications. They possess high discharge capacity, low potential faradaic reactions for efficient energy storage, and high‐rate performance compared to other alloy‐based materials [338]. Hard carbons consist of a mixture of disordered‐like domains with pseudo‐graphitic‐like short domains stacked in 3–5 layers and open/closed pores with interconnected channels that promote the ions migration. HCs can be produced from different families of precursors, ranging from synthetic to biomass to biopolymers, which are thermally treated between 800 and 1500°C [14]. For example, Adeniji et al. [23] utilized rice starch‐based HC negative electrode to assemble a full cell SIC with a nitrogen‐doped porous carbon. The HC negative electrode was pre‐sodiated vs. Na metal, and the initial Coulombic efficiency (ICE) was 88%, with a 5^th^ discharge capacity of 320 mAh g^−1^ at a C/10 rate (37.2 mA g^−1^). The assembled full cell achieved a maximum energy density of 209 Wh kg^−1^ at a maximum power density of 6000 W kg^−1^ within a potential window of 1.5–4.5 V. Also, Liu et al. [51] prepared HC material from magnolol‐based epoxy resin (MER) and thermally treated it between 1200 and 1500°C. The material prepared at 1200°C possesses a large pseudo‐graphitic spacing, which favors fast Na‐ion kinetics and enables efficient performance. The authors combined pre‐sodiated optimized HC (negative electrode) with KOH‐activated MER porous carbon (positive electrode) for full cell assembly in 1 M NaClO_4_ EC:DEC electrolyte at a voltage window of 1.0–4.0 V. The optimized SIC (MER1200//MERK14‐1.5) with a 1:1.5 mass ratio delivered energy densities between 85 and 195.4 Wh kg^−1^ and power densities between 72.3 and 13800 W kg^−1^, with cycling stability of 91.2% after 16,000 cycles.

On the other hand, soft carbons (SCs) are derived from aromatic hydrocarbons such as asphalt tar, petroleum, and coal refining products with low oxygen content. SC materials exhibit fewer defects and relatively high crystallinity, with a structure comprising both disordered (high strain) and graphitic (low strain) regions that enhance electrical conductivity. Despite their high conductivity and reduced defects, SCs generally offer limited sodium‐ion storage capacity (100–250 mAh g^−^^1^) due to narrow interlayer spacing and limited closed pores, leading to charge/discharge curves without a plateau and lower specific capacity compared to HC [339, 340]. Soft carbon offers distinct benefits, including excellent electrolyte compatibility and the absence of a distinct charge‐discharge plateau, which ensures good stability via gradual voltage change [340]. For instance, Chen and co‐workers [137] assembled an SIC full cell made up of a pre‐sodiated soft carbon negative electrode and a synthesized nitrogen‐doped microporous carbon. The SC negative electrode demonstrated a reversible capacity of 110 mAh g^−1^ and a corresponding capacity retention of 73%, which was acquired after 5000 cycles at a current density of 200 mA g^−1^. The obtained full cell, with the voltage window of 0–4.7 V versus Na/Na^+^, shows a high energy density of 245.7 Wh kg^−1^ at 1626 W kg^−1^ and a high‐power density of 13846.1 W kg^−1^ at 50 Wh kg^−1^. The performance of other carbon‐based negative electrodes is summarized in Table 15.

**TABLE 15:** Comparison of electrochemical performance of dual carbon‐based sodium‐ion capacitors from the literature. Reproduced with permission [23], the Royal Society of Chemistry.

S/N	Materials type: negative electrode//positive electrode	Pre‐sodiation method	Potential window (V)	Electrolyte	Maximum ED (Wh kg^−1^)	Maximum PD (W kg^−1^)	Cycling stability (cycles)	Refs.
SIC–carbon negative electrode//porous carbon positive electrode								
1	Hard carbon//porous carbon	Electrochemical (ex‐situ)	1.5–4.5	1 M NaPF_6_ EC:DMC (1:1)	209	6000	80% (4200)	[23]
2	Hard carbon//porous carbon	Electrochemical (ex‐situ)	1.0–4.0	1 M NaClO_4_ EC:DEC (1:1)	195.4	13800	91.2% (16000)	[51]
3	Reduced graphene oxide//reduced graphene oxide	Electrochemical (ex‐situ)	1.0–4.0	1 M NaPF_6_ DME	91	10896	98% (1800)	[72]
4	Carbon sponges//porous carbon	Electrochemical (ex‐situ)	1.0–4.0	1 M NaClO_4_ EC:DEC (1:1)	120	24400	78% (10000)	[121]
5	Hard carbon//porous activated carbon	none	0.15–3.29	1 M NaPF_6_ EC:DEC (1:1)	86	3440	92% (1000)	[161]
6	Porous framework carbon//porous framework activated carbon	Electrochemical (ex‐situ)	0–4.0	1 M NaClO_4_ EC:DMC (1:1)	101.6	20000	71.8% (10000)	[122]
7	Metal–azolate framework‐6s‐ derived carbons//KOH‐assisted pyrolysis of MDCs	Electrochemical (ex‐situ)	0–4.0	1 M NaClO_4_ EC:DEC (1:1)	100	20000	80% (1000)	[80]
8	N, P, O ternary‐doped mesoporous carbon//alkaline‐activated PNPOC‐800	Electrochemical (ex‐situ)	0–4.0	1 M NaPF_6_ EC:DEC (1:1)	105.5	13590	87.4% (9000)	[125]
9	S‐doped carbon nanoparticles//activated nanoparticles	Electrochemical (ex‐situ)	1.0–4.0	1 M NaClO_4_ EC:DEC (1:1)	105	25000	76% (10000)	[48]
10	Ordered microporous carbon//nitrogen‐doped ordered microporous carbon	Electrochemical (ex‐situ)	0–4.0	1 M NaClO_4_ EC:PC (1:1) 5.0% FEC	119	5807	85% (1800)	[55]
11	Microporous nitrogen‐rich carbon fibers//salt‐templated carbon	Electrochemical (ex‐situ)	0.5–4.0	1 M NaClO_4_ EC:PC:FEC (45:45:10, mass)	95	13000	90% (1000)	[341]
12	N‐doped carbon//activated carbon	Electrochemical (ex‐situ)	0.5–4.0	1 M NaPF_6_ EC:DEC (30:70, vol%)	224	10410	99.7% (600)	[342]
13	P,N‐doped interconnected carbon nanosheets with hierarchical porosity using KCl/Ice as dual‐templates//KOH‐treated N‐doped carbon nanosheets	Electrochemical (ex‐situ)	0–4.0	1 M NaClO_4_ EC:PC:FEC (1:1:0.05, vol%)	135	16100	88.6% (8000)	[177]
SIC–carbon composite negative electrode//carbon positive electrode								
14	Molybdenum carbide/carbon composite//porous carbon	Electrochemical (ex‐situ)	0–4.0	n.a.	50.2	10000	77.5% (1800)	[75]
15	Vanadium nitride quantum dots modified one‐dimensional carbon cages//activated N/F co‐doped carbon nanofiber cages	Electrochemical (ex‐situ)	0–4.0	n.a.	198.8	9100	73.5% (8000)	[124]
16	Sb‐carbon composite//polyaniline‐derived porous carbon	Electrochemical (ex‐situ)	1.0–4.0	1 M NaClO_4_ EC:DMC:EMC (1:1:1) 5.0% FEC	157	25000	80% (4000)	[67]
17	In_6_S_7_/nitrogen and sulfur co‐doped carbon hollow microspindles//polyaniline derived porous carbon	Electrochemical (ex‐situ)	1.0–3.8	1 M NaPF_6_ in DME	136.3	47466	68.5% (20000)	[343]

**FIGURE 43:**
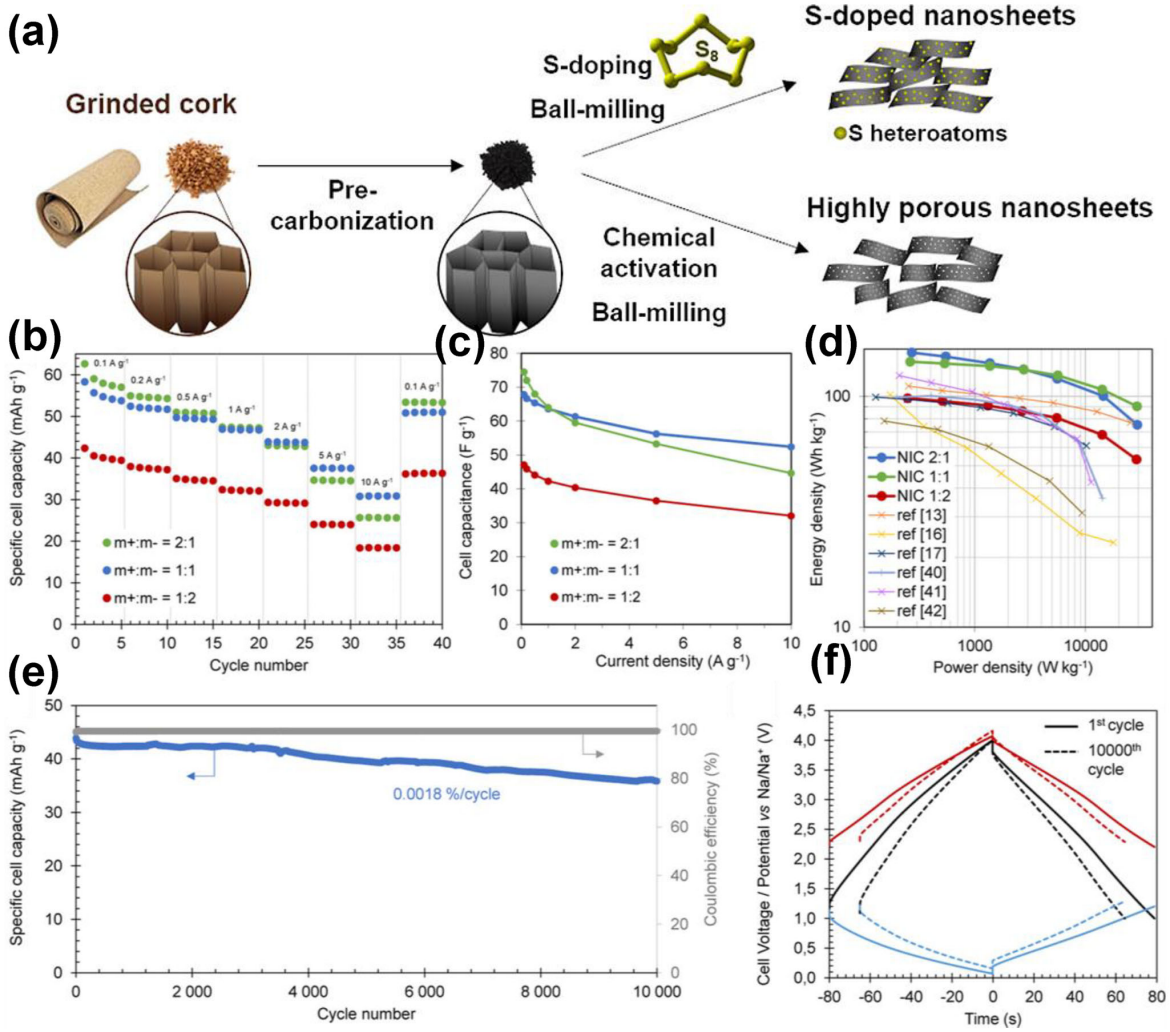
(a) Synthesis strategy of S‐doped nanosheet negative electrode and porous nanosheets from grinded cork. Rate capability based on (b) Specific capacity and (c) Specific capacitance. (d) Ragone plots of SIC at various negative electrode to positive electrode ratio (e) GDC cycling stability at 2 A g^−1^ for SIC, and (f) Electrode potential profiles recorded at the first (solid) and 10,000th (dashed) cycle. All experiments were performed in I M NaClO_4_ EC:DEC electrolyte. Reprinted with permission [171] under the terms of CC‐BY 4.0 license.

Moreover, other carbon negative electrode materials have been utilized for the development of dual carbon SIC. For instance, Casal et al. [171] synthesized sulfur‐doped negative electrode from ground cork and highly porous carbon sheets from cork biomass (Figure 43a). The process starts from the low thermal carbonization (600–750°C) of cork with sulfur as a heteroatom dopant and ball‐milled to form 2D carbon sheets from the 3D cellular structure of cork.

Porous carbon sheets were obtained by chemical activation of carbonized cork biomass precursor using NaOH/KOH. The negative electrode achieved a S‐doping level up to 27 wt.% that enhanced Na ion storage with discharge capacity from 300 to 550 mAh g^−1^ at 0.1 A g^−1^ and 55 to 140 mAh g^−1^ at 10 A g^−1^ while the ICE was 58%–76%. The porous carbon sheet delivered a capacity of 100–114 mAh g^−1^ (163–196 F g^−1^) at 0.1 A g^−1^ and 50–60 mAh g^−1^ (112–140 F g^−1^) at 10 A g^−1^, ascribed to their developed SSA and optimized micropore/mesopore distribution in I M NaClO_4_ EC:DEC electrolyte. Prior to SIC assembling, both positive and negative electrodes were independently pre‐cycled against sodium metal to minimize irreversible sodium losses. The developed full cell with a mass ratio of 2:1 (positive electrode to negative electrode) showed a capacity of 63 mAh g^−1^ (75 F g^−1^), while at a higher current density, the 1:1 SIC full cell retains 31 mAh g^−1^ (52 F g^−1^) capacity, as shown in Figure 43b and c. At the lowest current rate, the 2:1 and 1:1 SIC full cells deliver 155 and 141 Wh kg^−1^, respectively, and the cell with mass‐matched electrodes (1:1) shows the best performance under the high‐power regime, providing a high energy density of 90 Wh kg^−1^ at a high‐power density of 29 kW kg^−1^ (Figure 43d). The 1:1 SIC full cell showed 82% capacity retention after 10,000 cycles (Figure 43e) with high stability (Figure 43f). The performance of other carbon‐based negative electrode materials is summarized in Table 15.

**FIGURE 44:**
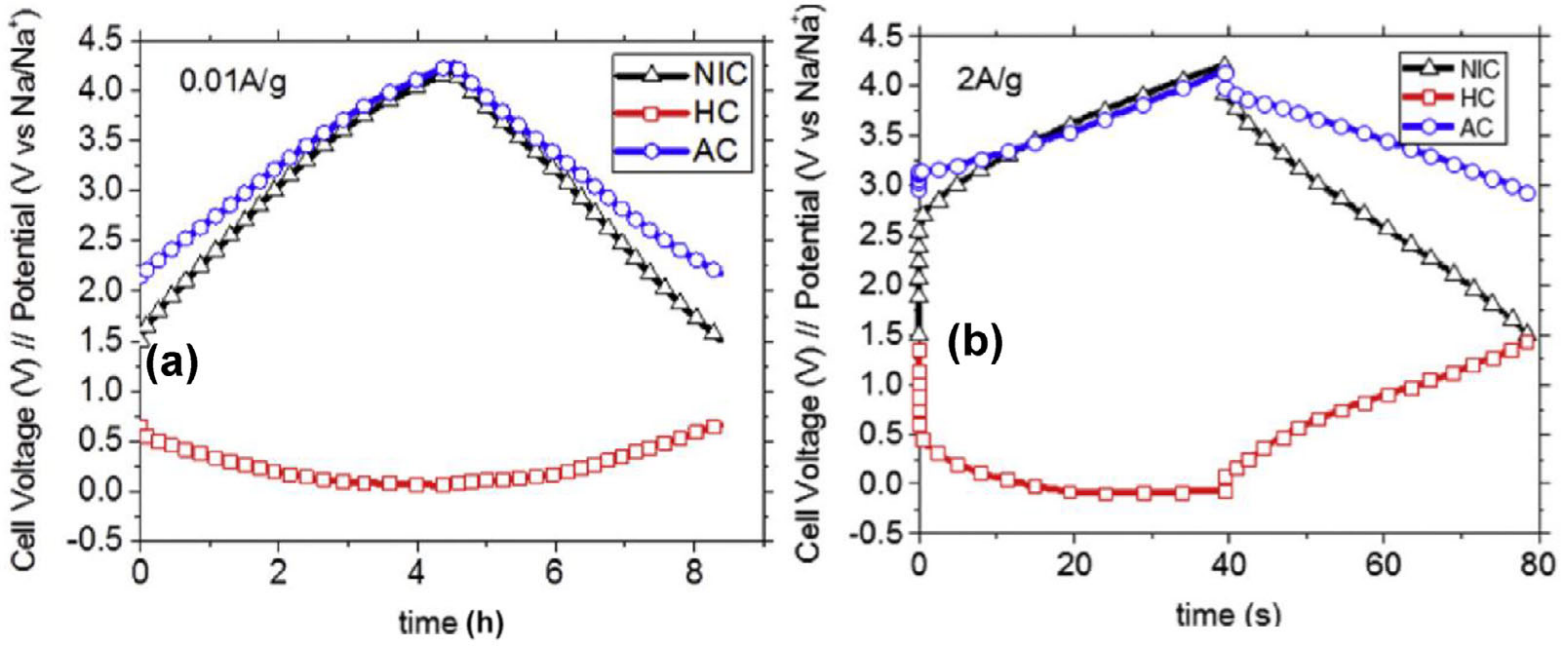
SIC full cell electrochemical performance involving hard carbon negative electrode and porous carbon positive electrode between 1.5 and 4.2 V in a three electrode Swagelok cell in 1 M NaFP_6_ EC:PC electrolyte (a) GCD at 0.01 A g^−1^ and (b) GCD at 2 A g^−1^. Reprinted with permission [33] Copyright (2017), Elsevier.

An interesting concept in dual carbon‐based sodium ion capacitor is the performance of the cell during high and low current regimes. Ajuria and co‐workers synthesized an HC negative electrode and a porous carbon positive electrode from raw olive pits [33]. The assembled dual carbon full cell featured an electrochemically pre‐sodiated hard carbon and porous carbon in a 1 M NaPF_6_ EC:PC electrolyte. HC was cycled five times between 0.002 and 2 V vs Na/Na^+^ at a C/10 rate, and a posterior cut‐off potential of 0.2 V vs Na/Na^+^ was set, while the AC was charged to a cut‐off potential of 4.2 V vs Na/Na^+^. As shown in Figure 44, the performance of the SIC full cell is shown at low and high current densities. At a low current density (0.1 A g^−1^), the HC negative electrode operates between 0.1 and 0.7 V, while the porous carbon works between 2.2 and 4.3 V (Figure 44a), offering efficient utilization of both the negative electrode and positive electrode. At high current density (2 A g^−1^), a change can be observed; there is a loss of symmetry in the HC electrode as a result of the high polarization (Figure 44b). The full cell discharge profile shows a steeper slope compared to that of the porous carbon positive electrode, which highlights that the HC electrode limits the performance of the cell. The HC electrode reached a maximum voltage swing of 1.5 V, which limits the potential window of the positive electrode to 1.2 V (3–4.2 V), thus reducing the full cell's overall capacity. In retrospect, the observations point out slow Na^+^ ion kinetics and restricted pore accessibility in HC during a high‐rate regime, which limits the overall performance of the full cell.

### 9.5 Carbon Composite Negative Electrode

Carbon composites are other negative electrode materials that have been explored in SIC. The combination of transition metal carbides (TMCs) with carbon materials has been reported in the SIC literature thanks to their high electrical conductivity and good stability for Na ion storage. For instance, Jia and co‐workers [75] fabricated Mo_2_C nanodisk arrays immobilized on porous carbon nanosheets as a negative electrode for SIC. The pre‐sodiated Mo_2_C/C‐2 negative electrode was coupled with a porous carbon positive electrode in the voltage range of 0.01 to 4.0 V and in NaClO_4_ electrolyte. The full cell achieved a high energy density of 50.2 Wh kg^−1^ at 200 W kg^−1^, maintaining 16.7 Wh kg^−1^ at an ultra‐high‐power density of 10,000 W kg^−1^, displaying 77.5% capacitance retention after 4000 cycles at 1 A g^−1^. The performance of other composite materials is summarized in Table 15.

## 10 Future Perspectives and SIC Application Areas

### 10.1 Future Perspectives

**FIGURE 45:**
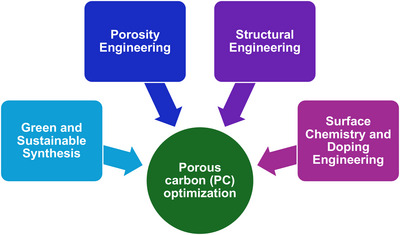
Schematic diagram of strategies for porous carbon optimization for improved electrochemical performance.

This section discusses the different strategies for porous carbon optimization (see Figure 45) for improved electrochemical performance.

#### 10.1.1 Green and Sustainable Synthesis

The use of green and sustainable precursors such as waste biomass (including agricultural and animal waste, wood, coconut shells, etc.) offers multiple benefits. It helps to limit the utilization of finite resources such as coal and petroleum products derived from carbon, thus helping in reducing the carbon footprint, aligning with the sustainable development goals. Furthermore, this sustainable precursor often follows a mild synthesis condition with green activation methods using a natural templated approach or benign chemical agents, which support the eco‐friendliness of the process. The obtained porous carbon keeps the important textural (high specific surface area) and structural (disorder and graphitic domains) and surface chemistry (optimized functional groups), which are crucial in attaining a high energy and power density in sodium ion capacitors. In addition, waste biomass could potentially enhance the scalability and cost‐effectiveness of SICs, leveraging the abundance and widespread availability of sodium metal resources, positioning them as a strong alternative to lithium‐ion capacitors. However, challenges such as impurities, toxic washing conditions, and material inhomogeneity remain. The adoption of green chemistry principles into the design of porous carbon electrodes can address environmental and ecological concerns for a sustainable energy future.

#### 10.1.2 Porosity Engineering

Porosity engineering is crucial for a porous carbon positive electrode for SICs. Precise control of the pore size, distribution, and volume plays a crucial role in enhancing the accessibility of active regions for electrolytic ion adsorption and the constraint regarding ions' solvation for fast ions transport towards the improvement of the storage discharge capacity. This can be potentially achieved using a novel approach that offers a high level of purity, providing the possibility for porosity engineering.

Biomass is abundant and cost‐effective, but controlling specific pore sizes is more challenging compared to biopolymers or synthetic precursors. Alternatively, a more rational design of biomass‐derived carbon is needed to produce carbons with controlled pore structures. However, the complexity and diversity of biomass complicate this task. Despite the challenges in achieving well‐controlled pores, many biomass‐derived carbons exhibit excellent performance. Emphasis should be placed on developing activation processes that are more sustainable and generate less hazardous waste. The synthesis of porous carbon with tuned porosity is essential for enhancing rate capability and cycling stability. An optimal SSA of 1500–3000 m^2^ g^−1^ balances high specific capacity (>100 mAh g^−1^) with ion accessibility, as SSA below 1000 m^2^ g^−1^ limits capacity, while above 3000 m^2^ g^−1^ increases diffusion resistance. The ideal pore distribution includes 50–70% micropores (0.8–1.5 nm) for maximizing ion adsorption, 20–40% mesopores (2–10 nm) to facilitate ion transport, and 5–10% macropores (>50 nm) as ion reservoirs, achieving a total pore volume of 1–2 cm^3^ g^−1^. This hierarchical structure ensures efficient electrolytic ion adsorption/desorption, with micropores tailored to electrolyte ion sizes (>0.8 nm). A well‐engineered pore size potentially can solve the challenge of volume expansion related to sodium ions, thus enhancing the electrode structural integrity. Pore architecture optimization provides support towards the quest for cost‐effective and environmentally friendly high‐power devices.

#### 10.1.3 Structural Engineering

Structural engineering is also an important aspect in the synthesis of porous carbon, particularly with striking a balance between the disorder or defects and graphitic domains towards achieving a high electronic conductivity, discharge capacity, and long‐term cycling. From the point of view of disorder, a highly disordered carbon is made up of a large range of defect sites with increased interlayer spacing, which is beneficial for ion adsorption, favoring improved discharge capacity. Active sites and defects, including edge and basal plane defects (stacking faults, dislocations, and atom vacancies) that are typically measured via Raman spectroscopy, and new methods such as TPD and ASA experiments, are now regarded as a means to improve the quantum capacitance of porous carbon, which improves the total capacitance of the porous carbon. On the other hand, excessive disorder could impact electrical conductivity, causing unexpected failures during long‐term cycling. The presence of graphitic domains helps improve electrical conductivity and capacity retention during long‐term cycling. Thus, striking a balance between defects and graphitic domains is imperative. This can be achieved by using a tailored synthesis approach such as controlled thermal treatment step, templated synthesis method, and or heteroatom doping. This will help researchers design porous carbons with a hybrid architecture: disorder regions to boost discharge capacity and graphitic domains to extend long‐term cycling. In retrospect, porous carbon's structural engineering is crucial towards advancing the field of sodium ion capacitors.

#### 10.1.4 Surface Chemistry and Doping Engineering

The surface chemistry, heteroelement doping, and the active sites are other critical factors affecting the performance of porous carbon electrodes. From the surface chemistry and dopants perspective, surface functionalities based on oxygen, nitrogen, or sulfur present on carbon surfaces can enhance the electrochemical performance of porous carbon. They have shown to improve the wettability of carbon electrodes by the electrolyte and offer pseudocapacitive contribution through rapid reversible redox and faradaic reactions, particularly the phenolic and carbonyl groups. Heteroelement‐doped carbonaceous materials have also been tipped to be a way to achieve “nobility” in carbon materials, that is, materials that are resistant to oxidation. On the other hand, excessive surface functionalities (carboxylic acid groups) could lead to low conductivity and loss of performance.

#### 10.1.5 Binder, Mass Loading, and Electrolyte

On the level of the electrode, the future of SICs depends on addressing critical challenges related to sustainable binders, high mass loading, electrolyte safety, and scalability to commercial applications. These advancements are essential to enhance the performance, environmental compatibility, and practical viability of SICs in energy storage systems. To tackle environmental and recycling concerns, sustainable, fluorine‐free, and aqueous‐formulated binders such as CMC, PAA, and PVP are vital. These binders must be carefully engineered to prevent porosity blockage, ensure compatibility with high mass loading, and support pre‐sodiation agents, particularly sacrificial salts used in porous carbon positive electrodes. Developing binders that meet these requirements will improve electrode performance and facilitate sustainable recycling processes. Achieving high areal mass loading, exceeding 10 mg cm^−2^, is crucial for SICs to meet commercial energy density demands while maintaining efficient ion and charge transport. This requires the development of porous carbon electrodes with engineered structures, such as binder‐free electrodes featuring well‐developed porosity. These designs will enable high mass loading without compromising the electrochemical performance of the electrodes. Electrolyte safety and innovation are equally critical for advancing SICs. While NaClO_4_ is a cost‐effective electrolyte, its explosive risk necessitates safer alternatives like NaPF_6_ and NaTFSI, which offer improved temperature stability and better SEI formation. Future research should prioritize exploring new electrolyte options, such as ionic liquids, fluorine‐free electrolytes, and hybrid organic electrolytes, to ensure high ionic conductivity, appropriate additives to stabilize SEI and cycle life, and a wide electrochemical window, further enhancing the safety and efficiency of SICs.

#### 10.1.6 In‐Situ Monitoring and Diagnostics

In‐situ monitoring via advanced characterization is essential for the detection of compatibility issues of porous carbons in SICs. Techniques such as in‐situ spectroscopy, microscopy, and diffraction allow for real‐time analysis and observation of electrode behavior and degradation mechanisms. The integration of experimental data with computer models requires deeper insight into the degradation mechanism, providing guidance towards mitigating performance loss. In‐situ techniques are also effective for uncovering structural changes and chemical composition variations in carbon electrode materials. By combining advanced in‐situ methods with computational simulations, researchers can comprehensively track carbon electrodes from material prediction and synthesis to electrochemical performance and failure analysis.

Overcoming these limitations of porous carbon‐based positive electrode and dual carbon SIC, extensive research is needed to enhance the performance of porous carbon and practical scalability. Innovative precursor and synthesis routes, advanced characterization, and optimized electrode and cell setup offer promising solutions. Addressing these challenges allows for achieving high performance, cost‐effective SIC towards their advancement up to commercial scale.

### 10.2 SIC Application Areas

**FIGURE 46:**
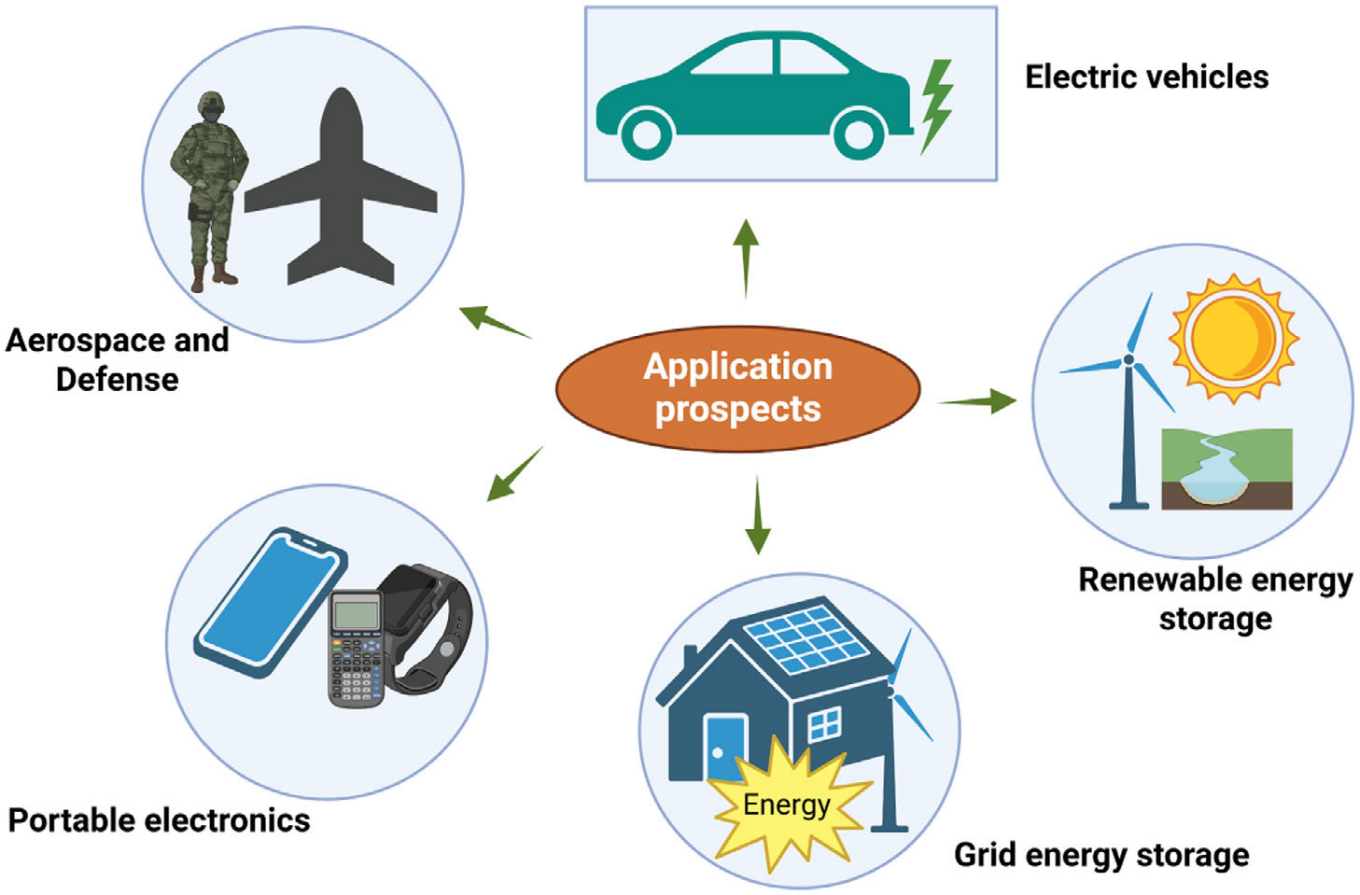
Potential application areas of SICs. Created by the authors with biorender.com.

Although SIC is still at an infant age, research work focusing on the development of electrodes for SIC is on the rise. To this end, we present some possible application areas of dual carbon SICs as shown in Figure 46.

1. **Electric vehicles**: Porous carbon electrodes based on SIC can be used for energy storage in electric vehicles, not only as a start‐stop energy recovery during braking. SIC has the potential to be used in place of conventional batteries. Capacitor‐based city buses are already running in major cities like Shanghai/Ningbo, China, and Turin/Bergamo, Italy. The prospect of dual carbon, based on delivering both high energy and power density, will be a game‐changer in the automotive industry.
2. **Renewable energy storage**: The rising increase in the demand for renewable energy sources due to the growing population, fast‐paced industrialization, and reduction of greenhouse gas emissions, dual carbon‐based SICs have an important role in energy storage. They can serve as backup power systems to renewable sources like wind, solar, and geothermal, thanks to their potentially high energy and power density.
3. **Portable electronics**: With the increasing demand for mobile devices, especially with the increasing social media usage and artificial intelligence, dual carbon‐based SIC offers durable power systems for smartphones, tablets, smart watches, computers, and laptops.
4. **Grid energy storage**: Dual carbon‐based SICs are interesting devices that can be utilized for grid energy storage, offering a reliable and stable energy supply for suburbs, cities, and regions.
5. **Aerospace and defense**: SICs can also provide backup power for aerospace and defense‐related applications, such as radars, satellites, and space probes. Their long‐term stability and strong flexibility make them interesting for detached and rough terrains.

## 11 Conclusions

This review comprehensively evaluates SICs in comparison to conventional batteries and electrochemical capacitors, highlighting their potential for achieving superior energy and power densities. The performance of SICs is limited by the low discharge capacity of commercial activated carbon, prompting extensive research into advanced porous carbon materials derived from biomass, biopolymers, and synthetic precursors. The properties of these porous carbons, encompassing structure (graphitic interlayer spacing, defects, and domain arrangement), porosity (specific surface area, pore size, and volume), morphology (particle shape and size), and surface chemistry (dopants and functional groups), are significantly influenced by precursor type and synthesis conditions, such as pyrolysis temperature, gas flow rate, and synthesis methods. Techniques like pre‐treatment (e.g., washing, grinding, hydrothermal carbonization) and post‐treatment (e.g., CO_2_/KOH/templated activation, heteroatom doping) enable precise tuning of these properties to enhance SIC performance.

Based on the knowledge gained from all these literature studies, an optimal SSA of 1500–3000 m^2^ g^−1^ balances high specific capacity (>100 mAh g^−^^1^) with effective ion accessibility. SSAs below 1000 m^2^ g^−^^1^ restrict capacity, while those above 3000 m^2^ g^−^^1^ increase diffusion resistance. An ideal pore distribution includes 50%–70% micropores (0.8–1.5 nm) for improved ion adsorption, 20%–40% mesopores (2–10 nm) for efficient ion transport, and 5%–10% macropores (>50 nm) as ion reservoirs, yielding a total pore volume of 1–2 cm^3^ g^−1^. This hierarchical pore structure supports efficient ion adsorption and desorption, with a minimum pore size of 0.8 nm, essential for accommodating PF_6_^−^ ions due to thermodynamic limitations. Optimized SSA, pore sizes, surface defects, and chemical functional groups enhance electrode capacity by aligning with electrolytic ions, increasing quantum capacitance, and enabling pseudocapacitance. However, these characteristics may reduce capacity retention and cause electrode degradation, which can be addressed by incorporating graphitic domains and selective heteroatoms. More research is required to better understand ageing, CEI, in order to extend the cycle life.

Electrochemical insights underscore the scalability potential of SICs. A typical electrode formulation consists of 80% active material, 10% binder, and 10% conductive carbon. Critical for large‐scale applications are high electrode mass loading (>10 mg cm^−^^2^), sustainable water‐based binders (e.g., carboxymethyl cellulose, polyacrylic acid), and safe electrolytes (e.g., NaPF_6_ or fluorine‐free alternatives) operating within a 2.2–4.2 V voltage range. The review examines charge storage mechanisms, such as EDLC and pseudocapacitance, alongside the effects of ion solvation and pre‐sodiation. The capacitive mechanism is predominant and matching the pore size with the ions’ ones is of prime importance, while pseudocapacitive reactions further improve the capacitance by faradaic charge storage.

The ex‐situ negative electrode pre‐sodiation remains the predominant method for full‐cell SICs; its commercial unfeasibility necessitates exploration of alternatives like sacrificial salts. Optimizing negative electrode materials, such as hard carbon, is essential for the SIC full cell to enhance Na^+^ ion kinetics and pore accessibility under high‐rate conditions, which currently limit performance. These improvements are crucial for surpassing the existing maximum energy and power densities of approximately 200 Wh kg^−1^ and 20 kW kg^−1^, respectively. Overall, this review provides a comprehensive framework for developing advanced capacitive electrodes, guiding the advancement of next‐generation energy storage solutions.

## Author Contributions

**Ademola Adeniji**: conceptualization, investigation, data curation, formal analysis, resources, writing – original draft, writing, review, and editing. **Adrian Beda**: conceptualization, supervision, writing, review, and editing. **Camélia Matei Ghimbeu**: conceptualization, project administration, funding acquisition, supervision, writing, review, and editing.

## Funding

French National Research Agency, ANR (22‐PEBA‐0003, PEPR HipoHyBat) via the France 2030 Program of the French government.

## Conflicts of Interest

The authors declare no conflicts of interest.

## Data Availability

The authors have nothing to report.
